# Analyses of 202 plastid genomes elucidate the phylogeny of *Solanum* section *Petota*

**DOI:** 10.1038/s41598-019-40790-5

**Published:** 2019-03-14

**Authors:** Binquan Huang, Holly Ruess, Qiqi Liang, Christophe Colleoni, David M. Spooner

**Affiliations:** 1 State Key Laboratory for Conservation and Utilization of Bio-Resources in Yunnan/School of Agriculture, Yunnan University, Kunming, China; 2 Department of Plant Sciences, University of Oxford, Oxford, UK; 3 Vegetable Crops Research Unit, USDA-Agricultural Research Service, Department of Horticulture, University of Wisconsin, Madison, USA; 4 Novogene Bioinformatics Institute, Beijing, China; 5 University of Lille, CNRS, UMR, 8576-UGSF Lille, France

## Abstract

Our paper analyzes full plastid DNA sequence data of 202 wild and cultivated diploid potatoes, *Solanum* section *Petota*, to explore its phylogenetic utility compared to prior analyses of the same accessions using genome-wide nuclear SNPs, and plastid DNA restriction site data. The present plastid analysis discovered the same major clades as the nuclear data but with some substantial differences in topology within the clades. The considerably larger plastid and nuclear data sets add phylogenetic resolution within the prior plastid DNA restriction site data, highlight plastid/nuclear incongruence that supports hypotheses of hybridization/introgression to help explain the taxonomic difficulty in the section.

## Introduction

The main phylogenetic utility of next generation sequencing techniques is to produce data quantities far above that needed for well-resolved phylogenies. This is certainly true with the plastid molecule that has proven useful as a phylogenetic marker beginning in the 1980s. The first plastid phylogenetic study by Palmer and Zamir^1^ used total plastid DNA restriction site data in *Solanum*. Hosaka *et al*.^2^ applied this technique to *Solanum* section *Petota*, but the technique suffered from overlapping bands making homology detection difficult. Refinements to this technique used filter hybridization using radiolabeled or chemiluminescent cloned heterologous probes spanning the plastid molecule, allowing for more accurate interpretations of the homology of digest patterns^3^. Jansen and Ruhlman^4^ provided a review of the many advantages of plastid DNA as a phylogenetic marker and reported the public availability of 200 plastid genomes that presently (September 2018) has grown to nearly 3000 for eukaryotes (https://www.ncbi.nlm.nih.gov/genome/browse#!/organelles/Viridiplantae).

Despite these advantages of plastid DNA as a phylogenetic marker, incongruence between plastid and nuclear genes are common in phylogenetic studies throughout the angiosperms, with plastid phylogenies often the most discordant relative to other molecular markers^5^. This led to phylogenetic studies using both plastid and nuclear markers. The value of generating phylogenies from both nuclear and plastid sequences is that hard incongruence can be quite informative, suggesting such evolutionary processes as “plastid capture” where incongruence can be caused by a history of hybridization between plants with differing plastid and nuclear genomes^6^, and backcrossing to the paternal parent but retaining the plastid genome that is (typically) maternally inherited. Other possible processes that can lead to such incongruence are gene duplication^7^, horizontal gene transfer^8^ and incomplete lineage sorting^9^. The relative structural conservatism, varying rates of DNA changes in different parts of the molecule, and plastid/nuclear phylogenetic incongruence has shown the plastid molecule to be extremely useful at different phylogenetic levels, but to be most useful when used with corroboration with nuclear data, as done in the present paper.

The taxonomy of wild and cultivated potatoes has long been controversial, with different authors providing varying hypotheses on the number of species and their relationships. In total, there are 494 epithets for wild taxa and 626 epithets for cultivated taxa, including names not validly published^10^. Hawkes^11^ provided the first modern conspectus of section *Petota* and synonymized many species, ending with his last treatment in 1990 where he recognized 232 species, partitioned into 21 taxonomic series. Spooner *et al*.^12^ provided the last conspectus of the section and recognized 107 wild species and four cultivated species partitioned into three main nuclear clades but not recognized as series, basing their decisions on a variety of morphological and molecular datasets including DNA markers (e.g., AFLPs, microsatellites), DNA sequence data of single and multiple nuclear orthologs that documented many polyploids to be allopolyploids among the three clades, and plastid DNA restriction site data^13–16^.

**Figure 1:**
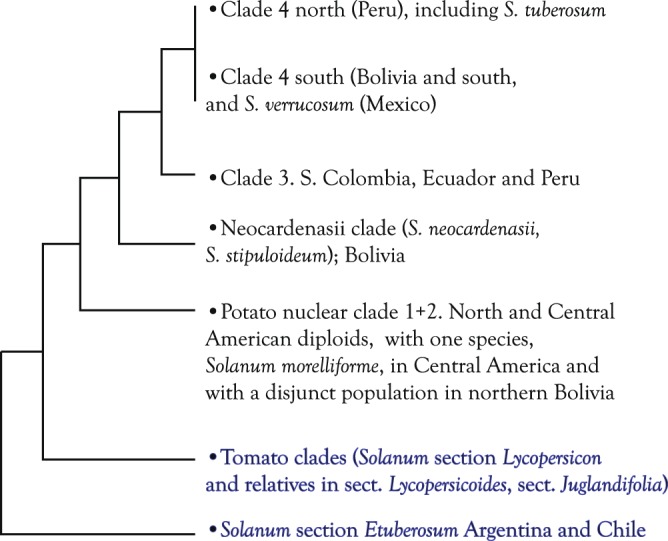
Cladistic relationships of the diploid species of wild potatoes (black) and immediate outgroups (blue) in tomatoes (*Solanum* section *Lycopersicon*) and *Solanum* section *Etuberosum*.

The present study reports the first whole-genome plastid DNA sequence phylogeny of section *Petota*, using 202 diploid accessions from all major clades of the section except a small clade of two diploid species that was discovered after this study was initiated, namely the “Neocardenasii clade” containing *S*. *neocardenasii* Hawkes and Hjert. and *S*. *stipuloideum* Rusby^17^ (Fig. 1). Genomes have been designated for most of the species in section *Petota* with most species in clade 4 (A genomes), clade 3 (P genomes), the Neocardenasii clade (unknown genomes), and clade 1 + 2 (B genomes). The present study does not include the polyploid species of the section, many of which are of allopolyploid origins^18^. It complements the recent study of Li *et al*.^19^ who investigated these same accessions with 66,666 genome-wide nuclear SNPs. The purpose of this study is to examine concordance of our new whole-genome plastid data with the nuclear data of the same accessions^19^ and with the prior plastid phylogeny based on restriction site data.

## Results

### Sequencing and assembly of potato plastid genome

**Figure 2:**
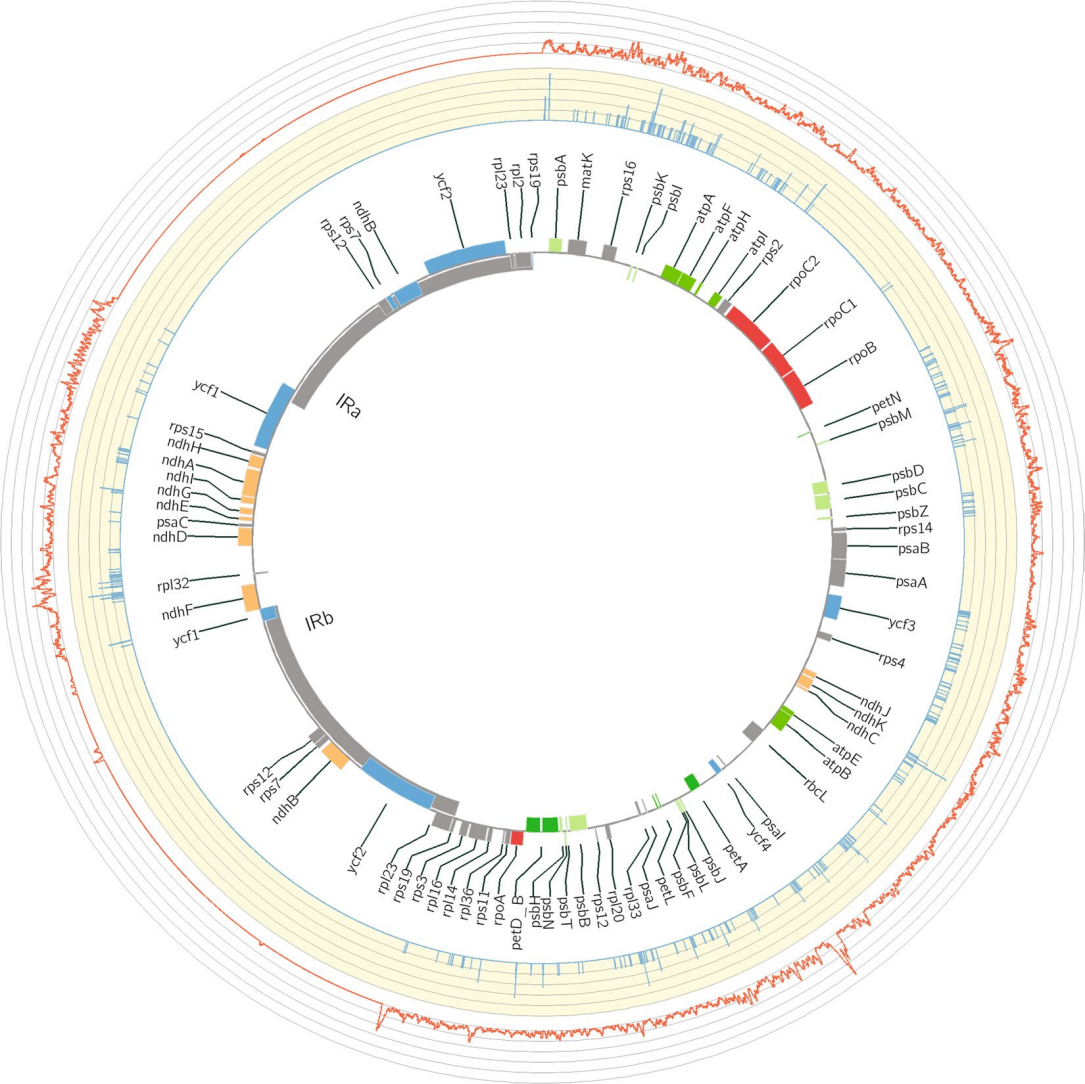
Circular variation map of potato plastid genome. The innermost circle is the plastid genome map showing its corresponding genes; the two inverted repeat regions (IRA and IRB) divided the large (LSC) and small (SSC) single regions, respectively; the relative density of SNPs is indicated in red and the density of indels in blue.

We obtained 202 complete plastid genomes with lengths ranging from 155,231 bp (*S*. *polyadenium*) to 155,696 bp (*S*. *gourlayi*), and an average read coverage depth of 403 (*S*. *andreanum*) to 4,050 (*S*. *gourlayi*) (Supplemental Table 1). All of the plastid genomes were composed of a single circular double-stranded DNA molecule, and they displayed the typical quadripartite structure of angiosperm plastid genomes, consisting of a pair of IRs ranging from 25,577–25,634 bp, separated by the LSC (85,656–86,107 bp) and SSC (18,325–18,443 bp) regions (Supplemental Table 1; Fig. 2). As previously noted^20^, there was an inverse relationship between the coverage depth and GC content, present in the inverted repeat region, likely the result of coverage bias in high GC regions, which has been known in the Illumina platform^21^ (Supplemental Fig. 1).

### Variation in the potato plastid genome

The aligned length of the entire plastid molecule was 158,949 nucleotides. Using the strategy of variant calling as detailed below we identified 5,232 high quality variant positions, including 4,803 SNPs. The SNPs are uniformly distributed in the plastid genome except with fewer in the inverted repeats (Fig. 2), which indicated the conservation of the IR region and is consistent with the observation in other species^21–24^. Approximately 52% SNP positions (2,540) were located in intergenic regions. The remaining SNPs affected 52 genes, some of which showed remarkably high SNP densities, such as the *ycf1* genes including 381 SNPs in exons, the *rpoC2* genes including 119 SNPs in exons and the *ndhF* gene including 84 SNPs in exons (Supplemental Tables 2 and 3). In addition, 429 indels were detected, including 254 insertions (59%) and 175 deletions (41%), of which 66 locate in genes and 363 (84%) locate in intergenic regions (Supplemental Tables 3 and 4). The insertions range from 1 bp to 30 bp, however, most of them were shorter than 20 bp (76% range from 1 to 10 bp, and 16% range from 11 to 20 bp). For the deletions, approximately 79% range from 1 to 10 bp and approximately 14 range from 11 to 20 bp. Of these 429 indels, only 20 locate in exons.

### Phylogenetic analyses

The aligned data set, excluding one of the two inverted repeat regions (deleting IRB) that provided duplicative information was 133,226 characters long. Maximum parsimony analysis of these data produced 5000 (set as the upper limit) equally parsimonious 6785-length trees with 2723 parsimony informative characters, consistency index 0.7298 and 0.6114, with and without autapomorphies, respectively, retention index of 0.9164, and rescaled consistency index of 0.6888.

**Figure 3:**
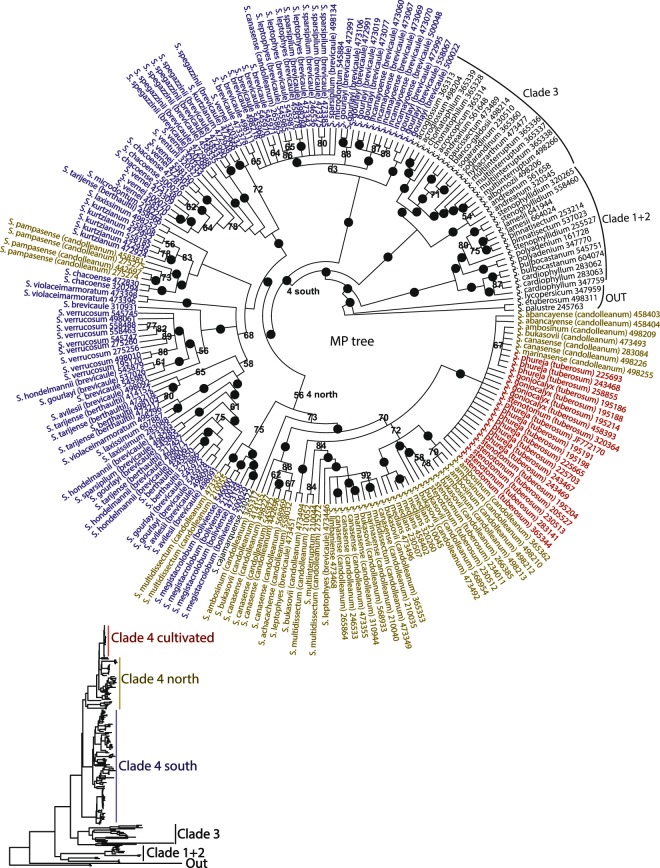
The phylogenetic relationship of all clades of section *Petota* as determined by strict consensus maximum parsimony (MP) analysis of 5000 equally parsimonious trees; the bottom figure is a phylogram of one of these trees to show relative branching lengths of the clades; the nodes with black dots are supported by bootstrap support values ≥ 90% and those from 50% to 89% shown numerically; the outgroup, clades 1 + 2 (see text) and clade 3 are indicated by black text and bracketed with their clade names, clade 4 south (Bolivia and south) wild species by blue text, clade 4 north (Peru) by brown text, and the cultivated species in red text; the blue color of the four accessions of *S*. *boliviense* (prior name *S*. *megistacrolobum*), the brown color of the four accessions of *S*. *pampasense*, and the brown color of the wild species in the cultivated species clade represents their former placement in the nuclear data^19^.

Figure 3 presents the maximum parsimony (MP) strict consensus tree with bootstrap support over 50% and with the main clades labelled. The phylogenetic results (1) partition members of section *Petota* into outgroup, clade 1 + 2, clade 3, and clade 4. This differs from the plastid DNA restriction site data that placed clade 1 and clade 2 as sister clades. (2) Clade 4 is partitioned into two subclades generally with a geographic component, (a) cultivated species and wild species from the north (Peru), and (b) wild species from the south (Bolivia south to Argentina, Chile, Paraguay and Uruguay). However, *S*. *megistacrolobum* (*S*. *boliviense*), placed in clade 4 southern species, and *S*. *pampasense* (*S*. *candolleanum*), placed in clade 4 northern species in the nuclear data^19^, are switched in position with the plastid data as indicated in Fig. 3. (3) The northern members of the *S*. *brevicaule* complex are supported as the progenitors of cultivated potato. (4) The previously defined *S*. *tuberosum* subspecies *phureja* and *stenotomum* are not partitioned into separate clades. (5) *Solanum verrucosum*, the sole A-genome diploid species from Mexico, is firmly placed into the southern South American subclade of clade 4. (5) Many of the species placed in synonymy of the wild species in the *S*. *brevicaule* complex (the new names, indicated in parentheses, being *S*. *candolleanum* in clade 4 north and *S*. *brevicaule* in clade 4 south) are not supported as monophyletic, supporting the new synonymy. However, some are supported as monophyletic, such as *S*. *ambosinum*, *S*. *incamayoense*, *S*. *spegazzinii*, and *S*. *pampasense*.

Supplemental Fig. 2 presents the results of maximum likelihood (ML) analysis. The ML analysis and MP analysis had all four clades containing the same species, including the placement of *megistacrolobum* in clade 4 northern species, and *S*. *pampasense* in clade 4 southern species, in contrast to switched placements in the nuclear data^19^. Species within all of the major clades (1 + 2, 3, 4 north, 4 south) of the plastid data, however, are sometimes in different topological arrangements in areas of the tree with low bootstrap support.

A notable result of the plastid data is the placement of *S*. *cajamarquense* in the clade 4 south in both analyses. The nuclear data placed *S*. *cajamarquense* in its expected position in clade 3 by SVD quartets analysis yet in clade 4 north by ML analysis.

## Discussion

### Phylogenetic congruence and incongruence of the plastid to the prior nuclear results of the same accessions

The present plastid data partition members of section *Petota* into outgroup, clade 1 + 2, clade 3, and clade 4; [mostly] separate members of clade 4 into subclades of cultivated, wild north (Peru), and wild south (Bolivia and south); support the northern members of the *S*. *brevicaule* complex are the progenitors of cultivated potato^25^; fail to separate previously defined *S*. *tuberosum* subspecies *phureja* and *stenotomum* into clades, supporting placing these names into synonymy^10^; place *Solanum verrucosum*, the sole A-genome diploid species from Mexico, firmly in the southern South American subclade of clade 4; support much of the recent synonymy of the wild species in the *S*. *brevicaule* complex (the new names being *S*. *candolleanum* in clade 4 north and *S*. *brevicaule* in clade 4 south)^12^.

There are some elements of discordance to the nuclear data of Li *et al*.^19^. Four accessions of *S*. *boliviense*, placed in the wild north clade with plastid data and wild south clade with nuclear data, and the four accessions of *S*. *pampasense* placed in the wild south clade with plastid data and wild north clade with nuclear data. Despite reconstructing the same main clades as the nuclear data the immediate sister group relationships of many species in clade 4 are often are quite different between data sets. This is certainly a result of reduced topological structure (greater polytomies and decreased bootstrap support) in the clades in the plastid relative to the nuclear trees. Although this reduced structure is present throughout branches of plastid clade 4, it is most evident in the cultivated subclade 4 that forms a nearly complete polytomy. These discordant results of individual species and accessions failing to cluster, and of reduced bootstrap support in clade 4 may have three causes. First, the nuclear data set of Li *et al*.^19^ is much larger, with the trees built using 66,666 SNPs vs. the plastid trees built on 2036 parsimony informative characters (mostly SNPs but some insertion/deletion characters), just 3.1% of the nuclear data; more than an order of magnitude less. Despite this fact, the present plastid data are more than an order of magnitude more than the prior-generation plastid DNA restriction site data^16^. Second, comparative studies of concordance and discordance in phylogenies built from various molecular markers^5^ have shown that plastid phylogenies are the most discordant relative to other molecular markers, caused by various reasons. Third, these discordances, despite the lower relative quantity of plastid to nuclear data, may reflect real phylogenetic signal, supporting a history of hybridization and introgression in section *Petota*.

*Solanum cajamarquense* presents the best-supported example of introgression in the present study, considering the nuclear data of the same accessions in Li *et al*.^19^. These nuclear data alone^19^ were highly suggestive of introgression in that *S*. *cajamarquense* was placed in its expected position in clade 3 based on SVD analysis, versus its placement in clade 4 by ML analysis. *Solanum cajamarquense* has many of the morphological and distributional characters supporting its placement in clade 3 (moniliform tubers and occurrence in northern Peru^26^). Its placement in clade 4 by plastid data strongly supports a hybrid origin or a history of introgression. In another example, a comparison of the plastid and nuclear data also suggests hybridity. For example, all seven accessions of *S*. *berthaultii* (some previously identified as *S*. *tarijense*) form a well-supported ≥90% bootstrap clade with the nuclear data, but not so with the plastid data. Indeed, section *Petota* has many morphological and biological patterns suggestive of widespread hybridization and introgression^27^. There are many other elements of discordances of both of these studies to prior taxonomies of Hawkes^28^ and others are discussed in Spooner *et al*.^12^.

## Materials and Methods

### Sample preparation and sequencing

Total DNA of 201 wild and cultivated potatoes and outgroups (Supplemental Table 5) was sequenced using the same short reads of Illumina HiSeq. 4000 (2 × 150 bp) as in Li *et al*.^19^ and the reads were pre-processed as described in that paper. The nuclear data of Li *et al*.^19^ had the same accessions except for *S*. *lycopersicum* (https://www.ncbi.nlm.nih.gov/; DQ347959.1) that we added here, bringing our total database to 202 accessions. Briefly, a total of 1.5 ug DNA of each accession of 202 wild and cultivated potatoes and outgroups (Supplemental Table 5) were used to make DNA sequencing libraries, and each library was labeled by index. During the sequencing process, one HiSeq run includes 8 lanes and each lane can produce 100 G data, and therefore a few samples were mixed for sequencing in each lane and were separated by index barcodes. These libraries were sequenced to an average of >12 X genome coverage depth, which produced 1.5 T data in total.

### Reference assisted de novo assembly

High quality reads were mapped to the plastid reference sequence (JF772170, *Solanum tuberosum* isolate DM1-3-516-R44 as downloaded from NCBI (https://www.ncbi.nlm.nih.gov/) with BWA-MEM version 0.7.15^29^). The resulting SAM file was converted to BAM format (view), sorted (sort), and filtered for both reads in pair mapping (view -F 8) using SAMtools version 0.1.19^30^. Reads that mapped to the plastid were pulled with Picard SamToFastq version 1.127 (https://broadinstitute.github.io/picard/), and de novo assembled with ABySS (k = 64)^31^. Contigs with lengths greater than 1000 bp were placed in order and oriented (as compared to the reference) using the MUMmer package, NUCmer version 3.1^32^. From this information, the contigs were manually concatenated and gaps between large contigs were manually filled in by using overlapping sequences of the smaller contigs (<1000 bp) of the highest coverage.

### Plastid correction for assembly errors

We mapped the reads to the de novo assembled plastid (one inverted repeat only due to reads being filtered out if they map to more than one location) using BWA-MEM version 0.7.15^29^. We converted the SAM file to BAM, sorted, and filtered with SAMtools version 0.1.19^30^. PCR duplicates were marked with Picard MarkDuplicates version 1.127 (https://broadinstitute.github.io/picard/). We identified SNPs and small insertions and deletions (indels) with GATK version 3.7-0^33^ using the following pipeline: RealignerTargetCreator (standard protocol), IndelRealigner (standard protocol), and UnifiedGenotyper (-ploidy 2 -glm BOTH -baq CALCULATE_AS_NECESSARY -dt NONE)^34,35^. In this particular case, we used UnifiedGenotyper instead of HaplotypeCaller because of the plastid’s high coverage; HaplotypeCaller downsamples reads, leading to a problem where SNPs that are from similar sequence regions in the mitochondria are oversampled and can lead to errors in SNP calls. We corrected variants in the sequence using a custom script. Soft clips were detected with bb.softclip (github.com/dsenalik/bb/blob/master/bb.softclip), manually reviewed, and corrected by identifying sequence of the reads in this region and filling in the gap with the correct contig (from the de novo assembly step). We repeated the process to check for errors until no more variants or soft clips occurred. The final coverage of the plastid regions was determined using GATK DepthofCoverage (standard protocol)^33^. Complete scripts can be found at github.com/HollyRuess/Solanum-Plastid-Assembly.

### Alignment and annotation

Each region of the plastid genome (LSC, SSC, and IR) was aligned separately using MUSCLE version 3.8.1551 (-diags -maxiters 1)^36^, and subsequently concatenated in a PAUP*^37^ compatible NEXUS file format^38^. Sequence alignments were viewed and corrected to minimize gaps using Mesquite version 3.31^39^. We checked and transferred annotations following Spooner *et al*.^20^. Annotations from the potato plastid reference genome (JF772170) were transferred to the aligned sequence. Corrections prior to transfer were made to the following genes: atpF complement(join(11899.12309,12988.13146)), trnG-TCC complement(join(9142.9164, 9856.9903)), and trnM-CAU complement(88023.88096) and 153327.153400.

### SNP/Indel calling

The SNPs/Indels of the plastid genome were called by using Genome Analysis Toolkit (GATK) UnifiedGenotyper version 3.8^33^. High-quality SNPs with the filter expression “QD < 4.0 || FS > 60.0 || MQ < 40.0” and “GQ < 20” and InDel with the filter expression “FS > 200.0 ||ReadPosRankSum < −20.0 || InbreedingCoeff < −0.8” were retained for subsequent analyses.

### Phylogenetic analyses

We analyzed the plastid sequence data of all 202 diploid accessions with two phylogenetic analyses, rooting all trees with *Solanum etuberosum* and *S*. *palustre*, and deleting inverted repeat (IR) B from analyses as it provided redundant information to IR A: (1) MP using the program PAUP* version 4.0a147^37^, with all characters treated as unordered and weighted equally^40^, and (2) ML using the program RAxML version 8.0.0^41^, using the GTR + G model and estimating individual alpha-shape parameters, GTR rates, and empirical base frequencies for each individual gene, and running 1000 nonparametric bootstrap inferences. We found the most parsimonious trees using a heuristic search^42^ by generating 100,000 random-addition sequence replicates and one tree held for each replicate. Branch swapping used tree–bisection–reconnection (TBR) retaining all most parsimonious trees. Then we ran a final heuristic search of the shortest trees from this analysis using TBR and MULPARS. We estimated bootstrap values^43^ using 1000 replicates setting maxtrees at 1000 and using TBR and MULPARS, and viewed the resulting trees in FigTree version 1.4.0 (http://tree.bio.ed.ac.uk/software/ figtree/). In order to compare old and new taxonomies, the taxon labels include, when appropriate, the older names accepted by Hawkes^28^ and in parentheses the new names^12^. Using this system, we examined 53 species using the prior taxonomy of Hawkes^28^ and others and 36 species using new taxonomy of Spooner and collaborators^12^.

### Data Records

The sequence data are deposited in the NCBI Sequence Read Archive (SRA) under project number PRJNA394943 and the SNP files as detailed in Supplementary Table 1.

## Data Availability

Supplementary information accompanies this paper at http\\www.xx as detailed in Supplemental File 1.

## Supplementary information

Supplementary Tables 1–5; Figures 1,2.
