# Supplementary material for: Analyses of 202 plastid genomes elucidate the phylogeny of Solanum section Petota
Source: Sci Rep. 2019 Mar 14;9:4454. doi: 10.1038/s41598-019-40790-5 (PMC6418237; doi:10.1038/s41598-019-40790-5)

Supplemental files for:

Analyses of 202 plastid genomes elucidate the phylogeny of *Solanum* section *Petota*

Binquan Huang[1,2], Holly Ruess, Qiqi Liang, Christophe Colleoni, and David M. Spooner

Supplemental Table 1. GenBank deposition numbers, large single copy (LSC), small single copy (SSC) and inverted repeat (IR) length and coverage statistics, coverage statistics, and inverted repeat junction types (Fig. 2) of the 202 accessions of wild potato and outgroups examined here.

Supplemental Table 2. Summary of SNPs of the potato plastid genome.

Supplemental Table 3. Statistics of genes in the plastid potato genome containing SNPs and indels.

Supplemental Table 4. Summary of INDELS of the potato plastid genome.

Supplemental Table 5. Accessions examined in this study, their cladistic relationships, provenance, elevation above sea level, and latitude and

longitude. All species identifications follow Spooner et al.[12]; when two names are present the former is the prior name following Hawkes[43] and the latter the currently accepted name.

Supplemental Figure 1. Coverage and CG plots of the accessions examined here.

Supplemental Figure 2. The phylogenetic relationship of all clades of section *Petota* as determined by maximum likelihood (ML) analysis; the nodes with black dots are supported by bootstrap support values ≥90% and those from 50% to 89% shown numerically; the outgroup, clades 1+2 (see text) and clade 3 are indicated by black text and bracketed with their clade names, clade 4 south (Bolivia and south) wild species by blue text, clade 4 north (Peru) by brown text, and the cultivated species in red text; the blue color of the four accessions of *S. boliviense* (prior name *S. megistacrolobum*), the brown color of the four accessions of *S. pampasense*, and the brown color of the wild species in the cultivated species clade represents their former placement in the nuclear data[19].

Supplemental Table 1. GenBank deposition numbers, large single copy (LSC), small single copy (SSC), inverted repeat (IR) length, and coverage statistics of the 202 accessions of wild and cultivated potatoes and outgroups examined here. We additionally used data for Solanum lycopersicum previously deposited at (https://www.ncbi.nlm.nih.gov/; DQ347959.1).

Species	NPGS accession	GenBank accession	Total Length of Plastid	LSC Length	SSC Length	IR length	Coverage LSC	Coverage SSC	Coverage IR	Average Plastid Coverage	Min. Plastid Coverage	Max. Plastid Coverage
S. abancayense	458403	MH021396	155492	85930	18376	25593	3291	3198	6099	3201	1514	7006
S. abancayense	458404	MH021397	155492	85930	18376	25593	1342	1250	2414	1287	648	2964
S. achacachense	558032	MH021398	155510	85956	18366	25594	1596	1623	2976	1564	714	3909
S. acroglossum	365313	MH021399	155270	85690	18356	25612	1837	1888	3031	1737	529	4673
S. acroglossum	498204	MH021401	155365	85743	18354	25634	3811	3933	6032	3563	1465	8774
S. acroscopicum	365314	MH021400	155477	85937	18362	25589	992	967	1875	971	458	2353
S. albornozii	498206	MH021402	155604	85937	18443	25612	2236	2192	3991	2151	1050	4879
S. ambosinum	365317	MH021403	155489	85945	18358	25593	1323	1273	2517	1296	633	2987
S. ambosinum	365362	MH021404	155531	85973	18372	25593	1347	1352	2465	1310	629	3065
S. ambosinum	498209	MH021405	155492	85930	18376	25593	1499	1501	2768	1461	612	3436
S. ambosinum	498210	MH021406	155534	85975	18373	25593	1487	1416	2766	1444	655	3354
S. ambosinum	498212	MH021407	155530	85972	18372	25593	1131	1111	2145	1109	545	2633
S. ambosinum	498213	MH021408	155532	85974	18372	25593	1158	1127	2145	1126	531	2588
S. andreanum	320345	MH021409	155536	85973	18375	25594	2861	2857	5221	2778	1273	6322
S. andreanum	561648	MH021410	155374	85778	18334	25631	1710	1700	3178	1669	815	3840
S. andreanum	561658	MH021411	155543	85901	18418	25612	2255	2222	2980	1999	116	6147
S. avilesii	498091	MH021412	155555	85994	18373	25594	1975	1930	3529	1901	902	4289
S. avilesii	498092	MH021413	155517	85951	18380	25593	2005	2016	3499	1922	880	4623
S. avilesii	498093	MH021414	155579	86019	18372	25594	1366	1398	2520	1335	595	3034
S. berthaultii	498105	MH021416	155296	85737	18373	25593	1580	1548	2805	1517	693	3397
S. berthaultii	527886	MH021417	155556	85997	18371	25594	2102	2105	3521	1990	1007	4754
S. berthaultii	545850	MH021418	155556	85997	18371	25594	1079	1112	2035	1063	520	2635
S. blanco-galdosii	498214	MH021419	155553	85949	18380	25612	1468	1478	2792	1445	637	3334
S. brevicaule	310931	MH021422	155522	85961	18375	25593	1893	1823	3508	1839	838	4231
S. brevicaule	473378	MH021423	155469	85917	18364	25594	1394	1403	2625	1368	622	3131
S. brevicaule	498111	MH021424	155470	85918	18364	25594	1852	1829	3250	1775	785	4053
S. brevicaule	498218	MH021425	155474	85922	18364	25594	2294	2239	4343	2247	1040	4963
S. brevicaule	545968	MH021426	155461	85909	18364	25594	735	684	1360	711	335	1709
S. brevicaule	545970	MH021427	155467	85916	18363	25594	1072	1062	1975	1043	558	2419
S. brevicaule	545971	MH021428	155464	85913	18363	25594	1440	1381	2719	1406	629	3339
S. brevicaule	545981	MH021429	155444	85883	18375	25593	767	712	1412	741	351	1833
S. bukasovii	266385	MH021430	155518	85968	18364	25593	742	762	1283	711	339	2116
S. bukasovii	365353	MH021431	155520	85971	18363	25593	1738	1697	3137	1678	819	3883

S. bukasovii	414155	MH021432	155476	85920	18368	25594	3374	3326	6175	3274	1466	7251
S. bukasovii	473492	MH021433	155482	85963	18333	25593	975	949	1787	945	474	2194
S. bukasovii	473493	MH021434	155492	85930	18376	25593	1366	1330	2481	1321	648	3073
S. bukasovii	473494	MH021435	155492	85948	18358	25593	1536	1480	2878	1497	685	3369
S. bukasovii	568933	MH021436	155515	85971	18358	25593	1381	1302	2524	1332	651	3016
S. bukasovii	568954	MH021437	155518	85968	18364	25593	1441	1393	2774	1417	640	3212
S. bulbocastanum	545751	MH021438	155406	85835	18381	25595	765	760	1458	753	329	1891
S. bulbocastanum	604074	MH021439	155410	85850	18382	25589	1636	1643	3001	1592	735	3646
S. cajamarquense	230522	MH021440	155525	85973	18366	25593	921	886	1776	906	414	2238
S. canasense	210035	MH021441	155506	85966	18364	25588	3023	2989	5689	2960	1362	6401
S. canasense	246533	MH021442	155506	85946	18406	25577	1225	1230	2262	1195	577	2748
S. canasense	265864	MH021443	155495	85946	18363	25593	903	894	1657	878	422	2093
S. canasense	265865	MH021444	155468	85917	18363	25594	2021	1968	3696	1958	928	4613
S. canasense	283084	MH021445	155492	85930	18376	25593	1191	1169	2171	1154	558	2629
S. canasense	442696	MH021446	155510	85956	18366	25594	1207	1186	2175	1165	555	2675
S. canasense	473355	MH021447	155529	85977	18366	25593	1491	1486	2655	1436	676	3350
S. canasense	498226	MH021448	155492	85930	18376	25593	2575	2539	4752	2505	1133	5555
S. canasense	498227	MH021449	155486	85931	18367	25594	1223	1173	2313	1195	569	2812
S. canasense	545972	MH021450	155510	85956	18366	25594	961	927	1738	927	467	2286
S. canasense	568969	MH021451	155514	85959	18367	25594	757	679	1441	736	344	1912
S. cardiophyllum	283062	MH021452	155456	85908	18372	25588	1558	1528	2843	1510	692	3507
S. cardiophyllum	283063	MH021453	155456	85908	18372	25588	1799	1787	3349	1756	836	3999
S. cardiophyllum	347759	MH021454	155469	85919	18374	25588	1128	1077	2107	1097	465	2534
S. chacoense	275138	MH021455	155518	85960	18372	25593	782	776	1500	771	356	1981
S. chacoense	320294	MH021456	155534	85974	18376	25592	2022	2017	3921	2001	864	4624
S. chacoense	472816	MH021457	155517	85959	18372	25593	956	935	1856	944	417	2235
S. chacoense	472830	MH021458	155526	85967	18375	25592	1424	1417	2658	1392	685	3356
S. chacoense	500020	MH021459	155515	85957	18372	25593	1234	1201	2323	1206	611	2770
S. chomatophilum	365328	MH021460	155427	85894	18355	25589	1514	1522	2750	1469	681	3376
S. chomatophilum	365339	MH021461	155402	85899	18325	25589	951	904	1775	924	429	2184
S. chomatophilum	473489	MH021536	155369	85789	18338	25621	1337	1244	2474	1293	614	2938
S. etuberosum	498311	MH021462	155302	85758	18358	25593	2479	2458	4613	2420	1216	5513
S. goniocalyx	195186	MH021463	155492	85930	18376	25593	1446	1395	2756	1417	682	3194
S. goniocalyx	195188	MH021464	155492	85930	18376	25593	1231	1099	2229	1177	527	2863
S. goniocalyx	195214	MH021465	155492	85930	18376	25593	718	631	1262	679	277	1581
S. goniocalyx	458393	MH021466	155492	85930	18376	25593	702	682	1264	677	325	1669
S. gourlayi	472911	MH021467	155448	85896	18364	25594	1794	1750	3453	1766	843	4033
S. gourlayi	472991	MH021468	155489	85937	18364	25594	1038	920	1929	1000	450	5159
S. gourlayi	472995	MH021469	155462	85909	18365	25594	4142	4116	7743	4050	1906	8819
S. gourlayi	473019	MH021470	155470	85907	18365	25599	1397	1329	2595	1357	583	3221
S. gourlayi	473077	MH021471	155470	85906	18366	25599	1120	1088	2082	1090	491	2586
S. gourlayi	473106	MH021472	155448	85896	18364	25594	2108	2086	3810	2039	964	4464
S. gourlayi	500022	MH021473	155447	85894	18365	25594	773	780	1338	740	388	2129

S. gourlayi	537026	MH021474	155526	85966	18374	25593	1618	1626	2966	1574	702	3715
S. gourlayi	545865	MH021475	155619	86059	18372	25594	1797	1779	3260	1740	839	4071
S. gourlayi	545975	MH021476	155669	86107	18374	25594	1215	1178	2323	1193	493	2831
S. gourlayi	545978	MH021477	155520	85960	18372	25594	1302	1267	2543	1288	572	3176
S. gourlayi	558067	MH021478	155470	85916	18366	25594	718	675	1397	707	298	1829
S. hondelmannii	473365	MH021480	155541	85983	18372	25593	1816	1828	3421	1783	820	4008
S. hondelmannii	498067	MH021481	155561	85997	18374	25595	1651	1706	3069	1619	750	3749
S. hondelmannii	498071	MH021482	155557	85996	18373	25594	2262	2253	4032	2180	1049	4837
S. hondelmannii	545879	MH021483	155526	85966	18374	25593	824	849	1412	788	426	2052
S. hypacrarthrum	473477	MH021486	155484	85921	18385	25589	1125	1030	2044	1080	468	2603
S. incamayoense	473060	MH021487	155462	85900	18364	25599	1693	1553	2932	1602	693	3615
S. incamayoense	473067	MH021488	155460	85900	18364	25598	2289	2263	4226	2228	1062	5014
S. incamayoense	473069	MH021489	155462	85902	18364	25598	1597	1481	2818	1521	719	3585
S. incamayoense	473070	MH021490	155478	85917	18365	25598	3790	3689	7041	3689	1563	8037
S. incamayoense	500048	MH021491	155467	85904	18367	25598	758	736	1432	741	294	2463
S. jamesii	641944	MH021492	155592	86006	18394	25596	2705	2708	5021	2641	1214	5993
S. jamesii	664024	MH021493	155602	86015	18395	25596	2314	2417	3721	2177	901	5729
S. kurtzianum	320327	MH021484	155477	85898	18393	25593	1229	1198	2254	1192	486	2832
S. kurtzianum	472924	MH021494	155506	85949	18373	25592	1686	1626	3116	1637	841	3835
S. kurtzianum	472936	MH021495	155553	85997	18372	25592	3061	3067	5609	2977	1466	6581
S. kurtzianum	472948	MH021496	155520	85960	18374	25593	3505	3544	6451	3418	1693	7446
S. kurtzianum	472952	MH021497	155475	85916	18373	25593	3558	3449	6234	3400	1684	7497
S. kurtzianum	558185	MH021498	155522	85966	18372	25592	2622	2533	4699	2522	1242	5466
S. kurtzianum	558208	MH021499	155517	85960	18371	25593	3888	3895	7076	3773	1874	8203
S. laxissimum	283088	MH021500	155540	85980	18374	25593	2728	2706	5211	2685	1235	5936
S. laxissimum	498252	MH021501	155451	85892	18373	25593	960	950	1884	953	455	2461
S. laxissimum	607887	MH021502	155542	85982	18374	25593	2366	2305	4418	2307	1112	5158
S. leptophyes	458378	MH021503	155470	85918	18364	25594	1092	1058	1911	1043	517	2452
S. leptophyes	473342	MH021504	155467	85915	18364	25594	1622	1551	2983	1570	673	3589
S. leptophyes	473446	MH021505	155532	85966	18380	25593	2620	2548	4790	2537	1130	5672
S. leptophyes	473451	MH021506	155510	85959	18365	25593	1459	1465	2703	1424	660	3177
S. leptophyes	545985	MH021507	155469	85918	18363	25594	1872	1855	3488	1828	810	4186
S. leptophyes	545987	MH021508	155469	85918	18363	25594	2012	2034	3620	1948	962	4408
S. limbaniense	473468	MH021509	155508	85957	18365	25593	2273	2259	4207	2216	954	5095
S. lycopersicum	LA3023	DQ347959	155461	85874	18363	25612						
S. marinasense	210040	MH021510	155527	85975	18366	25593	975	981	1603	918	469	2729
S. marinasense	310944	MH021511	155517	85972	18359	25593	2263	2181	4280	2213	991	4899
S. marinasense	498255	MH021485	155492	85930	18376	25593	2776	2699	5199	2709	1291	5893
S. medians	210045	MH021512	155530	85978	18366	25593	408	404	787	403	189	1645
S. medians	230507	MH021513	155522	85970	18366	25593	1127	1095	2169	1109	516	2587
S. medians	320260	MH021514	155530	85978	18366	25593	1221	1164	2300	1191	561	2822
S. medians	458402	MH021515	155530	85978	18366	25593	1057	1021	2012	1036	477	2432
S. medians	473496	MH021516	155525	85964	18375	25593	1146	1129	2140	1119	544	2617

S. megistacrolobum	210034	MH021517	155559	85998	18375	25593	1017	984	1893	990	456	2441
S. megistacrolobum	473158	MH021518	155536	85985	18365	25593	1593	1400	3063	1550	689	3736
S. megistacrolobum	500029	MH021519	155519	85968	18365	25593	978	912	1825	949	434	2356
S. megistacrolobum	546000	MH021520	155509	85958	18365	25593	1145	1090	2185	1121	490	2606
S. microdontum	218225	MH021415	155513	85953	18374	25593	818	806	1520	798	382	1898
S. microdontum	545884	MH021590	155466	85914	18364	25594	947	927	1766	924	460	2349
S. multidissectum	210052	MH021522	155523	85965	18372	25593	944	899	1660	901	409	2191
S. multidissectum	210055	MH021523	155448	85896	18366	25593	1173	1152	2248	1154	538	2745
S. multidissectum	275272	MH021524	155516	85965	18365	25593	1532	1501	2905	1502	685	3339
S. multidissectum	473349	MH021528	155506	85966	18364	25588	1109	1165	1893	1062	564	3194
S. multidissectum	473352	MH021529	155449	85897	18366	25593	930	980	1600	893	473	3531
S. multiinterruptum	210044	MH021521	155514	85933	18373	25604	1054	1020	1909	1017	439	2383
S. multiinterruptum	365336	MH021525	155490	85903	18363	25612	1784	1758	3335	1743	797	3883
S. multiinterruptum	365337	MH021526	155568	85960	18384	25612	1492	1437	2760	1449	676	3297
S. multiinterruptum	365338	MH021527	155506	85913	18369	25612	846	811	1668	838	375	2066
S. multiinterruptum	498266	MH021530	155503	85916	18363	25612	1259	1215	2370	1229	548	2882
S. palustre	245763	MH021531	155301	85758	18357	25593	1835	1834	3519	1810	831	4276
S. pampasense	275274	MH021532	155519	85958	18375	25593	818	783	1486	789	379	1898
S. pampasense	275275	MH021533	155524	85963	18375	25593	756	710	1460	742	359	1935
S. pampasense	442697	MH021534	155520	85959	18375	25593	436	433	864	435	165	1244
S. pampasense	458381	MH021535	155519	85959	18374	25593	1371	1333	2650	1352	644	3239
S. phureja	195191	MH021537	155492	85930	18376	25593	2523	2496	4850	2488	1175	5558
S. phureja	195198	MH021538	155492	85930	18376	25593	952	883	1757	920	440	2176
S. phureja	225665	MH021539	155492	85930	18376	25593	1258	1218	2384	1232	588	2745
S. phureja	225693	MH021540	155492	85930	18376	25593	1832	1807	3516	1805	754	4192
S. phureja	225703	MH021541	155493	85931	18376	25593	778	764	1431	755	395	1784
S. phureja	243467	MH021542	155492	85930	18376	25593	590	560	1139	580	255	1536
S. phureja	243468	MH021543	155492	85930	18376	25593	1562	1530	2968	1533	666	3499
S. phureja	243469	MH021544	155492	85930	18376	25593	1747	1678	3152	1682	806	3749
S. phureja	258855	MH021545	155492	85930	18376	25593	2269	2190	4109	2189	1072	4901
S. pinnatisectum	253214	MH021546	155614	86029	18395	25595	2022	1997	3704	1963	897	4394
S. pinnatisectum	537023	MH021547	155578	85994	18394	25595	2079	2033	3818	2017	888	4649
S. polyadenium	161728	MH021548	155257	85670	18405	25591	2514	2548	4599	2447	1091	5558
S. polyadenium	347770	MH021549	155231	85656	18397	25589	1734	1672	3141	1672	671	3811
S. sogarandinum	230510	MH021550	155562	85965	18373	25612	1770	1698	3365	1733	774	3930
S. sogarandinum	365360	MH021551	155543	85929	18390	25612	735	723	1432	727	331	1814
S. sparsipilum	246536	MH021552	155469	85917	18364	25594	1968	1934	3710	1927	918	4314
S. sparsipilum	473375	MH021553	155470	85918	18364	25594	2171	2159	3997	2113	983	4700
S. sparsipilum	473385	MH021554	155484	85932	18364	25594	1772	1806	3244	1726	802	3913
S. sparsipilum	498134	MH021555	155463	85911	18364	25594	1323	1347	2444	1292	559	3127
S. sparsipilum	498284	MH021556	155470	85918	18364	25594	1651	1671	3032	1609	789	3664
S. sparsipilum	498285	MH021557	155587	86027	18372	25594	2072	2038	3962	2038	996	4572
S. spegazzinii	320299	MH021558	155519	85981	18352	25593	1603	1576	2985	1563	747	3616

S. spegazzinii	458335	MH021559	155558	85999	18373	25593	1196	1118	2235	1161	550	2820
S. spegazzinii	458337	MH021560	155560	85999	18375	25593	1631	1635	3050	1597	743	3641
S. spegazzinii	472966	MH021561	155520	85960	18374	25593	2339	2291	4272	2267	1123	5025
S. spegazzinii	472988	MH021562	155519	85981	18352	25593	1348	1324	2555	1322	642	3060
S. spegazzinii	472990	MH021563	155560	85999	18375	25593	964	950	1747	933	439	2285
S. stenophyllidium	255527	MH021569	155583	86009	18382	25596	2084	2115	3924	2047	939	4575
S. stenophyllidium	320265	MH021420	155578	86006	18380	25596	2205	2185	4262	2178	1020	5016
S. stenophyllidium	558460	MH021421	155575	86003	18380	25596	2620	2596	4758	2538	1238	5577
S. stenotomum	195204	MH021564	155492	85930	18376	25593	1086	989	1959	1040	440	2453
S. stenotomum	205527	MH021565	155492	85930	18376	25593	2327	2239	4398	2274	1019	5088
S. stenotomum	230512	MH021566	155517	85967	18364	25593	1485	1420	2814	1452	637	3289
S. stenotomum	230513	MH021567	155493	85931	18376	25593	1347	1245	2445	1294	589	2992
S. stenotomum	234011	MH021568	155518	85968	18364	25593	701	662	1305	680	326	1680
S. stenotomum	283141	MH021570	155492	85930	18376	25593	759	726	1417	738	327	1727
S. stenotomum	365344	MH021571	155492	85930	18376	25593	1224	1128	2202	1172	619	2634
S. tarijense	217457	MH021572	155546	85980	18380	25593	3432	3700	5078	3170	628	8154
S. tarijense	414152	MH021573	155299	85741	18372	25593	2289	2375	3713	2157	910	5501
S. tarijense	458366	MH021574	155495	85938	18373	25592	1086	1047	1982	1050	513	2520
S. tarijense	473217	MH021575	155297	85738	18373	25593	1096	1075	1979	1058	532	2381
S. tarijense	473218	MH021576	155295	85736	18373	25593	1427	1438	2564	1381	682	3189
S. tuberosum	320364	MH021479	155492	85930	18376	25593	634	605	1214	622	276	1536
S. tuberosum	DM1-3-516-	JF772170	155492	85930	18376	25593	[redacted]					
S. vernei	320332	MH021580	155590	86033	18373	25592	805	758	1501	781	383	1958
S. vernei	320333	MH021581	155504	85945	18373	25593	3288	3206	6170	3211	1423	7008
S. vernei	458370	MH021582	155506	85946	18374	25593	854	820	1559	826	385	2062
S. vernei	473303	MH021583	155336	85777	18373	25593	995	953	1831	964	442	2328
S. vernei	473309	MH021584	155529	85949	18394	25593	1027	1031	1855	995	505	2494
S. vernei	500070	MH021587	155505	85946	18373	25593	2573	2522	4811	2512	1188	5701
S. vernei	558150	MH021591	155335	85776	18373	25593	891	822	1600	853	407	2145
S. verrucosum	195170	MH021577	155479	85946	18347	25593	795	768	1524	781	367	1919
S. verrucosum	275256	MH021578	155545	85975	18384	25593	1595	1521	3000	1555	736	3643
S. verrucosum	275260	MH021579	155523	85962	18375	25593	1594	1562	3062	1570	663	3626
S. verrucosum	498010	MH021585	155508	85940	18382	25593	1198	1174	2229	1168	580	2731
S. verrucosum	498061	MH021586	155549	85989	18374	25593	1264	1237	2580	1270	568	3607
S. verrucosum	545745	MH021588	155552	85995	18371	25593	1756	1744	3313	1722	803	3815
S. verrucosum	545747	MH021589	155536	85975	18375	25593	1564	1544	3070	1552	726	3649
S. verrucosum	558463	MH021592	155489	85913	18374	25601	2789	2814	5248	2738	1314	6128
S. verrucosum	558488	MH021593	155538	85977	18375	25593	1764	1730	3161	1700	789	4018
S. violaceimarmoratum	473396	MH021594	155505	85941	18378	25593	1426	1422	2670	1396	674	3303
S. violaceimarmoratum	473398	MH021595	155501	85942	18373	25593	3077	3069	5645	2992	1427	6680
S. violaceimarmoratum	498296	MH021596	155577	86018	18373	25593	3608	3627	6811	3544	1611	7936

Supplemental Table 2. Summary of SNPs of potato genome

Number	Position	Reference ge	New SNPs in our study
1	119	A	C
2	176	A	C
3	234	A	C
4	241	A	C
5	274	A	C
6	309	A	C
7	325	A	C
8	333	A	C
9	340	A	C
10	444	A	C
11	873	A	C
12	1224	A	C
13	1505	A	C
14	1764	A	C
15	1768	A	C
16	1923	A	C
17	2107	A	C
18	2216	A	C
19	2665	A	C
20	2869	A	C
21	3094	A	C
22	3416	A	C
23	3448	A	C
24	4423	A	C
25	4441	A	C
26	4505	A	C
27	4650	A	C
28	4726	A	C
29	4742	A	C
30	4888	A	C
31	4948	A	C
32	4952	A	C
33	4965	A	C
34	5187	A	C
35	5338	A	C
36	6103	A	C
37	6277	A	C
38	6289	A	C
39	6366	A	C
40	6532	A	C
41	7053	A	C
42	7054	A	C
43	7131	A	C
44	7402	A	C
45	7408	A	C
46	7451	A	C
47	7546	A	C

48	7548	A	C
49	7668	A	C
50	7913	A	C
51	8071	A	C
52	8162	A	C
53	8427	A	C
54	8689	A	C
55	8885	A	C
56	8932	A	C
57	8967	A	C
58	8980	A	C
59	8988	A	C
60	9085	A	C
61	9136	A	C
62	9361	A	C
63	9505	A	C
64	9556	A	C
65	9573	A	C
66	9871	A	C
67	9950	A	C
68	9992	A	C
69	10138	A	C
70	10259	A	C
71	10412	A	C
72	10603	A	C
73	11558	A	C
74	12512	A	C
75	12571	A	C
76	12675	A	C
77	12721	A	C
78	12925	A	C
79	13255	A	C
80	13290	A	C
81	13291	A	C
82	13325	A	C
83	13419	A	C
84	13552	A	C
85	13729	A	C
86	13812	A	C
87	14152	A	C
88	14256	A	C
89	14286	A	C
90	14382	A	C
91	14490	A	C
92	14529	A	C
93	14548	A	C
94	14679	A	C
95	14707	A	C
96	14939	A	C
97	14948	A	C

98	16686	A	C
99	16689	A	C
100	16718	A	C
101	17593	A	C
102	17952	A	C
103	17965	A	C
104	18106	A	C
105	18259	A	C
106	18373	A	C
107	18910	A	C
108	19456	A	C
109	19482	A	C
110	19924	A	C
111	20059	A	C
112	20527	A	C
113	20920	A	C
114	21170	A	C
115	21381	A	C
116	21411	A	C
117	21690	A	C
118	22032	A	C
119	22758	A	C
120	23311	A	C
121	24010	A	C
122	24154	A	C
123	25162	A	C
124	25189	A	C
125	25216	A	C
126	25455	A	C
127	25687	A	C
128	25929	A	C
129	25963	A	C
130	26356	A	C
131	26731	A	C
132	27153	A	C
133	27310	A	C
134	27382	A	C
135	27619	A	C
136	27632	A	C
137	27719	A	C
138	27770	A	C
139	27960	A	C
140	27977	A	C
141	28116	A	C
142	28129	A	C
143	28418	A	C
144	28451	A	C
145	28857	A	C
146	29001	A	C
147	29044	A	C

148	29139	A	C
149	29611	A	C
150	29614	A	C
151	29868	A	C
152	29875	A	C
153	30221	A	C
154	30711	A	C
155	30813	A	C
156	31071	A	C
157	31297	A	C
158	31329	A	C
159	31334	A	C
160	31335	A	C
161	31392	A	C
162	31473	A	C
163	31481	A	C
164	31523	A	C
165	31636	A	C
166	31650	A	C
167	32476	A	C
168	32568	A	C
169	32605	A	C
170	32624	A	C
171	32758	A	C
172	32778	A	C
173	32984	A	C
174	33287	A	C
175	33347	A	C
176	33504	A	C
177	34862	A	C
178	34865	A	C
179	35198	A	C
180	35887	A	C
181	36216	A	C
182	36294	A	C
183	36779	A	C
184	36918	A	C
185	37061	A	C
186	37306	A	C
187	37442	A	C
188	37847	A	C
189	37851	A	C
190	38143	A	C
191	38440	A	C
192	38899	A	C
193	39027	A	C
194	39178	A	C
195	39577	A	C
196	39583	A	C
197	41129	A	C

198	41651	A	C
199	42593	A	C
200	43073	A	C
201	43258	A	C
202	43274	A	C
203	43312	A	C
204	43405	A	C
205	43424	A	C
206	43806	A	C
207	43991	A	C
208	44091	A	C
209	44225	A	C
210	44710	A	C
211	45452	A	C
212	45559	A	C
213	45864	A	C
214	46009	A	C
215	46083	A	C
216	46120	A	C
217	46223	A	C
218	46250	A	C
219	46444	A	C
220	46535	A	C
221	46548	A	C
222	46571	A	C
223	46653	A	C
224	46658	A	C
225	46667	A	C
226	46840	A	C
227	47048	A	C
228	47864	A	C
229	48206	A	C
230	48293	A	C
231	48724	A	C
232	48809	A	C
233	49158	A	C
234	49267	A	C
235	49275	A	C
236	49283	A	C
237	49434	A	C
238	49557	A	C
239	49848	A	C
240	49906	A	C
241	50165	A	C
242	50674	A	C
243	50795	A	C
244	50870	A	C
245	51936	A	C
246	52144	A	C
247	52148	A	C

248	52168	A	C
249	52247	A	C
250	52271	A	C
251	52560	A	C
252	52578	A	C
253	53020	A	C
254	53055	A	C
255	53192	A	C
256	53703	A	C
257	53709	A	C
258	55770	A	C
259	55783	A	C
260	55851	A	C
261	56087	A	C
262	56185	A	C
263	56228	A	C
264	56285	A	C
265	56306	A	C
266	58018	A	C
267	58050	A	C
268	58167	A	C
269	58299	A	C
270	58377	A	C
271	58532	A	C
272	58617	A	C
273	58888	A	C
274	58916	A	C
275	59016	A	C
276	59343	A	C
277	59924	A	C
278	60268	A	C
279	60415	A	C
280	60507	A	C
281	60542	A	C
282	60650	A	C
283	60678	A	C
284	60791	A	C
285	61721	A	C
286	62146	A	C
287	62314	A	C
288	62336	A	C
289	62454	A	C
290	62506	A	C
291	62584	A	C
292	62665	A	C
293	62840	A	C
294	63494	A	C
295	63509	A	C
296	63538	A	C
297	63600	A	C

298	63803	A	C
299	63841	A	C
300	63973	A	C
301	63982	A	C
302	64364	A	C
303	64423	A	C
304	64578	A	C
305	64805	A	C
306	64840	A	C
307	64854	A	C
308	65099	A	C
309	65278	A	C
310	65396	A	C
311	65537	A	C
312	65584	A	C
313	65634	A	C
314	65690	A	C
315	65701	A	C
316	65752	A	C
317	66662	A	C
318	67014	A	C
319	67126	A	C
320	67200	A	C
321	67235	A	C
322	67285	A	C
323	67306	A	C
324	67445	A	C
325	67458	A	C
326	67527	A	C
327	67545	A	C
328	67590	A	C
329	67678	A	C
330	67816	A	C
331	67832	A	C
332	67843	A	C
333	67848	A	C
334	68189	A	C
335	68485	A	C
336	68967	A	C
337	69071	A	C
338	69144	A	C
339	69192	A	C
340	69622	A	C
341	70273	A	C
342	70640	A	C
343	70691	A	C
344	70964	A	C
345	71943	A	C
346	72371	A	C
347	72471	A	C

348	72636	A	C
349	72822	A	C
350	73012	A	C
351	73127	A	C
352	73412	A	C
353	73513	A	C
354	73686	A	C
355	73832	A	C
356	74420	A	C
357	75034	A	C
358	75061	A	C
359	75608	A	C
360	75798	A	C
361	76390	A	C
362	76589	A	C
363	76986	A	C
364	77324	A	C
365	77815	A	C
366	78209	A	C
367	78455	A	C
368	78460	A	C
369	78833	A	C
370	79110	A	C
371	79597	A	C
372	79840	A	C
373	80058	A	C
374	80109	A	C
375	80397	A	C
376	81866	A	C
377	82022	A	C
378	82143	A	C
379	82738	A	C
380	82745	A	C
381	82811	A	C
382	83077	A	C
383	83294	A	C
384	83449	A	C
385	83491	A	C
386	83523	A	C
387	83541	A	C
388	84108	A	C
389	85040	A	C
390	85284	A	C
391	86060	A	C
392	86163	A	C
393	106177	A	C
394	111208	A	C
395	111366	A	C
396	111677	A	C
397	111698	A	C

398	111910	A	C
399	112505	A	C
400	112675	A	C
401	112879	A	C
402	112880	A	C
403	113626	A	C
404	113701	A	C
405	113814	A	C
406	113825	A	C
407	113918	A	C
408	113921	A	C
409	113930	A	C
410	113968	A	C
411	114016	A	C
412	114051	A	C
413	114139	A	C
414	114257	A	C
415	114562	A	C
416	114571	A	C
417	114594	A	C
418	114796	A	C
419	114804	A	C
420	114810	A	C
421	114884	A	C
422	114955	A	C
423	114990	A	C
424	115103	A	C
425	115238	A	C
426	115258	A	C
427	115270	A	C
428	115365	A	C
429	115670	A	C
430	115681	A	C
431	115777	A	C
432	116207	A	C
433	116314	A	C
434	116459	A	C
435	116641	A	C
436	116722	A	C
437	116808	A	C
438	116937	A	C
439	117675	A	C
440	117723	A	C
441	118833	A	C
442	119271	A	C
443	119406	A	C
444	120028	A	C
445	120175	A	C
446	120211	A	C
447	120277	A	C

448	120713	A	C
449	120851	A	C
450	121617	A	C
451	121669	A	C
452	121698	A	C
453	121785	A	C
454	121927	A	C
455	121959	A	C
456	122298	A	C
457	122494	A	C
458	122562	A	C
459	122606	A	C
460	122622	A	C
461	122984	A	C
462	122996	A	C
463	123828	A	C
464	124804	A	C
465	125096	A	C
466	125114	A	C
467	125210	A	C
468	125990	A	C
469	126153	A	C
470	126175	A	C
471	126428	A	C
472	126443	A	C
473	126509	A	C
474	126564	A	C
475	126590	A	C
476	126643	A	C
477	126676	A	C
478	126775	A	C
479	127186	A	C
480	127419	A	C
481	127494	A	C
482	127520	A	C
483	127556	A	C
484	127655	A	C
485	127736	A	C
486	127893	A	C
487	127994	A	C
488	128009	A	C
489	128074	A	C
490	128975	A	C
491	129022	A	C
492	129052	A	C
493	129275	A	C
494	129355	A	C
495	129375	A	C
496	129747	A	C
497	129977	A	C

498	44	A	G
499	58	A	G
500	202	A	G
501	214	A	G
502	372	A	G
503	452	A	G
504	609	A	G
505	788	A	G
506	807	A	G
507	909	A	G
508	1122	A	G
509	1275	A	G
510	1685	A	G
511	1756	A	G
512	2511	A	G
513	2841	A	G
514	2902	A	G
515	3087	A	G
516	3285	A	G
517	3431	A	G
518	3829	A	G
519	4436	A	G
520	4488	A	G
521	4634	A	G
522	4673	A	G
523	4760	A	G
524	4890	A	G
525	4902	A	G
526	4981	A	G
527	5012	A	G
528	5040	A	G
529	5512	A	G
530	5576	A	G
531	5633	A	G
532	5663	A	G
533	5885	A	G
534	6019	A	G
535	6046	A	G
536	6108	A	G
537	6344	A	G
538	6443	A	G
539	6800	A	G
540	6865	A	G
541	6932	A	G
542	6968	A	G
543	6996	A	G
544	7140	A	G
545	7148	A	G
546	7362	A	G
547	7788	A	G

548	7910	A	G
549	8434	A	G
550	8586	A	G
551	8596	A	G
552	8740	A	G
553	8745	A	G
554	8776	A	G
555	8840	A	G
556	9028	A	G
557	9332	A	G
558	9366	A	G
559	9700	A	G
560	10334	A	G
561	10505	A	G
562	10811	A	G
563	11168	A	G
564	11312	A	G
565	11669	A	G
566	11792	A	G
567	12005	A	G
568	12198	A	G
569	12245	A	G
570	12624	A	G
571	13162	A	G
572	13217	A	G
573	13455	A	G
574	13531	A	G
575	14092	A	G
576	14161	A	G
577	14369	A	G
578	14610	A	G
579	14709	A	G
580	14749	A	G
581	14774	A	G
582	14857	A	G
583	14873	A	G
584	15173	A	G
585	15182	A	G
586	16570	A	G
587	16728	A	G
588	16729	A	G
589	16731	A	G
590	17048	A	G
591	17167	A	G
592	17470	A	G
593	17813	A	G
594	17824	A	G
595	18134	A	G
596	18255	A	G
597	18514	A	G

598	19225	A	G
599	19294	A	G
600	19428	A	G
601	19722	A	G
602	19744	A	G
603	19935	A	G
604	20461	A	G
605	20704	A	G
606	21208	A	G
607	21921	A	G
608	22344	A	G
609	22686	A	G
610	22969	A	G
611	22972	A	G
612	23290	A	G
613	25138	A	G
614	25192	A	G
615	25244	A	G
616	25853	A	G
617	26542	A	G
618	27136	A	G
619	27266	A	G
620	27477	A	G
621	27666	A	G
622	27744	A	G
623	27772	A	G
624	28425	A	G
625	28436	A	G
626	28507	A	G
627	28702	A	G
628	28869	A	G
629	28931	A	G
630	28990	A	G
631	28998	A	G
632	29002	A	G
633	29026	A	G
634	29056	A	G
635	29124	A	G
636	29151	A	G
637	29660	A	G
638	30600	A	G
639	31005	A	G
640	31245	A	G
641	31251	A	G
642	31724	A	G
643	31811	A	G
644	31815	A	G
645	32325	A	G
646	32390	A	G
647	32437	A	G

648	32816	A	G
649	32881	A	G
650	33051	A	G
651	33087	A	G
652	33219	A	G
653	33477	A	G
654	33511	A	G
655	33592	A	G
656	33751	A	G
657	33879	A	G
658	34026	A	G
659	34446	A	G
660	34698	A	G
661	34948	A	G
662	35148	A	G
663	35317	A	G
664	35319	A	G
665	35395	A	G
666	35398	A	G
667	35455	A	G
668	35487	A	G
669	36013	A	G
670	36085	A	G
671	36353	A	G
672	36373	A	G
673	36584	A	G
674	36673	A	G
675	36765	A	G
676	37276	A	G
677	37288	A	G
678	37646	A	G
679	37785	A	G
680	38413	A	G
681	38827	A	G
682	39067	A	G
683	39586	A	G
684	39592	A	G
685	39919	A	G
686	40111	A	G
687	40814	A	G
688	40853	A	G
689	41252	A	G
690	41324	A	G
691	41732	A	G
692	42002	A	G
693	42533	A	G
694	42810	A	G
695	43161	A	G
696	44203	A	G
697	44208	A	G

698	45210	A	G
699	45510	A	G
700	45660	A	G
701	45755	A	G
702	45930	A	G
703	46116	A	G
704	46200	A	G
705	46224	A	G
706	46449	A	G
707	46470	A	G
708	46599	A	G
709	47356	A	G
710	47502	A	G
711	47519	A	G
712	47618	A	G
713	47625	A	G
714	47927	A	G
715	48066	A	G
716	48141	A	G
717	48203	A	G
718	48216	A	G
719	48222	A	G
720	48819	A	G
721	48887	A	G
722	49230	A	G
723	49721	A	G
724	49815	A	G
725	49901	A	G
726	49988	A	G
727	50123	A	G
728	50281	A	G
729	50709	A	G
730	51050	A	G
731	51517	A	G
732	51559	A	G
733	51873	A	G
734	52076	A	G
735	52286	A	G
736	52404	A	G
737	52479	A	G
738	52517	A	G
739	52660	A	G
740	52738	A	G
741	52838	A	G
742	52936	A	G
743	53537	A	G
744	54147	A	G
745	54231	A	G
746	54980	A	G
747	55792	A	G

748	56566	A	G
749	56689	A	G
750	57314	A	G
751	57590	A	G
752	57944	A	G
753	58058	A	G
754	58397	A	G
755	58550	A	G
756	58575	A	G
757	58591	A	G
758	58681	A	G
759	58882	A	G
760	59095	A	G
761	59200	A	G
762	59254	A	G
763	59367	A	G
764	59368	A	G
765	60369	A	G
766	60469	A	G
767	60592	A	G
768	60912	A	G
769	61024	A	G
770	61190	A	G
771	61930	A	G
772	62100	A	G
773	62451	A	G
774	62462	A	G
775	62474	A	G
776	62489	A	G
777	62502	A	G
778	62526	A	G
779	62544	A	G
780	62650	A	G
781	62796	A	G
782	63040	A	G
783	63532	A	G
784	63623	A	G
785	63654	A	G
786	63872	A	G
787	63961	A	G
788	64003	A	G
789	64274	A	G
790	64356	A	G
791	64370	A	G
792	64412	A	G
793	64426	A	G
794	64459	A	G
795	64465	A	G
796	64484	A	G
797	64515	A	G

798	65244	A	G
799	65299	A	G
800	65332	A	G
801	65415	A	G
802	65564	A	G
803	65614	A	G
804	65772	A	G
805	66095	A	G
806	66128	A	G
807	66194	A	G
808	66743	A	G
809	66840	A	G
810	66920	A	G
811	67064	A	G
812	67167	A	G
813	67413	A	G
814	67747	A	G
815	67846	A	G
816	67854	A	G
817	68058	A	G
818	68097	A	G
819	68182	A	G
820	68443	A	G
821	68543	A	G
822	68664	A	G
823	68711	A	G
824	69089	A	G
825	69171	A	G
826	69420	A	G
827	69428	A	G
828	70214	A	G
829	70547	A	G
830	70810	A	G
831	71423	A	G
832	71609	A	G
833	71754	A	G
834	71982	A	G
835	72338	A	G
836	72467	A	G
837	72877	A	G
838	72880	A	G
839	73323	A	G
840	73350	A	G
841	74863	A	G
842	75047	A	G
843	75145	A	G
844	75307	A	G
845	76010	A	G
846	76016	A	G
847	76384	A	G

848	76566	A	G
849	77026	A	G
850	77217	A	G
851	77257	A	G
852	77698	A	G
853	78100	A	G
854	78201	A	G
855	78503	A	G
856	78570	A	G
857	78708	A	G
858	78807	A	G
859	79010	A	G
860	79338	A	G
861	79618	A	G
862	79956	A	G
863	79969	A	G
864	80088	A	G
865	80550	A	G
866	80619	A	G
867	81020	A	G
868	81077	A	G
869	81577	A	G
870	81710	A	G
871	81761	A	G
872	81996	A	G
873	82321	A	G
874	82682	A	G
875	82789	A	G
876	83008	A	G
877	84346	A	G
878	84734	A	G
879	85155	A	G
880	85215	A	G
881	85383	A	G
882	85660	A	G
883	85669	A	G
884	85946	A	G
885	86225	A	G
886	93203	A	G
887	105726	A	G
888	111300	A	G
889	111744	A	G
890	111815	A	G
891	111904	A	G
892	111957	A	G
893	112340	A	G
894	112463	A	G
895	112881	A	G
896	113042	A	G
897	113072	A	G

898	113348	A	G
899	113757	A	G
900	113788	A	G
901	113806	A	G
902	113829	A	G
903	113880	A	G
904	113915	A	G
905	114036	A	G
906	114073	A	G
907	114108	A	G
908	114119	A	G
909	114129	A	G
910	114135	A	G
911	114228	A	G
912	114231	A	G
913	114495	A	G
914	114996	A	G
915	114999	A	G
916	115112	A	G
917	115139	A	G
918	115356	A	G
919	115375	A	G
920	115429	A	G
921	115694	A	G
922	115750	A	G
923	115754	A	G
924	115801	A	G
925	115837	A	G
926	115921	A	G
927	116021	A	G
928	116032	A	G
929	116095	A	G
930	116359	A	G
931	116461	A	G
932	116591	A	G
933	116719	A	G
934	116734	A	G
935	116967	A	G
936	117282	A	G
937	117483	A	G
938	117768	A	G
939	117852	A	G
940	118179	A	G
941	118309	A	G
942	118539	A	G
943	118557	A	G
944	118848	A	G
945	118859	A	G
946	119141	A	G
947	119171	A	G

948	119377	A	G
949	119698	A	G
950	120021	A	G
951	120026	A	G
952	120119	A	G
953	120224	A	G
954	120320	A	G
955	120833	A	G
956	121757	A	G
957	121907	A	G
958	122051	A	G
959	122352	A	G
960	122411	A	G
961	122427	A	G
962	122903	A	G
963	123038	A	G
964	123166	A	G
965	123324	A	G
966	123669	A	G
967	123678	A	G
968	123777	A	G
969	124196	A	G
970	124387	A	G
971	124707	A	G
972	124729	A	G
973	124941	A	G
974	125146	A	G
975	125222	A	G
976	125528	A	G
977	125567	A	G
978	125690	A	G
979	125980	A	G
980	126053	A	G
981	126065	A	G
982	126146	A	G
983	126227	A	G
984	126284	A	G
985	126365	A	G
986	126591	A	G
987	126593	A	G
988	126613	A	G
989	126632	A	G
990	126722	A	G
991	126797	A	G
992	126923	A	G
993	127025	A	G
994	127030	A	G
995	127109	A	G
996	127252	A	G
997	127694	A	G

998	127727	A	G
999	128260	A	G
1000	128327	A	G
1001	128603	A	G
1002	128612	A	G
1003	128819	A	G
1004	128864	A	G
1005	128979	A	G
1006	129324	A	G
1007	129357	A	G
1008	129703	A	G
1009	129827	A	G
1010	129956	A	G
1011	141060	A	G
1012	443	A	T
1013	446	A	T
1014	448	A	T
1015	994	A	T
1016	995	A	T
1017	1887	A	T
1018	3205	A	T
1019	3789	A	T
1020	4229	A	T
1021	4440	A	T
1022	4667	A	T
1023	4759	A	T
1024	4798	A	T
1025	5659	A	T
1026	5706	A	T
1027	5984	A	T
1028	6333	A	T
1029	6356	A	T
1030	6398	A	T
1031	6429	A	T
1032	6708	A	T
1033	6903	A	T
1034	7146	A	T
1035	7160	A	T
1036	8197	A	T
1037	8198	A	T
1038	8199	A	T
1039	8200	A	T
1040	8201	A	T
1041	8934	A	T
1042	9093	A	T
1043	9225	A	T
1044	9513	A	T
1045	9952	A	T
1046	9987	A	T
1047	10245	A	T

1048	10247	A	T
1049	10251	A	T
1050	12614	A	T
1051	13553	A	T
1052	13556	A	T
1053	14531	A	T
1054	15769	A	T
1055	16591	A	T
1056	17966	A	T
1057	18155	A	T
1058	18345	A	T
1059	22812	A	T
1060	23310	A	T
1061	28110	A	T
1062	28123	A	T
1063	28923	A	T
1064	29459	A	T
1065	29540	A	T
1066	29604	A	T
1067	29914	A	T
1068	29982	A	T
1069	31943	A	T
1070	32244	A	T
1071	32260	A	T
1072	32261	A	T
1073	32279	A	T
1074	32886	A	T
1075	32993	A	T
1076	33308	A	T
1077	34416	A	T
1078	34828	A	T
1079	34870	A	T
1080	35370	A	T
1081	35898	A	T
1082	36374	A	T
1083	37104	A	T
1084	37575	A	T
1085	39580	A	T
1086	39594	A	T
1087	45717	A	T
1088	45868	A	T
1089	45936	A	T
1090	46081	A	T
1091	46262	A	T
1092	46572	A	T
1093	46587	A	T
1094	46649	A	T
1095	46663	A	T
1096	47147	A	T
1097	47413	A	T

1098	47507	A	T
1099	47680	A	T
1100	47935	A	T
1101	48059	A	T
1102	49053	A	T
1103	51668	A	T
1104	51669	A	T
1105	51954	A	T
1106	52670	A	T
1107	55806	A	T
1108	56053	A	T
1109	56173	A	T
1110	56418	A	T
1111	57268	A	T
1112	57646	A	T
1113	58437	A	T
1114	60687	A	T
1115	60693	A	T
1116	60873	A	T
1117	62141	A	T
1118	62226	A	T
1119	62316	A	T
1120	62351	A	T
1121	62436	A	T
1122	62508	A	T
1123	62536	A	T
1124	62561	A	T
1125	62585	A	T
1126	64393	A	T
1127	64736	A	T
1128	64749	A	T
1129	64800	A	T
1130	64899	A	T
1131	65116	A	T
1132	65329	A	T
1133	65887	A	T
1134	65994	A	T
1135	66146	A	T
1136	66147	A	T
1137	66197	A	T
1138	66198	A	T
1139	66967	A	T
1140	67220	A	T
1141	67425	A	T
1142	68668	A	T
1143	71371	A	T
1144	71894	A	T
1145	72302	A	T
1146	73791	A	T
1147	74155	A	T

1148	74446	A	T
1149	75158	A	T
1150	77150	A	T
1151	77318	A	T
1152	78097	A	T
1153	78500	A	T
1154	78501	A	T
1155	78966	A	T
1156	79639	A	T
1157	83364	A	T
1158	83467	A	T
1159	83577	A	T
1160	83773	A	T
1161	84715	A	T
1162	84940	A	T
1163	85819	A	T
1164	86152	A	T
1165	105693	A	T
1166	105740	A	T
1167	111539	A	T
1168	111845	A	T
1169	112034	A	T
1170	113668	A	T
1171	113948	A	T
1172	113960	A	T
1173	113981	A	T
1174	114008	A	T
1175	114123	A	T
1176	114538	A	T
1177	114570	A	T
1178	114603	A	T
1179	114798	A	T
1180	114799	A	T
1181	114802	A	T
1182	114803	A	T
1183	115289	A	T
1184	115893	A	T
1185	116721	A	T
1186	116724	A	T
1187	118339	A	T
1188	118868	A	T
1189	118903	A	T
1190	121507	A	T
1191	121845	A	T
1192	121860	A	T
1193	122565	A	T
1194	125070	A	T
1195	125305	A	T
1196	125423	A	T
1197	126216	A	T

1198	127268	A	T
1199	127290	A	T
1200	127476	A	T
1201	127477	A	T
1202	128860	A	T
1203	129104	A	T
1204	129290	A	T
1205	129380	A	T
1206	129391	A	T
1207	146	C	A
1208	221	C	A
1209	346	C	A
1210	520	C	A
1211	999	C	A
1212	1671	C	A
1213	1793	C	A
1214	2125	C	A
1215	2565	C	A
1216	2938	C	A
1217	3233	C	A
1218	3434	C	A
1219	3441	C	A
1220	3787	C	A
1221	3957	C	A
1222	3964	C	A
1223	4194	C	A
1224	4236	C	A
1225	4420	C	A
1226	4486	C	A
1227	4503	C	A
1228	4690	C	A
1229	4707	C	A
1230	4836	C	A
1231	5571	C	A
1232	5834	C	A
1233	5867	C	A
1234	5934	C	A
1235	6069	C	A
1236	6098	C	A
1237	6309	C	A
1238	6316	C	A
1239	6560	C	A
1240	6574	C	A
1241	6670	C	A
1242	6701	C	A
1243	6902	C	A
1244	6930	C	A
1245	6951	C	A
1246	7034	C	A
1247	7042	C	A

1248	7130	C	A
1249	7170	C	A
1250	7429	C	A
1251	7607	C	A
1252	7749	C	A
1253	7920	C	A
1254	8010	C	A
1255	8029	C	A
1256	8364	C	A
1257	8850	C	A
1258	8879	C	A
1259	8922	C	A
1260	8948	C	A
1261	9073	C	A
1262	9963	C	A
1263	9999	C	A
1264	10353	C	A
1265	10681	C	A
1266	11146	C	A
1267	11639	C	A
1268	12570	C	A
1269	12894	C	A
1270	13212	C	A
1271	13442	C	A
1272	13451	C	A
1273	13465	C	A
1274	13496	C	A
1275	13534	C	A
1276	13571	C	A
1277	13907	C	A
1278	14083	C	A
1279	14242	C	A
1280	14421	C	A
1281	14523	C	A
1282	15754	C	A
1283	15889	C	A
1284	15920	C	A
1285	16710	C	A
1286	17928	C	A
1287	18103	C	A
1288	18830	C	A
1289	20719	C	A
1290	20884	C	A
1291	21165	C	A
1292	22434	C	A
1293	23060	C	A
1294	23493	C	A
1295	24445	C	A
1296	25177	C	A
1297	26434	C	A

1298	27262	C	A
1299	27337	C	A
1300	27678	C	A
1301	27720	C	A
1302	27750	C	A
1303	27976	C	A
1304	27983	C	A
1305	28122	C	A
1306	28218	C	A
1307	28287	C	A
1308	28386	C	A
1309	28416	C	A
1310	28650	C	A
1311	28813	C	A
1312	28820	C	A
1313	28855	C	A
1314	28889	C	A
1315	28947	C	A
1316	28968	C	A
1317	28992	C	A
1318	29019	C	A
1319	29136	C	A
1320	29381	C	A
1321	29913	C	A
1322	30084	C	A
1323	30100	C	A
1324	30335	C	A
1325	30425	C	A
1326	30456	C	A
1327	30710	C	A
1328	30945	C	A
1329	31203	C	A
1330	31246	C	A
1331	31319	C	A
1332	31549	C	A
1333	31611	C	A
1334	31800	C	A
1335	31942	C	A
1336	32095	C	A
1337	32343	C	A
1338	32386	C	A
1339	32563	C	A
1340	32599	C	A
1341	32900	C	A
1342	33008	C	A
1343	33181	C	A
1344	33260	C	A
1345	33294	C	A
1346	33351	C	A
1347	33570	C	A

1348	34077	C	A
1349	34677	C	A
1350	34953	C	A
1351	34966	C	A
1352	35344	C	A
1353	35782	C	A
1354	36046	C	A
1355	36276	C	A
1356	36359	C	A
1357	36381	C	A
1358	37046	C	A
1359	37051	C	A
1360	37085	C	A
1361	37437	C	A
1362	37576	C	A
1363	37658	C	A
1364	39619	C	A
1365	42802	C	A
1366	43051	C	A
1367	43250	C	A
1368	43346	C	A
1369	43410	C	A
1370	43751	C	A
1371	44113	C	A
1372	44805	C	A
1373	45289	C	A
1374	45315	C	A
1375	45872	C	A
1376	45917	C	A
1377	46042	C	A
1378	46210	C	A
1379	46904	C	A
1380	47229	C	A
1381	47783	C	A
1382	48005	C	A
1383	48061	C	A
1384	48087	C	A
1385	48163	C	A
1386	48199	C	A
1387	48329	C	A
1388	48599	C	A
1389	48618	C	A
1390	49148	C	A
1391	49561	C	A
1392	49595	C	A
1393	49791	C	A
1394	49865	C	A
1395	49915	C	A
1396	49980	C	A
1397	50144	C	A

1398	52084	C	A
1399	52197	C	A
1400	52236	C	A
1401	52295	C	A
1402	52536	C	A
1403	52659	C	A
1404	52688	C	A
1405	53019	C	A
1406	53119	C	A
1407	53133	C	A
1408	53770	C	A
1409	55264	C	A
1410	55713	C	A
1411	55750	C	A
1412	55858	C	A
1413	56006	C	A
1414	56035	C	A
1415	56052	C	A
1416	56282	C	A
1417	56305	C	A
1418	56455	C	A
1419	57267	C	A
1420	58059	C	A
1421	58174	C	A
1422	58278	C	A
1423	59078	C	A
1424	59215	C	A
1425	60505	C	A
1426	60842	C	A
1427	60845	C	A
1428	60883	C	A
1429	61262	C	A
1430	61376	C	A
1431	61479	C	A
1432	61486	C	A
1433	61580	C	A
1434	62236	C	A
1435	62323	C	A
1436	62439	C	A
1437	62453	C	A
1438	62464	C	A
1439	62490	C	A
1440	62575	C	A
1441	62663	C	A
1442	62712	C	A
1443	62732	C	A
1444	63492	C	A
1445	63534	C	A
1446	63622	C	A
1447	63632	C	A

1448	63742	C	A
1449	63768	C	A
1450	64159	C	A
1451	64172	C	A
1452	64194	C	A
1453	64256	C	A
1454	64405	C	A
1455	64601	C	A
1456	64618	C	A
1457	64720	C	A
1458	64761	C	A
1459	64839	C	A
1460	65044	C	A
1461	65204	C	A
1462	65621	C	A
1463	65627	C	A
1464	65805	C	A
1465	66091	C	A
1466	66140	C	A
1467	66148	C	A
1468	66666	C	A
1469	66793	C	A
1470	66822	C	A
1471	66931	C	A
1472	67044	C	A
1473	67045	C	A
1474	67110	C	A
1475	67116	C	A
1476	67175	C	A
1477	67212	C	A
1478	67241	C	A
1479	67358	C	A
1480	67418	C	A
1481	67435	C	A
1482	67489	C	A
1483	67495	C	A
1484	67552	C	A
1485	67631	C	A
1486	67691	C	A
1487	67746	C	A
1488	67753	C	A
1489	67758	C	A
1490	67809	C	A
1491	67830	C	A
1492	67921	C	A
1493	68009	C	A
1494	68025	C	A
1495	68199	C	A
1496	68265	C	A
1497	69011	C	A

1498	69341	C	A
1499	69392	C	A
1500	70280	C	A
1501	70293	C	A
1502	70831	C	A
1503	71684	C	A
1504	72102	C	A
1505	72165	C	A
1506	72485	C	A
1507	73077	C	A
1508	73244	C	A
1509	73294	C	A
1510	73440	C	A
1511	73581	C	A
1512	73859	C	A
1513	73893	C	A
1514	73916	C	A
1515	74123	C	A
1516	75800	C	A
1517	76633	C	A
1518	76859	C	A
1519	76890	C	A
1520	76981	C	A
1521	77186	C	A
1522	77214	C	A
1523	77322	C	A
1524	77421	C	A
1525	78096	C	A
1526	78891	C	A
1527	79561	C	A
1528	79572	C	A
1529	79609	C	A
1530	79671	C	A
1531	80789	C	A
1532	81409	C	A
1533	81707	C	A
1534	81779	C	A
1535	81932	C	A
1536	82734	C	A
1537	83303	C	A
1538	83327	C	A
1539	83710	C	A
1540	83772	C	A
1541	84363	C	A
1542	84460	C	A
1543	85081	C	A
1544	85108	C	A
1545	86260	C	A
1546	93165	C	A
1547	105700	C	A

1548	109923	C	A
1549	111077	C	A
1550	111183	C	A
1551	111328	C	A
1552	112834	C	A
1553	113173	C	A
1554	113837	C	A
1555	113927	C	A
1556	113979	C	A
1557	113987	C	A
1558	113994	C	A
1559	114018	C	A
1560	114047	C	A
1561	114170	C	A
1562	114329	C	A
1563	114542	C	A
1564	114550	C	A
1565	114657	C	A
1566	114694	C	A
1567	114818	C	A
1568	114895	C	A
1569	114918	C	A
1570	114930	C	A
1571	115057	C	A
1572	115268	C	A
1573	115283	C	A
1574	115288	C	A
1575	115311	C	A
1576	115317	C	A
1577	115348	C	A
1578	116725	C	A
1579	118819	C	A
1580	118879	C	A
1581	119237	C	A
1582	119416	C	A
1583	119661	C	A
1584	120184	C	A
1585	120275	C	A
1586	120294	C	A
1587	120301	C	A
1588	120607	C	A
1589	121259	C	A
1590	121531	C	A
1591	121637	C	A
1592	121696	C	A
1593	121804	C	A
1594	121937	C	A
1595	121969	C	A
1596	121972	C	A
1597	122344	C	A

1598	122414	C	A
1599	122416	C	A
1600	122586	C	A
1601	123531	C	A
1602	123743	C	A
1603	124484	C	A
1604	124668	C	A
1605	124832	C	A
1606	124935	C	A
1607	125379	C	A
1608	125555	C	A
1609	125816	C	A
1610	126325	C	A
1611	126332	C	A
1612	126597	C	A
1613	127313	C	A
1614	127646	C	A
1615	127653	C	A
1616	127849	C	A
1617	127904	C	A
1618	128061	C	A
1619	128268	C	A
1620	128517	C	A
1621	128621	C	A
1622	128647	C	A
1623	128918	C	A
1624	128944	C	A
1625	128960	C	A
1626	128978	C	A
1627	128982	C	A
1628	129143	C	A
1629	129186	C	A
1630	129370	C	A
1631	129607	C	A
1632	120	C	G
1633	853	C	G
1634	964	C	G
1635	1007	C	G
1636	1014	C	G
1637	1902	C	G
1638	2816	C	G
1639	6562	C	G
1640	6722	C	G
1641	7491	C	G
1642	10148	C	G
1643	14317	C	G
1644	14375	C	G
1645	16716	C	G
1646	16732	C	G
1647	19550	C	G

1648	20371	C	G
1649	25153	C	G
1650	26222	C	G
1651	28392	C	G
1652	28417	C	G
1653	28642	C	G
1654	29444	C	G
1655	31449	C	G
1656	31823	C	G
1657	32211	C	G
1658	34779	C	G
1659	34824	C	G
1660	35516	C	G
1661	37273	C	G
1662	37572	C	G
1663	38344	C	G
1664	40210	C	G
1665	42955	C	G
1666	44063	C	G
1667	46688	C	G
1668	47390	C	G
1669	47660	C	G
1670	48140	C	G
1671	48239	C	G
1672	48384	C	G
1673	48741	C	G
1674	49063	C	G
1675	49870	C	G
1676	51682	C	G
1677	52029	C	G
1678	55252	C	G
1679	55555	C	G
1680	56461	C	G
1681	57876	C	G
1682	58154	C	G
1683	61188	C	G
1684	62434	C	G
1685	63515	C	G
1686	63797	C	G
1687	64078	C	G
1688	64083	C	G
1689	64366	C	G
1690	64685	C	G
1691	65108	C	G
1692	65892	C	G
1693	66061	C	G
1694	68032	C	G
1695	68050	C	G
1696	68381	C	G
1697	68611	C	G

1698	68614	C	G
1699	71850	C	G
1700	71949	C	G
1701	72995	C	G
1702	73834	C	G
1703	81665	C	G
1704	81720	C	G
1705	83443	C	G
1706	85095	C	G
1707	86072	C	G
1708	86085	C	G
1709	86221	C	G
1710	105673	C	G
1711	109332	C	G
1712	111198	C	G
1713	112101	C	G
1714	113565	C	G
1715	113828	C	G
1716	113854	C	G
1717	114208	C	G
1718	114588	C	G
1719	115968	C	G
1720	119369	C	G
1721	120104	C	G
1722	120121	C	G
1723	120177	C	G
1724	120260	C	G
1725	123830	C	G
1726	124409	C	G
1727	125127	C	G
1728	126195	C	G
1729	128693	C	G
1730	128909	C	G
1731	128929	C	G
1732	128985	C	G
1733	139804	C	G
1734	14	C	T
1735	113	C	T
1736	381	C	T
1737	390	C	T
1738	478	C	T
1739	801	C	T
1740	1050	C	T
1741	1215	C	T
1742	1586	C	T
1743	2156	C	T
1744	2191	C	T
1745	2574	C	T
1746	2643	C	T
1747	2669	C	T

1748	2671	C	T
1749	2832	C	T
1750	2879	C	T
1751	2939	C	T
1752	3174	C	T
1753	3345	C	T
1754	3369	C	T
1755	3483	C	T
1756	3540	C	T
1757	3627	C	T
1758	3683	C	T
1759	3766	C	T
1760	3896	C	T
1761	3987	C	T
1762	3999	C	T
1763	4078	C	T
1764	4135	C	T
1765	4138	C	T
1766	4362	C	T
1767	4443	C	T
1768	4471	C	T
1769	4476	C	T
1770	4541	C	T
1771	4736	C	T
1772	5551	C	T
1773	5992	C	T
1774	6275	C	T
1775	6479	C	T
1776	6600	C	T
1777	6603	C	T
1778	6730	C	T
1779	6795	C	T
1780	6882	C	T
1781	6897	C	T
1782	6918	C	T
1783	6931	C	T
1784	6941	C	T
1785	6943	C	T
1786	7015	C	T
1787	7026	C	T
1788	7045	C	T
1789	7149	C	T
1790	7154	C	T
1791	7297	C	T
1792	7533	C	T
1793	7608	C	T
1794	8004	C	T
1795	8185	C	T
1796	8306	C	T
1797	8452	C	T

1798	8780	C	T
1799	9004	C	T
1800	9057	C	T
1801	9107	C	T
1802	9137	C	T
1803	9138	C	T
1804	9331	C	T
1805	9609	C	T
1806	9680	C	T
1807	9735	C	T
1808	9836	C	T
1809	10163	C	T
1810	10335	C	T
1811	10417	C	T
1812	10520	C	T
1813	10688	C	T
1814	11192	C	T
1815	11255	C	T
1816	11598	C	T
1817	12613	C	T
1818	12809	C	T
1819	12959	C	T
1820	13148	C	T
1821	13191	C	T
1822	13395	C	T
1823	13732	C	T
1824	14263	C	T
1825	14279	C	T
1826	14343	C	T
1827	14350	C	T
1828	14697	C	T
1829	14720	C	T
1830	14729	C	T
1831	14821	C	T
1832	14947	C	T
1833	14999	C	T
1834	16060	C	T
1835	16409	C	T
1836	17109	C	T
1837	17243	C	T
1838	17860	C	T
1839	18142	C	T
1840	18331	C	T
1841	18428	C	T
1842	18519	C	T
1843	18559	C	T
1844	18760	C	T
1845	19211	C	T
1846	19421	C	T
1847	19470	C	T

1848	19489	C	T
1849	19512	C	T
1850	19527	C	T
1851	19585	C	T
1852	19616	C	T
1853	19945	C	T
1854	20274	C	T
1855	20378	C	T
1856	20386	C	T
1857	20650	C	T
1858	20865	C	T
1859	21459	C	T
1860	21586	C	T
1861	21625	C	T
1862	21849	C	T
1863	22524	C	T
1864	22614	C	T
1865	22705	C	T
1866	23059	C	T
1867	23328	C	T
1868	23347	C	T
1869	23781	C	T
1870	24510	C	T
1871	25003	C	T
1872	25552	C	T
1873	25918	C	T
1874	26209	C	T
1875	26812	C	T
1876	27650	C	T
1877	27885	C	T
1878	27931	C	T
1879	28069	C	T
1880	28258	C	T
1881	28388	C	T
1882	28415	C	T
1883	28692	C	T
1884	28766	C	T
1885	28805	C	T
1886	28840	C	T
1887	28876	C	T
1888	28904	C	T
1889	28905	C	T
1890	28917	C	T
1891	28938	C	T
1892	28981	C	T
1893	29054	C	T
1894	29324	C	T
1895	29696	C	T
1896	29778	C	T
1897	29877	C	T

1898	30017	C	T
1899	30264	C	T
1900	30391	C	T
1901	30946	C	T
1902	31164	C	T
1903	31483	C	T
1904	31553	C	T
1905	31998	C	T
1906	32066	C	T
1907	32166	C	T
1908	32566	C	T
1909	32570	C	T
1910	32712	C	T
1911	32720	C	T
1912	33378	C	T
1913	33546	C	T
1914	33671	C	T
1915	34181	C	T
1916	34425	C	T
1917	34448	C	T
1918	34560	C	T
1919	34846	C	T
1920	34868	C	T
1921	34872	C	T
1922	35000	C	T
1923	35059	C	T
1924	35068	C	T
1925	35076	C	T
1926	35273	C	T
1927	35351	C	T
1928	35352	C	T
1929	35359	C	T
1930	35419	C	T
1931	35575	C	T
1932	35953	C	T
1933	36121	C	T
1934	36332	C	T
1935	36548	C	T
1936	36736	C	T
1937	36762	C	T
1938	36915	C	T
1939	37017	C	T
1940	37179	C	T
1941	37643	C	T
1942	37648	C	T
1943	37821	C	T
1944	38139	C	T
1945	38185	C	T
1946	38248	C	T
1947	38506	C	T

1948	38704	C	T
1949	38810	C	T
1950	38826	C	T
1951	38985	C	T
1952	39582	C	T
1953	40201	C	T
1954	40574	C	T
1955	40687	C	T
1956	40751	C	T
1957	40961	C	T
1958	41084	C	T
1959	41159	C	T
1960	41231	C	T
1961	41429	C	T
1962	41858	C	T
1963	41906	C	T
1964	41924	C	T
1965	42731	C	T
1966	42968	C	T
1967	43012	C	T
1968	43215	C	T
1969	43251	C	T
1970	43656	C	T
1971	44082	C	T
1972	44159	C	T
1973	44256	C	T
1974	44282	C	T
1975	45086	C	T
1976	45107	C	T
1977	45404	C	T
1978	45447	C	T
1979	45477	C	T
1980	45611	C	T
1981	45694	C	T
1982	45911	C	T
1983	46035	C	T
1984	46333	C	T
1985	46351	C	T
1986	46402	C	T
1987	46478	C	T
1988	47608	C	T
1989	47776	C	T
1990	47903	C	T
1991	48262	C	T
1992	48291	C	T
1993	48448	C	T
1994	48654	C	T
1995	48753	C	T
1996	48834	C	T
1997	49117	C	T

1998	49270	C	T
1999	49761	C	T
2000	49773	C	T
2001	49831	C	T
2002	50018	C	T
2003	50120	C	T
2004	50157	C	T
2005	51840	C	T
2006	51880	C	T
2007	52037	C	T
2008	52096	C	T
2009	52292	C	T
2010	52498	C	T
2011	53555	C	T
2012	53844	C	T
2013	53982	C	T
2014	54078	C	T
2015	55520	C	T
2016	55563	C	T
2017	55567	C	T
2018	55692	C	T
2019	55785	C	T
2020	55929	C	T
2021	56064	C	T
2022	56164	C	T
2023	56488	C	T
2024	56650	C	T
2025	56879	C	T
2026	56980	C	T
2027	57034	C	T
2028	57315	C	T
2029	57352	C	T
2030	58640	C	T
2031	58655	C	T
2032	58894	C	T
2033	59394	C	T
2034	59703	C	T
2035	59853	C	T
2036	60821	C	T
2037	61099	C	T
2038	61101	C	T
2039	61107	C	T
2040	61680	C	T
2041	62104	C	T
2042	62112	C	T
2043	62125	C	T
2044	62147	C	T
2045	62165	C	T
2046	62172	C	T
2047	62196	C	T

2048	62219	C	T
2049	62233	C	T
2050	62238	C	T
2051	62242	C	T
2052	62245	C	T
2053	62250	C	T
2054	62261	C	T
2055	62309	C	T
2056	62318	C	T
2057	62340	C	T
2058	62344	C	T
2059	62354	C	T
2060	62428	C	T
2061	62435	C	T
2062	62456	C	T
2063	62457	C	T
2064	62468	C	T
2065	62470	C	T
2066	62480	C	T
2067	62538	C	T
2068	62548	C	T
2069	62607	C	T
2070	62818	C	T
2071	62834	C	T
2072	62845	C	T
2073	63092	C	T
2074	63635	C	T
2075	63713	C	T
2076	63838	C	T
2077	63853	C	T
2078	64117	C	T
2079	64348	C	T
2080	64587	C	T
2081	64814	C	T
2082	64845	C	T
2083	64876	C	T
2084	64999	C	T
2085	65169	C	T
2086	65263	C	T
2087	65269	C	T
2088	65309	C	T
2089	65612	C	T
2090	65889	C	T
2091	66021	C	T
2092	66055	C	T
2093	66429	C	T
2094	66619	C	T
2095	66624	C	T
2096	67168	C	T
2097	67182	C	T

2098	67477	C	T
2099	67519	C	T
2100	67565	C	T
2101	67567	C	T
2102	67626	C	T
2103	67705	C	T
2104	67808	C	T
2105	68244	C	T
2106	68306	C	T
2107	68399	C	T
2108	68467	C	T
2109	68512	C	T
2110	68631	C	T
2111	69127	C	T
2112	69272	C	T
2113	69310	C	T
2114	69567	C	T
2115	69810	C	T
2116	70000	C	T
2117	70545	C	T
2118	70713	C	T
2119	71462	C	T
2120	71606	C	T
2121	71895	C	T
2122	71991	C	T
2123	72316	C	T
2124	72320	C	T
2125	72916	C	T
2126	72993	C	T
2127	73018	C	T
2128	73056	C	T
2129	73289	C	T
2130	73968	C	T
2131	74087	C	T
2132	74100	C	T
2133	74575	C	T
2134	74908	C	T
2135	75038	C	T
2136	75056	C	T
2137	75211	C	T
2138	75282	C	T
2139	75400	C	T
2140	75517	C	T
2141	75759	C	T
2142	76077	C	T
2143	76565	C	T
2144	76936	C	T
2145	77067	C	T
2146	77139	C	T
2147	77152	C	T

2148	77336	C	T
2149	77464	C	T
2150	77641	C	T
2151	77710	C	T
2152	77851	C	T
2153	77860	C	T
2154	78333	C	T
2155	78709	C	T
2156	78832	C	T
2157	79150	C	T
2158	79242	C	T
2159	79413	C	T
2160	80007	C	T
2161	80131	C	T
2162	80629	C	T
2163	80774	C	T
2164	80886	C	T
2165	81170	C	T
2166	81343	C	T
2167	81384	C	T
2168	81485	C	T
2169	81519	C	T
2170	81556	C	T
2171	81578	C	T
2172	81613	C	T
2173	81672	C	T
2174	81673	C	T
2175	81677	C	T
2176	81769	C	T
2177	82033	C	T
2178	82236	C	T
2179	82296	C	T
2180	82393	C	T
2181	82486	C	T
2182	82506	C	T
2183	82618	C	T
2184	82642	C	T
2185	82861	C	T
2186	82862	C	T
2187	82865	C	T
2188	83245	C	T
2189	83506	C	T
2190	83645	C	T
2191	83677	C	T
2192	83766	C	T
2193	83793	C	T
2194	83894	C	T
2195	83898	C	T
2196	84288	C	T
2197	84318	C	T

2198	84832	C	T
2199	85048	C	T
2200	85104	C	T
2201	85106	C	T
2202	85272	C	T
2203	85362	C	T
2204	85509	C	T
2205	85618	C	T
2206	85699	C	T
2207	85953	C	T
2208	86050	C	T
2209	86055	C	T
2210	86070	C	T
2211	86079	C	T
2212	86099	C	T
2213	86108	C	T
2214	86110	C	T
2215	86120	C	T
2216	86128	C	T
2217	86137	C	T
2218	86160	C	T
2219	86165	C	T
2220	86168	C	T
2221	86170	C	T
2222	86192	C	T
2223	86198	C	T
2224	86199	C	T
2225	86213	C	T
2226	86231	C	T
2227	86254	C	T
2228	86259	C	T
2229	86287	C	T
2230	105706	C	T
2231	105749	C	T
2232	106084	C	T
2233	106199	C	T
2234	106209	C	T
2235	109675	C	T
2236	111367	C	T
2237	111410	C	T
2238	111457	C	T
2239	111476	C	T
2240	111776	C	T
2241	111785	C	T
2242	111944	C	T
2243	111959	C	T
2244	112102	C	T
2245	112378	C	T
2246	112538	C	T
2247	112663	C	T

2248	112736	C	T
2249	112766	C	T
2250	112868	C	T
2251	113489	C	T
2252	113588	C	T
2253	113678	C	T
2254	113820	C	T
2255	113944	C	T
2256	114011	C	T
2257	114181	C	T
2258	114195	C	T
2259	114601	C	T
2260	114807	C	T
2261	114928	C	T
2262	115250	C	T
2263	115309	C	T
2264	115318	C	T
2265	115349	C	T
2266	115593	C	T
2267	115596	C	T
2268	115627	C	T
2269	115680	C	T
2270	115705	C	T
2271	115878	C	T
2272	115885	C	T
2273	116164	C	T
2274	116457	C	T
2275	116613	C	T
2276	118145	C	T
2277	118251	C	T
2278	118350	C	T
2279	118500	C	T
2280	119453	C	T
2281	119678	C	T
2282	119784	C	T
2283	120096	C	T
2284	120326	C	T
2285	120428	C	T
2286	121249	C	T
2287	121587	C	T
2288	122005	C	T
2289	122015	C	T
2290	122173	C	T
2291	122244	C	T
2292	122306	C	T
2293	122394	C	T
2294	122536	C	T
2295	122544	C	T
2296	122848	C	T
2297	122900	C	T

2298	123014	C	T
2299	123112	C	T
2300	123549	C	T
2301	123621	C	T
2302	123692	C	T
2303	123966	C	T
2304	124017	C	T
2305	124079	C	T
2306	124338	C	T
2307	124501	C	T
2308	124568	C	T
2309	124581	C	T
2310	124620	C	T
2311	124666	C	T
2312	124894	C	T
2313	125038	C	T
2314	125068	C	T
2315	125073	C	T
2316	125084	C	T
2317	125140	C	T
2318	125174	C	T
2319	125195	C	T
2320	125333	C	T
2321	125386	C	T
2322	125404	C	T
2323	125545	C	T
2324	125672	C	T
2325	125693	C	T
2326	125699	C	T
2327	125702	C	T
2328	125735	C	T
2329	125741	C	T
2330	125876	C	T
2331	126447	C	T
2332	126565	C	T
2333	126656	C	T
2334	126826	C	T
2335	126874	C	T
2336	126996	C	T
2337	127022	C	T
2338	127054	C	T
2339	127226	C	T
2340	127550	C	T
2341	127660	C	T
2342	127668	C	T
2343	127918	C	T
2344	128059	C	T
2345	128252	C	T
2346	128423	C	T
2347	128486	C	T

2348	128497	C	T
2349	128717	C	T
2350	128884	C	T
2351	128992	C	T
2352	129016	C	T
2353	129079	C	T
2354	129085	C	T
2355	129142	C	T
2356	129170	C	T
2357	129184	C	T
2358	129235	C	T
2359	129272	C	T
2360	129332	C	T
2361	129340	C	T
2362	129350	C	T
2363	129365	C	T
2364	129404	C	T
2365	129454	C	T
2366	129464	C	T
2367	129491	C	T
2368	129610	C	T
2369	129614	C	T
2370	129929	C	T
2371	196	G	A
2372	225	G	A
2373	275	G	A
2374	300	G	A
2375	369	G	A
2376	415	G	A
2377	651	G	A
2378	741	G	A
2379	806	G	A
2380	861	G	A
2381	1053	G	A
2382	1131	G	A
2383	1533	G	A
2384	1689	G	A
2385	1740	G	A
2386	1802	G	A
2387	1880	G	A
2388	1938	G	A
2389	2001	G	A
2390	2066	G	A
2391	2508	G	A
2392	2633	G	A
2393	2658	G	A
2394	2673	G	A
2395	2710	G	A
2396	2933	G	A
2397	2978	G	A

2398	2991	G	A
2399	3198	G	A
2400	3303	G	A
2401	3313	G	A
2402	3865	G	A
2403	3938	G	A
2404	3965	G	A
2405	3978	G	A
2406	4137	G	A
2407	4689	G	A
2408	4752	G	A
2409	4755	G	A
2410	4816	G	A
2411	4936	G	A
2412	4945	G	A
2413	4978	G	A
2414	5086	G	A
2415	5292	G	A
2416	5320	G	A
2417	5345	G	A
2418	5360	G	A
2419	5567	G	A
2420	5696	G	A
2421	6339	G	A
2422	6533	G	A
2423	6668	G	A
2424	6706	G	A
2425	6713	G	A
2426	6883	G	A
2427	6895	G	A
2428	6899	G	A
2429	6908	G	A
2430	7025	G	A
2431	7138	G	A
2432	7158	G	A
2433	7414	G	A
2434	8022	G	A
2435	8024	G	A
2436	8037	G	A
2437	8053	G	A
2438	8358	G	A
2439	8433	G	A
2440	9060	G	A
2441	9072	G	A
2442	9097	G	A
2443	9158	G	A
2444	9262	G	A
2445	9363	G	A
2446	9466	G	A
2447	9561	G	A

2448	10135	G	A
2449	10454	G	A
2450	10550	G	A
2451	10670	G	A
2452	10841	G	A
2453	10868	G	A
2454	11497	G	A
2455	12031	G	A
2456	12218	G	A
2457	12415	G	A
2458	12625	G	A
2459	12990	G	A
2460	13413	G	A
2461	13436	G	A
2462	13471	G	A
2463	13533	G	A
2464	13810	G	A
2465	13922	G	A
2466	14096	G	A
2467	14150	G	A
2468	14312	G	A
2469	14615	G	A
2470	14752	G	A
2471	14769	G	A
2472	14930	G	A
2473	15221	G	A
2474	15584	G	A
2475	15710	G	A
2476	16087	G	A
2477	19239	G	A
2478	19255	G	A
2479	19283	G	A
2480	19315	G	A
2481	19342	G	A
2482	19408	G	A
2483	19511	G	A
2484	19541	G	A
2485	19696	G	A
2486	20027	G	A
2487	20050	G	A
2488	20401	G	A
2489	20464	G	A
2490	20536	G	A
2491	20794	G	A
2492	21284	G	A
2493	21286	G	A
2494	21396	G	A
2495	21703	G	A
2496	21781	G	A
2497	21882	G	A

2498	22140	G	A
2499	22529	G	A
2500	23027	G	A
2501	23137	G	A
2502	23231	G	A
2503	23360	G	A
2504	23490	G	A
2505	23540	G	A
2506	23885	G	A
2507	23887	G	A
2508	24487	G	A
2509	25263	G	A
2510	25642	G	A
2511	26026	G	A
2512	26035	G	A
2513	26539	G	A
2514	26647	G	A
2515	26771	G	A
2516	26842	G	A
2517	27038	G	A
2518	27304	G	A
2519	27439	G	A
2520	27737	G	A
2521	27783	G	A
2522	27817	G	A
2523	27922	G	A
2524	28316	G	A
2525	28629	G	A
2526	28833	G	A
2527	28933	G	A
2528	28939	G	A
2529	28960	G	A
2530	28971	G	A
2531	29360	G	A
2532	29380	G	A
2533	29635	G	A
2534	29732	G	A
2535	29768	G	A
2536	29798	G	A
2537	29823	G	A
2538	29973	G	A
2539	30204	G	A
2540	31045	G	A
2541	31113	G	A
2542	31162	G	A
2543	31234	G	A
2544	31396	G	A
2545	31541	G	A
2546	31560	G	A
2547	31603	G	A

2548	31660	G	A
2549	31743	G	A
2550	31834	G	A
2551	32194	G	A
2552	32335	G	A
2553	32336	G	A
2554	32609	G	A
2555	32987	G	A
2556	33046	G	A
2557	33413	G	A
2558	33447	G	A
2559	33527	G	A
2560	34258	G	A
2561	34308	G	A
2562	34427	G	A
2563	34523	G	A
2564	34602	G	A
2565	34857	G	A
2566	34886	G	A
2567	34949	G	A
2568	34972	G	A
2569	34988	G	A
2570	34993	G	A
2571	35132	G	A
2572	35149	G	A
2573	35284	G	A
2574	35321	G	A
2575	35393	G	A
2576	35401	G	A
2577	35451	G	A
2578	35484	G	A
2579	36182	G	A
2580	36372	G	A
2581	36578	G	A
2582	36624	G	A
2583	36922	G	A
2584	37258	G	A
2585	37282	G	A
2586	37293	G	A
2587	37475	G	A
2588	37660	G	A
2589	38181	G	A
2590	38209	G	A
2591	38281	G	A
2592	38644	G	A
2593	38755	G	A
2594	38779	G	A
2595	38939	G	A
2596	39346	G	A
2597	39433	G	A

2598	39788	G	A
2599	39925	G	A
2600	40057	G	A
2601	40138	G	A
2602	40587	G	A
2603	41097	G	A
2604	41186	G	A
2605	42302	G	A
2606	42491	G	A
2607	42815	G	A
2608	43042	G	A
2609	43276	G	A
2610	43434	G	A
2611	43568	G	A
2612	43788	G	A
2613	44144	G	A
2614	44903	G	A
2615	45300	G	A
2616	45445	G	A
2617	45537	G	A
2618	45676	G	A
2619	45748	G	A
2620	45919	G	A
2621	46036	G	A
2622	46141	G	A
2623	46194	G	A
2624	46475	G	A
2625	46628	G	A
2626	46771	G	A
2627	47447	G	A
2628	47521	G	A
2629	47538	G	A
2630	47980	G	A
2631	48229	G	A
2632	48359	G	A
2633	48828	G	A
2634	49604	G	A
2635	49611	G	A
2636	49654	G	A
2637	49806	G	A
2638	50043	G	A
2639	50534	G	A
2640	50593	G	A
2641	50904	G	A
2642	51068	G	A
2643	51293	G	A
2644	51828	G	A
2645	51950	G	A
2646	51955	G	A
2647	52400	G	A

2648	52402	G	A
2649	52417	G	A
2650	52862	G	A
2651	52879	G	A
2652	53054	G	A
2653	53238	G	A
2654	53571	G	A
2655	53778	G	A
2656	53789	G	A
2657	53960	G	A
2658	54111	G	A
2659	54367	G	A
2660	54644	G	A
2661	54959	G	A
2662	55235	G	A
2663	55332	G	A
2664	55640	G	A
2665	55780	G	A
2666	55814	G	A
2667	56626	G	A
2668	56698	G	A
2669	56944	G	A
2670	57218	G	A
2671	57293	G	A
2672	57442	G	A
2673	57846	G	A
2674	58393	G	A
2675	58624	G	A
2676	58742	G	A
2677	58797	G	A
2678	59261	G	A
2679	59286	G	A
2680	59328	G	A
2681	59415	G	A
2682	59519	G	A
2683	59673	G	A
2684	59847	G	A
2685	60365	G	A
2686	60599	G	A
2687	61131	G	A
2688	61202	G	A
2689	61281	G	A
2690	62105	G	A
2691	62120	G	A
2692	62132	G	A
2693	62148	G	A
2694	62162	G	A
2695	62191	G	A
2696	62195	G	A
2697	62220	G	A

2698	62244	G	A
2699	62287	G	A
2700	62319	G	A
2701	62412	G	A
2702	62437	G	A
2703	62488	G	A
2704	62496	G	A
2705	62503	G	A
2706	62525	G	A
2707	62543	G	A
2708	62553	G	A
2709	62565	G	A
2710	62588	G	A
2711	62679	G	A
2712	63495	G	A
2713	63546	G	A
2714	63580	G	A
2715	63807	G	A
2716	63850	G	A
2717	63910	G	A
2718	63977	G	A
2719	64042	G	A
2720	64105	G	A
2721	64349	G	A
2722	64432	G	A
2723	64774	G	A
2724	64882	G	A
2725	64905	G	A
2726	65063	G	A
2727	65076	G	A
2728	65087	G	A
2729	65429	G	A
2730	65872	G	A
2731	66392	G	A
2732	66450	G	A
2733	66933	G	A
2734	67020	G	A
2735	67158	G	A
2736	67268	G	A
2737	67518	G	A
2738	68057	G	A
2739	68392	G	A
2740	68998	G	A
2741	69117	G	A
2742	69204	G	A
2743	69597	G	A
2744	69651	G	A
2745	69731	G	A
2746	70007	G	A
2747	70167	G	A

2748	70212	G	A
2749	70511	G	A
2750	71218	G	A
2751	71721	G	A
2752	71820	G	A
2753	72016	G	A
2754	72151	G	A
2755	72192	G	A
2756	72401	G	A
2757	72499	G	A
2758	72991	G	A
2759	73011	G	A
2760	73122	G	A
2761	73171	G	A
2762	73241	G	A
2763	73534	G	A
2764	73876	G	A
2765	74060	G	A
2766	74428	G	A
2767	74440	G	A
2768	74581	G	A
2769	74974	G	A
2770	75083	G	A
2771	75700	G	A
2772	75835	G	A
2773	76106	G	A
2774	76262	G	A
2775	77294	G	A
2776	77302	G	A
2777	77353	G	A
2778	77482	G	A
2779	77627	G	A
2780	77953	G	A
2781	78084	G	A
2782	78141	G	A
2783	78261	G	A
2784	78329	G	A
2785	78623	G	A
2786	79215	G	A
2787	79419	G	A
2788	79926	G	A
2789	80185	G	A
2790	80214	G	A
2791	80473	G	A
2792	80478	G	A
2793	80577	G	A
2794	80731	G	A
2795	80958	G	A
2796	81080	G	A
2797	81278	G	A

2798	81495	G	A
2799	81650	G	A
2800	81700	G	A
2801	81788	G	A
2802	82365	G	A
2803	82366	G	A
2804	82656	G	A
2805	82709	G	A
2806	83599	G	A
2807	83672	G	A
2808	83947	G	A
2809	84311	G	A
2810	84655	G	A
2811	84661	G	A
2812	84817	G	A
2813	85230	G	A
2814	85236	G	A
2815	85886	G	A
2816	86071	G	A
2817	86133	G	A
2818	86139	G	A
2819	86144	G	A
2820	86162	G	A
2821	86172	G	A
2822	86190	G	A
2823	86193	G	A
2824	86208	G	A
2825	86226	G	A
2826	86285	G	A
2827	86299	G	A
2828	93263	G	A
2829	105695	G	A
2830	106181	G	A
2831	106212	G	A
2832	109296	G	A
2833	109317	G	A
2834	109320	G	A
2835	111413	G	A
2836	111467	G	A
2837	111542	G	A
2838	111786	G	A
2839	111876	G	A
2840	111950	G	A
2841	112817	G	A
2842	112855	G	A
2843	113156	G	A
2844	113611	G	A
2845	113755	G	A
2846	113855	G	A
2847	113892	G	A

2848	113945	G	A
2849	114012	G	A
2850	114133	G	A
2851	114276	G	A
2852	114314	G	A
2853	114806	G	A
2854	114913	G	A
2855	115040	G	A
2856	115111	G	A
2857	115211	G	A
2858	115252	G	A
2859	115393	G	A
2860	115456	G	A
2861	115506	G	A
2862	115732	G	A
2863	115764	G	A
2864	115772	G	A
2865	115781	G	A
2866	116880	G	A
2867	116945	G	A
2868	116949	G	A
2869	116983	G	A
2870	117138	G	A
2871	117360	G	A
2872	117619	G	A
2873	117657	G	A
2874	117879	G	A
2875	118144	G	A
2876	118260	G	A
2877	118485	G	A
2878	118515	G	A
2879	118752	G	A
2880	118829	G	A
2881	118837	G	A
2882	119250	G	A
2883	119372	G	A
2884	119487	G	A
2885	119636	G	A
2886	120118	G	A
2887	120437	G	A
2888	120449	G	A
2889	120743	G	A
2890	121652	G	A
2891	122202	G	A
2892	122307	G	A
2893	122552	G	A
2894	122777	G	A
2895	122872	G	A
2896	122942	G	A
2897	123354	G	A

2898	123485	G	A
2899	123750	G	A
2900	124077	G	A
2901	124272	G	A
2902	124372	G	A
2903	124425	G	A
2904	124504	G	A
2905	124872	G	A
2906	124945	G	A
2907	124950	G	A
2908	125094	G	A
2909	125103	G	A
2910	125112	G	A
2911	125122	G	A
2912	125207	G	A
2913	125581	G	A
2914	125582	G	A
2915	125599	G	A
2916	125743	G	A
2917	125966	G	A
2918	125970	G	A
2919	126004	G	A
2920	126009	G	A
2921	126452	G	A
2922	126792	G	A
2923	126986	G	A
2924	127175	G	A
2925	127430	G	A
2926	127679	G	A
2927	127958	G	A
2928	127973	G	A
2929	128065	G	A
2930	128072	G	A
2931	128317	G	A
2932	128417	G	A
2933	128570	G	A
2934	128579	G	A
2935	128855	G	A
2936	128934	G	A
2937	129044	G	A
2938	129477	G	A
2939	129533	G	A
2940	129608	G	A
2941	129635	G	A
2942	129706	G	A
2943	129924	G	A
2944	474	G	C
2945	931	G	C
2946	3159	G	C
2947	3819	G	C

2948	5318	G	C
2949	7989	G	C
2950	8870	G	C
2951	9367	G	C
2952	12044	G	C
2953	12532	G	C
2954	12838	G	C
2955	12895	G	C
2956	13398	G	C
2957	14189	G	C
2958	15083	G	C
2959	20095	G	C
2960	22683	G	C
2961	23009	G	C
2962	23074	G	C
2963	24364	G	C
2964	27906	G	C
2965	28213	G	C
2966	30865	G	C
2967	31968	G	C
2968	32943	G	C
2969	33168	G	C
2970	35907	G	C
2971	38867	G	C
2972	39559	G	C
2973	43005	G	C
2974	43273	G	C
2975	43727	G	C
2976	46150	G	C
2977	46237	G	C
2978	48731	G	C
2979	49272	G	C
2980	51687	G	C
2981	53009	G	C
2982	53941	G	C
2983	55469	G	C
2984	57283	G	C
2985	57457	G	C
2986	58336	G	C
2987	58609	G	C
2988	58794	G	C
2989	59713	G	C
2990	60447	G	C
2991	60590	G	C
2992	62326	G	C
2993	62410	G	C
2994	62524	G	C
2995	63524	G	C
2996	63576	G	C
2997	63734	G	C

2998	64144	G	C
2999	67917	G	C
3000	68180	G	C
3001	70456	G	C
3002	71936	G	C
3003	77191	G	C
3004	77210	G	C
3005	78713	G	C
3006	78756	G	C
3007	86276	G	C
3008	106100	G	C
3009	109674	G	C
3010	109986	G	C
3011	111369	G	C
3012	113438	G	C
3013	114881	G	C
3014	114969	G	C
3015	115512	G	C
3016	116074	G	C
3017	116737	G	C
3018	119353	G	C
3019	123186	G	C
3020	123664	G	C
3021	124347	G	C
3022	124521	G	C
3023	125602	G	C
3024	126278	G	C
3025	126563	G	C
3026	127345	G	C
3027	127391	G	C
3028	128482	G	C
3029	129117	G	C
3030	129154	G	C
3031	129162	G	C
3032	129261	G	C
3033	129461	G	C
3034	129836	G	C
3035	147	G	T
3036	257	G	T
3037	545	G	T
3038	993	G	T
3039	1800	G	T
3040	1808	G	T
3041	2091	G	T
3042	2119	G	T
3043	2458	G	T
3044	2683	G	T
3045	3041	G	T
3046	3309	G	T
3047	3386	G	T

3048	3641	G	T
3049	3675	G	T
3050	3701	G	T
3051	4325	G	T
3052	4454	G	T
3053	4466	G	T
3054	4661	G	T
3055	4704	G	T
3056	4753	G	T
3057	4946	G	T
3058	5387	G	T
3059	5467	G	T
3060	5502	G	T
3061	6314	G	T
3062	6364	G	T
3063	6546	G	T
3064	6586	G	T
3065	7032	G	T
3066	7110	G	T
3067	7126	G	T
3068	7360	G	T
3069	7545	G	T
3070	7547	G	T
3071	8383	G	T
3072	8763	G	T
3073	9006	G	T
3074	9013	G	T
3075	9348	G	T
3076	9406	G	T
3077	9414	G	T
3078	9667	G	T
3079	9726	G	T
3080	9774	G	T
3081	9973	G	T
3082	10185	G	T
3083	10342	G	T
3084	11311	G	T
3085	11600	G	T
3086	12546	G	T
3087	13278	G	T
3088	13319	G	T
3089	13401	G	T
3090	13509	G	T
3091	14126	G	T
3092	14736	G	T
3093	14747	G	T
3094	15166	G	T
3095	15871	G	T
3096	15939	G	T
3097	16237	G	T

3098	17027	G	T
3099	17830	G	T
3100	18015	G	T
3101	18609	G	T
3102	18793	G	T
3103	19359	G	T
3104	19476	G	T
3105	20533	G	T
3106	21051	G	T
3107	21572	G	T
3108	21864	G	T
3109	22704	G	T
3110	22743	G	T
3111	22926	G	T
3112	22973	G	T
3113	22999	G	T
3114	23391	G	T
3115	23553	G	T
3116	24148	G	T
3117	24370	G	T
3118	25164	G	T
3119	27344	G	T
3120	27476	G	T
3121	27545	G	T
3122	27561	G	T
3123	27797	G	T
3124	28289	G	T
3125	28303	G	T
3126	28330	G	T
3127	28371	G	T
3128	28378	G	T
3129	28450	G	T
3130	28522	G	T
3131	28541	G	T
3132	28645	G	T
3133	28712	G	T
3134	28732	G	T
3135	28907	G	T
3136	28940	G	T
3137	29033	G	T
3138	29150	G	T
3139	29221	G	T
3140	29475	G	T
3141	29739	G	T
3142	29838	G	T
3143	30117	G	T
3144	30414	G	T
3145	30446	G	T
3146	30500	G	T
3147	31027	G	T

3148	31137	G	T
3149	31181	G	T
3150	31339	G	T
3151	31359	G	T
3152	31770	G	T
3153	31842	G	T
3154	32183	G	T
3155	32242	G	T
3156	32259	G	T
3157	32371	G	T
3158	32590	G	T
3159	33240	G	T
3160	33262	G	T
3161	33280	G	T
3162	33288	G	T
3163	33390	G	T
3164	33619	G	T
3165	34663	G	T
3166	34716	G	T
3167	34952	G	T
3168	35112	G	T
3169	36014	G	T
3170	37110	G	T
3171	37488	G	T
3172	39769	G	T
3173	39802	G	T
3174	39853	G	T
3175	41507	G	T
3176	42029	G	T
3177	42795	G	T
3178	43831	G	T
3179	43980	G	T
3180	44240	G	T
3181	44257	G	T
3182	44737	G	T
3183	45639	G	T
3184	45721	G	T
3185	45729	G	T
3186	46071	G	T
3187	46082	G	T
3188	46119	G	T
3189	46129	G	T
3190	46260	G	T
3191	46293	G	T
3192	46656	G	T
3193	46793	G	T
3194	46838	G	T
3195	46900	G	T
3196	47647	G	T
3197	47897	G	T

3198	47968	G	T
3199	47989	G	T
3200	48198	G	T
3201	48258	G	T
3202	48454	G	T
3203	48690	G	T
3204	48858	G	T
3205	49132	G	T
3206	49181	G	T
3207	49337	G	T
3208	49433	G	T
3209	49693	G	T
3210	49892	G	T
3211	49907	G	T
3212	50122	G	T
3213	50754	G	T
3214	51251	G	T
3215	51541	G	T
3216	51660	G	T
3217	52019	G	T
3218	52300	G	T
3219	52403	G	T
3220	52680	G	T
3221	53064	G	T
3222	53150	G	T
3223	53370	G	T
3224	53658	G	T
3225	53674	G	T
3226	54335	G	T
3227	54764	G	T
3228	55423	G	T
3229	55449	G	T
3230	55660	G	T
3231	55761	G	T
3232	55813	G	T
3233	55884	G	T
3234	56172	G	T
3235	56423	G	T
3236	57371	G	T
3237	57466	G	T
3238	57875	G	T
3239	58137	G	T
3240	58148	G	T
3241	58385	G	T
3242	58404	G	T
3243	58598	G	T
3244	59022	G	T
3245	60386	G	T
3246	60443	G	T
3247	60456	G	T

3248	60468	G	T
3249	60475	G	T
3250	60498	G	T
3251	60526	G	T
3252	60632	G	T
3253	60670	G	T
3254	62126	G	T
3255	62229	G	T
3256	62243	G	T
3257	62256	G	T
3258	62315	G	T
3259	62334	G	T
3260	62390	G	T
3261	62433	G	T
3262	62450	G	T
3263	62537	G	T
3264	62586	G	T
3265	62608	G	T
3266	62990	G	T
3267	63175	G	T
3268	63199	G	T
3269	63296	G	T
3270	63517	G	T
3271	63667	G	T
3272	63756	G	T
3273	64165	G	T
3274	64335	G	T
3275	64462	G	T
3276	64775	G	T
3277	64787	G	T
3278	64807	G	T
3279	64941	G	T
3280	65092	G	T
3281	65193	G	T
3282	65501	G	T
3283	65613	G	T
3284	65644	G	T
3285	65660	G	T
3286	65671	G	T
3287	65807	G	T
3288	65854	G	T
3289	66210	G	T
3290	66325	G	T
3291	66904	G	T
3292	66968	G	T
3293	66984	G	T
3294	67006	G	T
3295	67008	G	T
3296	67152	G	T
3297	67159	G	T

3298	67201	G	T
3299	67378	G	T
3300	67457	G	T
3301	67463	G	T
3302	67465	G	T
3303	67472	G	T
3304	67503	G	T
3305	67542	G	T
3306	67670	G	T
3307	67699	G	T
3308	67751	G	T
3309	67872	G	T
3310	67984	G	T
3311	68033	G	T
3312	68087	G	T
3313	68367	G	T
3314	68501	G	T
3315	69090	G	T
3316	69229	G	T
3317	69265	G	T
3318	69295	G	T
3319	69308	G	T
3320	69712	G	T
3321	69752	G	T
3322	70183	G	T
3323	70221	G	T
3324	70261	G	T
3325	70809	G	T
3326	70923	G	T
3327	70971	G	T
3328	71554	G	T
3329	71830	G	T
3330	72100	G	T
3331	72406	G	T
3332	72408	G	T
3333	72732	G	T
3334	73046	G	T
3335	73062	G	T
3336	73091	G	T
3337	73300	G	T
3338	73316	G	T
3339	73337	G	T
3340	73387	G	T
3341	73566	G	T
3342	73628	G	T
3343	73935	G	T
3344	74032	G	T
3345	75164	G	T
3346	75719	G	T
3347	76079	G	T

3348	76732	G	T
3349	76956	G	T
3350	77872	G	T
3351	78090	G	T
3352	78591	G	T
3353	79722	G	T
3354	81502	G	T
3355	81508	G	T
3356	81538	G	T
3357	82206	G	T
3358	82444	G	T
3359	82608	G	T
3360	83144	G	T
3361	83309	G	T
3362	83433	G	T
3363	83530	G	T
3364	83601	G	T
3365	83768	G	T
3366	84027	G	T
3367	84095	G	T
3368	84828	G	T
3369	84883	G	T
3370	85049	G	T
3371	85849	G	T
3372	86011	G	T
3373	86101	G	T
3374	86220	G	T
3375	93242	G	T
3376	105683	G	T
3377	109677	G	T
3378	111336	G	T
3379	111793	G	T
3380	112142	G	T
3381	112162	G	T
3382	112207	G	T
3383	112288	G	T
3384	112361	G	T
3385	112714	G	T
3386	112886	G	T
3387	113624	G	T
3388	113733	G	T
3389	113938	G	T
3390	114040	G	T
3391	114153	G	T
3392	114180	G	T
3393	114227	G	T
3394	114265	G	T
3395	114530	G	T
3396	114736	G	T
3397	114743	G	T

3398	114797	G	T
3399	114837	G	T
3400	114872	G	T
3401	114902	G	T
3402	114978	G	T
3403	114995	G	T
3404	115009	G	T
3405	115017	G	T
3406	115031	G	T
3407	115124	G	T
3408	115161	G	T
3409	115225	G	T
3410	115408	G	T
3411	115527	G	T
3412	115572	G	T
3413	116194	G	T
3414	116393	G	T
3415	116640	G	T
3416	116707	G	T
3417	116723	G	T
3418	117044	G	T
3419	117606	G	T
3420	117759	G	T
3421	118005	G	T
3422	118227	G	T
3423	118801	G	T
3424	118889	G	T
3425	119140	G	T
3426	119309	G	T
3427	119410	G	T
3428	119555	G	T
3429	119576	G	T
3430	119988	G	T
3431	120055	G	T
3432	120172	G	T
3433	120221	G	T
3434	120393	G	T
3435	120403	G	T
3436	120704	G	T
3437	121292	G	T
3438	121672	G	T
3439	121735	G	T
3440	121824	G	T
3441	121899	G	T
3442	121911	G	T
3443	121945	G	T
3444	121983	G	T
3445	122016	G	T
3446	122189	G	T
3447	122609	G	T

3448	122621	G	T
3449	123365	G	T
3450	123980	G	T
3451	124529	G	T
3452	124887	G	T
3453	125370	G	T
3454	125434	G	T
3455	125499	G	T
3456	125523	G	T
3457	125546	G	T
3458	125819	G	T
3459	125834	G	T
3460	125843	G	T
3461	125953	G	T
3462	125992	G	T
3463	125997	G	T
3464	126070	G	T
3465	126094	G	T
3466	126118	G	T
3467	126121	G	T
3468	126128	G	T
3469	126133	G	T
3470	126139	G	T
3471	126140	G	T
3472	126343	G	T
3473	126345	G	T
3474	126355	G	T
3475	126506	G	T
3476	126556	G	T
3477	126624	G	T
3478	126633	G	T
3479	126681	G	T
3480	126924	G	T
3481	127039	G	T
3482	127041	G	T
3483	127073	G	T
3484	127195	G	T
3485	127255	G	T
3486	127272	G	T
3487	127302	G	T
3488	127307	G	T
3489	127420	G	T
3490	127462	G	T
3491	127579	G	T
3492	127586	G	T
3493	127623	G	T
3494	127633	G	T
3495	127666	G	T
3496	127804	G	T
3497	127866	G	T

3498	127877	G	T
3499	127894	G	T
3500	128044	G	T
3501	128123	G	T
3502	128272	G	T
3503	128302	G	T
3504	128489	G	T
3505	128518	G	T
3506	128591	G	T
3507	128674	G	T
3508	128804	G	T
3509	128854	G	T
3510	128879	G	T
3511	128916	G	T
3512	129040	G	T
3513	129178	G	T
3514	129369	G	T
3515	129385	G	T
3516	129487	G	T
3517	129658	G	T
3518	129851	G	T
3519	129957	G	T
3520	139673	G	T
3521	6	T	A
3522	171	T	A
3523	339	T	A
3524	442	T	A
3525	3047	T	A
3526	3827	T	A
3527	4192	T	A
3528	4653	T	A
3529	4679	T	A
3530	4758	T	A
3531	4768	T	A
3532	4886	T	A
3533	4960	T	A
3534	5861	T	A
3535	6315	T	A
3536	6397	T	A
3537	6419	T	A
3538	6449	T	A
3539	6452	T	A
3540	6461	T	A
3541	6571	T	A
3542	6678	T	A
3543	6682	T	A
3544	7085	T	A
3545	8432	T	A
3546	9040	T	A
3547	9298	T	A

3548	9368	T	A
3549	9957	T	A
3550	10253	T	A
3551	10591	T	A
3552	11672	T	A
3553	12759	T	A
3554	13246	T	A
3555	13895	T	A
3556	14366	T	A
3557	14514	T	A
3558	14629	T	A
3559	14950	T	A
3560	15814	T	A
3561	16867	T	A
3562	17935	T	A
3563	17970	T	A
3564	17971	T	A
3565	18028	T	A
3566	27543	T	A
3567	27757	T	A
3568	28206	T	A
3569	28304	T	A
3570	28540	T	A
3571	28860	T	A
3572	29404	T	A
3573	29981	T	A
3574	30339	T	A
3575	30987	T	A
3576	32189	T	A
3577	32246	T	A
3578	32276	T	A
3579	32284	T	A
3580	32630	T	A
3581	32887	T	A
3582	34860	T	A
3583	34861	T	A
3584	35409	T	A
3585	35606	T	A
3586	36823	T	A
3587	36843	T	A
3588	37080	T	A
3589	37405	T	A
3590	43049	T	A
3591	43050	T	A
3592	44350	T	A
3593	45110	T	A
3594	45251	T	A
3595	45560	T	A
3596	45715	T	A
3597	45929	T	A

3598	45937	T	A
3599	45938	T	A
3600	46000	T	A
3601	46257	T	A
3602	47979	T	A
3603	48051	T	A
3604	48309	T	A
3605	49140	T	A
3606	49328	T	A
3607	49329	T	A
3608	49332	T	A
3609	49406	T	A
3610	49867	T	A
3611	49910	T	A
3612	52149	T	A
3613	52195	T	A
3614	52198	T	A
3615	52675	T	A
3616	52851	T	A
3617	53498	T	A
3618	53662	T	A
3619	53823	T	A
3620	55723	T	A
3621	58388	T	A
3622	58439	T	A
3623	58635	T	A
3624	60517	T	A
3625	60835	T	A
3626	60896	T	A
3627	61113	T	A
3628	62155	T	A
3629	62284	T	A
3630	62338	T	A
3631	62339	T	A
3632	62438	T	A
3633	62441	T	A
3634	62465	T	A
3635	62466	T	A
3636	62475	T	A
3637	62513	T	A
3638	62530	T	A
3639	62628	T	A
3640	63424	T	A
3641	63725	T	A
3642	63839	T	A
3643	63840	T	A
3644	64296	T	A
3645	64827	T	A
3646	64953	T	A
3647	64954	T	A

3648	64955	T	A
3649	65327	T	A
3650	65328	T	A
3651	67099	T	A
3652	67198	T	A
3653	67215	T	A
3654	67218	T	A
3655	67471	T	A
3656	67500	T	A
3657	67693	T	A
3658	67822	T	A
3659	67898	T	A
3660	68318	T	A
3661	68319	T	A
3662	68670	T	A
3663	69683	T	A
3664	70170	T	A
3665	70875	T	A
3666	70884	T	A
3667	72307	T	A
3668	72369	T	A
3669	73375	T	A
3670	73793	T	A
3671	75990	T	A
3672	77351	T	A
3673	77506	T	A
3674	77830	T	A
3675	78079	T	A
3676	78692	T	A
3677	78701	T	A
3678	79498	T	A
3679	79514	T	A
3680	79624	T	A
3681	83017	T	A
3682	83445	T	A
3683	83446	T	A
3684	84266	T	A
3685	86153	T	A
3686	111329	T	A
3687	111774	T	A
3688	113675	T	A
3689	113955	T	A
3690	113993	T	A
3691	114017	T	A
3692	114321	T	A
3693	114322	T	A
3694	114323	T	A
3695	114330	T	A
3696	114599	T	A
3697	114835	T	A

3698	114896	T	A
3699	114937	T	A
3700	115232	T	A
3701	115269	T	A
3702	115374	T	A
3703	115520	T	A
3704	115602	T	A
3705	115864	T	A
3706	115898	T	A
3707	116567	T	A
3708	116686	T	A
3709	117332	T	A
3710	117798	T	A
3711	118269	T	A
3712	121778	T	A
3713	122328	T	A
3714	122567	T	A
3715	123916	T	A
3716	124860	T	A
3717	124989	T	A
3718	126836	T	A
3719	127479	T	A
3720	127696	T	A
3721	127850	T	A
3722	127865	T	A
3723	128630	T	A
3724	128666	T	A
3725	129389	T	A
3726	109	T	C
3727	387	T	C
3728	542	T	C
3729	883	T	C
3730	1745	T	C
3731	1927	T	C
3732	2247	T	C
3733	2281	T	C
3734	3038	T	C
3735	3091	T	C
3736	3229	T	C
3737	3380	T	C
3738	3457	T	C
3739	3963	T	C
3740	4010	T	C
3741	4419	T	C
3742	4442	T	C
3743	4518	T	C
3744	4691	T	C
3745	4730	T	C
3746	4800	T	C
3747	4889	T	C

3748	5635	T	C
3749	5771	T	C
3750	5816	T	C
3751	5842	T	C
3752	6674	T	C
3753	6725	T	C
3754	6762	T	C
3755	6944	T	C
3756	6966	T	C
3757	7122	T	C
3758	7427	T	C
3759	7613	T	C
3760	8015	T	C
3761	8186	T	C
3762	8195	T	C
3763	8282	T	C
3764	8300	T	C
3765	8393	T	C
3766	8799	T	C
3767	8838	T	C
3768	8868	T	C
3769	8992	T	C
3770	9029	T	C
3771	9764	T	C
3772	10003	T	C
3773	10080	T	C
3774	10081	T	C
3775	10088	T	C
3776	10404	T	C
3777	10421	T	C
3778	10438	T	C
3779	10523	T	C
3780	10640	T	C
3781	10718	T	C
3782	10745	T	C
3783	10946	T	C
3784	11147	T	C
3785	11228	T	C
3786	11273	T	C
3787	11336	T	C
3788	11363	T	C
3789	12140	T	C
3790	12357	T	C
3791	12386	T	C
3792	12569	T	C
3793	12603	T	C
3794	13461	T	C
3795	13566	T	C
3796	13752	T	C
3797	13815	T	C

3798	13961	T	C
3799	14015	T	C
3800	14034	T	C
3801	14214	T	C
3802	14377	T	C
3803	14404	T	C
3804	14470	T	C
3805	14929	T	C
3806	14943	T	C
3807	15786	T	C
3808	16468	T	C
3809	16692	T	C
3810	16699	T	C
3811	16742	T	C
3812	17077	T	C
3813	17332	T	C
3814	17559	T	C
3815	17821	T	C
3816	18108	T	C
3817	18109	T	C
3818	18163	T	C
3819	18246	T	C
3820	18377	T	C
3821	18492	T	C
3822	19521	T	C
3823	20062	T	C
3824	20355	T	C
3825	20641	T	C
3826	20702	T	C
3827	21402	T	C
3828	21444	T	C
3829	21957	T	C
3830	22015	T	C
3831	22239	T	C
3832	22337	T	C
3833	22560	T	C
3834	22593	T	C
3835	23348	T	C
3836	23527	T	C
3837	24053	T	C
3838	24124	T	C
3839	24152	T	C
3840	24175	T	C
3841	24733	T	C
3842	24778	T	C
3843	24787	T	C
3844	25075	T	C
3845	25485	T	C
3846	25639	T	C
3847	25945	T	C

3848	26266	T	C
3849	26338	T	C
3850	27962	T	C
3851	28165	T	C
3852	28321	T	C
3853	28340	T	C
3854	28487	T	C
3855	28533	T	C
3856	28715	T	C
3857	28790	T	C
3858	28881	T	C
3859	28945	T	C
3860	28952	T	C
3861	28994	T	C
3862	29105	T	C
3863	29218	T	C
3864	29363	T	C
3865	29447	T	C
3866	29603	T	C
3867	29631	T	C
3868	29672	T	C
3869	29692	T	C
3870	29767	T	C
3871	30099	T	C
3872	30186	T	C
3873	30252	T	C
3874	30407	T	C
3875	30420	T	C
3876	30455	T	C
3877	30482	T	C
3878	30893	T	C
3879	31097	T	C
3880	31278	T	C
3881	31289	T	C
3882	31542	T	C
3883	31948	T	C
3884	31976	T	C
3885	32889	T	C
3886	33065	T	C
3887	33551	T	C
3888	34011	T	C
3889	34483	T	C
3890	35035	T	C
3891	35331	T	C
3892	35372	T	C
3893	35601	T	C
3894	35704	T	C
3895	35848	T	C
3896	36044	T	C
3897	36138	T	C

3898	36250	T	C
3899	36258	T	C
3900	36330	T	C
3901	36629	T	C
3902	36661	T	C
3903	36781	T	C
3904	37234	T	C
3905	37255	T	C
3906	37260	T	C
3907	37390	T	C
3908	37944	T	C
3909	37980	T	C
3910	38746	T	C
3911	38749	T	C
3912	39499	T	C
3913	39517	T	C
3914	39628	T	C
3915	40820	T	C
3916	40991	T	C
3917	41075	T	C
3918	42497	T	C
3919	42602	T	C
3920	42957	T	C
3921	42979	T	C
3922	43214	T	C
3923	43232	T	C
3924	43241	T	C
3925	43789	T	C
3926	44142	T	C
3927	45270	T	C
3928	45413	T	C
3929	45854	T	C
3930	46328	T	C
3931	46502	T	C
3932	47123	T	C
3933	47546	T	C
3934	48829	T	C
3935	48893	T	C
3936	49080	T	C
3937	49159	T	C
3938	49416	T	C
3939	49739	T	C
3940	49775	T	C
3941	49871	T	C
3942	49889	T	C
3943	49900	T	C
3944	49905	T	C
3945	50145	T	C
3946	50403	T	C
3947	51254	T	C

3948	51748	T	C
3949	51883	T	C
3950	52068	T	C
3951	52209	T	C
3952	52572	T	C
3953	52846	T	C
3954	53279	T	C
3955	53544	T	C
3956	53556	T	C
3957	53654	T	C
3958	54305	T	C
3959	54491	T	C
3960	54866	T	C
3961	55406	T	C
3962	55457	T	C
3963	55568	T	C
3964	55678	T	C
3965	55708	T	C
3966	55991	T	C
3967	56103	T	C
3968	56154	T	C
3969	56167	T	C
3970	56575	T	C
3971	56734	T	C
3972	56955	T	C
3973	56989	T	C
3974	57025	T	C
3975	57316	T	C
3976	57826	T	C
3977	58337	T	C
3978	58456	T	C
3979	58647	T	C
3980	58677	T	C
3981	59102	T	C
3982	59295	T	C
3983	59721	T	C
3984	60024	T	C
3985	60367	T	C
3986	60379	T	C
3987	60398	T	C
3988	60418	T	C
3989	60529	T	C
3990	60691	T	C
3991	61211	T	C
3992	61278	T	C
3993	61295	T	C
3994	61629	T	C
3995	61881	T	C
3996	62044	T	C
3997	62301	T	C

3998	62307	T	C
3999	62322	T	C
4000	62430	T	C
4001	62500	T	C
4002	62504	T	C
4003	62532	T	C
4004	62620	T	C
4005	62761	T	C
4006	62804	T	C
4007	63151	T	C
4008	63438	T	C
4009	63454	T	C
4010	63493	T	C
4011	63586	T	C
4012	63819	T	C
4013	63844	T	C
4014	63873	T	C
4015	64132	T	C
4016	64138	T	C
4017	64189	T	C
4018	64741	T	C
4019	64767	T	C
4020	64816	T	C
4021	64817	T	C
4022	64832	T	C
4023	65163	T	C
4024	65222	T	C
4025	65296	T	C
4026	65539	T	C
4027	65771	T	C
4028	65992	T	C
4029	66025	T	C
4030	66110	T	C
4031	66150	T	C
4032	66399	T	C
4033	66528	T	C
4034	66869	T	C
4035	67024	T	C
4036	67132	T	C
4037	67480	T	C
4038	67523	T	C
4039	67668	T	C
4040	67703	T	C
4041	67856	T	C
4042	67886	T	C
4043	67937	T	C
4044	68066	T	C
4045	68264	T	C
4046	68298	T	C
4047	68504	T	C

4048	68622	T	C
4049	68970	T	C
4050	69594	T	C
4051	70276	T	C
4052	70924	T	C
4053	70926	T	C
4054	71740	T	C
4055	71953	T	C
4056	72108	T	C
4057	72182	T	C
4058	72392	T	C
4059	72759	T	C
4060	73000	T	C
4061	73031	T	C
4062	73068	T	C
4063	73202	T	C
4064	73806	T	C
4065	73966	T	C
4066	74111	T	C
4067	74153	T	C
4068	74287	T	C
4069	74692	T	C
4070	75100	T	C
4071	75265	T	C
4072	75721	T	C
4073	75745	T	C
4074	76094	T	C
4075	76109	T	C
4076	76253	T	C
4077	76572	T	C
4078	76576	T	C
4079	76991	T	C
4080	77623	T	C
4081	77846	T	C
4082	78498	T	C
4083	79119	T	C
4084	79122	T	C
4085	79739	T	C
4086	80286	T	C
4087	80915	T	C
4088	81016	T	C
4089	81023	T	C
4090	81483	T	C
4091	81604	T	C
4092	81669	T	C
4093	81678	T	C
4094	81821	T	C
4095	82064	T	C
4096	82423	T	C
4097	82452	T	C

4098	82597	T	C
4099	82764	T	C
4100	82773	T	C
4101	82954	T	C
4102	82966	T	C
4103	83056	T	C
4104	83132	T	C
4105	83960	T	C
4106	84183	T	C
4107	84370	T	C
4108	85026	T	C
4109	85188	T	C
4110	85264	T	C
4111	85484	T	C
4112	86218	T	C
4113	86222	T	C
4114	105675	T	C
4115	111207	T	C
4116	111685	T	C
4117	111695	T	C
4118	111902	T	C
4119	112003	T	C
4120	112069	T	C
4121	112152	T	C
4122	112472	T	C
4123	112885	T	C
4124	112954	T	C
4125	112967	T	C
4126	113707	T	C
4127	114088	T	C
4128	114131	T	C
4129	114159	T	C
4130	114627	T	C
4131	114702	T	C
4132	114800	T	C
4133	114845	T	C
4134	115230	T	C
4135	115282	T	C
4136	115331	T	C
4137	115490	T	C
4138	115697	T	C
4139	115714	T	C
4140	115757	T	C
4141	115999	T	C
4142	116068	T	C
4143	116282	T	C
4144	116434	T	C
4145	116636	T	C
4146	116739	T	C
4147	116782	T	C

4148	116938	T	C
4149	117132	T	C
4150	117588	T	C
4151	117685	T	C
4152	117975	T	C
4153	118034	T	C
4154	118476	T	C
4155	118774	T	C
4156	118873	T	C
4157	119126	T	C
4158	119138	T	C
4159	119201	T	C
4160	119586	T	C
4161	119651	T	C
4162	119763	T	C
4163	119837	T	C
4164	120126	T	C
4165	120153	T	C
4166	120285	T	C
4167	120818	T	C
4168	121157	T	C
4169	121358	T	C
4170	121472	T	C
4171	121598	T	C
4172	121609	T	C
4173	122232	T	C
4174	122288	T	C
4175	122477	T	C
4176	122548	T	C
4177	122841	T	C
4178	123019	T	C
4179	123118	T	C
4180	123308	T	C
4181	123315	T	C
4182	123318	T	C
4183	123371	T	C
4184	123696	T	C
4185	123755	T	C
4186	123903	T	C
4187	124584	T	C
4188	124724	T	C
4189	124764	T	C
4190	124847	T	C
4191	124944	T	C
4192	125109	T	C
4193	125368	T	C
4194	125577	T	C
4195	125703	T	C
4196	125946	T	C
4197	126032	T	C

4198	126082	T	C
4199	126105	T	C
4200	126116	T	C
4201	126144	T	C
4202	126277	T	C
4203	126386	T	C
4204	126689	T	C
4205	126716	T	C
4206	126885	T	C
4207	126894	T	C
4208	126929	T	C
4209	126999	T	C
4210	127108	T	C
4211	127139	T	C
4212	127263	T	C
4213	127318	T	C
4214	127673	T	C
4215	127813	T	C
4216	128039	T	C
4217	128345	T	C
4218	128441	T	C
4219	128786	T	C
4220	128906	T	C
4221	128981	T	C
4222	129075	T	C
4223	129239	T	C
4224	129415	T	C
4225	129441	T	C
4226	129576	T	C
4227	129596	T	C
4228	129734	T	C
4229	13	T	G
4230	131	T	G
4231	139	T	G
4232	235	T	G
4233	251	T	G
4234	324	T	G
4235	1762	T	G
4236	1763	T	G
4237	1775	T	G
4238	2029	T	G
4239	2090	T	G
4240	2108	T	G
4241	2112	T	G
4242	2143	T	G
4243	2607	T	G
4244	2723	T	G
4245	2736	T	G
4246	3051	T	G
4247	3260	T	G

4248	3449	T	G
4249	3831	T	G
4250	4155	T	G
4251	4347	T	G
4252	4437	T	G
4253	4504	T	G
4254	4554	T	G
4255	4594	T	G
4256	4628	T	G
4257	4795	T	G
4258	5404	T	G
4259	5604	T	G
4260	5828	T	G
4261	5844	T	G
4262	5857	T	G
4263	5872	T	G
4264	6278	T	G
4265	6308	T	G
4266	6411	T	G
4267	6483	T	G
4268	6631	T	G
4269	6637	T	G
4270	6681	T	G
4271	6695	T	G
4272	6709	T	G
4273	6774	T	G
4274	6823	T	G
4275	6825	T	G
4276	6874	T	G
4277	6952	T	G
4278	6997	T	G
4279	7058	T	G
4280	7243	T	G
4281	7401	T	G
4282	7437	T	G
4283	7474	T	G
4284	7590	T	G
4285	7869	T	G
4286	7873	T	G
4287	7914	T	G
4288	7972	T	G
4289	7999	T	G
4290	8114	T	G
4291	8884	T	G
4292	8888	T	G
4293	8892	T	G
4294	8961	T	G
4295	9156	T	G
4296	9413	T	G
4297	9615	T	G

4298	9783	T	G
4299	9981	T	G
4300	10127	T	G
4301	10153	T	G
4302	10262	T	G
4303	10291	T	G
4304	10304	T	G
4305	10320	T	G
4306	11384	T	G
4307	11670	T	G
4308	11815	T	G
4309	12629	T	G
4310	12708	T	G
4311	12932	T	G
4312	13289	T	G
4313	13377	T	G
4314	13557	T	G
4315	13891	T	G
4316	13994	T	G
4317	14045	T	G
4318	14280	T	G
4319	14282	T	G
4320	14547	T	G
4321	14763	T	G
4322	14823	T	G
4323	15834	T	G
4324	15865	T	G
4325	15940	T	G
4326	15941	T	G
4327	16301	T	G
4328	16711	T	G
4329	17583	T	G
4330	17881	T	G
4331	17964	T	G
4332	18001	T	G
4333	18014	T	G
4334	18275	T	G
4335	18305	T	G
4336	18552	T	G
4337	18669	T	G
4338	18811	T	G
4339	18970	T	G
4340	19395	T	G
4341	19789	T	G
4342	21126	T	G
4343	21360	T	G
4344	21577	T	G
4345	21927	T	G
4346	22974	T	G
4347	23067	T	G

4348	23511	T	G
4349	24298	T	G
4350	25588	T	G
4351	26417	T	G
4352	27025	T	G
4353	27054	T	G
4354	27197	T	G
4355	27544	T	G
4356	27658	T	G
4357	27709	T	G
4358	27866	T	G
4359	27875	T	G
4360	27953	T	G
4361	27965	T	G
4362	27982	T	G
4363	28117	T	G
4364	28157	T	G
4365	28535	T	G
4366	28728	T	G
4367	28797	T	G
4368	28865	T	G
4369	28886	T	G
4370	28922	T	G
4371	29037	T	G
4372	29040	T	G
4373	29457	T	G
4374	29471	T	G
4375	30008	T	G
4376	30092	T	G
4377	30156	T	G
4378	30235	T	G
4379	30241	T	G
4380	30263	T	G
4381	30301	T	G
4382	30336	T	G
4383	30437	T	G
4384	31089	T	G
4385	31159	T	G
4386	31340	T	G
4387	31374	T	G
4388	31504	T	G
4389	32139	T	G
4390	32243	T	G
4391	32252	T	G
4392	32267	T	G
4393	32571	T	G
4394	32660	T	G
4395	32672	T	G
4396	32998	T	G
4397	33284	T	G

4398	33286	T	G
4399	33449	T	G
4400	34389	T	G
4401	34419	T	G
4402	34599	T	G
4403	34728	T	G
4404	34755	T	G
4405	34998	T	G
4406	35214	T	G
4407	35415	T	G
4408	35585	T	G
4409	35629	T	G
4410	36288	T	G
4411	36354	T	G
4412	36679	T	G
4413	37026	T	G
4414	37107	T	G
4415	37109	T	G
4416	37268	T	G
4417	37418	T	G
4418	37424	T	G
4419	37436	T	G
4420	37439	T	G
4421	38551	T	G
4422	39591	T	G
4423	39821	T	G
4424	43252	T	G
4425	43411	T	G
4426	43423	T	G
4427	43775	T	G
4428	43918	T	G
4429	44224	T	G
4430	44667	T	G
4431	44736	T	G
4432	44774	T	G
4433	44974	T	G
4434	45272	T	G
4435	45309	T	G
4436	45568	T	G
4437	45674	T	G
4438	45861	T	G
4439	46063	T	G
4440	46222	T	G
4441	46410	T	G
4442	46657	T	G
4443	47412	T	G
4444	47529	T	G
4445	48003	T	G
4446	48088	T	G
4447	48108	T	G

4448	49112	T	G
4449	49370	T	G
4450	49438	T	G
4451	49562	T	G
4452	49624	T	G
4453	49734	T	G
4454	49803	T	G
4455	50255	T	G
4456	50297	T	G
4457	50712	T	G
4458	50737	T	G
4459	51634	T	G
4460	51667	T	G
4461	51784	T	G
4462	51937	T	G
4463	52049	T	G
4464	52196	T	G
4465	52316	T	G
4466	52484	T	G
4467	52553	T	G
4468	52858	T	G
4469	53236	T	G
4470	53464	T	G
4471	53678	T	G
4472	53750	T	G
4473	53958	T	G
4474	54077	T	G
4475	54356	T	G
4476	55064	T	G
4477	55178	T	G
4478	55220	T	G
4479	55285	T	G
4480	55499	T	G
4481	55669	T	G
4482	55824	T	G
4483	55957	T	G
4484	56097	T	G
4485	56150	T	G
4486	56161	T	G
4487	58010	T	G
4488	58316	T	G
4489	58357	T	G
4490	58383	T	G
4491	58429	T	G
4492	58540	T	G
4493	58702	T	G
4494	58771	T	G
4495	58877	T	G
4496	59235	T	G
4497	60439	T	G

4498	60492	T	G
4499	60510	T	G
4500	60571	T	G
4501	60597	T	G
4502	60748	T	G
4503	60834	T	G
4504	60870	T	G
4505	61122	T	G
4506	61685	T	G
4507	62111	T	G
4508	62142	T	G
4509	62270	T	G
4510	62295	T	G
4511	62333	T	G
4512	62409	T	G
4513	62431	T	G
4514	62446	T	G
4515	62484	T	G
4516	62497	T	G
4517	62510	T	G
4518	62577	T	G
4519	63502	T	G
4520	63512	T	G
4521	63596	T	G
4522	63988	T	G
4523	64106	T	G
4524	64198	T	G
4525	64295	T	G
4526	64457	T	G
4527	64573	T	G
4528	64829	T	G
4529	64838	T	G
4530	64910	T	G
4531	65251	T	G
4532	65478	T	G
4533	65575	T	G
4534	65620	T	G
4535	65714	T	G
4536	65985	T	G
4537	66003	T	G
4538	66139	T	G
4539	66196	T	G
4540	66665	T	G
4541	66803	T	G
4542	66810	T	G
4543	66935	T	G
4544	67063	T	G
4545	67213	T	G
4546	67366	T	G
4547	67711	T	G

4548	67762	T	G
4549	67946	T	G
4550	68002	T	G
4551	68024	T	G
4552	68063	T	G
4553	68195	T	G
4554	68274	T	G
4555	68494	T	G
4556	68502	T	G
4557	68683	T	G
4558	68713	T	G
4559	68757	T	G
4560	68815	T	G
4561	69015	T	G
4562	69093	T	G
4563	69825	T	G
4564	70528	T	G
4565	70603	T	G
4566	70773	T	G
4567	71041	T	G
4568	71062	T	G
4569	71286	T	G
4570	71385	T	G
4571	71456	T	G
4572	71811	T	G
4573	71819	T	G
4574	71836	T	G
4575	71886	T	G
4576	71904	T	G
4577	71942	T	G
4578	72027	T	G
4579	72164	T	G
4580	72235	T	G
4581	72238	T	G
4582	73051	T	G
4583	73083	T	G
4584	73308	T	G
4585	73396	T	G
4586	73737	T	G
4587	73810	T	G
4588	73887	T	G
4589	75077	T	G
4590	75820	T	G
4591	75840	T	G
4592	76435	T	G
4593	76522	T	G
4594	76831	T	G
4595	77169	T	G
4596	77171	T	G
4597	77833	T	G

4598	78658	T	G
4599	78844	T	G
4600	79248	T	G
4601	79505	T	G
4602	79987	T	G
4603	80280	T	G
4604	80918	T	G
4605	81467	T	G
4606	81601	T	G
4607	81632	T	G
4608	81646	T	G
4609	81683	T	G
4610	81706	T	G
4611	81724	T	G
4612	82162	T	G
4613	82517	T	G
4614	82903	T	G
4615	83160	T	G
4616	83302	T	G
4617	83312	T	G
4618	83362	T	G
4619	83401	T	G
4620	83634	T	G
4621	83901	T	G
4622	84150	T	G
4623	84281	T	G
4624	84374	T	G
4625	84431	T	G
4626	84700	T	G
4627	84781	T	G
4628	84945	T	G
4629	85051	T	G
4630	85055	T	G
4631	85140	T	G
4632	85350	T	G
4633	85353	T	G
4634	85512	T	G
4635	85856	T	G
4636	85863	T	G
4637	86032	T	G
4638	86258	T	G
4639	111070	T	G
4640	111287	T	G
4641	111348	T	G
4642	111359	T	G
4643	111518	T	G
4644	111563	T	G
4645	112014	T	G
4646	112018	T	G
4647	112033	T	G

4648	112115	T	G
4649	112161	T	G
4650	112252	T	G
4651	112634	T	G
4652	113291	T	G
4653	113577	T	G
4654	113747	T	G
4655	113836	T	G
4656	113862	T	G
4657	113926	T	G
4658	113952	T	G
4659	114063	T	G
4660	114237	T	G
4661	114249	T	G
4662	114388	T	G
4663	114501	T	G
4664	114580	T	G
4665	114811	T	G
4666	114879	T	G
4667	114889	T	G
4668	114890	T	G
4669	114899	T	G
4670	114976	T	G
4671	115096	T	G
4672	115097	T	G
4673	115102	T	G
4674	115104	T	G
4675	115110	T	G
4676	115340	T	G
4677	115369	T	G
4678	115835	T	G
4679	115984	T	G
4680	116140	T	G
4681	116192	T	G
4682	116443	T	G
4683	116464	T	G
4684	116795	T	G
4685	116883	T	G
4686	117435	T	G
4687	117721	T	G
4688	118029	T	G
4689	118196	T	G
4690	118343	T	G
4691	118344	T	G
4692	118377	T	G
4693	118650	T	G
4694	118681	T	G
4695	118847	T	G
4696	118901	T	G
4697	118967	T	G

4698	119084	T	G
4699	119573	T	G
4700	119904	T	G
4701	120049	T	G
4702	120088	T	G
4703	120165	T	G
4704	120207	T	G
4705	120231	T	G
4706	120300	T	G
4707	120305	T	G
4708	120346	T	G
4709	120883	T	G
4710	121191	T	G
4711	121493	T	G
4712	121506	T	G
4713	121552	T	G
4714	121714	T	G
4715	121741	T	G
4716	121795	T	G
4717	121854	T	G
4718	121910	T	G
4719	122010	T	G
4720	122090	T	G
4721	122354	T	G
4722	122482	T	G
4723	122561	T	G
4724	122620	T	G
4725	122717	T	G
4726	123507	T	G
4727	123597	T	G
4728	123744	T	G
4729	123815	T	G
4730	124139	T	G
4731	124185	T	G
4732	124305	T	G
4733	124607	T	G
4734	124722	T	G
4735	124801	T	G
4736	124947	T	G
4737	125053	T	G
4738	125057	T	G
4739	125136	T	G
4740	125363	T	G
4741	125395	T	G
4742	125509	T	G
4743	125515	T	G
4744	125576	T	G
4745	125854	T	G
4746	126039	T	G
4747	126041	T	G

4748	126101	T	G
4749	126145	T	G
4750	126295	T	G
4751	126331	T	G
4752	126339	T	G
4753	126342	T	G
4754	126639	T	G
4755	126754	T	G
4756	126810	T	G
4757	126822	T	G
4758	126830	T	G
4759	126857	T	G
4760	126859	T	G
4761	126865	T	G
4762	126871	T	G
4763	126993	T	G
4764	127063	T	G
4765	127196	T	G
4766	127218	T	G
4767	127267	T	G
4768	127284	T	G
4769	127457	T	G
4770	127501	T	G
4771	127509	T	G
4772	127518	T	G
4773	127595	T	G
4774	127676	T	G
4775	127712	T	G
4776	127715	T	G
4777	127743	T	G
4778	127772	T	G
4779	127793	T	G
4780	127819	T	G
4781	127910	T	G
4782	128053	T	G
4783	128073	T	G
4784	128098	T	G
4785	128263	T	G
4786	128501	T	G
4787	128509	T	G
4788	128524	T	G
4789	128635	T	G
4790	128890	T	G
4791	128917	T	G
4792	128951	T	G
4793	128966	T	G
4794	129026	T	G
4795	129057	T	G
4796	129101	T	G
4797	129260	T	G

4798	129348	T	G
4799	129352	T	G
4800	129366	T	G
4801	129388	T	G
4802	129473	T	G
4803	129826	T	G

Supplemental Table 3. Statistics of genes in the plastid potato genome containing SNPs and Indels

Gene name	Function description	Nmuber of SNPs		Number of Indels		Indel affect on gene frame
		exon	intron	exon	intron	
psbA	photosystem II protein D1	30	-	-	-	
matK	maturase K	67	-	2	-	6bp insertion
rps16	ribosomal protein S16	3	41	-	3	
psbK	photosystem II protein K	2	-	-	-	
psbI	photosystem II protein I	3	-	-	-	
atpA	ATP synthase CF1 alpha subunit	44	-	-	-	
atpF	ATP synthase CF0 B subunit	9	27	-	3	
atpH	ATP synthase CF0 C subunit	6	-	-	-	
atpI	ATP synthase CF0 A subunit	8	-	-	-	
rps2	ribosomal protein S2	8	-	-	-	
rpoC2	RNA polymerase beta subunit	119	-	-	-	
rpoC1	RNA polymerase beta subunit	45	28		2	
rpoB	RNA polymerase beta subunit	61	-	-	-	
petN	cytochrome b6/f complex subunit VIII	3	-	-	-	
psbM	photosystem II protein M	1	-	-	-	
psbD	photosystem II protein D2	27	-	-	-	
psbC	photosystem II 44 kDa protein	76	-	-	-	
psbZ	photosystem II protein Z	5	-	-	-	
rps14	ribosomal protein S14	5	-	-	-	
psaB	photosystem I P700 apoprotein A2	50	-	-	-	
psaA	photosystem I P700 apoprotein A1	33	-	-	-	
ycf3	photosystem I assembly protein Ycf3	6	46	-	5	
rps4	ribosomal protein S4	10	-	-	-	
ndhJ	NADH dehydrogenase subunit J	8	-	-	-	
ndhK	NADH dehydrogenase subunit K	12	-	-	-	
ndhC	NADH dehydrogenase subunit 3	13	-	-	-	
atpE	ATP synthase CF1 epsilon subunit	10	-	1	-	2bp insertion
atpB	ATP synthase CF1 beta subunit	37	-	-	-	
rbcl	ribulose-1%2C5-bisphosphate carboxylase/oxygenase large subunit	34	-	-	-	

accD	acetyl-CoA carboxylase carboxyltransferase beta subunit	36	-	-	-	
psaI	photosystem I subunit VIII	1	-	3	-	3,12 bp insertion; 1bp deletion
ycf4	photosystem I assembly protein Ycf4	8	-	-	-	
cemA	envelope membrane protein	23	-	-	-	
petA	cytochrome f	66	-	-	-	
psbJ	photosystem II protein J	5	-	-	-	
psbL	photosystem II protein L	10	-	-	-	
psbF	photosystem II protein VI	1	-	-	-	
psbE	photosystem II protein V	5	-	-	-	
petL	cytochrome b6/f complex subunit VI	3	-	-	-	
petG	cytochrome b6/f complex subunit V	9	-	-	-	
psaJ	photosystem I subunit IX	1	-	-	-	
rpl33	ribosomal protein L33	5	-	-	-	
rpl18	ribosomal protein S18	4	-	-	-	
rpl20	ribosomal protein L20	5	-	-	-	
rps12	ribosomal protein S12	1	-	-	-	
clpP	ATP-dependent Clp protease proteolytic subunit	22	72		14	
psbB	photosystem II 47 kDa protein	30	-	1	-	10bp insersion
psbT	photosystem II protein T	2	-	-	-	
psbN	photosystem II protein N	3	-	-	-	
psbH	photosystem II protein H	4	-	-	-	
petB	cytochrome b6	16	31	2	2	1,8bp deletion
petD	cytochrome b6/f complex subunit IV	11	25	-	1	
rpoA	RNA polymerase alpha subunit	23	-	-	-	
rps11	ribosomal protein S11	12	-	-	-	
rpl36	ribosomal protein L36	2	-	-	-	
rps8	ribosomal protein S8	11	-	-	-	
rpL14	ribosomal protein L14	15	-	-	-	
rpL16	ribosomal protein L16	14	44	-	3	
rps3	ribosomal protein S3	16	-	-	-	
rpl22	ribosomal protein L22	24	-	-	-	

rps19	ribosomal protein S19	4	-	-	-	
rpl2	ribosomal protein L2	54	-	2	-	1bp deletion;2bp insertion
ycf2	Ycf2	4	-	-	-	
ndhF	NADH dehydrogenase subunit 5	84	-	1	-	13bp insersion
rpl32	ribosomal protein L32	2	-	-	-	
ccsA	cytochrome c biogenesis protein	48	-	1	-	10bp insersion
ndhD	NADH dehydrogenase subunit 4	43	-	-	-	
psaC	photosystem I subunit VII	7	-	-	-	
ndhE	NADH dehydrogenase subunit 4L	8	-	-	-	
ndhG	NADH dehydrogenase subunit 6	15	-	-	-	
ndhI	NADH dehydrogenase subunit I	12	-	-	-	
ndhA	NADH dehydrogenase subunit 1	24	79	-	12	
ndhH	NADH dehydrogenase subunit 7	41	-	-	-	
rps15	ribosomal protein S15	15	-	-	-	
ycf1	hypothetical chloroplast RF1	381	-	7	-	6bp deletion; 6;9;21bp insersion

Supplemental Table 4. Summary of INDELS in potato plastid genome

Number	Position	Reference genome	New Indels in our study
1	163	AGTTCGAAT	A
2	209	T	TTATTATTATTTCA
3	210	G	GATTATTATTTCAA
4	216	T	TATTTCAAA
5	218	T	TTTAAAA
6	442	T	TAATA
7	446	A	AAAT
8	448	A	AT
9	450	A	AAAT
10	1881	T	TTAAATAA
11	2096	CTTCGTCATTTA	C
12	2574	C	CTTAATT
13	3047	T	TTTTTGA
14	3806	T	TA
15	3958	C	CATAATCGAA
16	4406	CCCGAAGAAAATATCGA	C
17	4490	T	TGAAAGAAGAAATATAAAAA
18	4679	T	TAAA
19	4765	AAAT	A
20	4881	GAATTTAATA	G
21	4888	A	ATATTTC
22	5052	G	GTATTTCCTTAATT
23	5841	GTTTGATCAATTCCAATA	G
24	5909	C	CT
25	5984	A	AATTAATACTTTCT
26	6291	TTTCTATATC	T
27	6301	T	TATAG
28	6312	T	TAAATA
29	6315	TCTATAGATATAAATAAT	T
30	6337	T	TAGATATAGA
31	6356	A	AT
32	6408	A	ACATTATAATATTAATTAATAAAT
33	6411	T	TTATAATATTAATTAATAAATATA
34	6419	T	TTAATTAATAAATATATATAATAAA
35	6434	A	ATATAAT
36	6449	T	TATTTATATAA
37	6450	A	ATTTATATAAATAAAATAAG
38	6451	T	TTTATATAA
39	6452	T	TTATA
40	6456	A	ATAAAT
41	6459	A	AATAAAATAAGACGAATAAACG
42	6568	A	AAATTTCGAATC
43	6646	ATCATCTTAGGAAAAAA	A

44	6851	G	GT
45	6868	C	CT
46	6894	TGTCTGGGCA	T
47	6942	C	CTTT
48	6991	AGTGAATTGAATTAGTA	A
49	7137	TGAAAAAAAAAACGATT	T
50	7186	ATTCCAGCTAACATTTCC	A
51	7532	TC	T
52	7604	CTTCCTTACT	C
53	7629	T	TAAATAACTC
54	7869	T	TTAATAA
55	8014	CT	C
56	8112	T	TTC
57	8114	TGA	T
58	8195	TCAAAAA	T
59	8196	C	CA
60	8358	G	GATTTTAA
61	8393	T	TACATTTAACTAAGAATAAG
62	8662	CTTTATCTCTTTATCT	C
63	8670	CTTTATCT	C
64	8684	TC	T
65	8691	C	CTAAAG
66	8776	A	AG
67	8860	T	TGTTATA
68	8927	TTTTTATA	T
69	8934	A	AT
70	8978	TC	T
71	9040	T	TA
72	9069	AATGC	A
73	9107	C	CCCTATCTTAT
74	9136	A	AC
75	9368	T	TTAGACATTGA
76	9946	C	CTCTATA
77	9978	TATTCATTAA	T
78	10016	TATTAGTACATCATTGA	T
79	10144	A	AATCAG
80	10247	A	ATATTTT
81	10249	A	AT
82	10321	T	TATTAAG
83	10328	A	AT
84	12612	TCA	T
85	12772	A	ATATTAACATAGTGGTAGAAAGAG
86	12773	T	TATTAACATAGTGGTAGAAAGA
87	13246	T	TTTTTA
88	13336	C	CTTTT

89	13391	TGAACGGGAAG	T
90	13547	AAAAT	A
91	13566	T	TATTTATAAGCTAAGATTAAAC
92	13880	GAATTATGACTT	G
93	14270	GATTCAATCCT	G
94	14369	A	AG
95	14411	ATTATTCCACC	A
96	14423	A	AGAGAAATC
97	14460	A	ATTCCATGGG
98	14514	T	TTAATAAA
99	14548	A	ATTAT
100	14773	GA	G
101	14798	CTTTGTTTT	C
102	14802	G	GT
103	14807	T	TAAAAACAA
104	14821	C	CT
105	14942	ATATACATT	A
106	15911	AAGGGATTTCT	A
107	15915	GATTTCTAGGGATTTCT[illegible]	G
108	16698	CT	C
109	16702	TTTTTATTC	T
110	16703	TTTTATTC	T
111	16704	TTTATTC	T
112	16720	A	AAGAAT
113	16721	A	AGAAT
114	16729	A	AGG
115	16867	T	TACCAAATGCAGATAGCGAAA
116	23122	T	TCTTAATAATTA
117	23347	CT	C
118	27434	AAATTG	A
119	27476	GA	G
120	27880	TTCTTC	T
121	27895	G	GT
122	28206	T	TCA
123	28297	AATTTTGT	A
124	28325	CATTTGATTTTTCAAT	C
125	28407	GAATCAAACCC	G
126	28420	T	TA
127	28681	GAATGA	G
128	29071	T	TTGAGA
129	29215	TTATTAG	T
130	29604	A	AATTCTTACTAAATT
131	29818	A	ATTGCG
132	29854	ATTTAGACTTT	A
133	30016	C	CCTATAT

134	30308	A	AATAAAT
135	30329	ATTCATCT	A
136	30338	T	TGA
137	30392	A	ATTTC
138	30435	CGTCAATTTTGGCTAAG	C
139	31234	GGGACACCTATA	G
140	31236	G	GAC
141	31336	A	ATT
142	31337	TTG	T
143	31429	A	AAAAGT
144	32124	AAT	A
145	32228	G	GAAATCTAGTA
146	32246	T	TTTTTG
147	32255	TTTTG	T
148	32257	TTGAATTATTTCAA	T
149	32275	AT	A
150	32326	G	GA
151	32334	AGGTAAATCC	A
152	32450	C	CATAGTGAATAATT
153	32884	T	TG
154	33110	T	TTATA
155	33267	C	CTATT
156	33268	TATTTATTTATTGA	T
157	33470	C	CAAAACA
158	36245	TTC	T
159	36358	G	GCAAAA
160	36372	GA	G
161	36661	T	TC
162	36762	CATATTTTATCACAATTA	C
163	37055	CCCAAAA	C
164	37276	A	ATATGTG
165	37305	CA	C
166	37383	CCCAAGAT	C
167	37404	AT	A
168	37575	A	ACCAGAATAAGATGTT
169	43137	G	GTA
170	43173	T	TTAGTTTTAATAATA
171	43185	ATAATTTT	A
172	43198	A	ATTTAAT
173	43265	ATGAAATAGAAG	A
174	43348	G	GT
175	43443	C	CTAAATA
176	43727	G	GATCTATACCATATCTAAAAAAATCTAATC
177	43989	T	TCATAA
178	44202	GA	G

179	45110	T	TA
180	45314	T	TAAAAA
181	45659	G	GATTTCATACTAATGTAGTAGT
182	45672	T	TGTA
183	45714	ATTAGAAGAACTCGAG/	A
184	45929	T	TA
185	45977	G	GATTTA
186	46000	T	TA
187	46026	CAGTTGATTCGTTCTACT	C
188	46164	G	GA
189	46419	A	AATTTTACTAACAAC
190	46567	TGGAAAGAAAAGAATA(	T
191	46648	GATTGATCGTACTTAAC	G
192	46654	TCGTACTTAA	T
193	46694	C	CTA
194	47442	AAACTG	A
195	47646	T	TGATTTAA
196	47680	A	AT
197	47927	A	ATATTATTAAATAG
198	48118	T	TAATGA
199	48309	T	TAGAAGATAGTTAAGAAA
200	48390	G	GTATAAAT
201	48442	T	TCAAAA
202	48891	T	TC
203	49332	T	TA
204	49577	ACCT	A
205	49582	TTCGAAGCTGC	T
206	49596	CGGTATGCGAATG	C
207	49714	T	TA
208	49738	CT	C
209	49787	G	GAAAGGTAAAATTAGTTAATTGTTGAAAGGCTCAAA
210	49804	A	AG
211	49866	ATTTCTTTGTCTTTTCTTT	A
212	52022	TTTATTTCCATTATAC	T
213	52080	A	ATTACT
214	52143	T	TACTTATCTTATTTCTTCTTTA
215	52144	ACTTAT	A
216	52271	A	AAGAGAATGT
217	52290	G	GAGAAT
218	52297	CAAGA	C
219	52304	T	TAGATCAA
220	52330	T	TTTTATTATTATTTAA
221	52334	ATTATTATTTAATTATTA	A
222	52336	TATTATTTAATTA	T
223	52337	A	AT

224	52342	TTA	T
225	52344	AATTATTATTTATTATTT	A
226	52345	ATTATTAT	A
227	52348	A	AT
228	52351	AT	A
229	52354	TATTATTTC	T
230	52361	TC	T
231	52362	C	CATTATT
232	52375	T	TAATAGTATTAGG
233	52618	A	ATTTCATTTAGTTACAAG
234	52678	TTG	T
235	53192	A	AC
236	53493	ATGGGT	A
237	53657	AGTGAT	A
238	53667	A	AT
239	53823	T	TTA
240	55724	A	AAAAT
241	55743	G	GT
242	56052	C	CATTATTTGTATCTA
243	56153	CT	C
244	56164	CTTTCATAGATTCATAG/	C
245	56165	TTTCATAGA	T
246	58004	GAAGGAT	G
247	58054	AATTAC	A
248	58193	C	CTTTCT
249	58262	C	CTGTAGTT
250	58374	ATTAGATT	A
251	58377	A	AGATTTTC
252	58378	G	GATTTTCT
253	58388	T	TGTTAGATTA
254	58447	A	ATTTCT
255	58451	C	CTTTATA
256	58452	T	TTTATAGGA
257	58550	A	AG
258	58645	G	GAAAC
259	58735	G	GCAA
260	58741	AG	A
261	58818	G	GCTAAATAAATCA
262	60267	CA	C
263	60378	CT	C
264	60416	C	CCTTTATCA
265	60516	AT	A
266	60677	A	AG
267	60681	A	AAG
268	60692	C	CG

269	60693	A	ATTATCATACATATCT
270	60852	T	TATTAA
271	60873	A	AATTAGAATTCAAATTCTT
272	61202	G	GGAAA
273	62155	TTTTTATGGTCTTTATGC	T
274	62258	AAGC	A
275	62266	AT	A
276	62309	CAGAT	C
277	62367	CAATTGGATTTCTTAATT	C
278	62448	TTG	T
279	62449	TG	T
280	62535	T	TAGCAAA
281	62561	A	ATCTGAAT
282	63755	TG	T
283	65166	TTTC	T
284	65206	G	GTTTGTTAAAAA
285	65279	T	TATAATATATGAATTGGTGGACAAACA
286	65345	T	TTCTTCAATC
287	65454	C	CATTAGATTAGTATAGAAAGG
288	65457	T	TAGATTAGTATAGAA
289	65541	TGAAAGTACTG	T
290	66653	T	TTAACTGA
291	66935	T	TTTGTTG
292	66958	TA	T
293	66997	C	CA
294	67035	T	TTTC
295	67366	T	TTTATATG
296	67370	T	TATGTCTGG
297	67730	T	TATTTACTTACGTTACC
298	67996	CCTTTCTTTA	C
299	68039	TGTGA	T
300	68044	A	AGATT
301	68068	T	TTCTAATTCGATCTAAGAAGAAAAAAATC
302	68471	C	CATTGAT
303	68500	CGTCT	C
304	69093	TA	T
305	69170	GA	G
306	69175	T	TG
307	69253	G	GTATTTTAC
308	69256	TTTTACTATGACTTTGC	T
309	69665	T	TTATATA
310	70199	G	GA
311	70213	GA	G
312	71368	TTTA	T
313	71370	T	TA

314	71609	A	ATACATAATATG
315	71673	A	AT
316	72006	AAAGCAAAGAG	A
317	72128	G	GA
318	72232	ATTTTTTC	A
319	72238	TC	T
320	72239	CT	C
321	72302	A	AAT
322	72314	TTC	T
323	72383	T	TGAACTGA
324	72408	GA	G
325	72935	CA	C
326	72969	C	CCTCTTTCTTTTTTTCTACAAGGG
327	72987	CAAGGGCTCTTTCTTAC[illegible]	C
328	73282	C	CCATTACCAT
329	73526	T	TCAAAAA
330	73876	G	GACAGAA
331	75760	T	TTTTATTGACATAGGGTA
332	75998	C	CAACTAAAAAG
333	77139	C	CAAATAAT
334	77217	A	AATAAAG
335	78069	CTTTATAGA	C
336	78076	GA	G
337	78078	T	TAGAGAAAATCA
338	78082	T	TA
339	78765	G	GTATT
340	79618	A	ATATGATATATTAATTGTG
341	79623	AT	A
342	79629	TAATTGTGCTA	T
343	79630	A	AATTGT
344	79659	ATTTTCAC	A
345	81720	C	CATATTATAA
346	81724	T	TTATAAAG
347	81725	T	TATAAA
348	82255	A	ATTAAT
349	82692	T	TGAATGCA
350	82732	TC	T
351	82743	AAAAAG	A
352	82789	A	AT
353	83354	TTACTGAGTAA	T
354	83441	AACTTT	A
355	83577	A	ATTTCATT
356	84266	T	TAGAATTAAGAATTATAAA
357	86128	C	CGA
358	86215	GT	G

359	111071	ATCCTTC	A
360	111304	G	GATAAAAAAAA
361	111326	A	AATTTGATT
362	111330	C	CTTCTGGTTTCAAA
363	113678	C	CTTACTTAT
364	113849	TA	T
365	113945	G	GTTATT
366	113948	A	ATTTTATT
367	113954	TTTTTGA	T
368	113957	TTGATTTTTATATTAAGT	T
369	113982	AT	A
370	113993	T	TCATTTCTA
371	114058	T	TGAAATAAAGATACCAGTCAATAG
372	114130	CT	C
373	114132	TG	T
374	114138	A	AT
375	114141	AAAC	A
376	114142	AAC	A
377	114143	AC	A
378	114181	C	CAACTTT
379	114195	C	CTTTTGCCTA
380	114530	G	GA
381	114550	CTTCAATTA	C
382	114551	TTCAATTATTCAATTAGA	T
383	114560	TCAATTAG	T
384	114569	TA	T
385	114571	A	ATC
386	114598	TTCCTA	T
387	114709	T	TGAATA
388	114833	T	TATAGAAGAACGTATATAAAA
389	114835	T	TAGAAGAACGTATATAAAA
390	114906	CCTTTTTGTTTTCTCGAA	C
391	114995	GA	G
392	115086	C	CTTT
393	115109	AT	A
394	115156	T	TTTTTGAACTC
395	115179	GAGTATAGA	G
396	115250	C	CAGGTTAAAT
397	115259	T	TA
398	115396	T	TGCTTATTC
399	115566	A	ATTTTAGAATTATGTAATT
400	116544	C	CATTTAATTGA
401	116686	T	TTTA
402	116712	GA	G
403	118349	TC	T

404	118880	T	TCAATACAATATTAGAATCTAGATGATTAATATTAATTTCTCAATA
405	118881	C	CAATATTAGAATCTAGATGATT
406	120020	GA	G
407	120123	C	CTATCCATT
408	120196	C	CATTACTATTATAAT
409	120199	T	TACTATTATAA
410	121778	T	TAA
411	121785	A	ATGTGCTTTAT
412	121838	G	GA
413	121937	CAAAAAAAGAAAAAAG[illegible]	C
414	121955	AG	A
415	122050	GA	G
416	122295	CAAA	C
417	122460	A	AG
418	122476	CT	C
419	122555	GAAAAAT	G
420	122584	A	ATCTTT
421	122598	G	GTTTCTAGAA
422	124484	CAAATTAATCAAA	C
423	125986	TTTCAAG	T
424	126140	GTTTTTA	G
425	126662	T	TACTTGA
426	127205	C	CTTACTAATA
427	127718	T	TTTTGTTTCA
428	129200	T	TTCTAGCAAAGTATTGTTTATG
429	129950	GGAAGGA	G

Supplemental Table 5. Accessions examined in this study, their cladistic relationships, provenance, elevation above sea level, and latitude and longitude. All species identifications follow Spooner et al. (2014); when two names are present the former is the prior name (following Hawkes, 1990) and the latter the currently accepted name. We additionally examined Solanum lycopersicum with data deposited at (https://www.ncbi.nlm.nih.gov/; DQ347959.1).

Species	Subgroup	Country, City	Elevation	Latitude	Longitude
Solanum abancayense (S. candolleanum) 458404	Clade 4 north	Peru, Apurimac			
S. abancayense (S. candolleanum) 458403	Clade 4 north	Peru, Apurimac			
S. achacachense (S. candolleanum) 558032	Clade 4 north	Bolivia, La Paz			
S. acroglossum 365313	Clade 3	Peru, Huanuco	2200	-10	-76.5
S. acroglossum 498204	Clade 3	Peru, Pasco		-10.66666667	-76.08333333
S. acroscopicum 365314	Clade 3	Peru, Arequipa	3200	-15.2	-72.93333333
S. albornozii 498206	Clade 3	Ecuador, Loja		-4	-79.21666667
S. ambosinum (S. candolleanum) 498210	Clade 4 north	Peru, Ancash	3500	-9.26666667	-77.26666667
S. ambosinum (S. candolleanum) 498212	Clade 4 north	Peru, Ancash	3750	-10.65	-77.66666667
S. ambosinum (S. candolleanum) 498213	Clade 4 north	Peru, Ancash	3400	-8.91666667	-77.83333333
S. ambosinum (S. candolleanum) 365317	Clade 4 north	Peru, Pasco	3500	-10.5	-76.5
S. ambosinum (S. candolleanum) 365362	Clade 4 north	Peru, Ancash	3500	-8.26666667	-77.85
S. ambosinum (S. candolleanum) 498209	Clade 4 north	Peru, Ancash	3200	-9.26666667	-77.26666667
S. andreanum 561658	Clade 3	Ecuador, Bolívar	3000	-1.7	-79.08333333
S. andreanum 320345	Clade 3	Columbia, Cauca	2100	1.2	-76.91666667
S. andreanum 561648	Clade 3	Ecuador, Napo	2100	-0.53333333	-77.91666667
S. avilesii (S. brevicaule) 498091	Clade 4 south	Bolivia, Santa Cruz	2850	-18.63333333	-64.15
S. avilesii (S. brevicaule) 498092	Clade 4 south	Bolivia, Santa Cruz	2950	-18.63333333	-64.15
S. avilesii (S. brevicaule) 498093	Clade 4 south	Bolivia, Santa Cruz	2850	-18.63333333	-64.15
S. berthaultii 527886	Clade 4 south	Bolivia, Potosi	2880	-19.21666667	-65.16666667
S. berthaultii 498105	Clade 4 south	Bolivia, Cochabamba	2440	-17.95	-65.91666667
S. berthaultii 545850 (S. no 241 bp deletion)	Clade 4 south	Bolivia, Chuquisaca	2600	-19.26666667	-65.18333333
S. blanco-galdosii 498214	Clade 3	Peru, Ancash	3100	-8.48333333	-78.61666667
S. brevicaule 498111	Clade 4 south	Bolivia, Cochabamba	3730	-17.21666667	-66.05
S. brevicaule 498218	Clade 4 south	Bolivia, Cochabamba	3810	-15.78333333	-68.16666667
S. brevicaule 545968	Clade 4 south	Bolivia, Cochabamba	3960	-17.63333333	-66.65
S. brevicaule 545970	Clade 4 south	Bolivia, La Paz	3900	-15.6	-69.01666667
S. brevicaule 545971	Clade 4 south	Bolivia, Cochabamba	3840	-17.63333333	-66.71666667
S. brevicaule 545981	Clade 4 south	Bolivia, Chuquisaca	3200	-19	-65.33333333

S. brevicaule 310931	Clade 4 south	Bolivia, Chuquisaca		-19.03333333	-65.28333333
S. brevicaule 473378	Clade 4 south	Bolivia, Cochabamba	3150	-17.33333333	-66.35
S. bukasovii (S. candolleanum) 414155	Clade 4 north	Peru, Apurimac	3900	-13.65	-73.38333333
S. bukasovii (S. candolleanum) 473492	Clade 4 north	Peru, Huancavelica	3800		
S. bukasovii (S. candolleanum) 473493	Clade 4 north	Peru, Huancavelica	3650	-12.83333333	-74.53333333
S. bukasovii (S. candolleanum) 473494	Clade 4 north	Peru, Huancavelica			
S. bukasovii (S. candolleanum) 568933	Clade 4 north	Peru, Puno			
S. bukasovii (S. candolleanum) 568954	Clade 4 north	Peru, Puno			
S. bukasovii (S. candolleanum) 266385	Clade 4 north	Peru, Junin	3840	-11.6	-75.36666667
S. bukasovii (S. candolleanum) 365353	Clade 4 north	Peru, Cuzco	3550	-13.33333333	-72.11666667
S. bulbocastanum 545751	Clade 1+2	Mexico, Jalisco	1990	20.98333333	-103.1666667
S. bulbocastanum 604074	Clade 1+2	Guatemala, Huehuetenango	2000	15.32166667	-91.54833333
S. cajamarquense 230522	Clade 4 south	Peru, Cajamarca	2600	-6.81666667	-79.2
S. canasense (S. candolleanum) 265864	Clade 4 north	Peru, Cuzco	3800	-13.51666667	-71.98333333
S. canasense (S. candolleanum) 283084	Clade 4 north	Peru, Puno	4000	-15.83333333	-70.03333333
S. canasense (S. candolleanum) 442696	Clade 4 north	Peru, Puno	3900	-15.83333333	-70.03333333
S. canasense (S. candolleanum) 498226	Clade 4 north	Bolivia, La Paz	3600	-15.4	-69.06666667
S. canasense (S. candolleanum) 498227	Clade 4 north	Bolivia, La Paz	4000	-15.78333333	-68.66666667
S. canasense (S. candolleanum) 545972	Clade 4 north	Bolivia, La Paz	3900	-15.6	-69.01666667
S. canasense (S. candolleanum) 568969	Clade 4 north	Bolivia, La Paz	3600		
S. canasense (S. candolleanum) 265865	Clade 4 south	Bolivia, La Paz	3800	-17.36694444	-67.15
S. canasense (S. candolleanum) 210035	Clade 4 north	Peru, Cuzco	3350	-13.43333333	-71.85
S. canasense (S. candolleanum) 473355	Clade 4 north	Peru, Ayacucho	3800	-13.4	-73.9
S. canasense (S. candolleanum) 246533	Clade 4 north	Peru, Cuzco	3600	-13.46666667	-71.91666667
S. cardiophyllum 283062	Clade 1+2	Mexico			
S. cardiophyllum 347759	Clade 1+2	Mexico,Puebla	2060	18.61666667	-97.58333333
S. cardiophyllum 283063	Clade 1+2	Mexico			
S. chacoense 320294	Clade 4 south	Argentina, Buenos Aires	20	-34.66666667	-58.66666667
S. chacoense 472816	Clade 4 south	Argentina, Salta	2500	-25.18333333	-65.78333333
S. chacoense 472830	Clade 4 south	Argentina, La Rioja	1140	-29.23333333	-66.85
S. chacoense 500020	Clade 4 south	Argentina, Jujuy	2000	-23.96666667	-65.45
S. chacoense 275138	Clade 4 south	Argentina, Túcuman	3000	-27.25	-65.86666667

S. chomatophilum 365339	Clade 3	Peru, Junín	3550	-10.88333333	-75.95
S. chomatophilum 365328	Clade 3	Peru Amazonas-		-5.83333333	-77.91666667
S. goniocalyx (S. tuberosum) 195186	cultivated	Peru, Ayacucho		-13.2	-74.25
S. goniocalyx (S. tuberosum) 195214	cultivated	Peru, Huanuco			
S. goniocalyx (S. tuberosum) 458393	cultivated	Bolivia, La Paz	3950	-17.03333333	-68.85
S. goniocalyx (S. tuberosum) 195188	cultivated	Peru, Ayacucho			
S. gourlayi (S. brevicaule) 472995	Clade 4 south	Argentina, Salta	3000	-22.71666667	-65.2
S. gourlayi (S. brevicaule) 473019	Clade 4 south	Argentina, Jujuy	3000	-23.65	-65.6
S. gourlayi (S. brevicaule) 473077	Clade 4 south	Argentina, Salta	3700	-24.53333333	-66.2
S. gourlayi (S. brevicaule) 473106	Clade 4 south	Argentina, Salta	3500	-22.18333333	-65.18333333
S. gourlayi (S. brevicaule) 500022	Clade 4 south	Argentina, Jujuy	3000	-23.65	-65.6
S. gourlayi (S. brevicaule) 537026	Clade 4 south	Bolivia, Potosi	3280	-19.6	-65.26666667
S. gourlayi (S. brevicaule) 545865	Clade 4 south	Bolivia, Potosi	3460	-19.56694444	-65.38277778
S. gourlayi (S. brevicaule) 545975	Clade 4 south	Bolivia, Chuquisaca	3200	-18.93333333	-65.38333333
S. gourlayi (S. brevicaule) 545978	Clade 4 south	Bolivia, Chuquisaca	3000	-18.95	-65.31666667
S. gourlayi (S. brevicaule) 558067	Clade 4 south	Argentina, Jujuy	3450	-24.1	-65.66666667
S. gourlayi (S. brevicaule) 472911	Clade 4 south	Argentina, Salta	3400	-22.2	-65.16666667
S. gourlayi (S. brevicaule) 472991	Clade 4 south	Argentina, Jujuy	3300	-23.2	-65.45
S. hondelmannii (S. brevicaule) 498071	Clade 4 south	Bolivia, Potosi	2350	-19.33333333	-65.16666667
S. hondelmannii (S. brevicaule) 473365	Clade 4 south	Bolivia, Chuquisaca	2575	-19.16666667	-65.28333333
S. hondelmannii (S. brevicaule) 545879	Clade 4 south	Bolivia, Cochabamba	2260	-17.98333333	-65.11666667
S. hondelmannii (S. brevicaule) 498067	Clade 4 south	Bolivia, Chuquisaca	2900	-19.1	-65.23333333
S. hypacrarthrum 473477	Clade 3	Peru, Lima		-11.41666667	-76.63333333
S. incamayoense (S. brevicaule) 473070	Clade 4 south	Argentina, Salta	2700	-24.51666667	-65.85
S. incamayoense (S. brevicaule) 500048	Clade 4 south	Argentina, Salta	2000	-24.85	-65.71666667
S. incamayoense (S. brevicaule) 473060	Clade 4 south	Argentina, Salta	1900	-24.81666667	-65.7
S. incamayoense (S. brevicaule) 473067	Clade 4 south	Argentina, Salta	2280	-24.75	-65.73333333
S. incamayoense (S. brevicaule) 473069	Clade 4 south	Argentina, Salta	2500	-24.66666667	-65.76666667
S. jamesii 641944	Clade 1+2	USA, Utah	1734	37.77666667	-111.5766667
S. jamesii 664024	Clade 1+2	USA, Texas	1807	30.69633138	-104.1165507
S. kurtzianum 472948	Clade 4 south	Argentina, La Rioja	1400	-29.38333333	-68.01666667
S. kurtzianum 472952	Clade 4 south	Argentina, Catamarca	1840	-27.9	-66.36666667
S. kurtzianum 558185	Clade 4 south	Argentina, Mendoza	1180	-32.6	-68.86666667
S. kurtzianum 558208	Clade 4 south	Argentina, La Rioja	1790	-29.16666667	-67.65

S. venturii 320327	Clade 4 south	Argentina, Salta	2700	-25.15	-65.86666667
S. kurtzianum 472936	Clade 4 south	Argentina, Catamarca	1840	-27.46666667	-66.43333333
S. kurtzianum 472924	Clade 4 south	Argentina, La Rioja	1100	-33.98333333	-69.23333333
S. laxissimum 498252	Clade 4 north	Bolivia			
S. laxissimum 607887	Clade 4 north	Peru, Cuzco	2650	-13.17944444	-72.54166667
S. laxissimum 283088	Clade 4 north	Peru, Cuzco	2500		
S. leptophyes (S. brevicaule) 473446	Clade 4 north	Peru,Cuzco		-13.56666667	-72.75
S. leptophyes (S. brevicaule) 473451	Clade 4 north	Peru, Ayacucho		-15.55	-73.63333333
S. leptophyes (S. brevicaule) 545985	Clade 4 south	Bolivia, Oruro	3860	-17.96666667	-66.93333333
S. leptophyes (S. brevicaule) 545987	Clade 4 south	Bolivia, Potosi	3680	-18.01666667	-66.36666667
S. leptophyes (S. brevicaule) 458378	Clade 4 south	Peru, Puno	3900	-16.369547	-69.221425
S. leptophyes (S. brevicaule) 473342	Clade 4 south	Bolivia, La Paz	3300	-16.55	-68.1
S. limbaniense 473468	Clade 4 north	Peru			
S. marinasense 210040	Clade 4 north	Peru, Cuzco	3350	-13.43333333	-71.85
S. marinasense 310944	Clade 4 north	Peru, Puno		-13.16666667	-71.41666667
S. marinasense 498255	Clade 4 north	Peru, Piura			
S. medians 320260	Clade 4 north	Peru, Lima	2850	-11.41666667	-76.63333333
S. medians 473496	Clade 4 north	Peru, Lima	2850	-11.56666667	-76.61666667
S. medians 210045	Clade 4 north	Peru, Lima	2200	-11.41666667	-76.63333333
S. medians 458402	Clade 4 north	Peru, Lima	2900	-11.36666667	-77.01666667
S. medians 230507	Clade 4 north	Peru, Junín		-11.5	-75.83333333
S. megistacrolobum (S. boliviense) 500029	Clade 4 south	Argentina, Salta	3450	-25.16666667	-65.86666667
S. megistacrolobum (S. boliviense) 546000	Clade 4 south	Bolivia, Chuquisaca	3350	-19.45	-64.73333333
S. megistacrolobum (S. boliviense) 210034	Clade 4 south	Bolivia, Potosi	3500	-19.91666667	-65.66666667
S. megistacrolobum (S. boliviense) 473158	Clade 4 south	Argentina, Salta	3400	-22.26666667	-65.18333333
S. microdontum 218225	Clade 4 south	Bolivia, Potosi			
S. microdontum 545884	Clade 4 south	Bolivia, Cochabamba	2740	-17.66666667	-65.3
S. multidissectum (S. candolleanum) 275272	Clade 4 north	Peru, Cuzco	2500	-13.33333333	-71.96666667
S. multidissectum (S. candolleanum) 473349	Clade 4 north	Peru, Cuzco	3300	-13.43333333	-71.85
S. multidissectum (S. candolleanum) 473352	Clade 4 north	Peru, Puno	4175	-14.75	-70.75
S. multidissectum (S. candolleanum) 210052	Clade 4 north	Peru, Ayacucho	3900	-13.26666667	-73.85
S. multidissectum (S. candolleanum) 210055	Clade 4 north	Peru, Cuzco	3950	-14.16666667	-71.1
S. multiinterruptum 365337	Clade 3	Peru, Lima	3175	-12.4	-75.95
S. multiinterruptum 365338	Clade 3	Peru, Ancash	3800	-9.71666667	-77.46666667

S. multiinterruptum 498266	Clade 3	Peru, Ancash	3500	-8.63333333	-77.6
S. multiinterruptum 210044	Clade 4 north	Peru, Junín	4000	-11.75	-75.46666667
S. multiinterruptum 365336	Clade 3	Peru, Ancash	3550	-9.86666667	-77.73333333
S. pampasense (S. candolleanum) 458381	Clade 4 north	Peru, Ayacucho	2400	-13.38333333	-73.25
S. pampasense (S. candolleanum) 275274	Clade 4 north	Peru, Ayacucho	2300	-13.45	-73.73333333
S. pampasense (S. candolleanum) 442697	Clade 4 north	Peru, Apurimac	2600	-13.11666667	-74.21666667
S. pampasense (S. candolleanum) 275275	Clade 4 north	Peru, Ayacucho	2600	-13.41666667	-73.9
S. paucissectum 473489	Clade 3	Peru, Piura	3050	-5.33333333	-79.43333333
S. phureja (S. tuberosum) 225665	cultivated	Colombia, Cauca	2950	2.61666667	-76.28333333
S. phureja (S. tuberosum) 225693	cultivated	Colombia, Cundinamarca		5.15	-73.68333333
S. phureja (S. tuberosum) 225703	cultivated	Colombia, Boyaca		5.51666667	-73.36666667
S. phureja (S. tuberosum) 243467	cultivated	Colombia, Valle		3.53333333	-76.26666667
S. phureja (S. tuberosum) 243468	cultivated	Colombia, Narino	3000	0.88333333	-77.63333333
S. phureja (S. tuberosum) 243469	cultivated	Peru, Apurimac			
S. phureja (S. tuberosum) 258855	cultivated	Bolivia, Santa Cruz		-18.33333333	-64.16666667
S. phureja (S. tuberosum) 195191	cultivated	Ecuador, Pichincha		0.06666667	-78.4
S. phureja (S. tuberosum) 195198	cultivated	Colombia, Narino		1.21666667	-77.28333333
S. phureja (S. tuberosum) 195198 (GenBank reference)	cultivated	Colombia, Narino			
S. pinnatisectum 253214	Clade 1+2	Mexico, Queretaro	1000	20.5	-100.1333333
S. pinnatisectum 537023	Clade 1+2	Mexico, Guanajuato	1900	20.8	-101.1833333
S. polyadenium 161728	Clade 1+2	Mexico, Michoacan		19.73333333	-101.6166667
S. polyadenium 347770	Clade 1+2	Mexico,Veracruz	2340	18.71666667	-97.31666667
S. sogarandinum 230510	Clade 3	Peru, La Libertad	3600	-8.15	-78.18333333
S. sogarandinum 365360	Clade 3	Peru, Ancash	3500		
S. sparsipilum (S. brevicaule) 473385	Clade 4 south	Peru, Cuzco	2850	-13.36666667	-71.98333333
S. sparsipilum (S. brevicaule) 498134	Clade 4 south	Bolivia, Cochabamba	2560	-17.95	-65.91666667
S. sparsipilum (S. brevicaule) 498284	Clade 4 south	Bolivia, La Paz	3850	-16.16666667	-69.08333333
S. sparsipilum (S. brevicaule) 498285	Clade 4 south	Bolivia, Potosi	3030	-19.58333333	-65.28333333
S. sparsipilum (S. brevicaule) 246536	Clade 4 south	Peru, Cuzco	2900	-13.3	-72.11666667
S. sparsipilum (S. brevicaule) 473375	Clade 4 south	Bolivia, Cochabamba	2400	-17.56666667	-66.35
S. spegazzinii (S. brevicaule) 472966	Clade 4 south	Argentina, La Rioja	2850	-28.58333333	-68.15
S. spegazzinii (S. brevicaule) 472988	Clade 4 south	Argentina, Tucuman	2700	-26.66666667	-65.81666667

S. spegazzinii (S. brevicaule) 472990	Clade 4 south	Argentina, La Rioja	2750	-29	-67.85
S. spegazzinii (S. brevicaule) 320299	Clade 4 south	Argentina, Catamarca	2800	-27.23333333	-66.25
S. spegazzinii (S. brevicaule) 458335		Argentina, Salta	3200	-25.41666667	-65.93333333
S. spegazzinii (S. brevicaule) 458337	Clade 4 south	Argentina, La Rioja	2400	-28.66666667	-67.75
S. stenophyllidium 558460	Clade 1+2	Mexico, Jalisco	1400	21	-103.4166667
S. stenophyllidium 255527	Clade 1+2	Mexico, Aguascalientes		21.86666667	-102.5
S. stenophyllidium 320265	Clade 1+2	Mexico, Chihuahua	2150	29.13333333	-106.0833333
S. stenotomum (S. tuberosum) 230512	cultivated	Peru, Amazonas		-6.21666667	-77.85
S. stenotomum (S. tuberosum) 234011	cultivated	Bolivia			
S. stenotomum (S. tuberosum) 283141	cultivated	Colombia			
S. stenotomum (S. tuberosum) 365344	cultivated	Peru, Junin		-12.06666667	-75.23333333
S. stenotomum (S. tuberosum) 195204	cultivated	Peru, Cuzco		-13.16666667	-71.41666667
S. stenotomum (S. tuberosum) 205527	cultivated	Peru, Huanuco		-9.83333333	-76.33333333
S. stenotomum (S. tuberosum) 230513	cultivated	Peru, Amazonas	2800	-6.21666667	-77.96666667
S. stenotomum (S. tuberosum) 320364	cultivated	Colombia, Norte de Santander		7.9	-72.51666667
S. tarijense (S. berthaultii) 217457	Clade 4 south	Argentina, Salta	2500	-22.21666667	-64.88333333
S. tarijense (S. berthaultii) 414152	Clade 4 south	Bolivia, Tarija	2350	-21.51694444	-64.55
S. tarijense (S. berthaultii) 473217	Clade 4 south	Argentina, Salta	2550	-22.6	-65.11666667
S. tarijense (S. berthaultii) 473218	Clade 4 south	Argentina, Salta	2200	-22.25	-64.96666667
S. tarijense (S. berthaultii) 458366 (S. no 241 bp deletion)	Clade 4 south	Argentina, Salta	2100	-22.25	-64.95
S. vernei 458370	Clade 4 south	Argentina, Salta	3600	-23.16666667	-64.95
S. vernei 558150	Clade 4 south	Argentina, Jujuy	3350	-23.6	-65.18333333
S. vernei 320332	Clade 4 south	Argentina, Catamarca	2450	-27.35	-66.03333333
S. vernei 473303	Clade 4 south	Argentina, Jujuy	3200	-23.6	-65.13333333
S. vernei 473309	Clade 4 south	Argentina, Salta	3140	-23.11666667	-64.53333333
S. vernei 500070	Clade 4 south	Argentina, Salta	3100	-23.2	-64.91666667
S. vernei 320333	Clade 4 south	Argentina, Jujuy	2960	-23.91666667	-65.41666667
S. verrucosum 195170	Clade 4 south	Mexico, Tlaxcala		19.23333333	-98.03333333
S. verrucosum 275260	Clade 4 south	Mexico, Hidalgo	2700	20.11666667	-98.73333333
S. verrucosum 498061	Clade 4 south	Mexico, Coahuila	2720	25.2	-100.0666667

S. verrucosum 545745	Clade 4 south	Mexico, Nuevo León	2600	23.96666667	-99.76666667
S. verrucosum 545747	Clade 4 south	Mexico, Mexico	3200	19.71666667	-99.78333333
S. verrucosum 558463	Clade 4 south	Mexico, Mexico	3630	19.13333333	-99.78333333
S. verrucosum 275256	Clade 4 south	Mexico, Michoacán	2950	19.4	-101.6
S. verrucosum 498010	Clade 4 south	Mexico, Hidalgo	2960	20.03277778	-98.68277778
S. verrucosum 558488	Clade 4 south	Mexico, Jalisco	3410	19.56666667	-103.5833333
S. violaceimarmoratum 473398	Clade 4 north	Bolivia, La Paz	3225	-16.31666667	-67.9
S. violaceimarmoratum 498296	Clade 4 north	Bolivia, La Paz	3500		
S. violaceimarmoratum 473396	Clade 4 north	Bolivia, Cochabamba	3150		
S. etuberosum 498311	Outgroup	Chile, Bio-Bio	615	-36.41666667	-70.96666667
S. palustre 245763	Outgroup	Chile, La Araucania	800	-38.7	-71.88333333
S. lycopersicum 347959 (GenBank reference)	Outgroup		475	47.29861	8.12028

Suppl. Figs. 1. Coverage and CG plots of the accessions examined here.

Solanum abancayense PI 458403

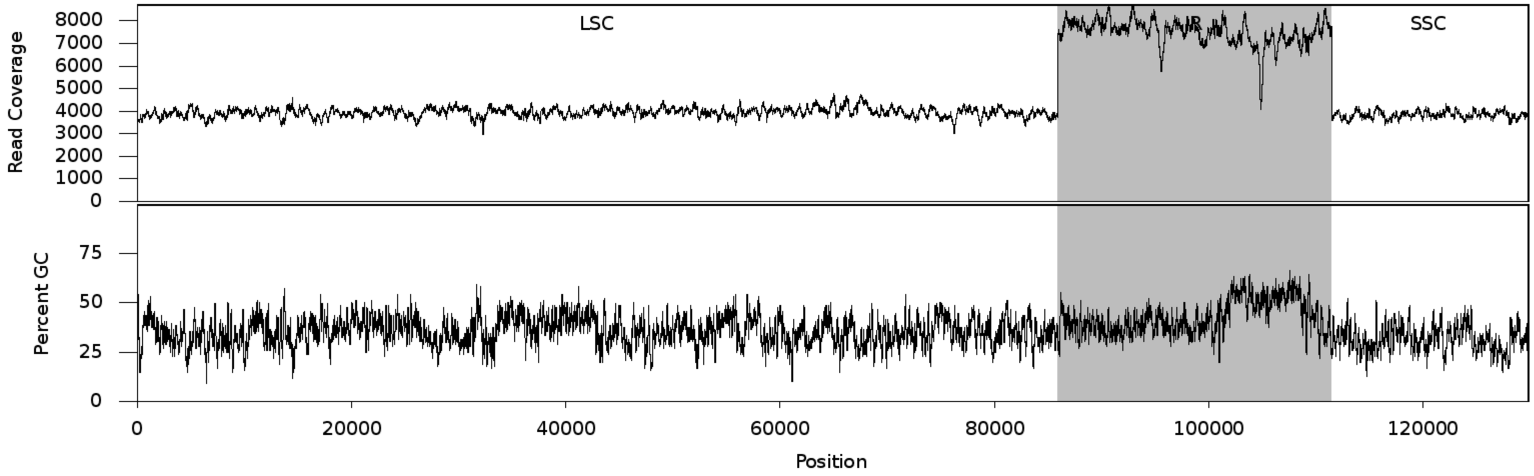

Solanum abancayense PI 458404

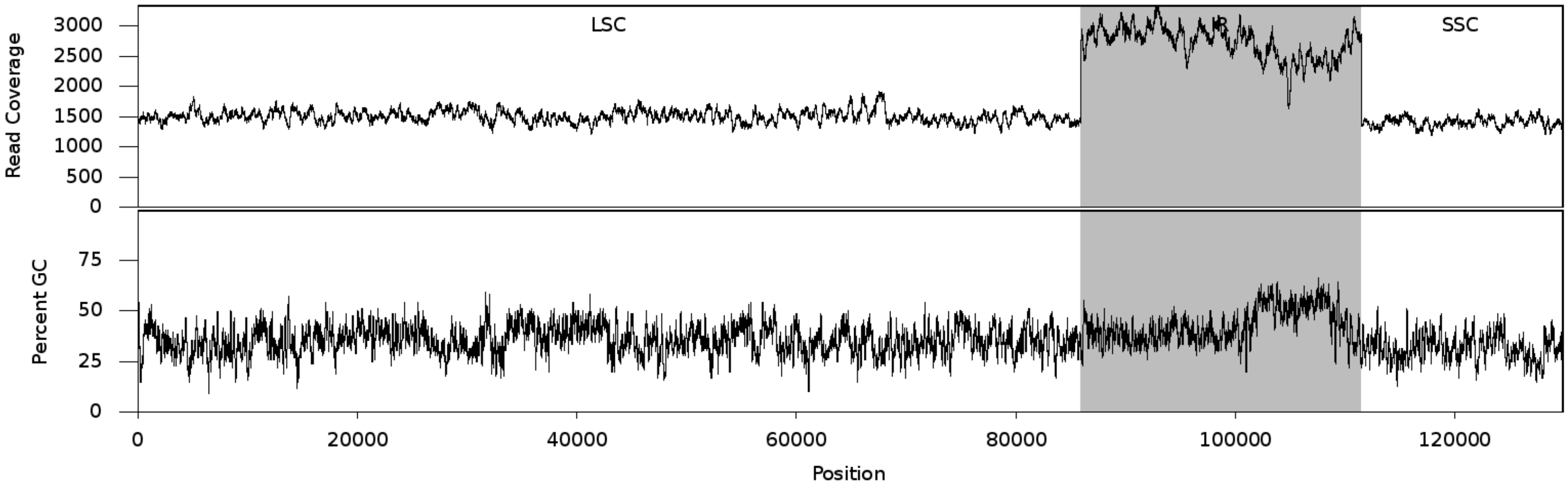

Solanum achacachense PI 558032

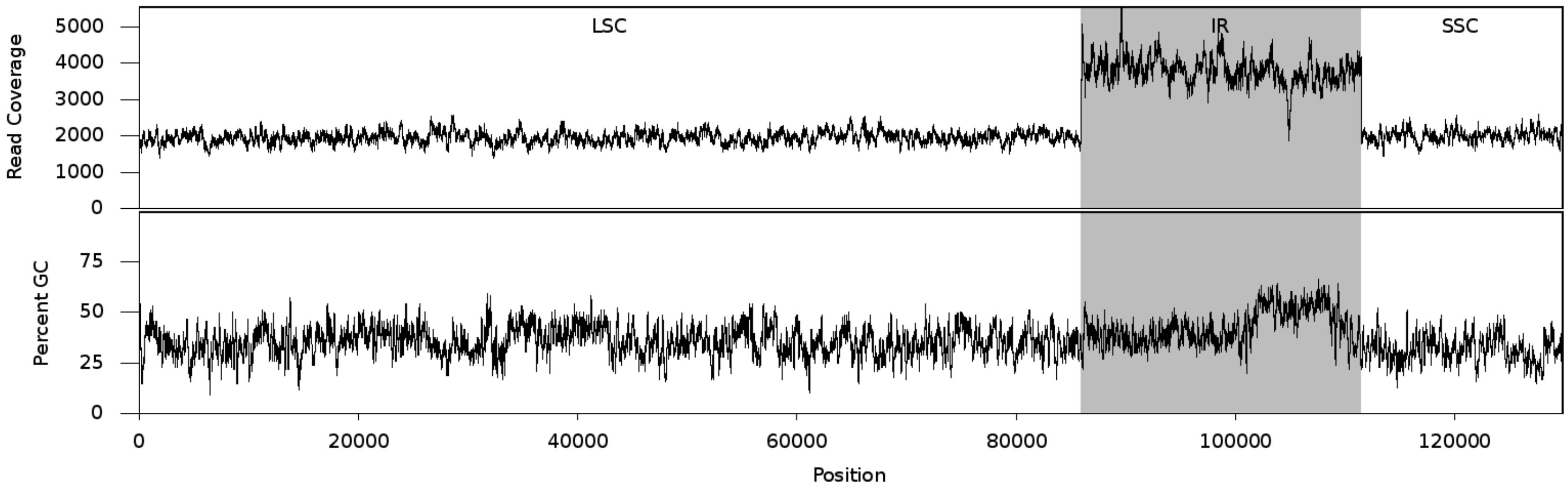

Solanum acroglossum PI 365313

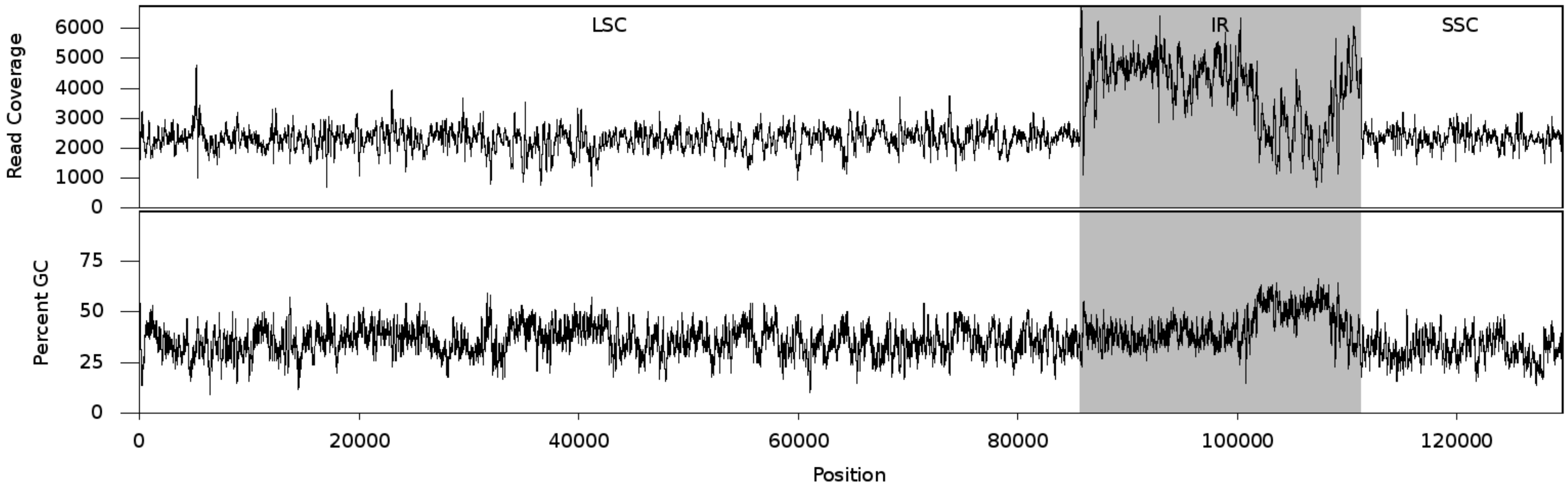

Solanum acroglossum PI 498204

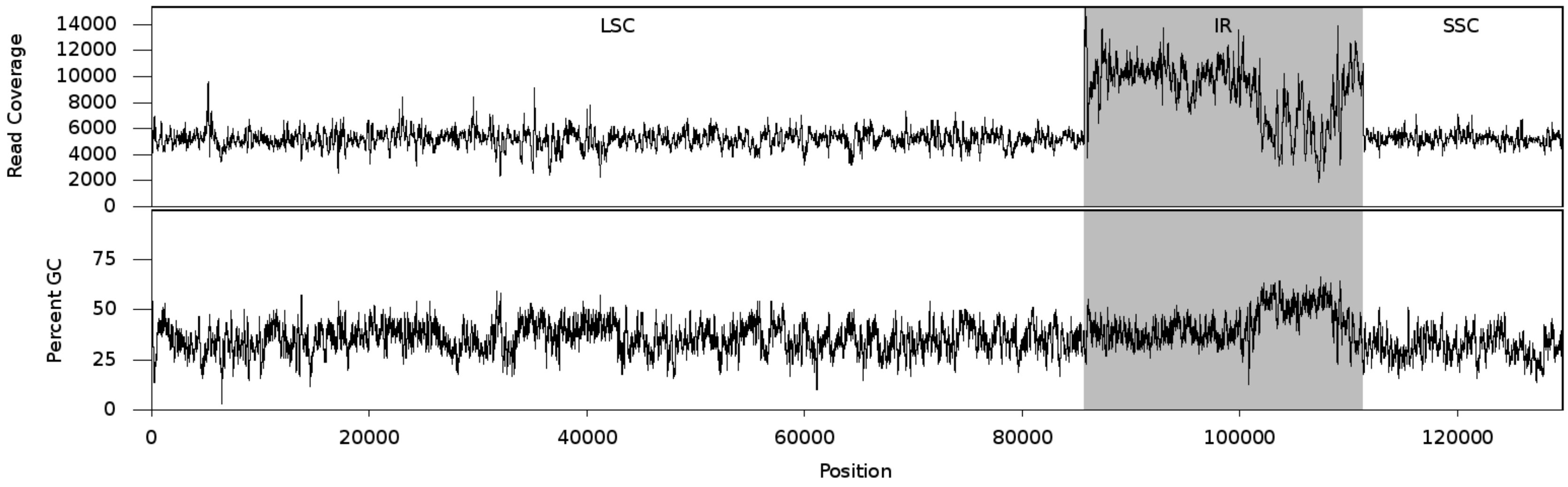

Solanum acroscopicum PI 365314

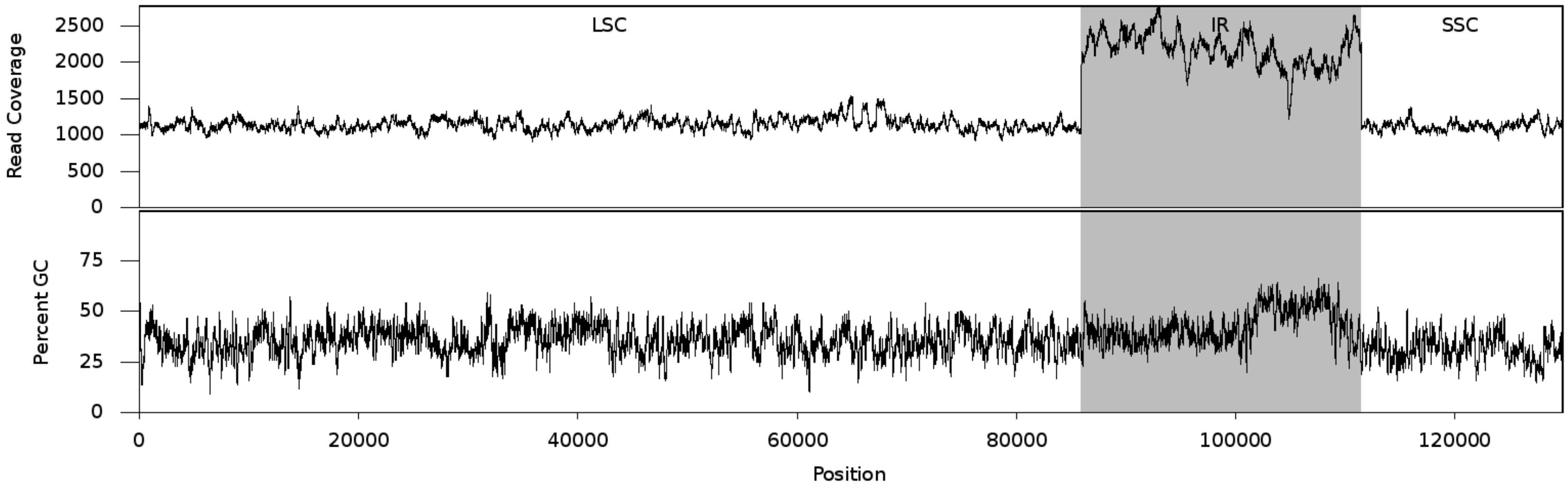

Solanum albornozii PI 498206

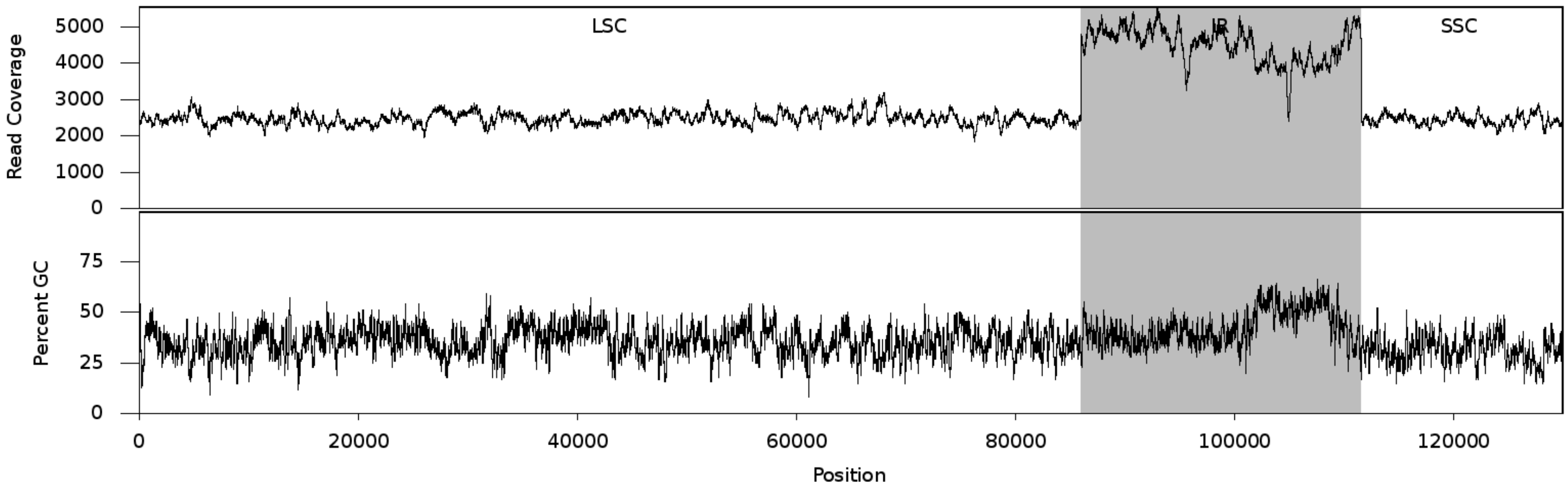

Solanum ambosinum PI 365317

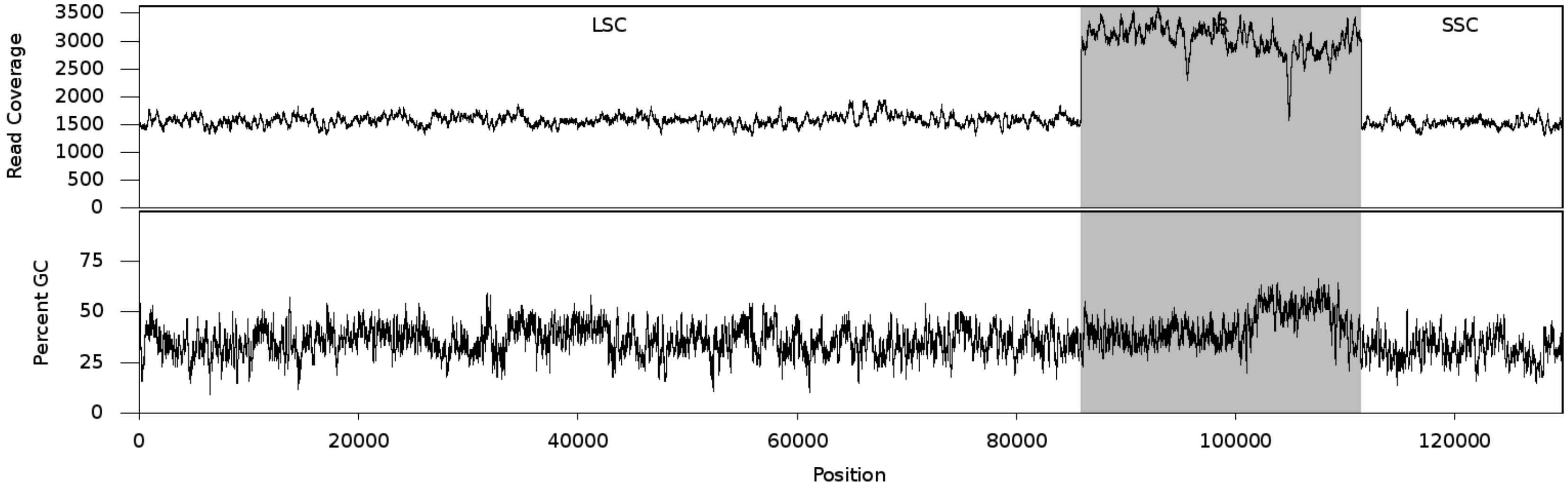

Solanum ambosinum PI 365362

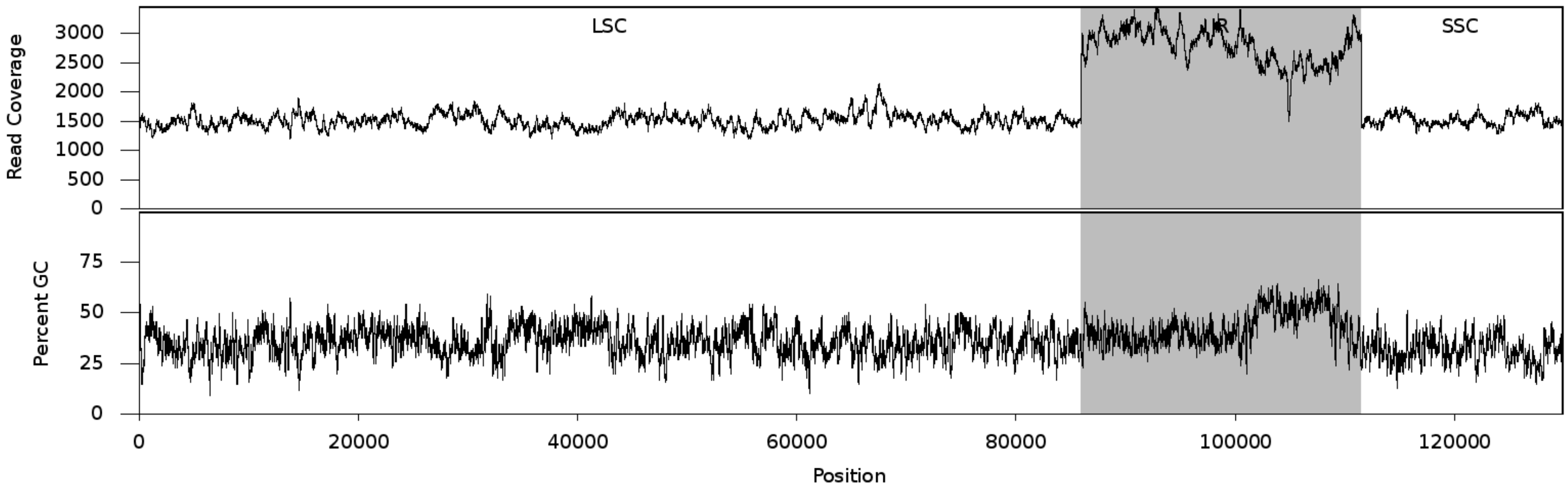

Solanum ambosinum PI 498209

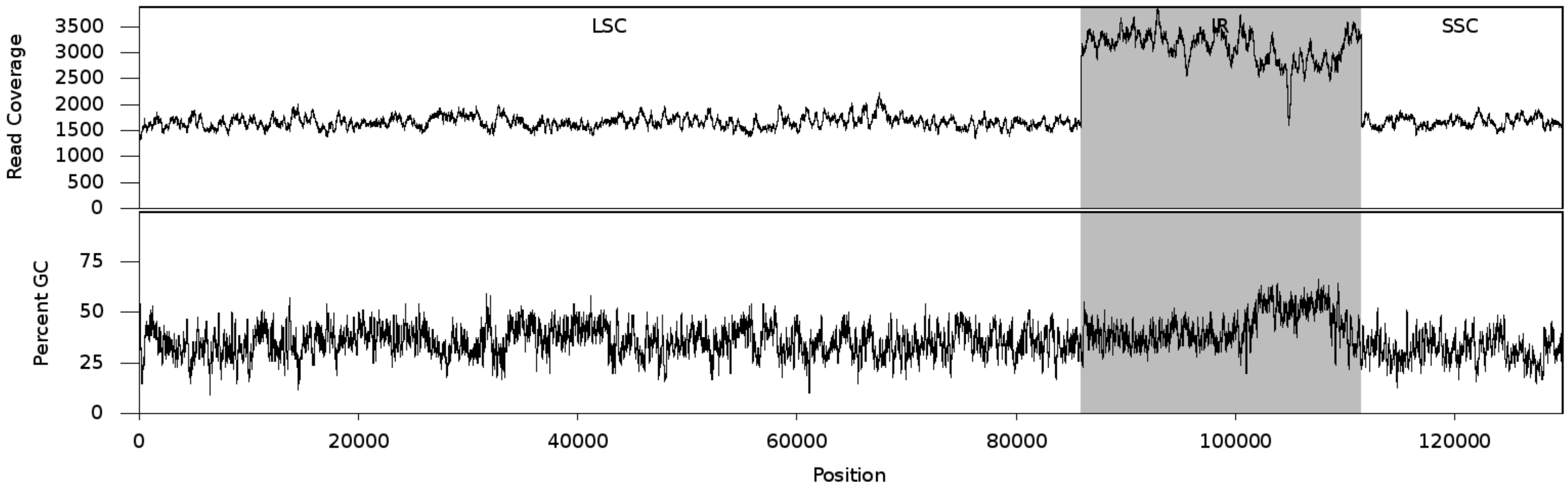

Solanum ambosinum PI 498210

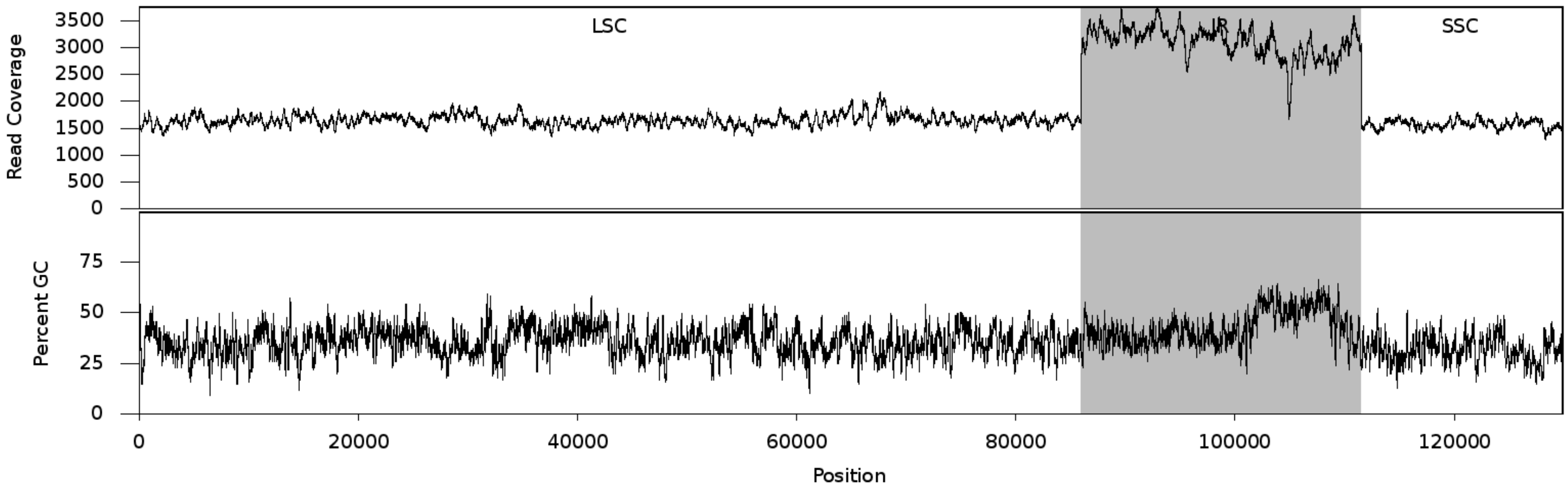

Solanum ambosinum PI 498212

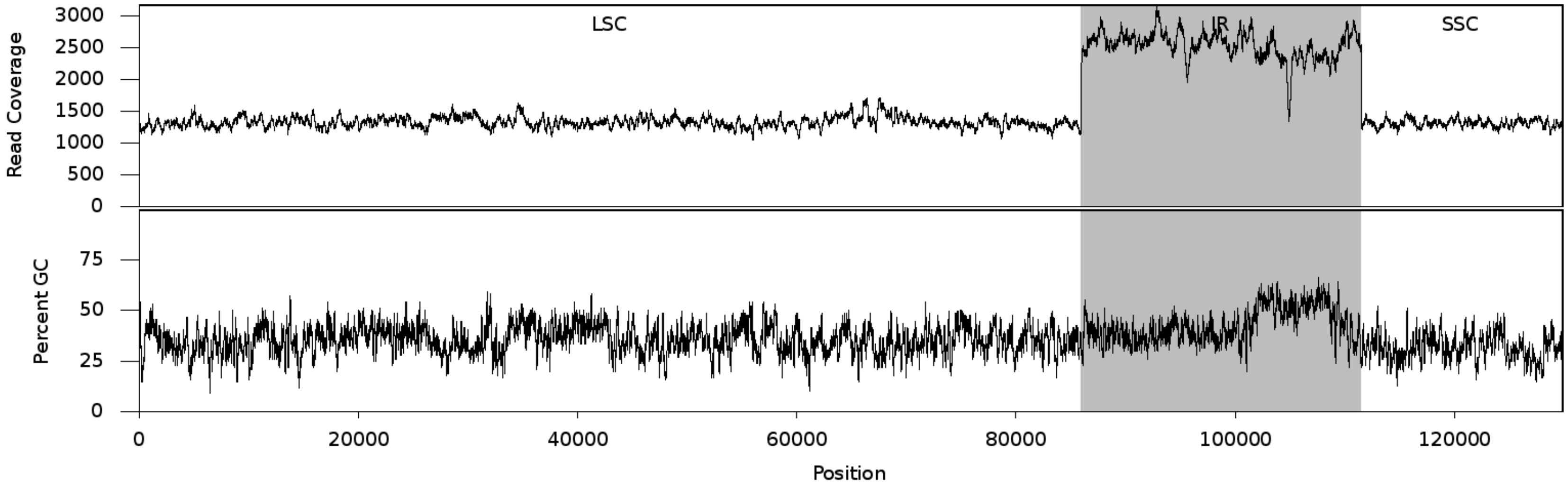

Solanum ambosinum PI 498213

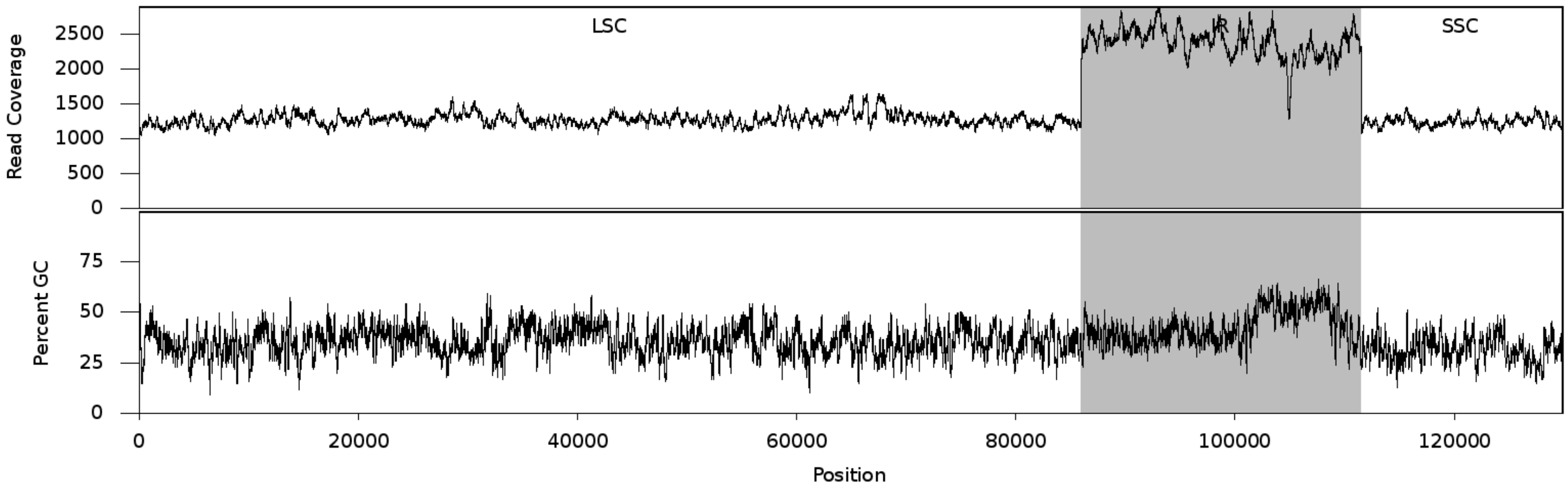

Solanum andreanum PI 320345

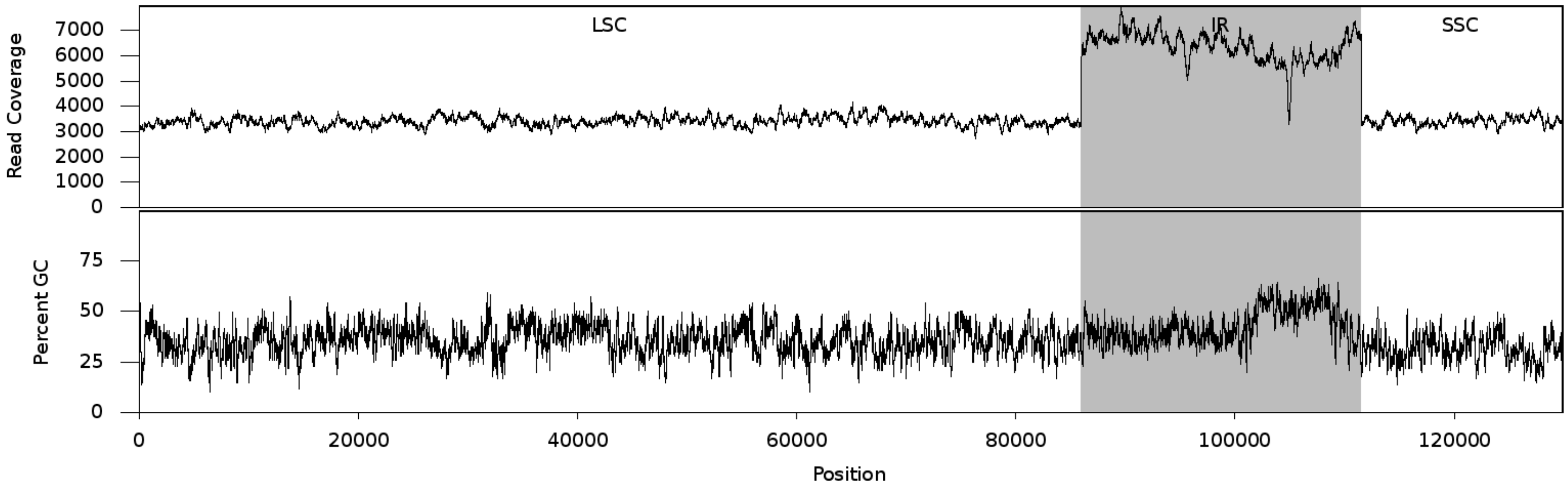

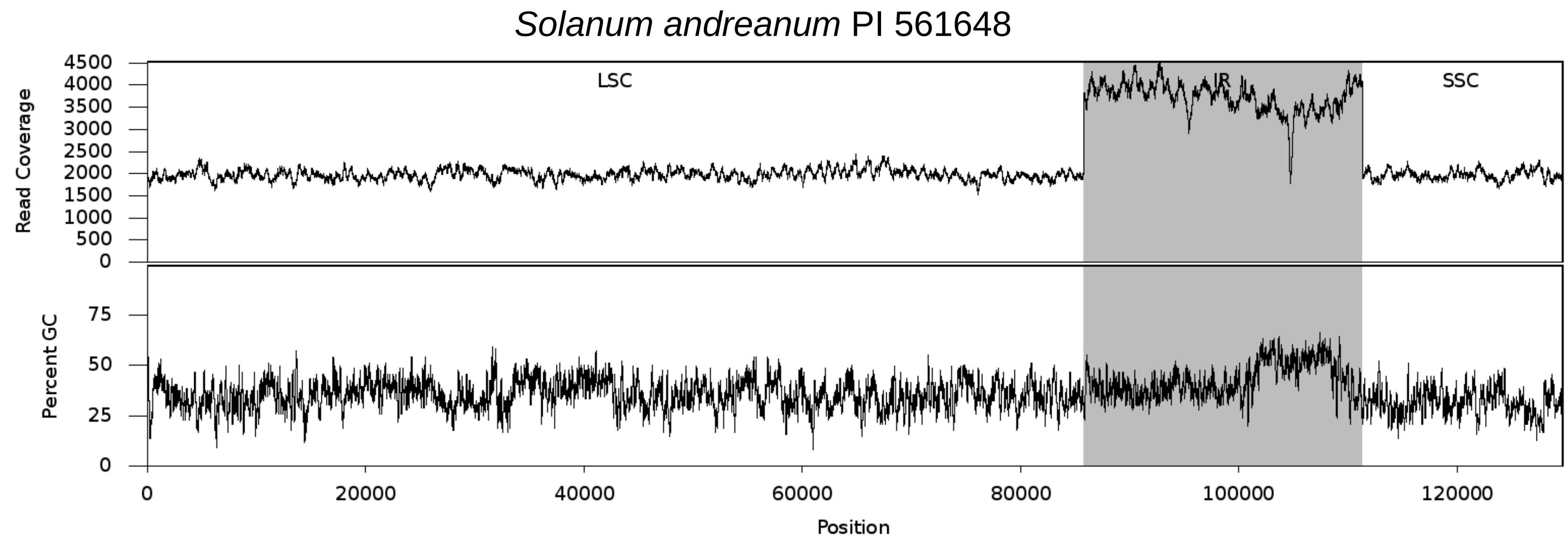
Solanum andreanum PI 561648
LSC
IR
SSC
Read Coverage
4500
4000
3500
3000
2500
2000
1500
1000
500
0
Percent GC
75
50
25
0
0
20000
40000
60000
80000
100000
120000
Position

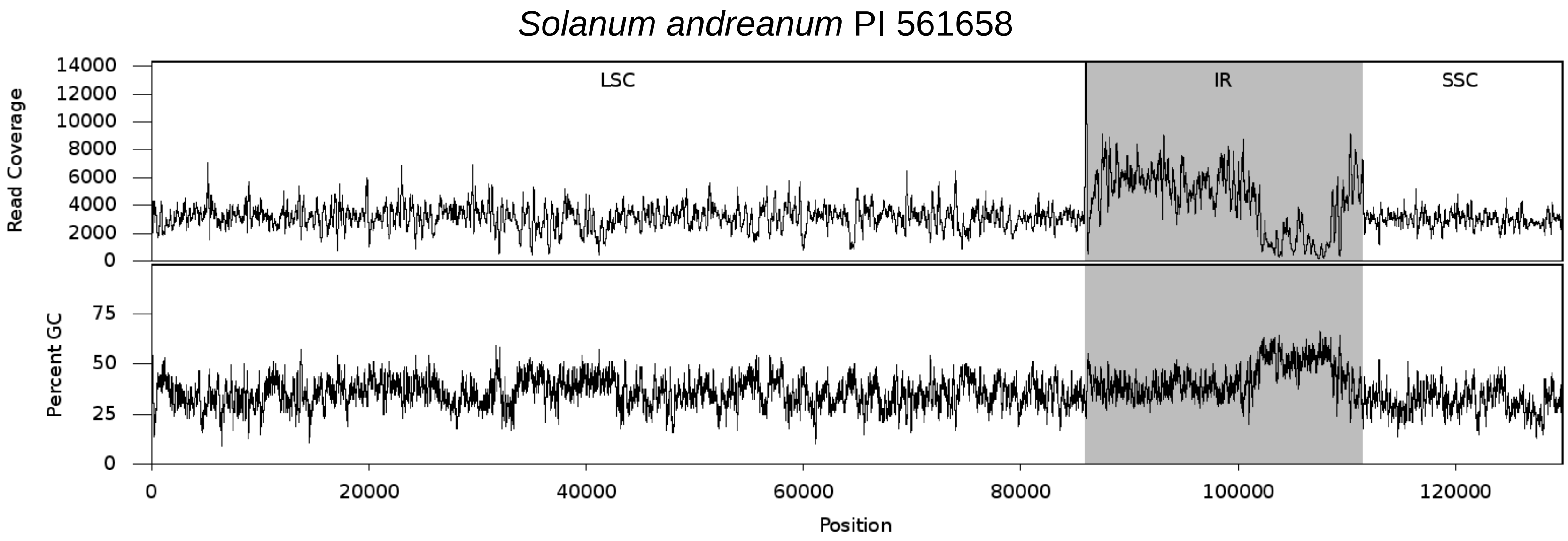
Solanum andreanum PI 561658
LSC
IR
SSC
Read Coverage
14000
12000
10000
8000
6000
4000
2000
0
Percent GC
75
50
25
0
0
20000
40000
60000
80000
100000
120000
Position

Solanum avilesii PI 498091

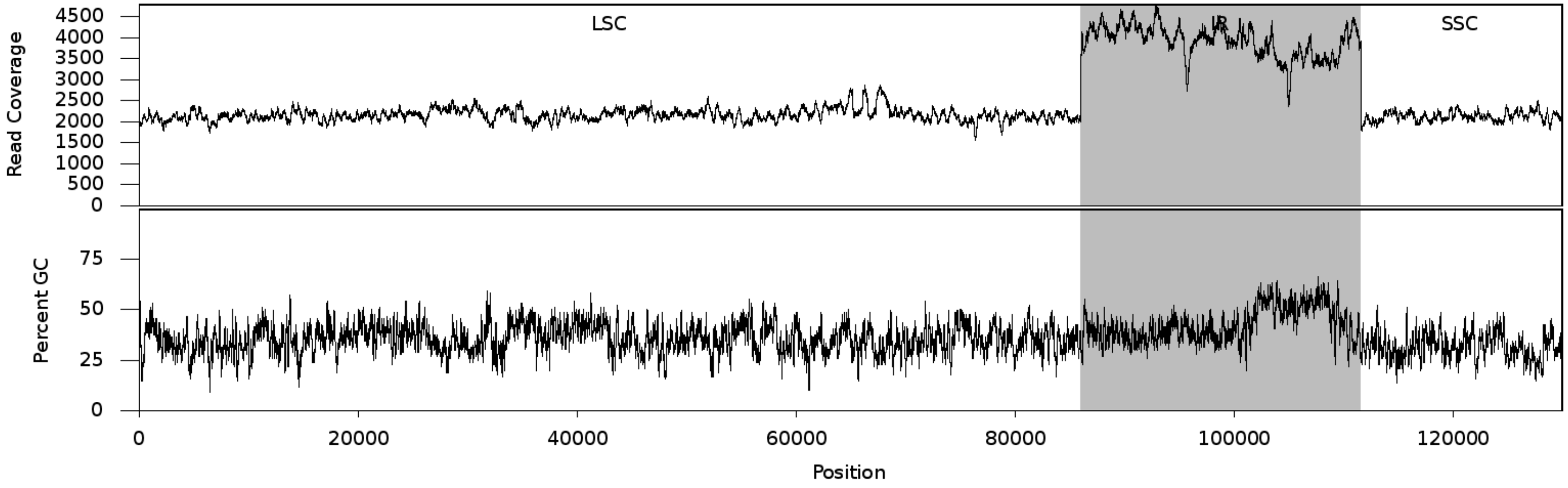

Solanum avilesii PI 498092

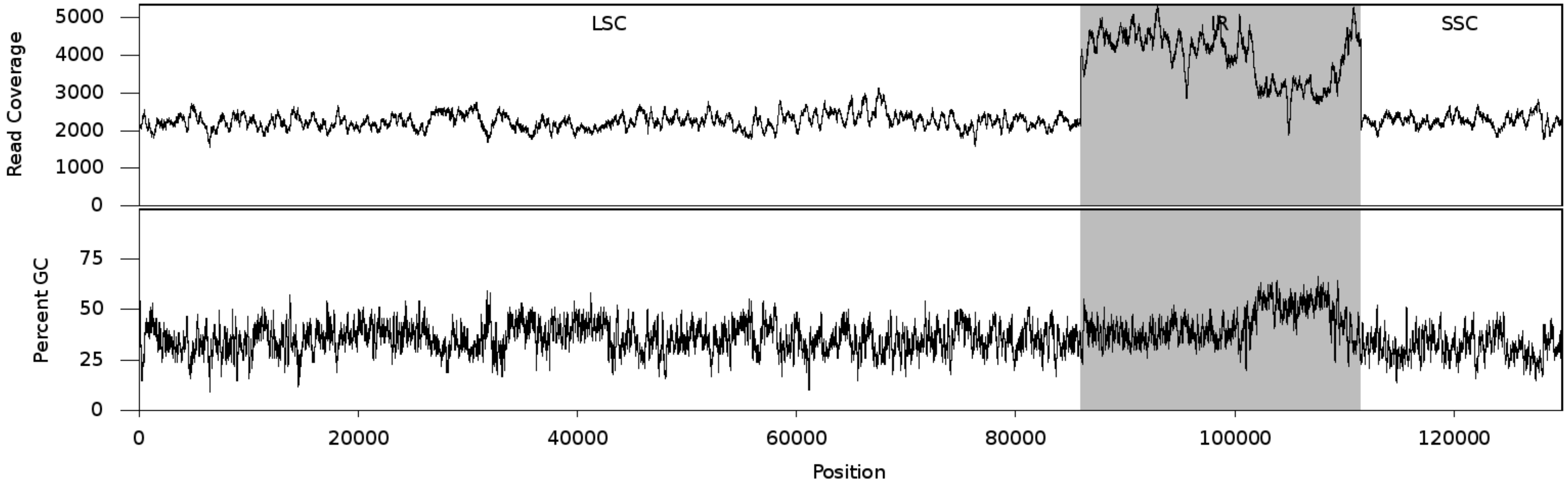

Solanum avilesii PI 498093

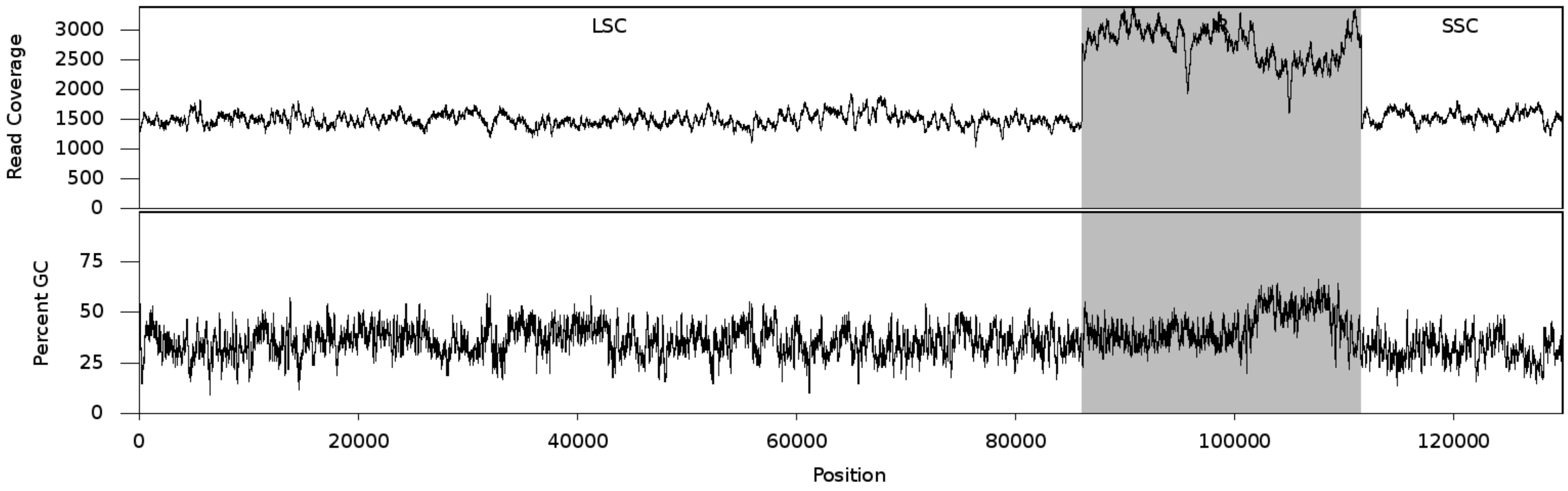

Solanum berthaultii PI 498105

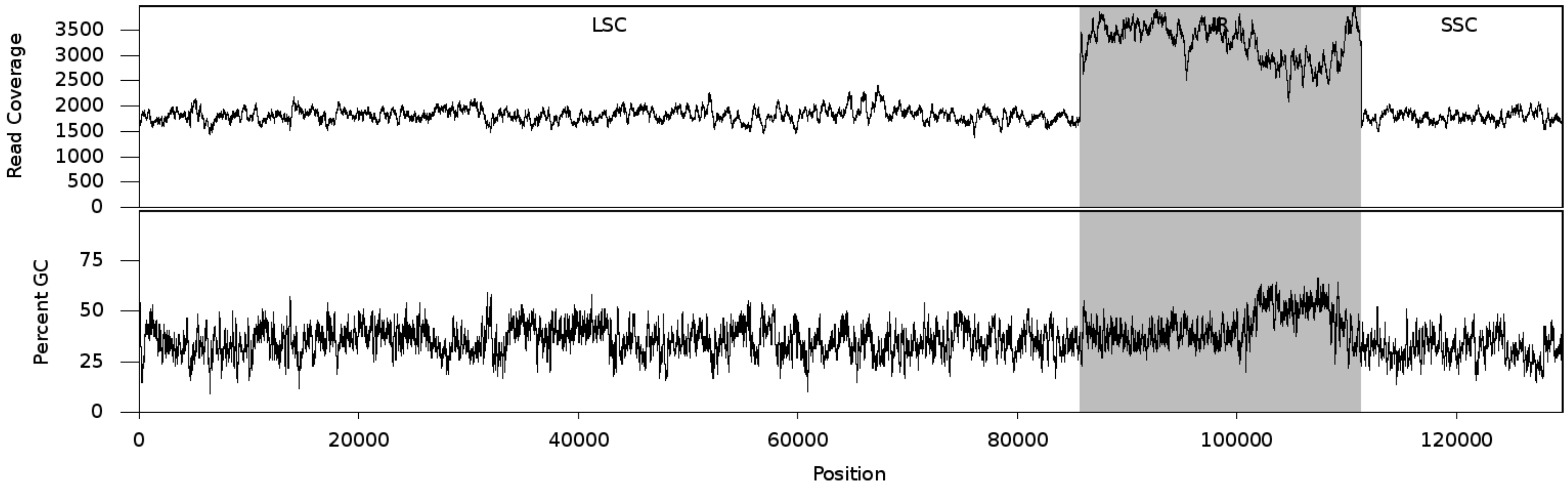

Solanum berthaultii PI 527886

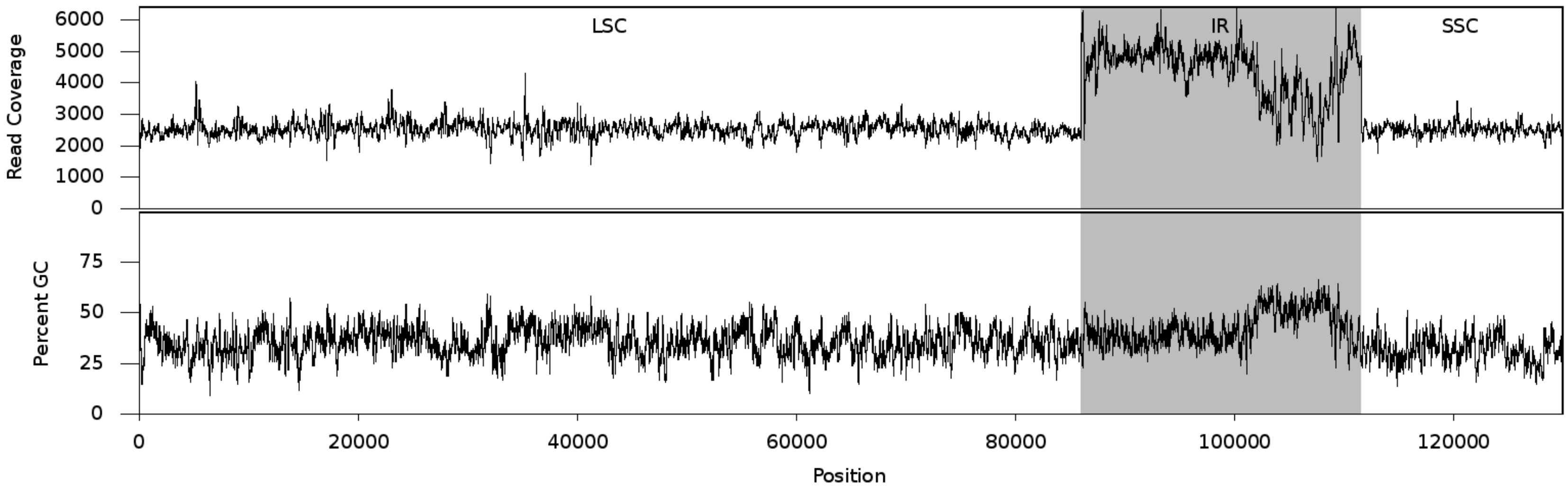

Solanum berthaultii PI 545850

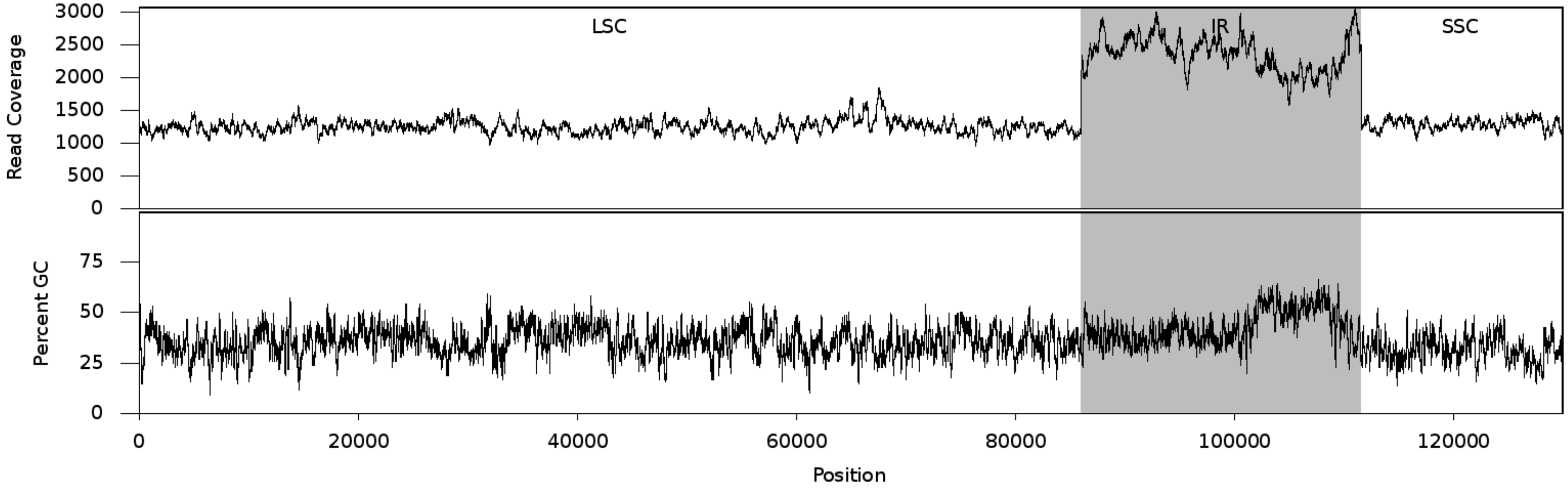

Solanum blanco-galdosii PI 498214

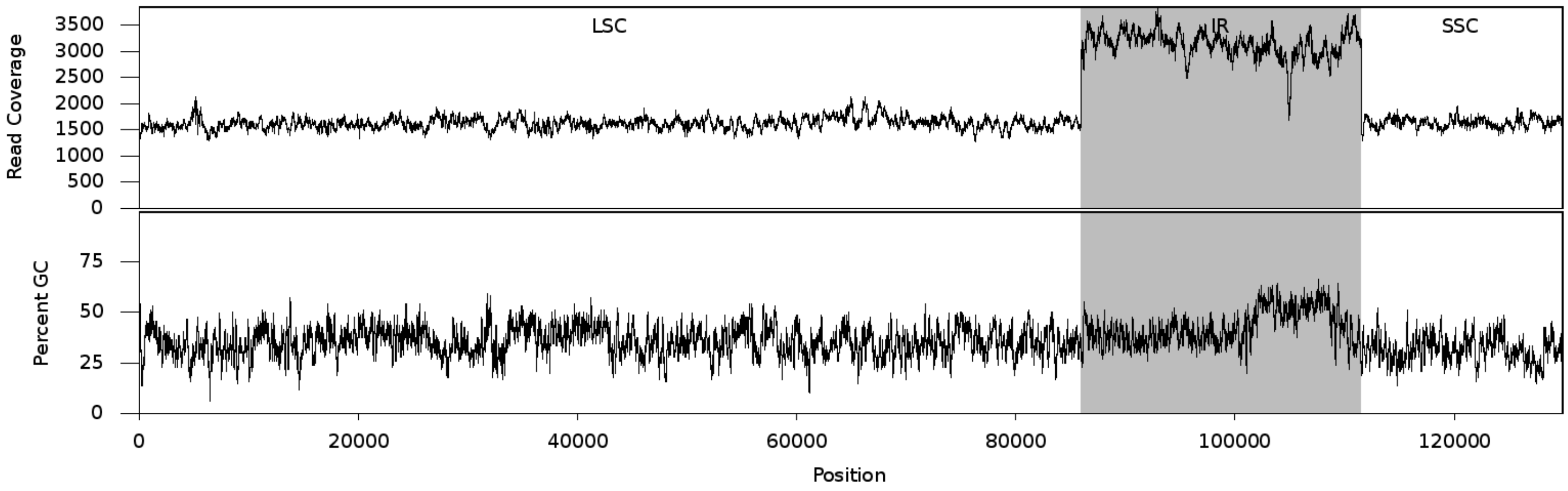

Solanum brevicaule PI 310931

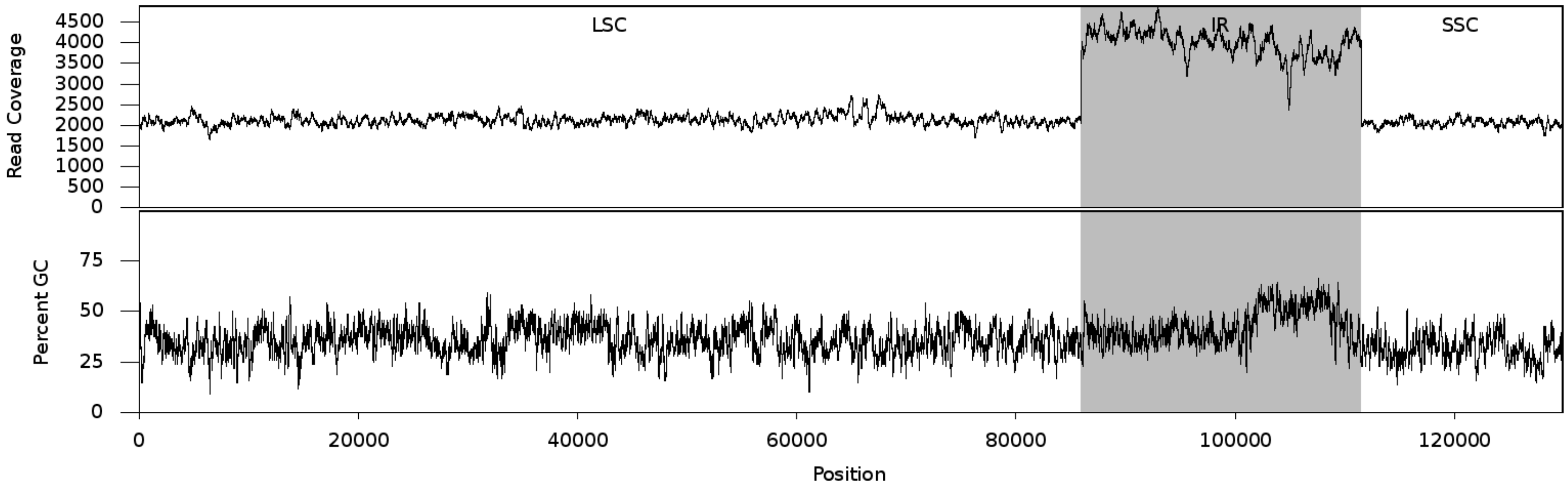

Solanum brevicaule PI 473378

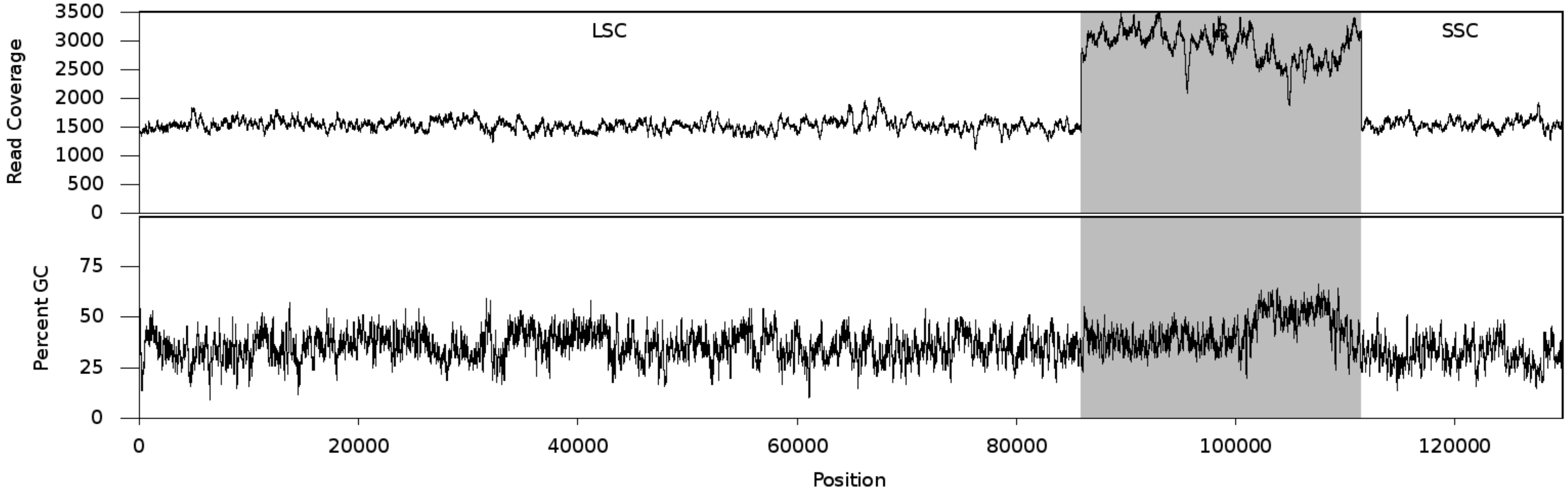

Solanum brevicaule PI 498111

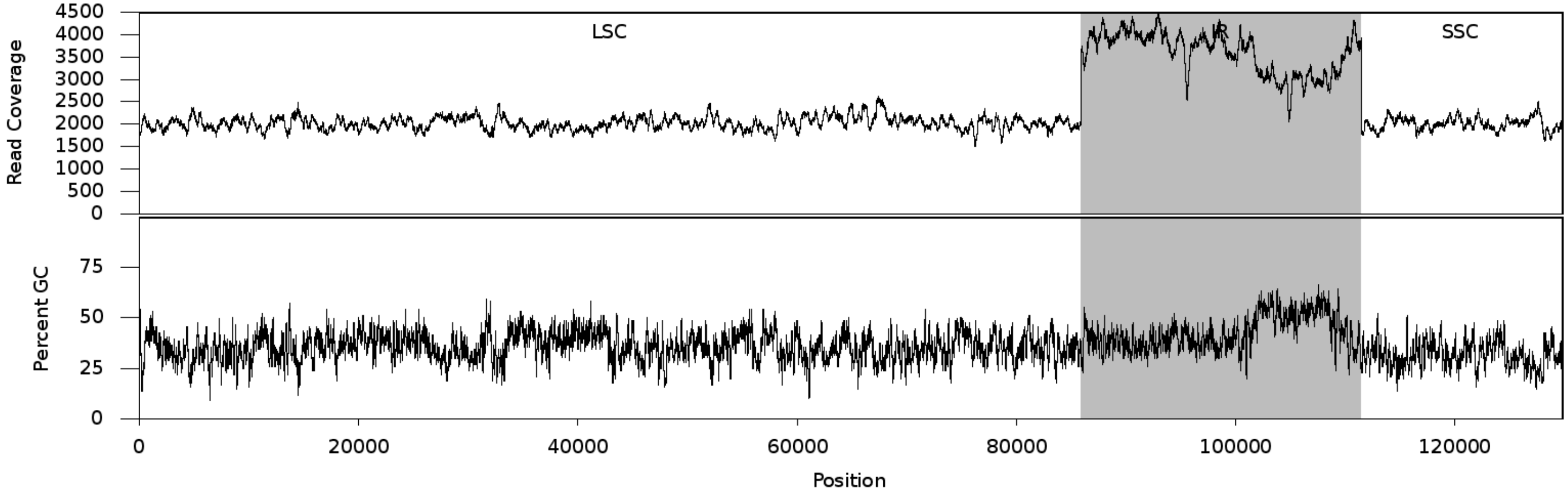

Solanum brevicaule PI 498218

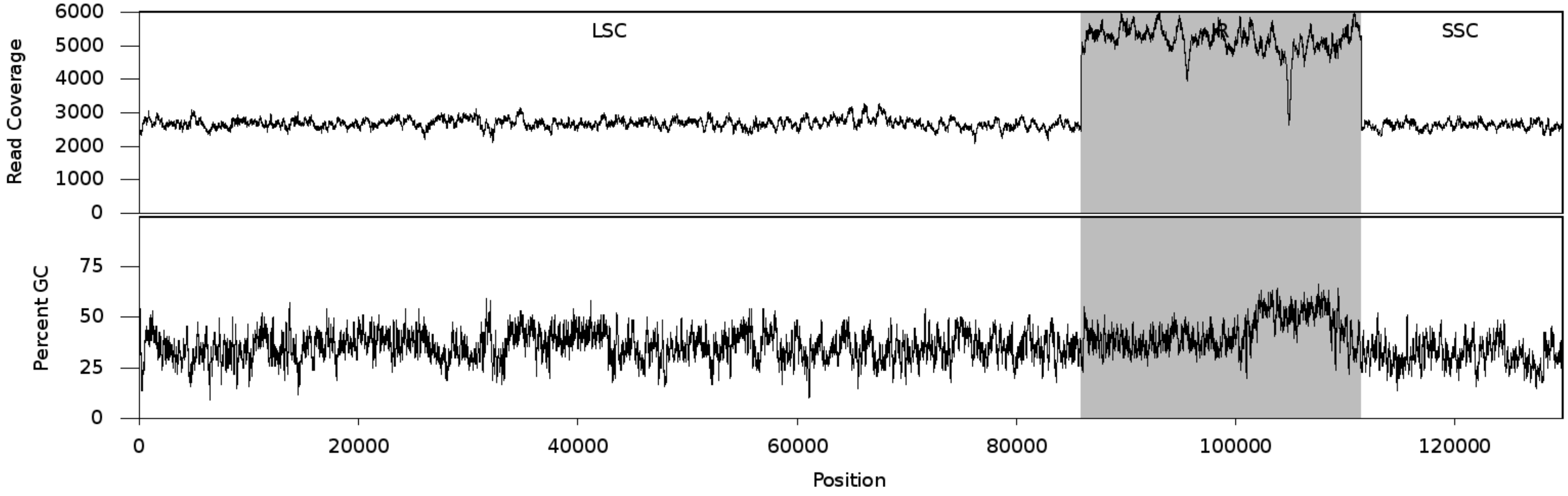

Solanum brevicaule PI 545968

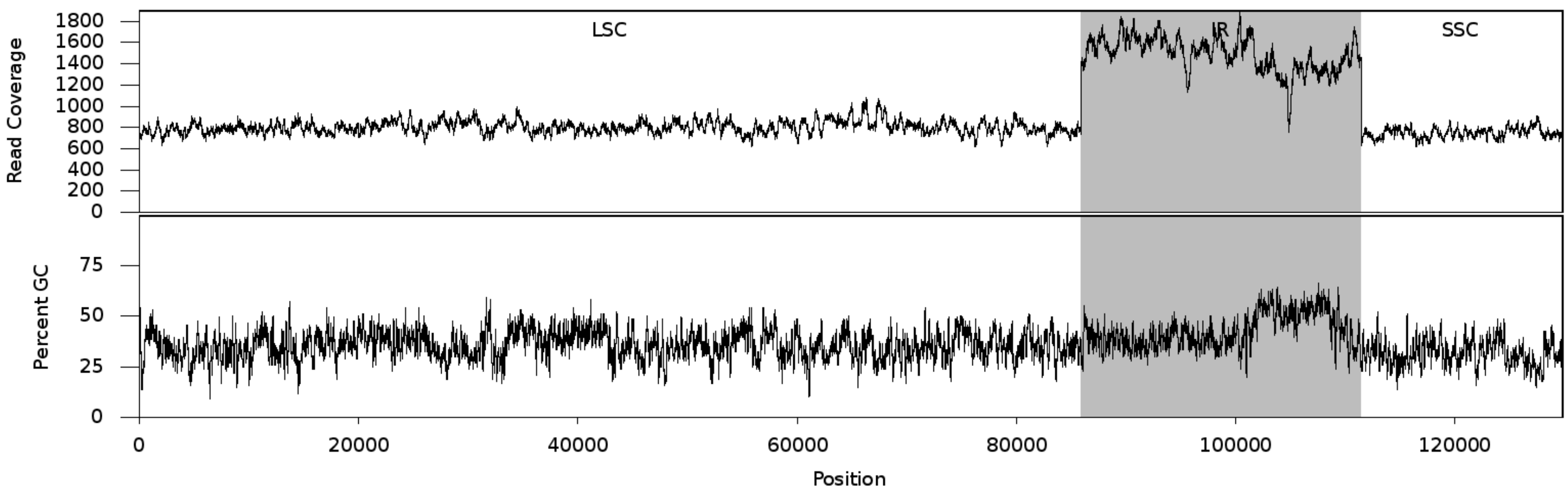

Solanum brevicaule PI 545970

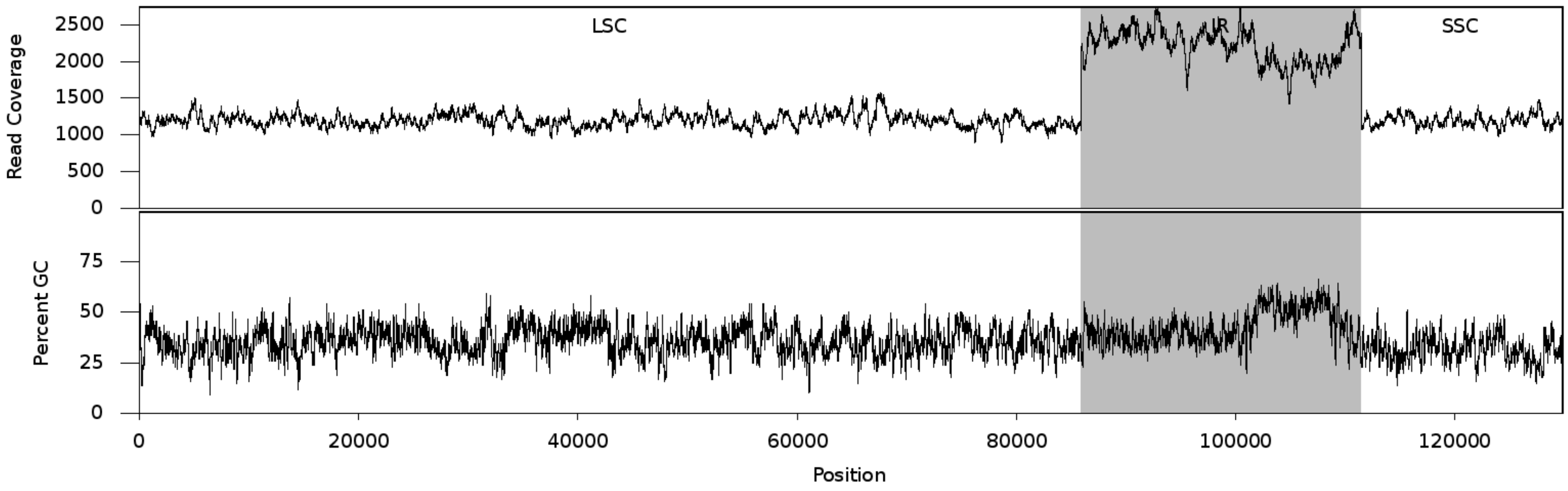

Solanum brevicaule PI 545971

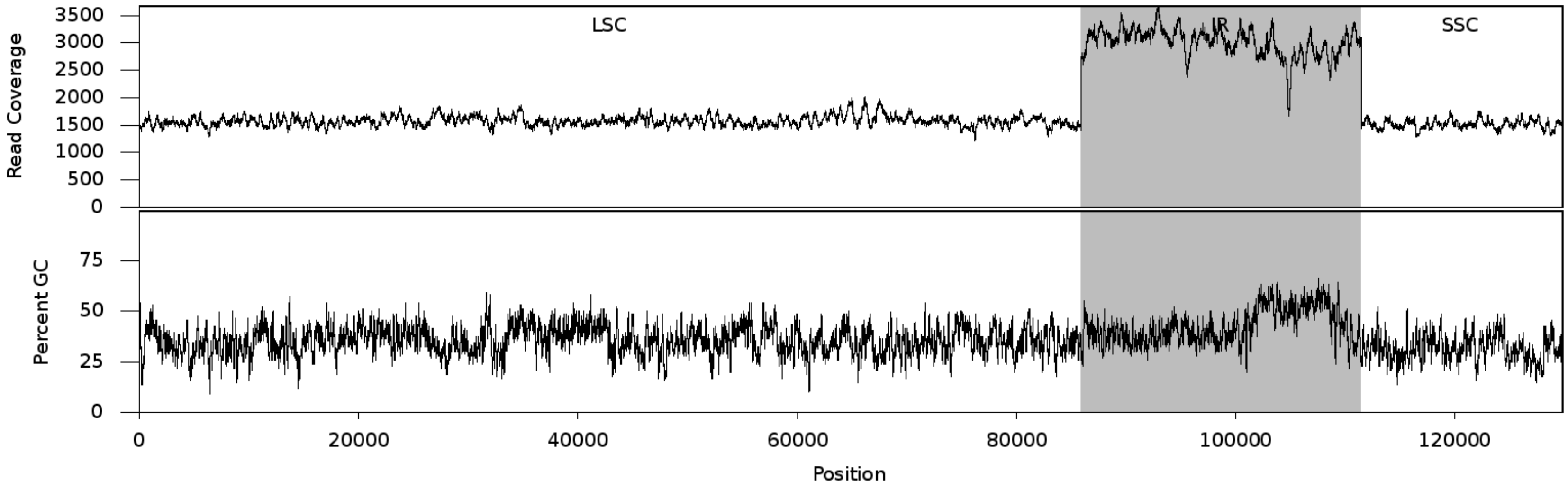

Solanum brevicaule PI 545981

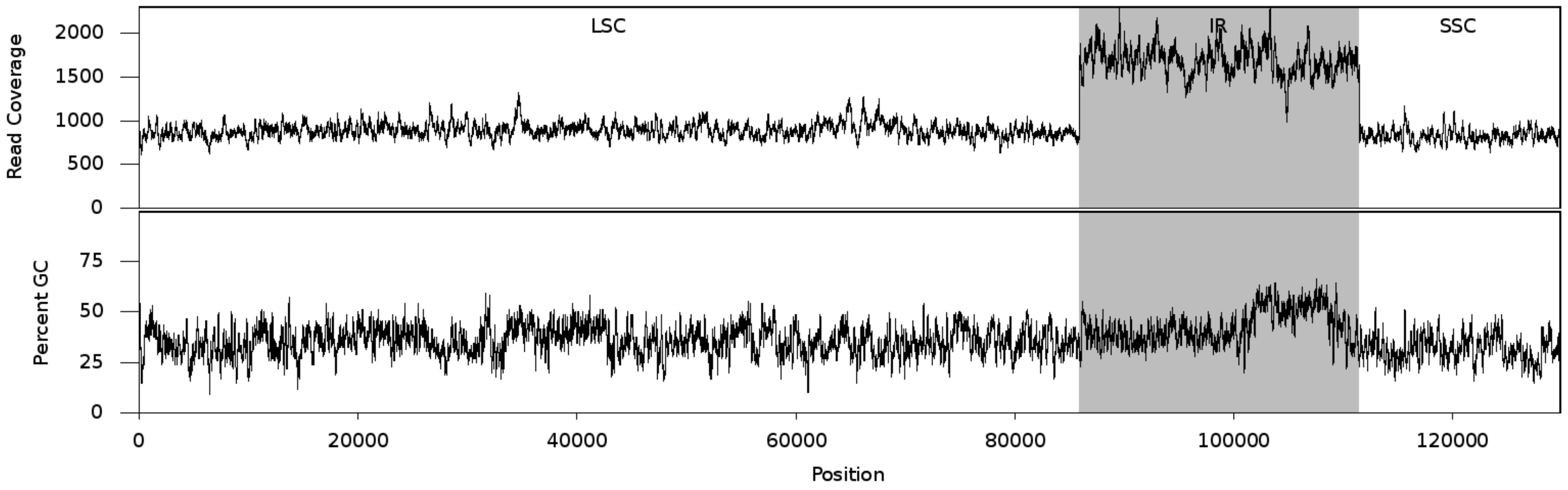

Solanum bukasovii PI 266385

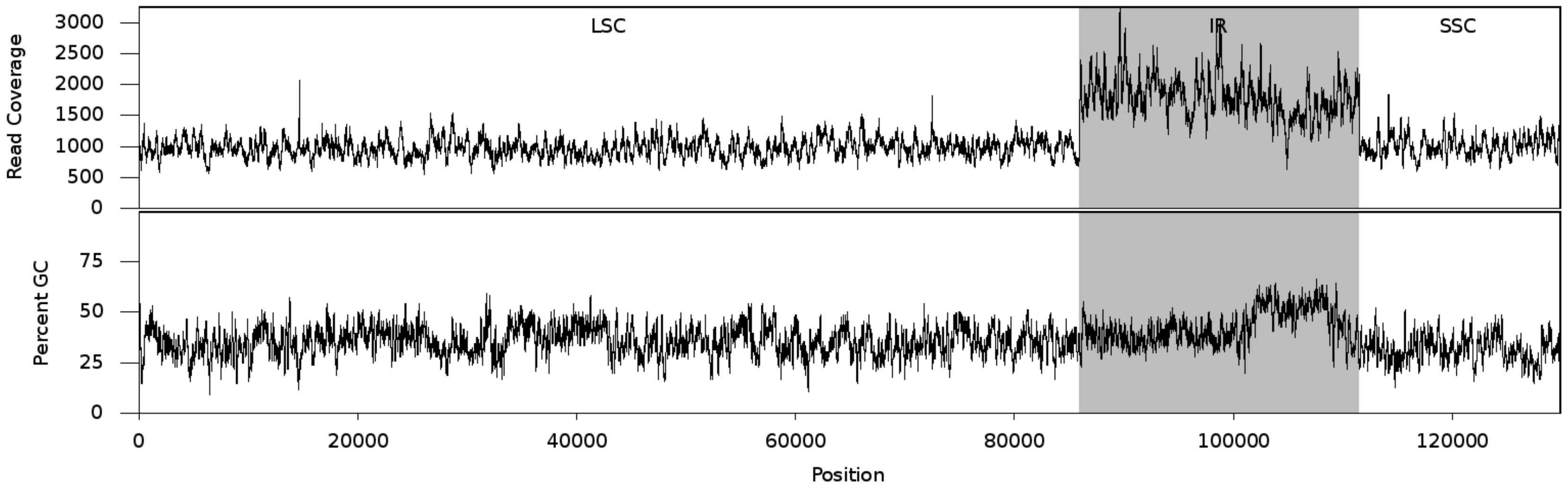

Solanum bukasovii PI 365353

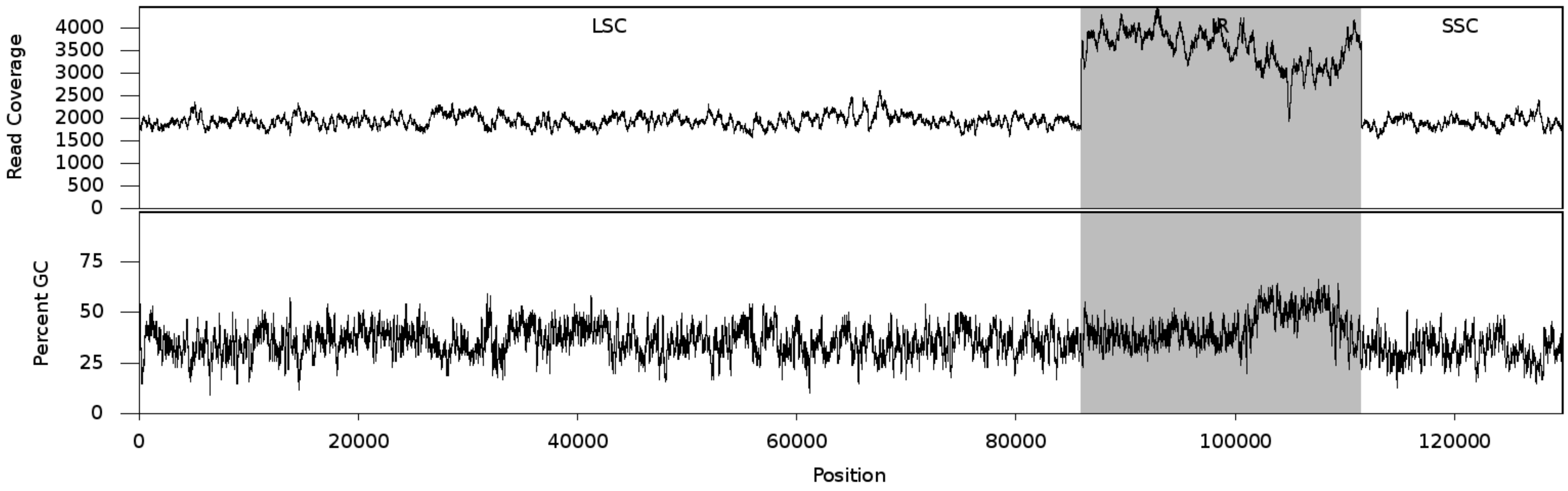

Solanum bukasovii PI 414155

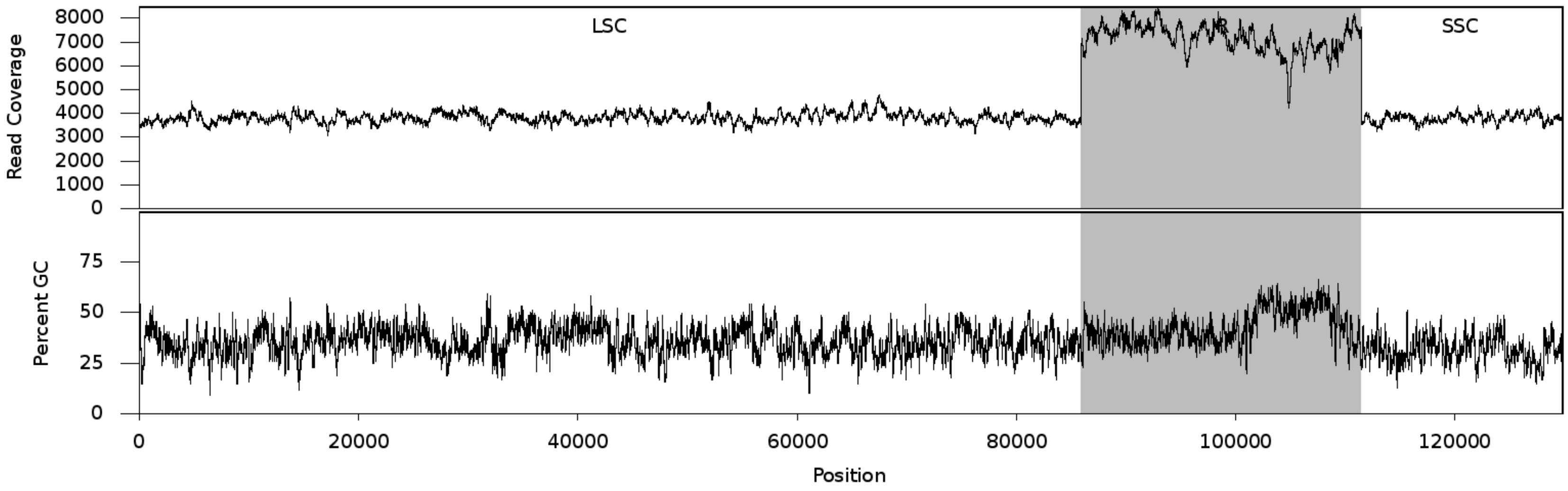

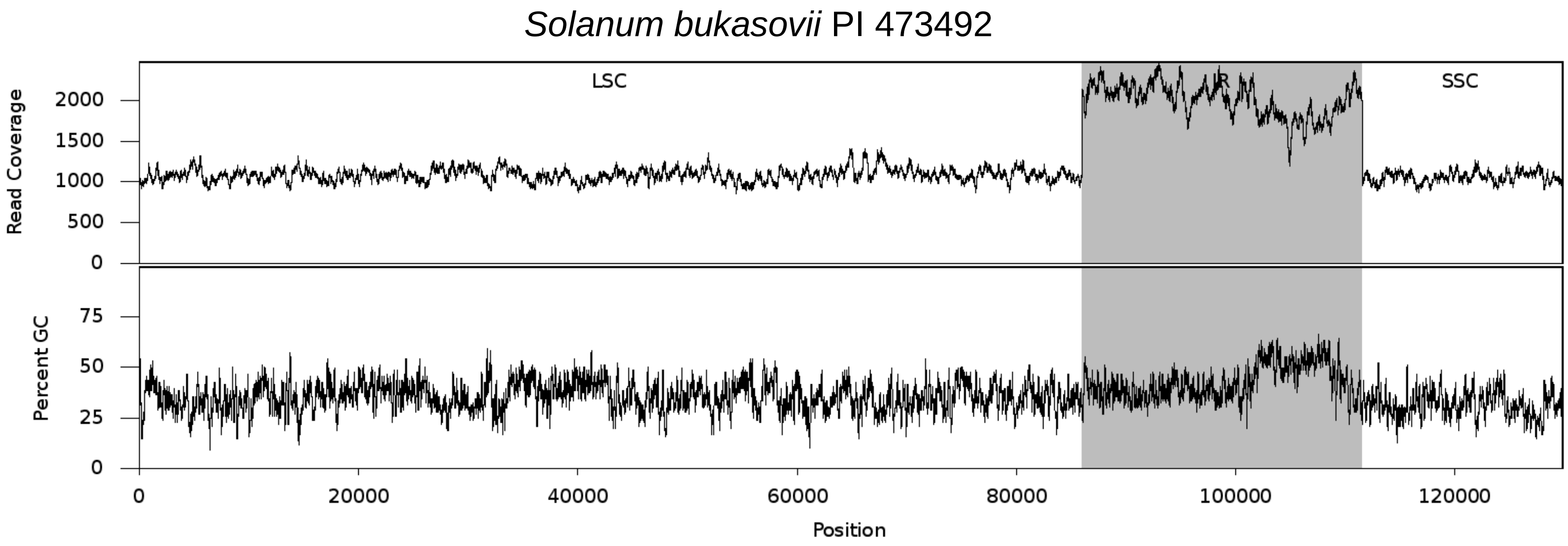
Solanum bukasovii PI 473492
LSC
IR
SSC
Read Coverage
Percent GC
Position

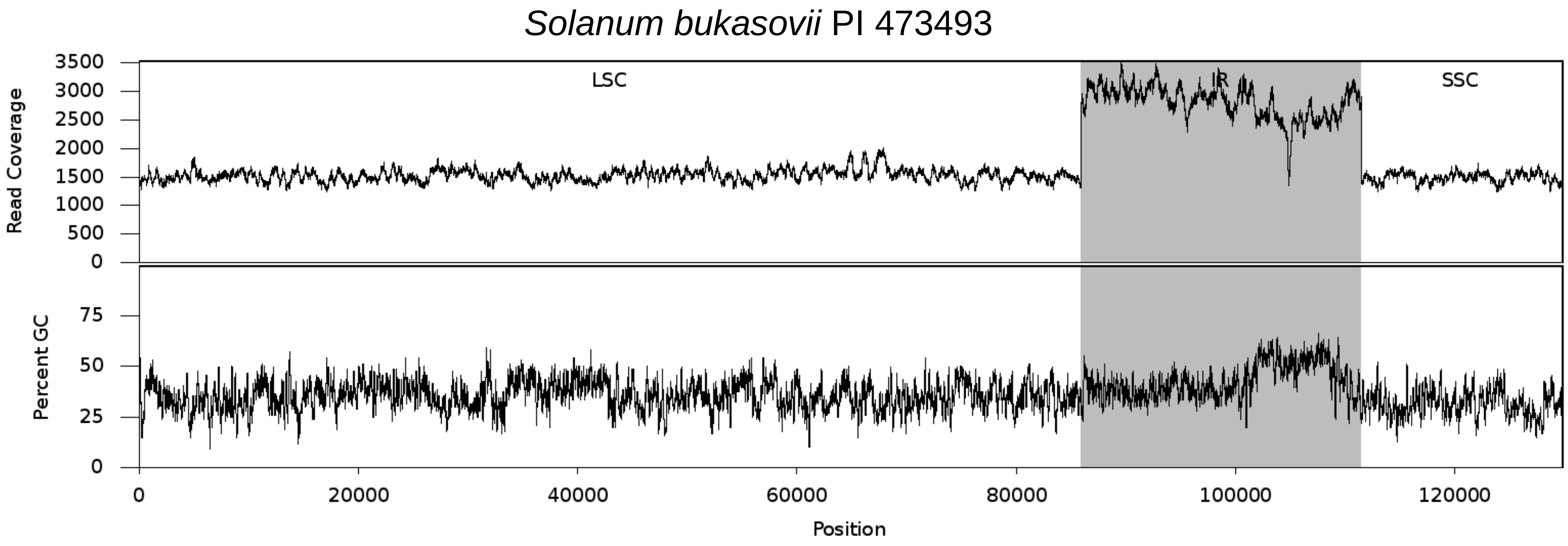
Solanum bukasovii PI 473493
LSC
IR
SSC
Read Coverage
Percent GC
Position

Solanum bukasovii PI 473494

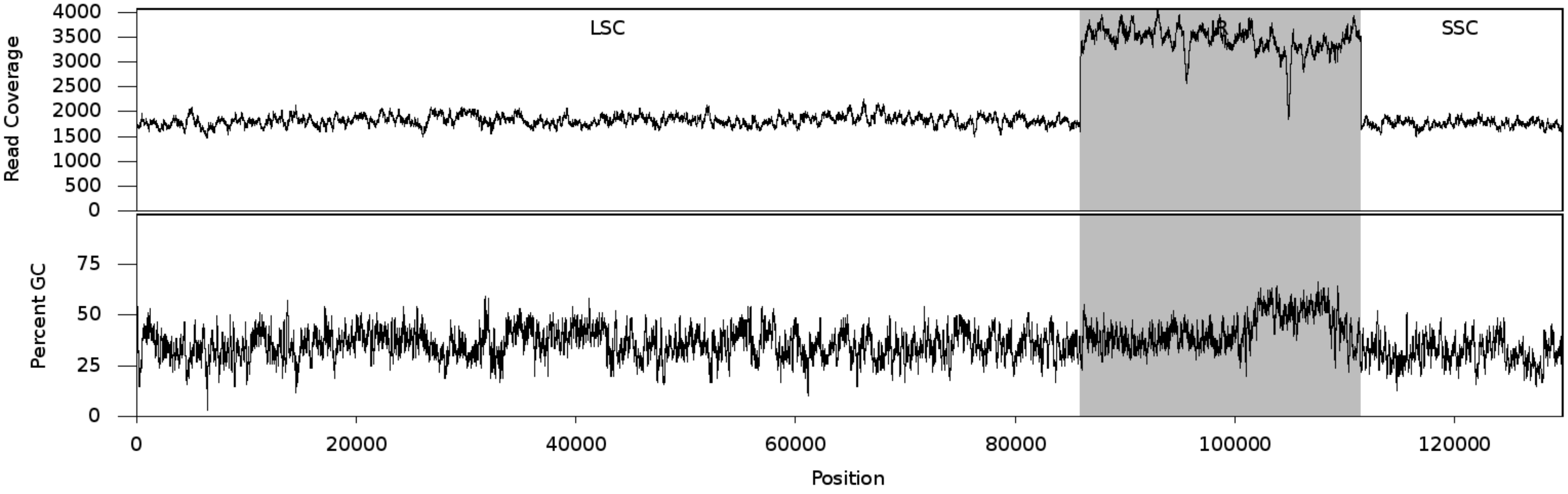

Solanum bukasovii PI 568933

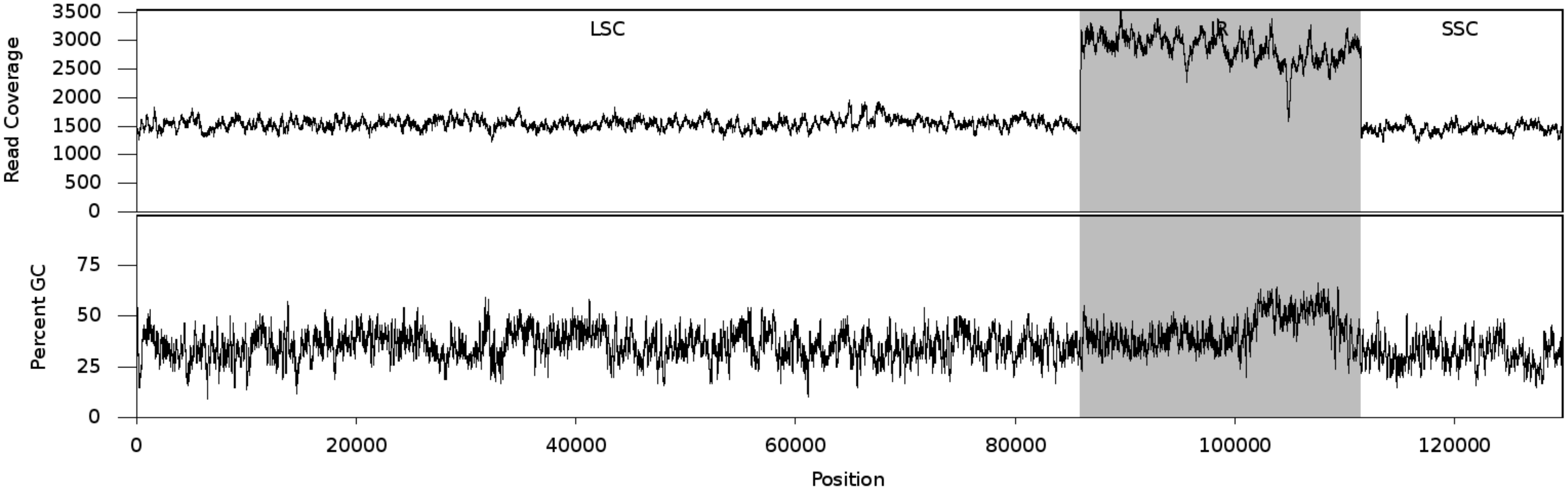

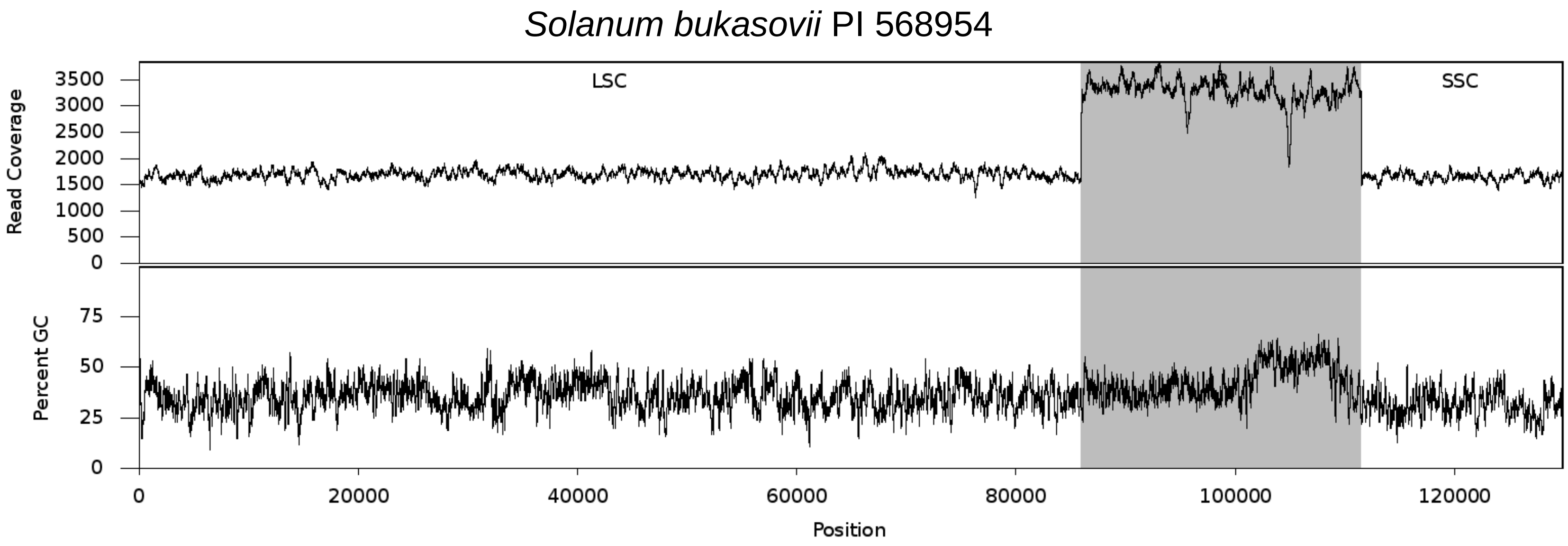

Solanum bukasovii PI 568954
LSC
IR
SSC
Read Coverage
3500
3000
2500
2000
1500
1000
500
0
Percent GC
75
50
25
0
0
20000
40000
60000
80000
100000
120000
Position

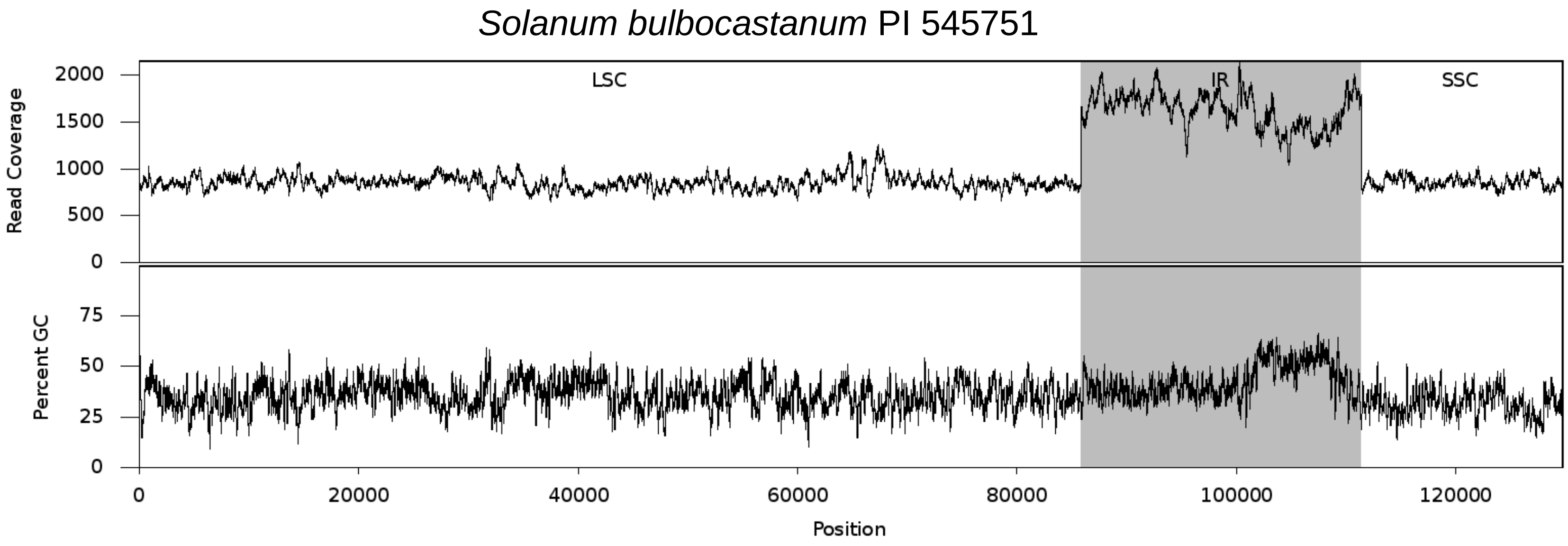

Solanum bulbocastanum PI 545751
LSC
IR
SSC
Read Coverage
2000
1500
1000
500
0
Percent GC
75
50
25
0
0
20000
40000
60000
80000
100000
120000
Position

Solanum bulbocastanum PI 604074

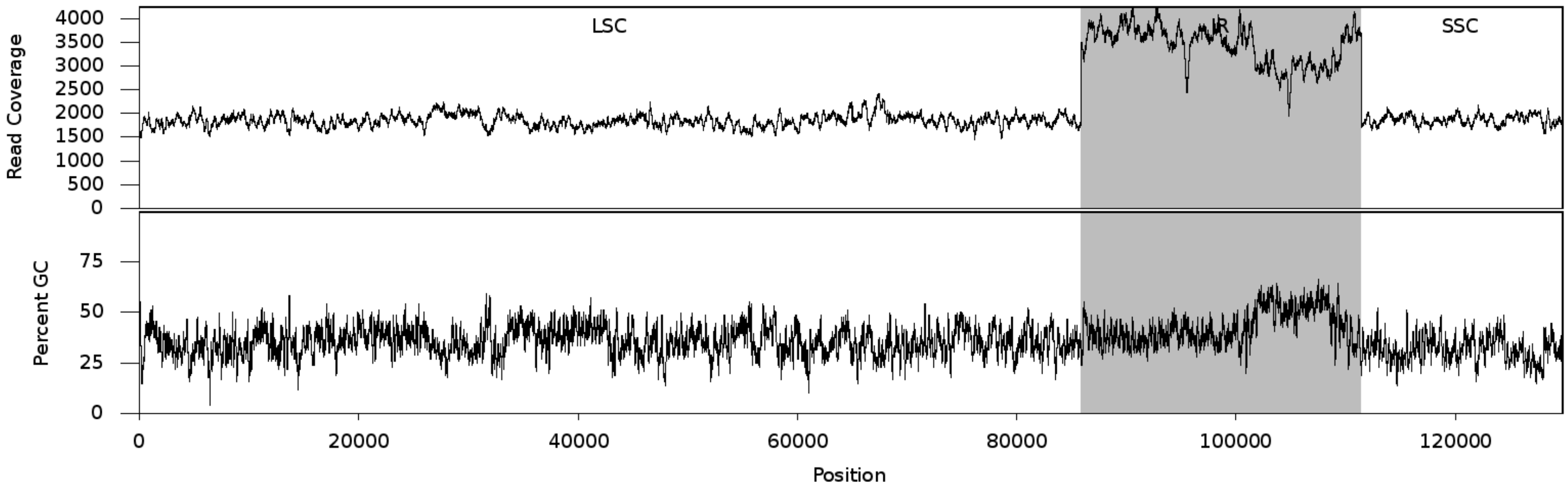

Solanum cajamarquense PI 230522

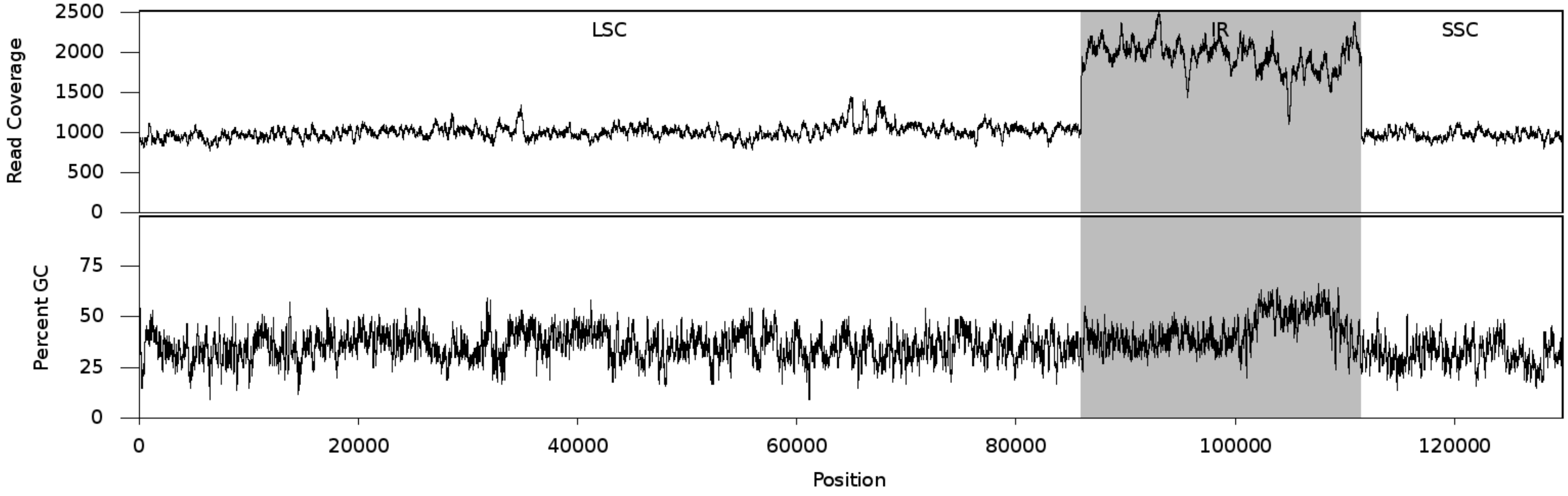

Solanum canasense PI 210035

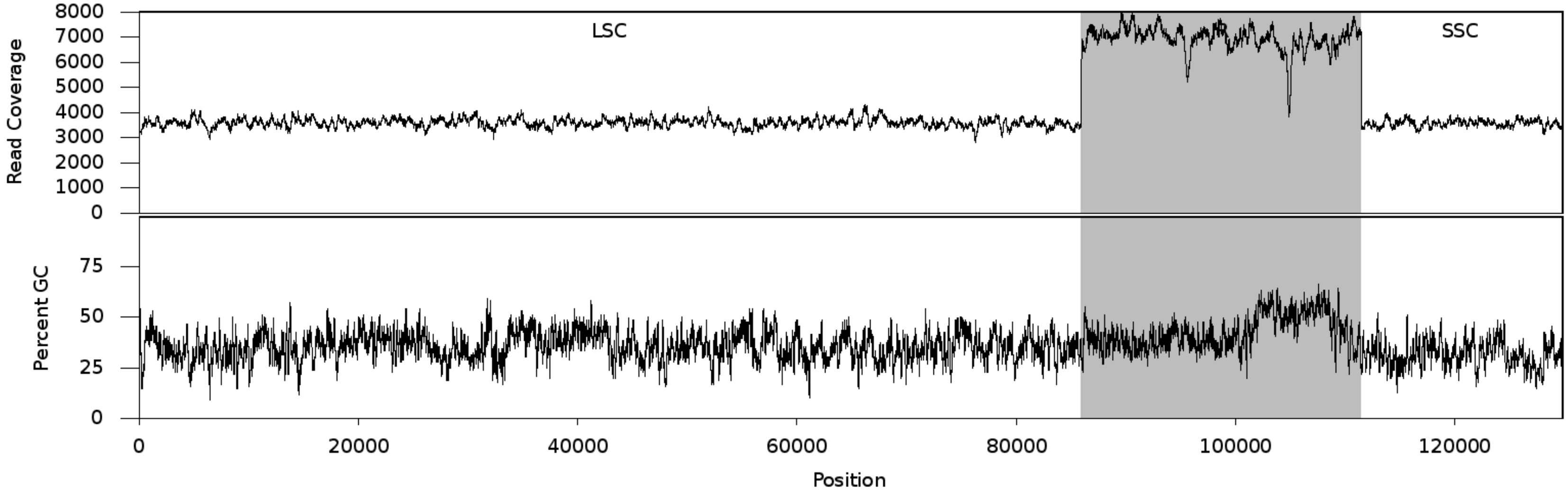

Solanum canasense PI 246533

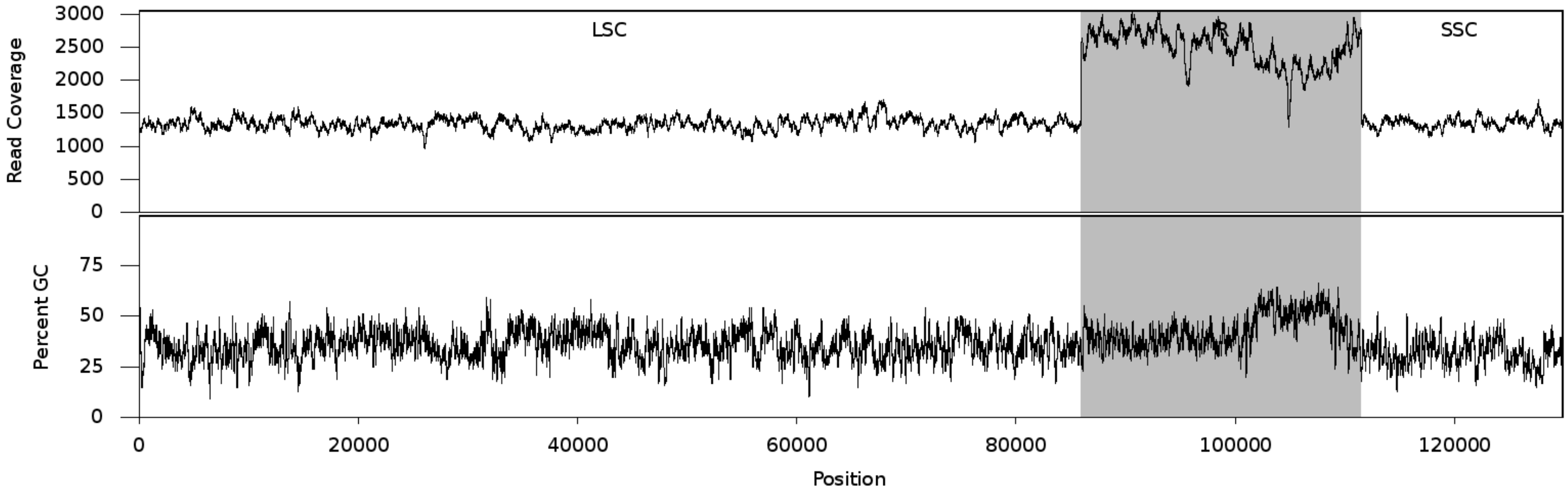

Solanum canasense PI 265864

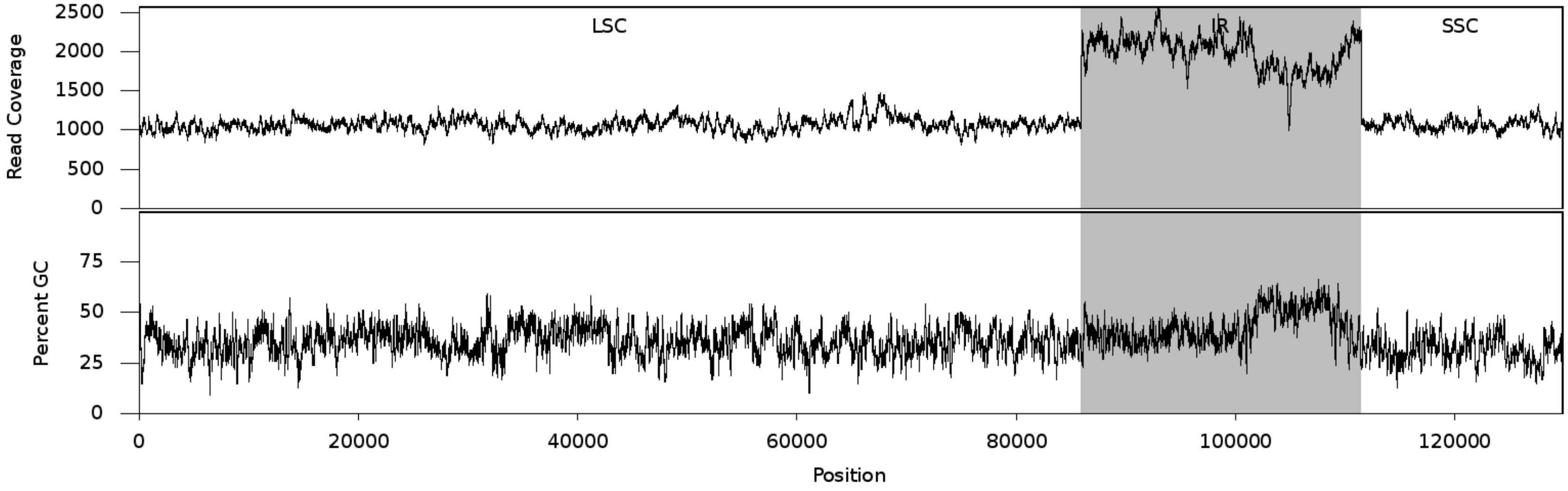

Solanum canasense PI 265865

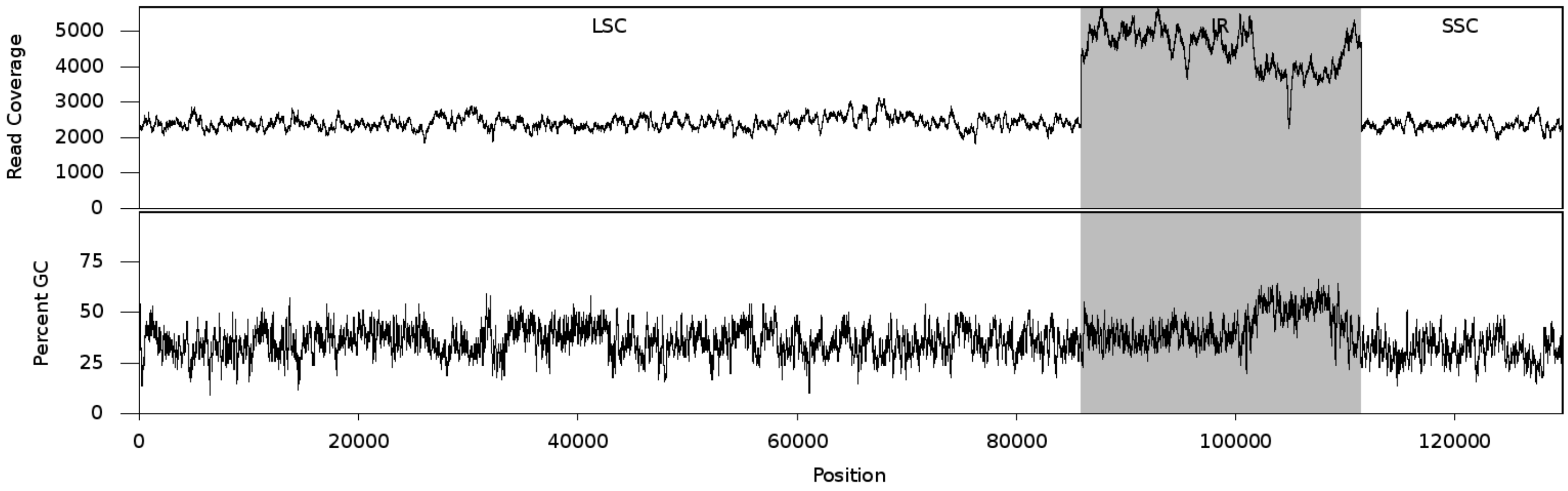

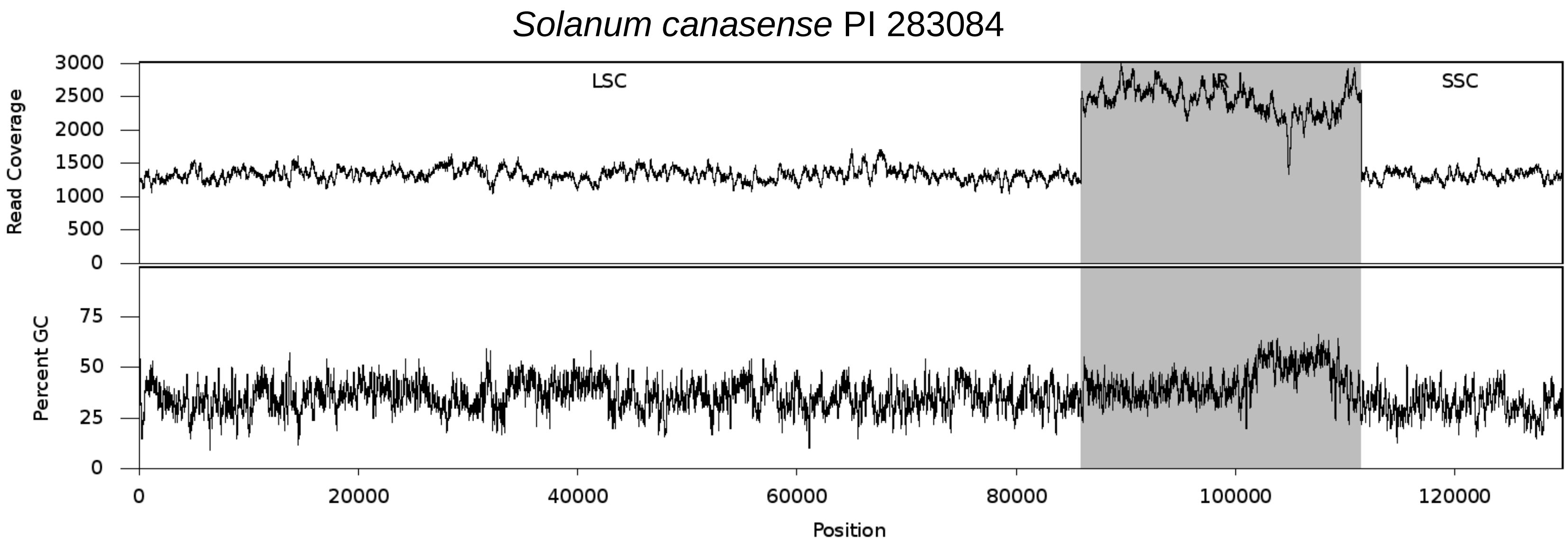
Solanum canasense PI 283084
Read Coverage
Percent GC
LSC
IR
SSC
Position

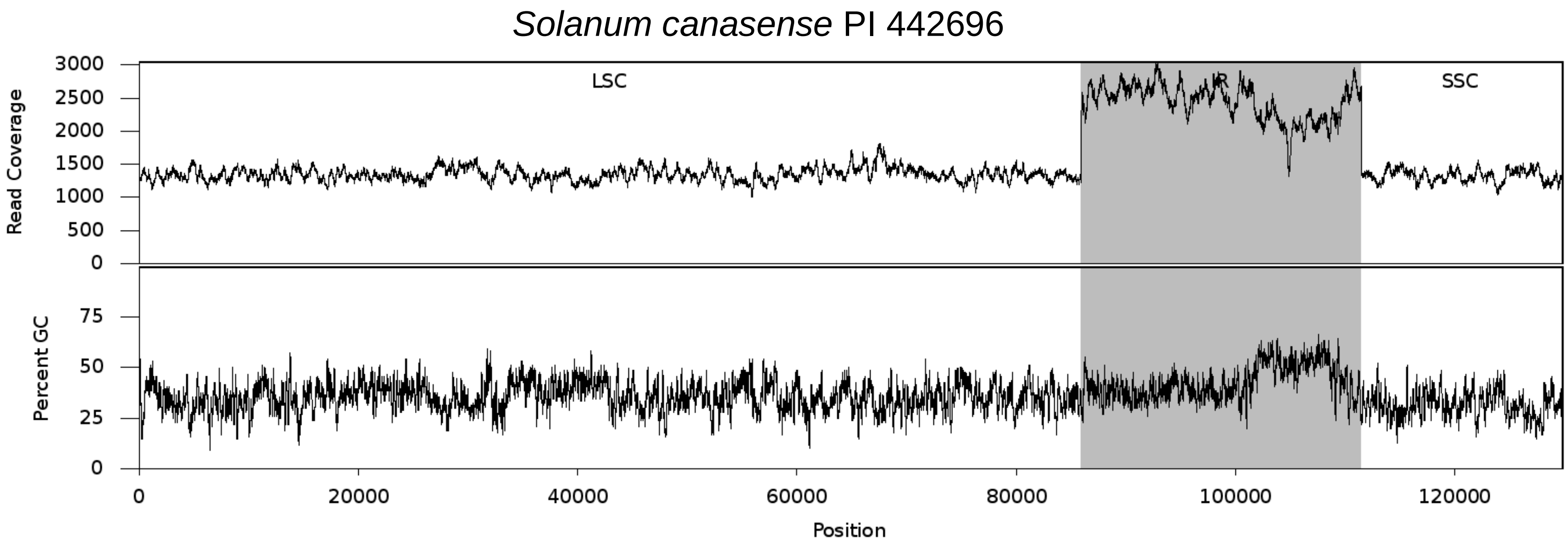
Solanum canasense PI 442696
Read Coverage
Percent GC
LSC
IR
SSC
Position

Solanum canasense PI 473355

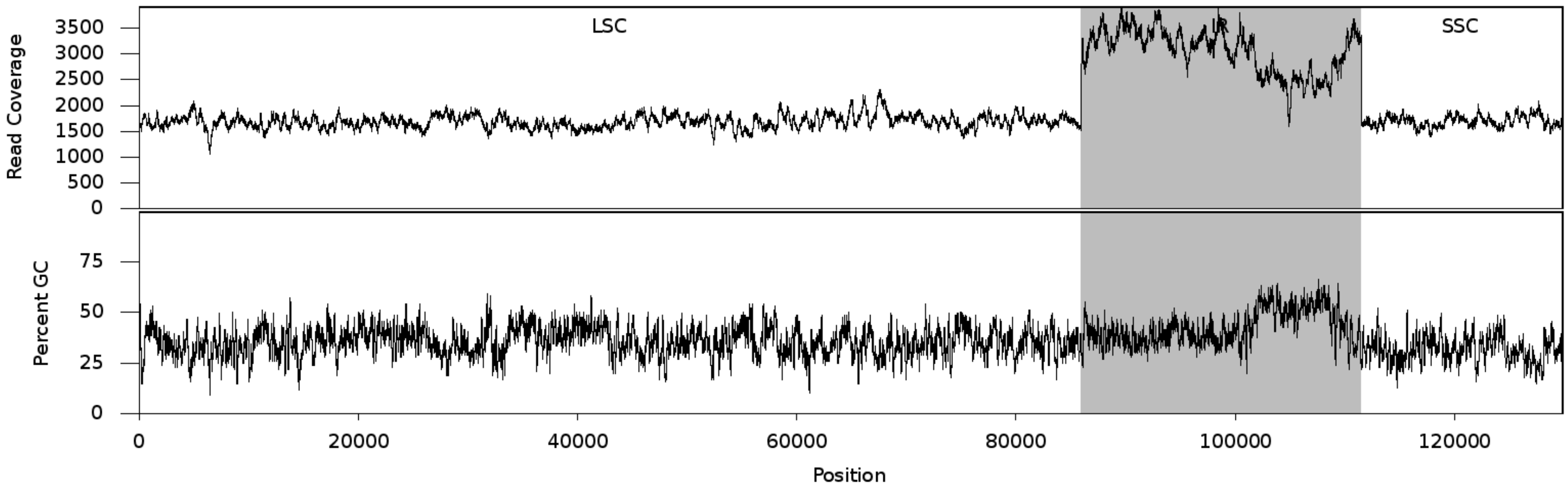

Solanum canasense PI 498226

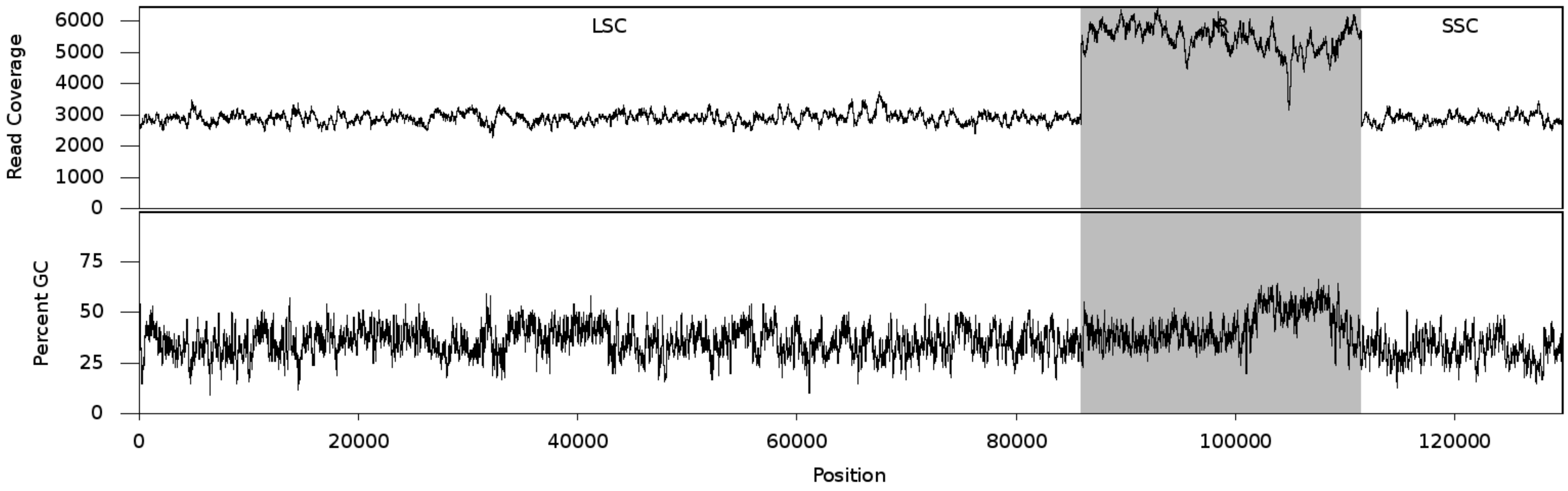

Solanum canasense PI 498227

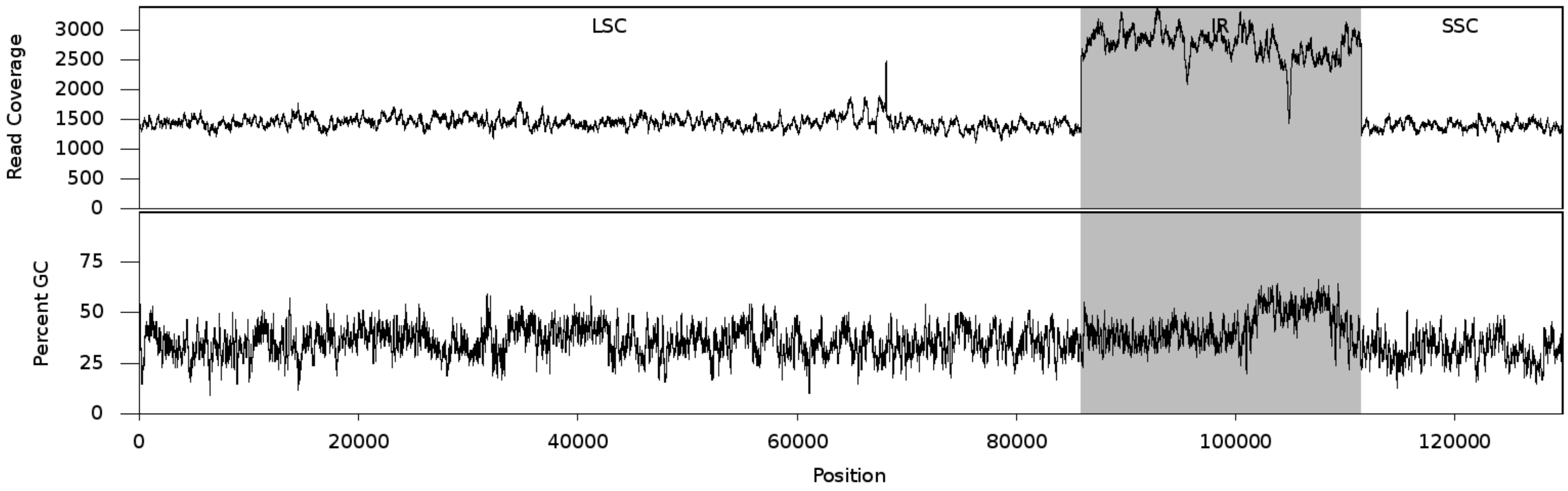

Solanum canasense PI 545972

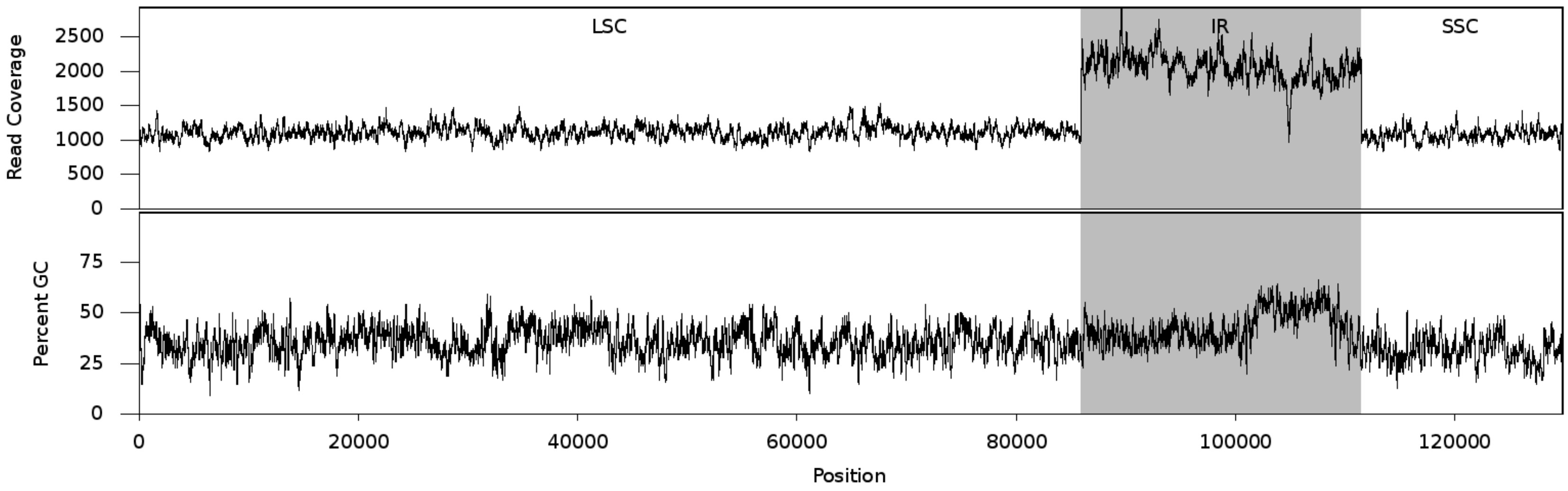

Solanum canasense PI 568969

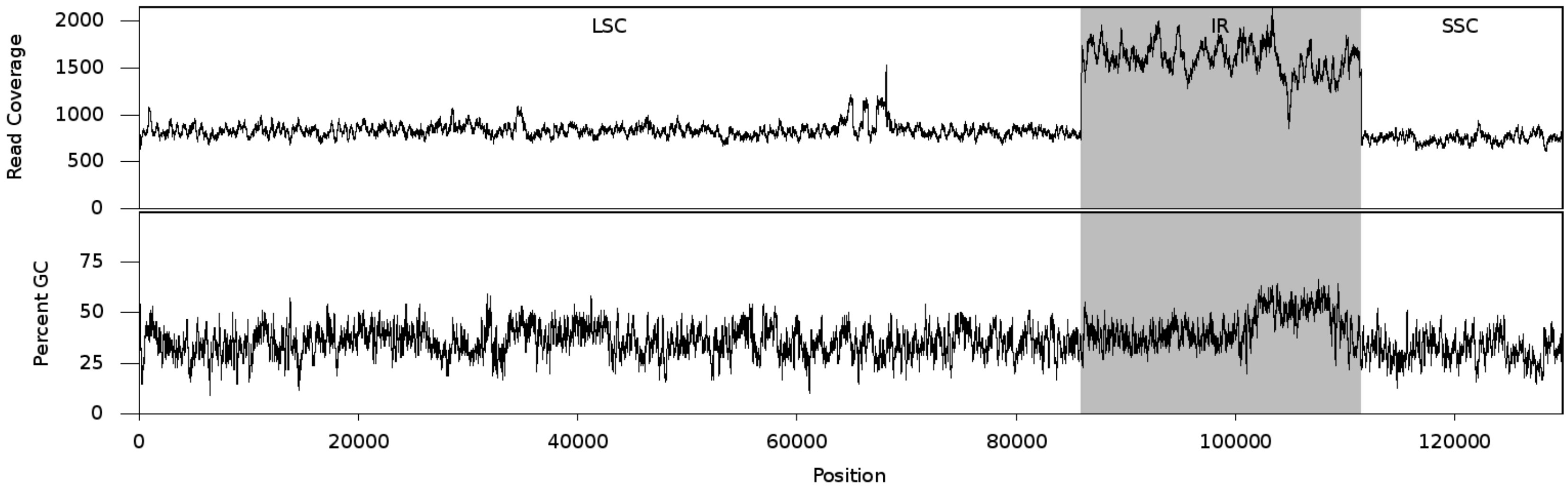

Solanum cardiophyllum PI 283062

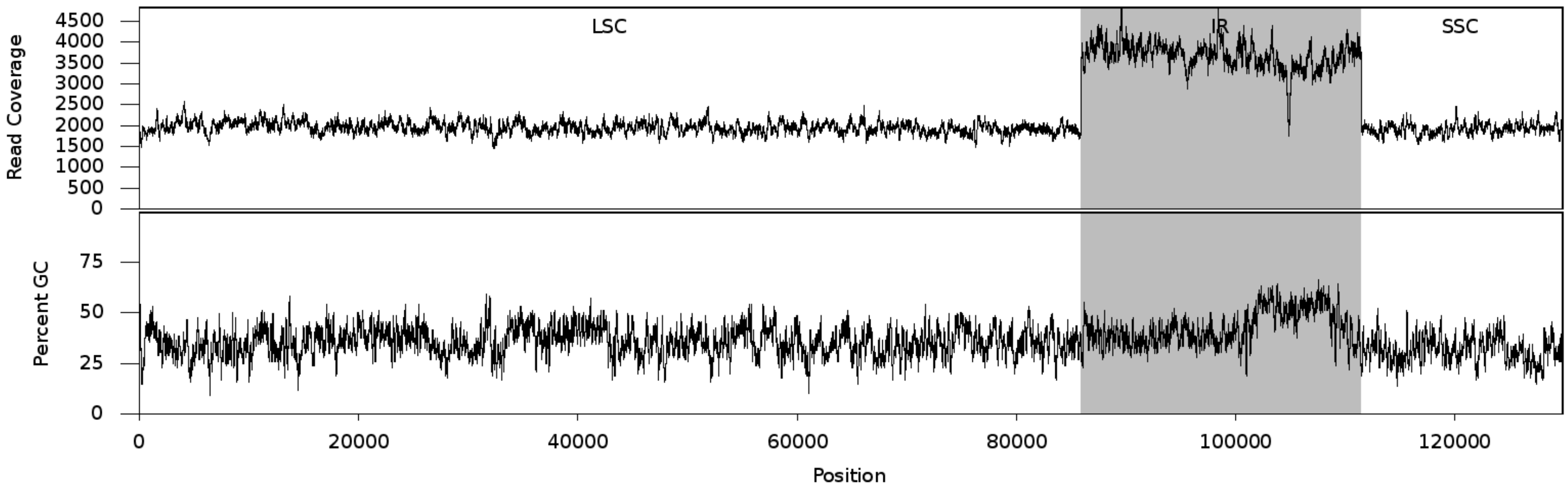

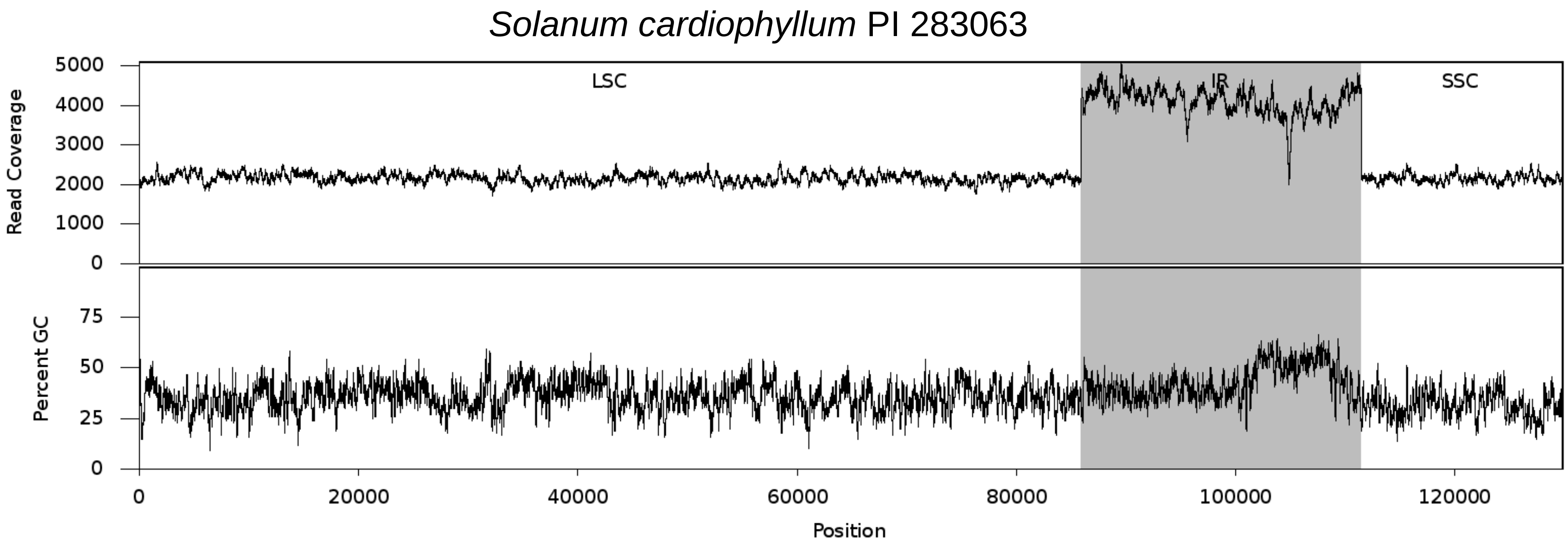
Solanum cardiophyllum PI 283063
LSC
IR
SSC
Read Coverage
Percent GC
Position
0
20000
40000
60000
80000
100000
120000

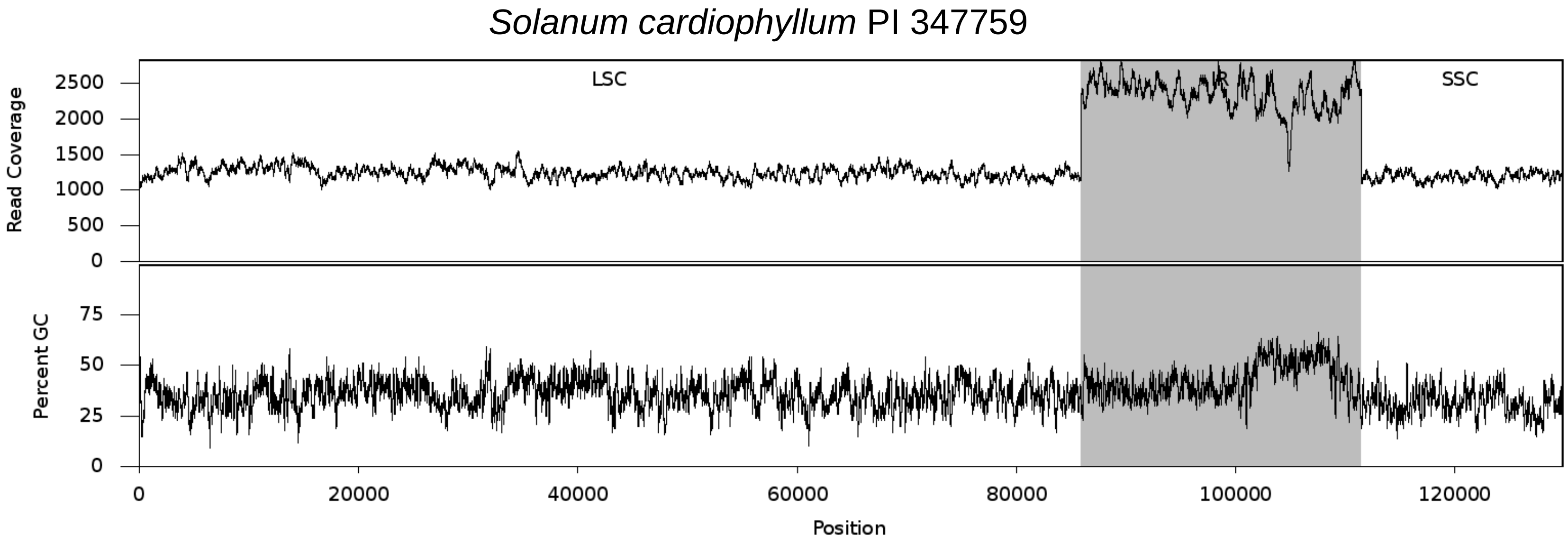
Solanum cardiophyllum PI 347759
LSC
IR
SSC
Read Coverage
Percent GC
Position
0
20000
40000
60000
80000
100000
120000

Solanum chacoense PI 275138

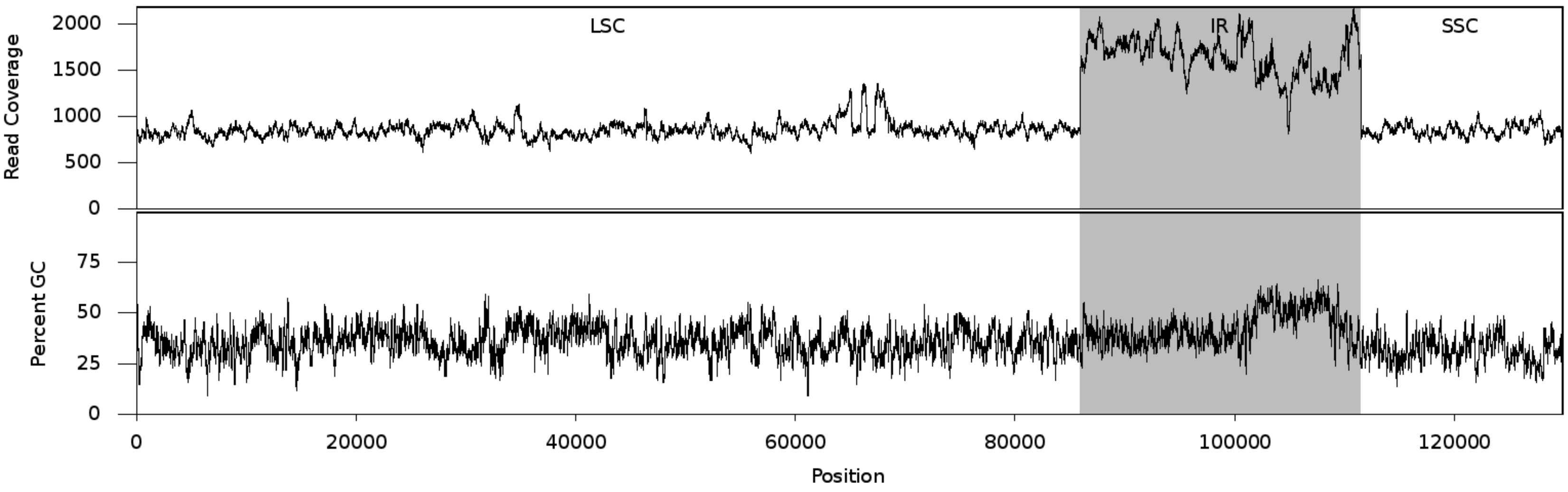

Solanum chacoense PI 320294

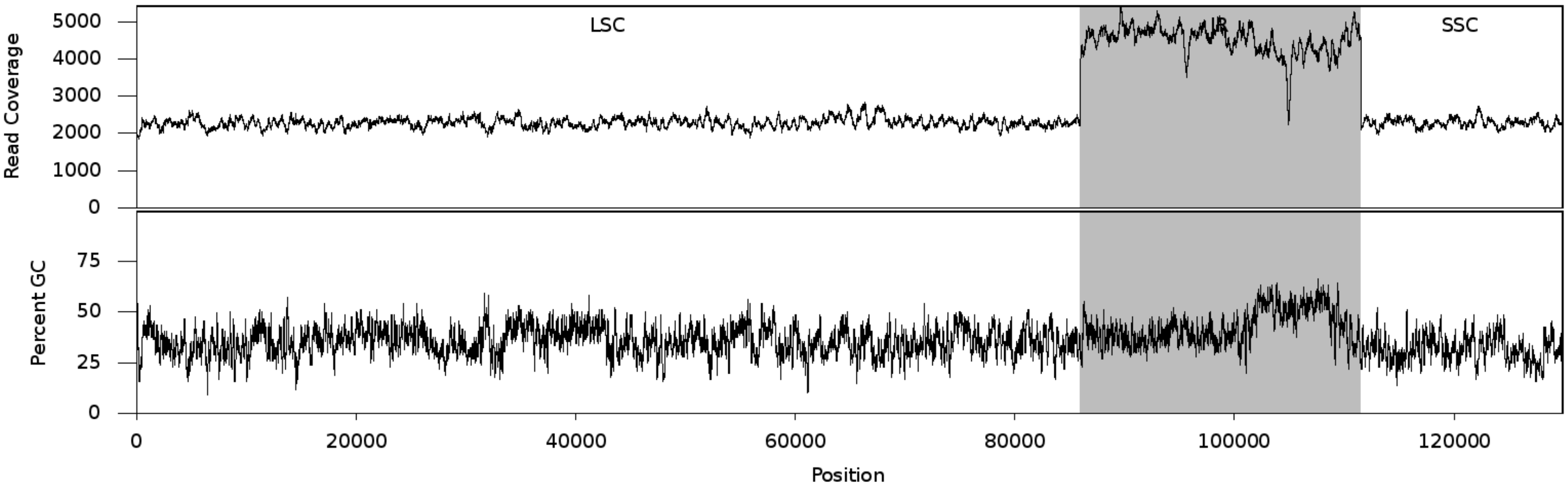

Solanum chacoense PI 472816

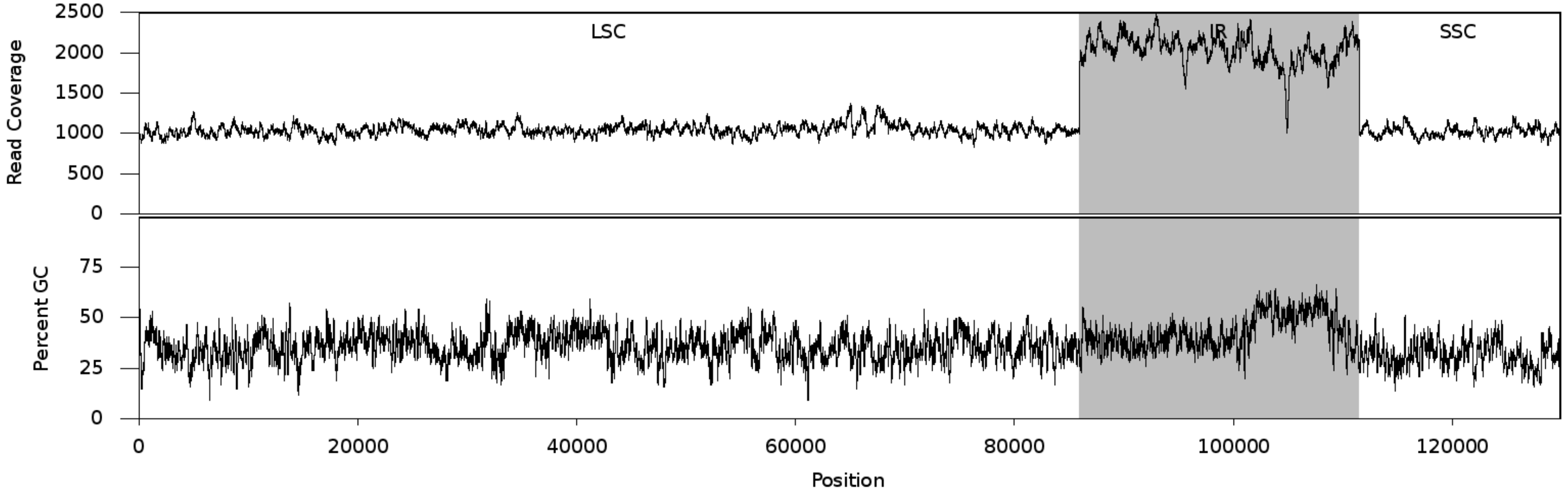

Solanum chacoense PI 472830

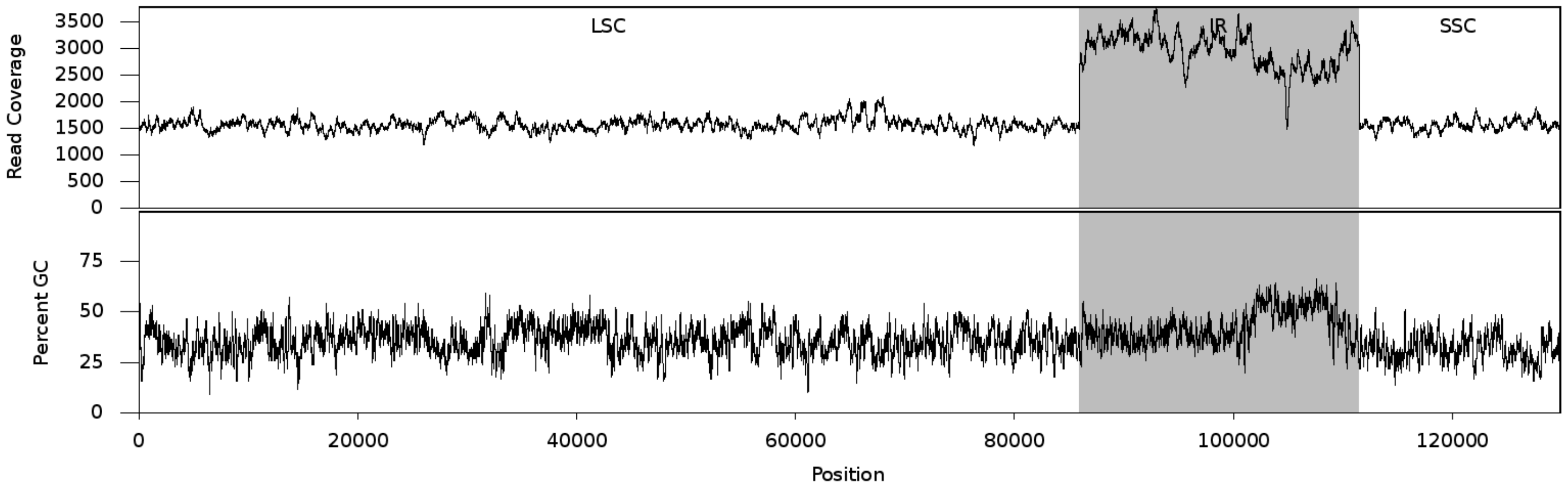

Solanum chacoense PI 500020

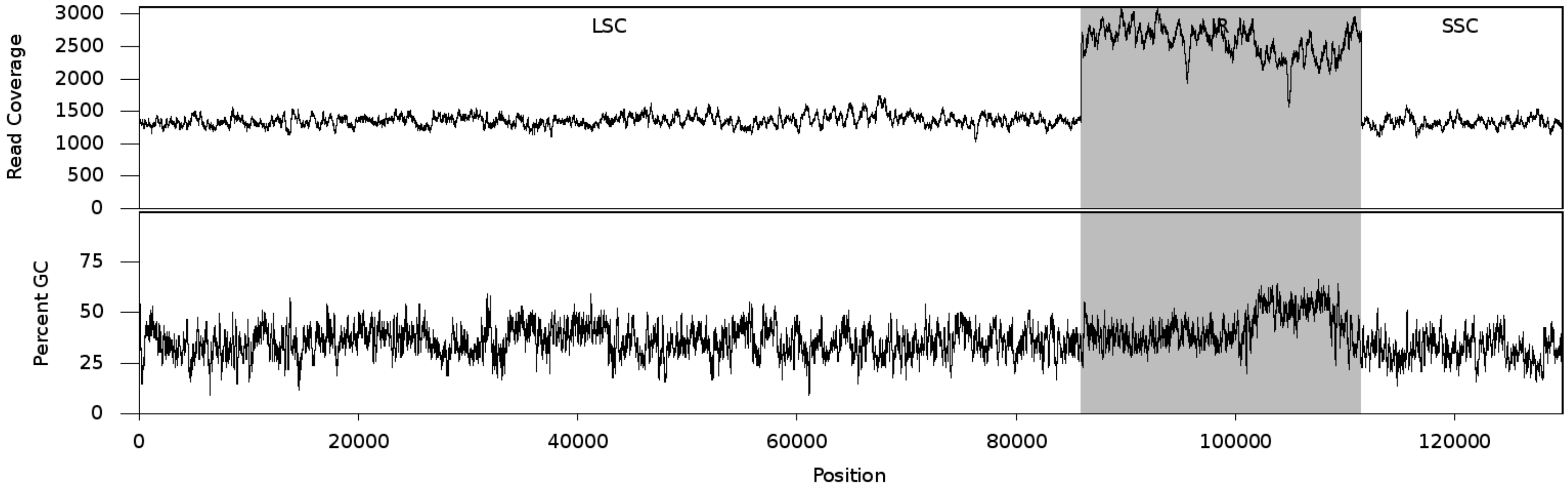

Solanum chomatophilum PI 365328

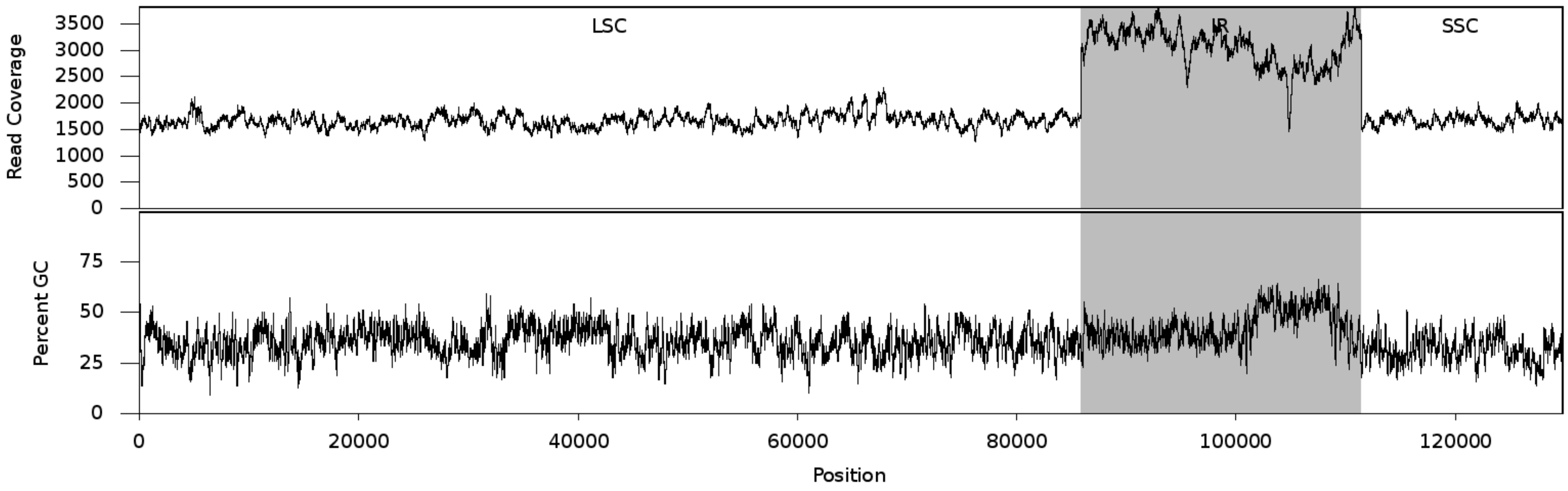

Solanum chomatophilum PI 365339

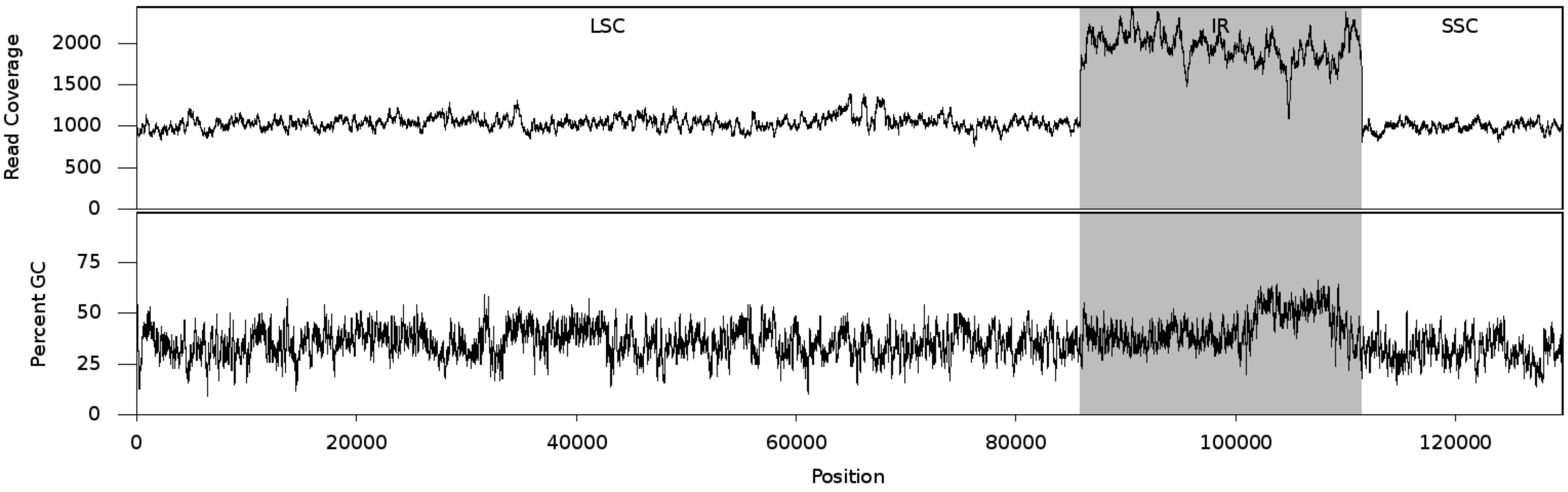

Solanum chomatophilum PI 473489

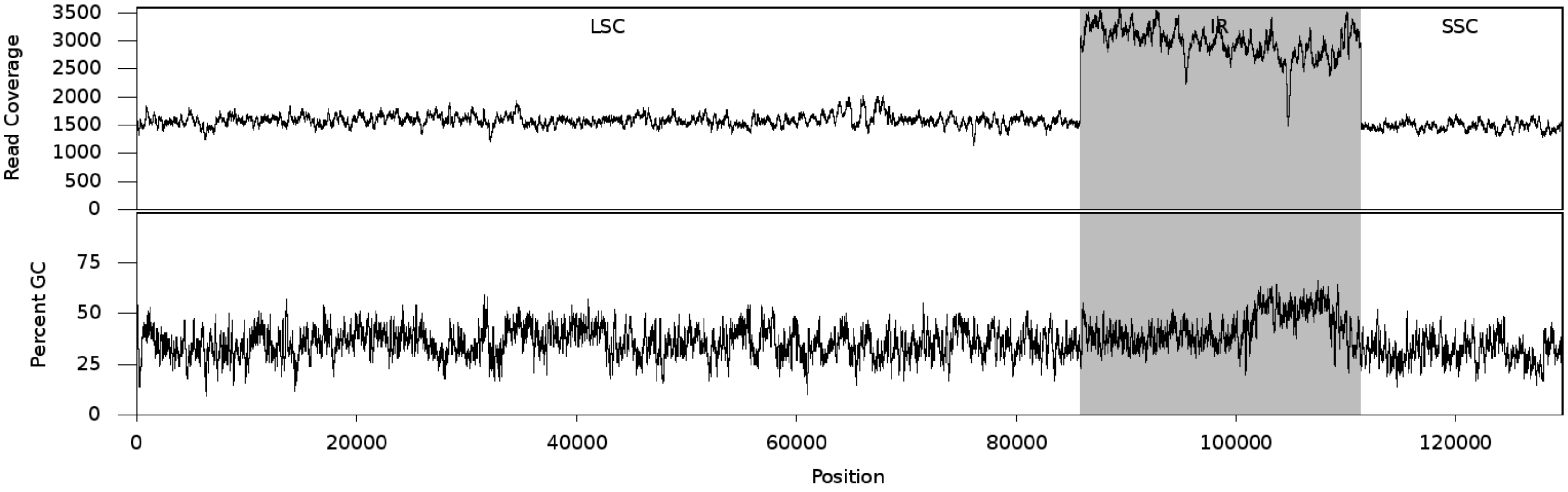

Solanum etuberosum PI 498311

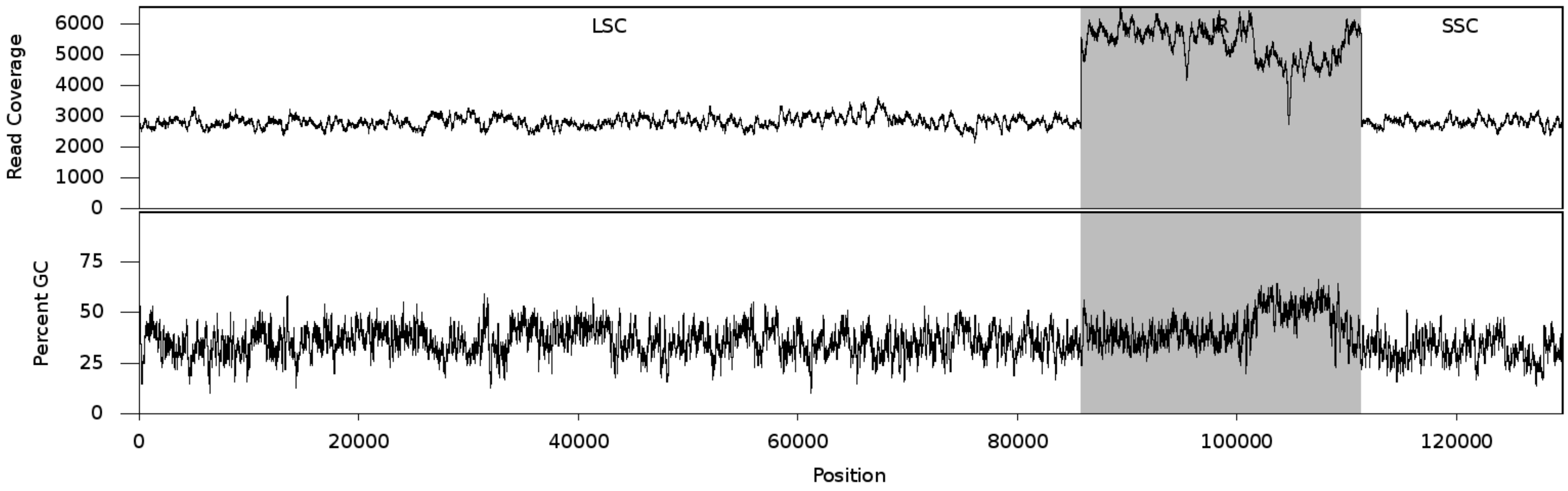

Solanum goniocalyx PI 195186

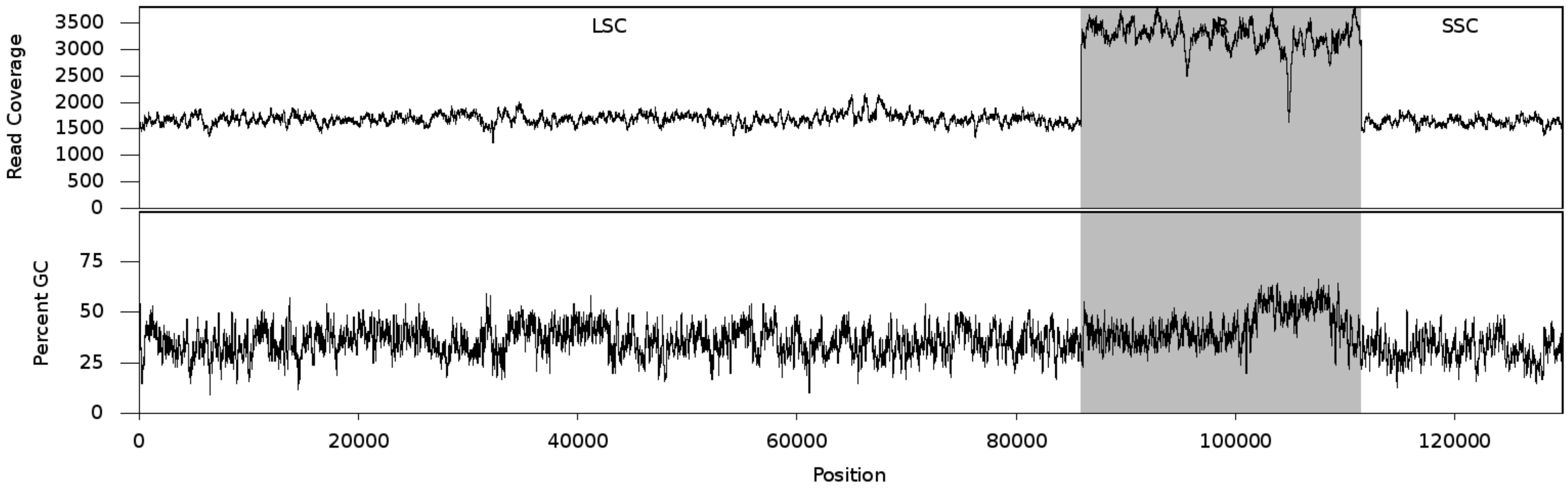

Solanum goniocalyx PI 195188

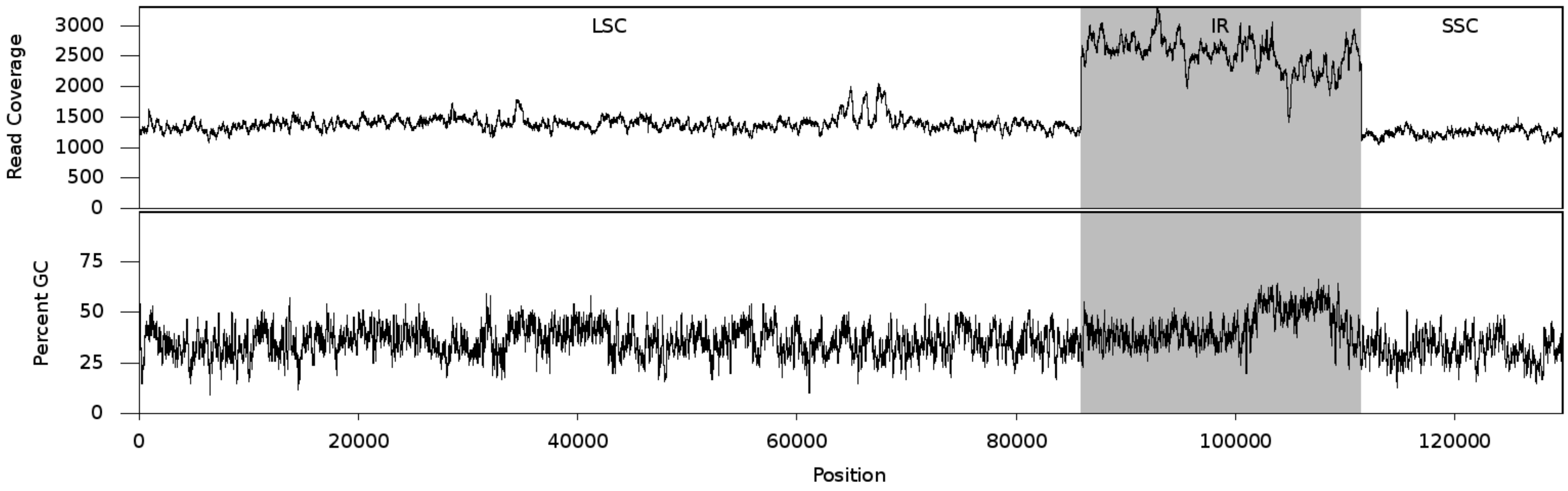

Solanum goniocalyx PI 195214

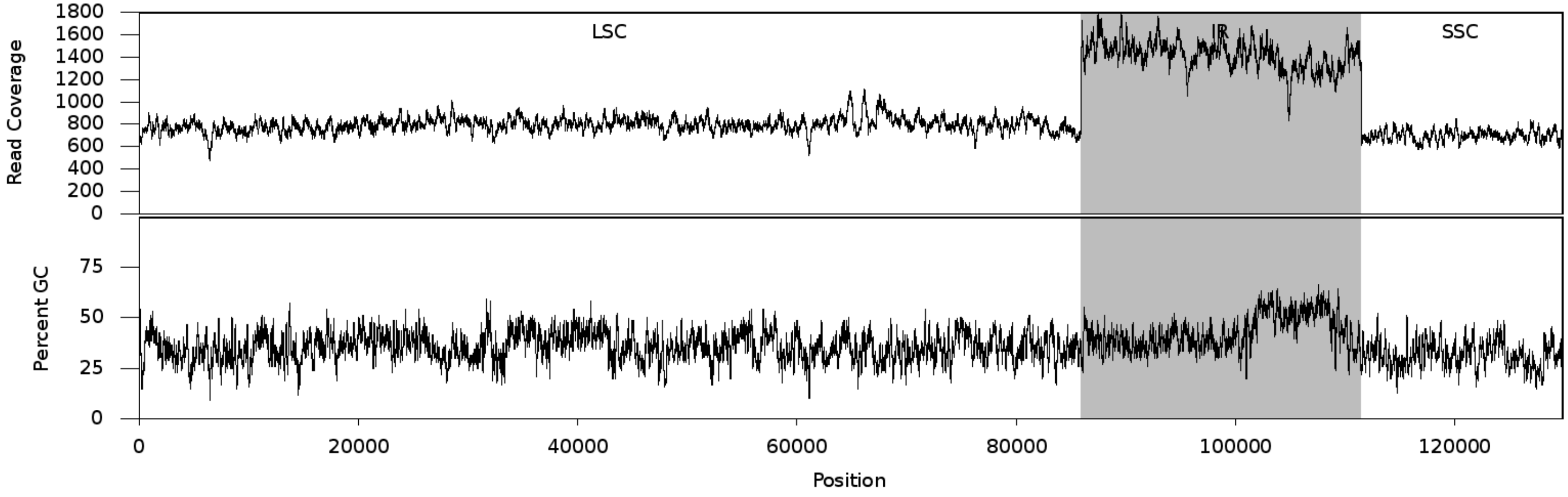

Solanum goniocalyx PI 458393

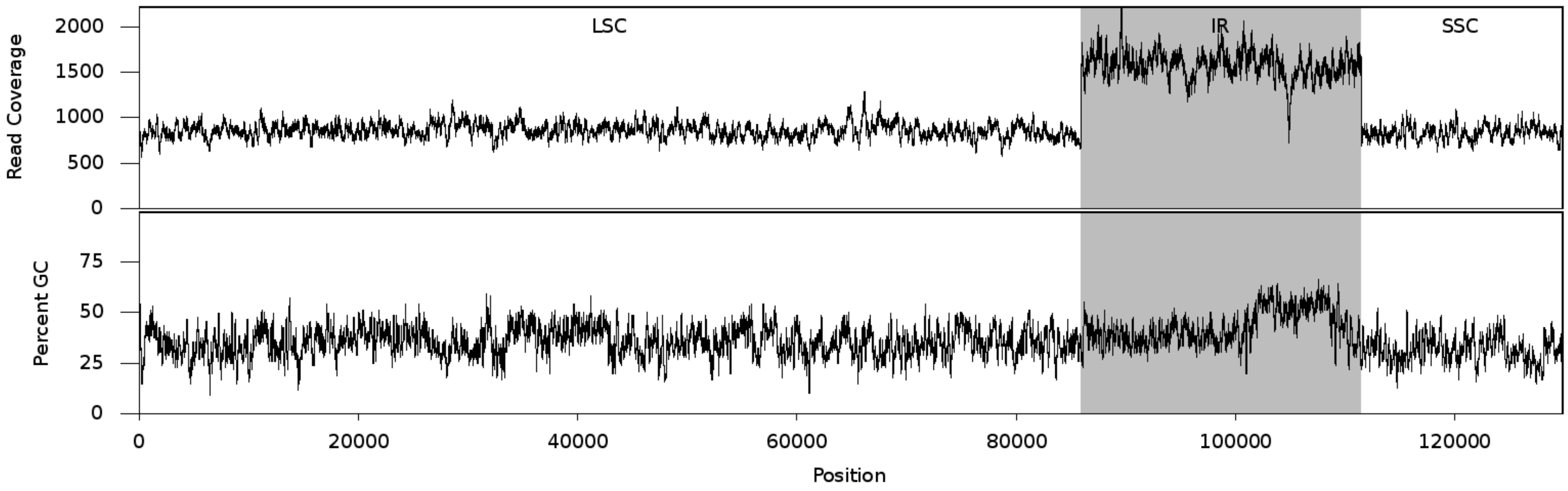

Solanum gourlayi PI 472911

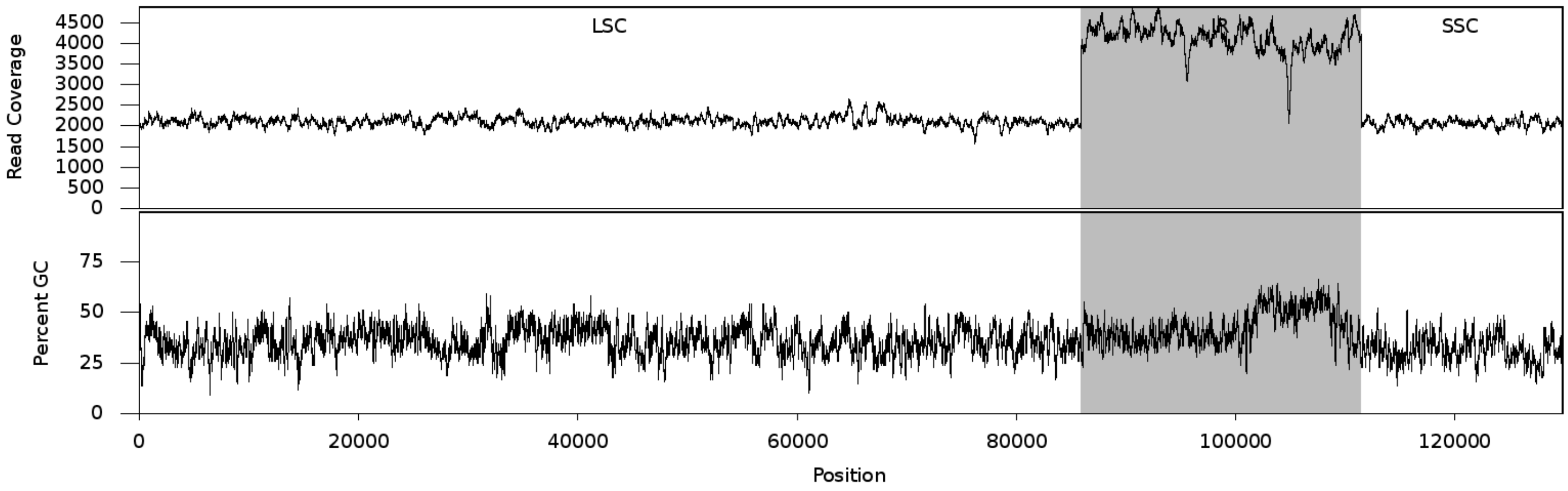

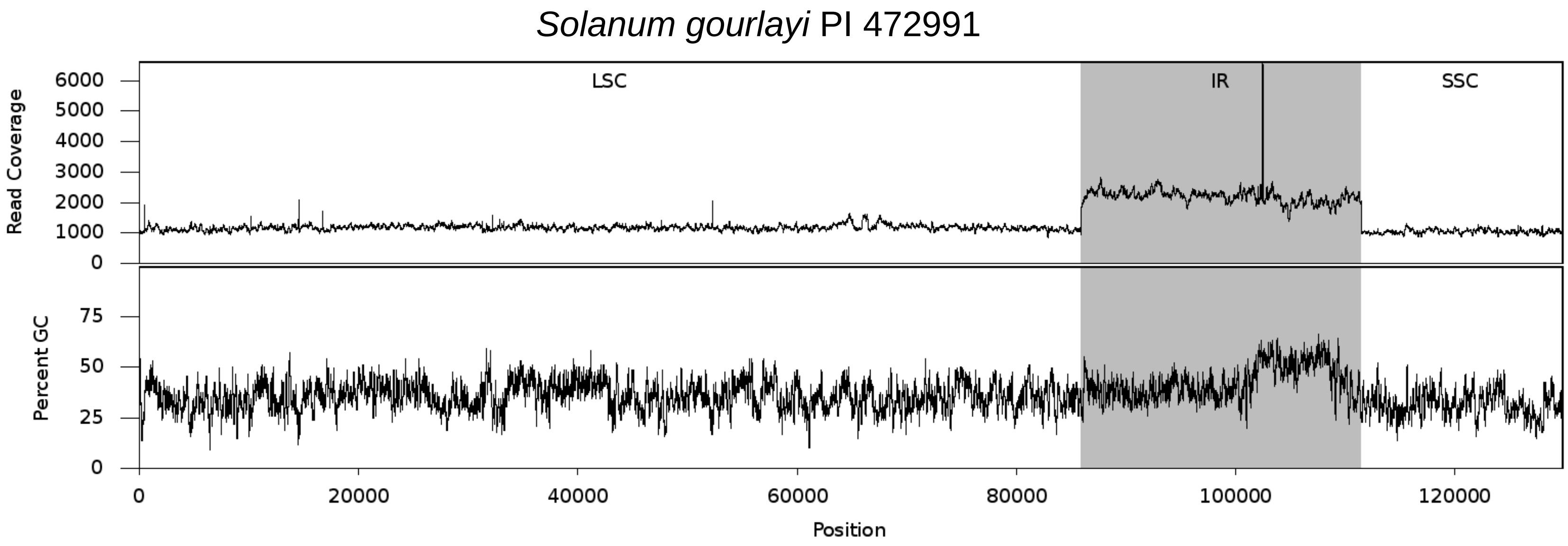
Solanum gourlayi PI 472991
LSC
IR
SSC
Read Coverage
6000
5000
4000
3000
2000
1000
0
Percent GC
75
50
25
0
0
20000
40000
60000
80000
100000
120000
Position

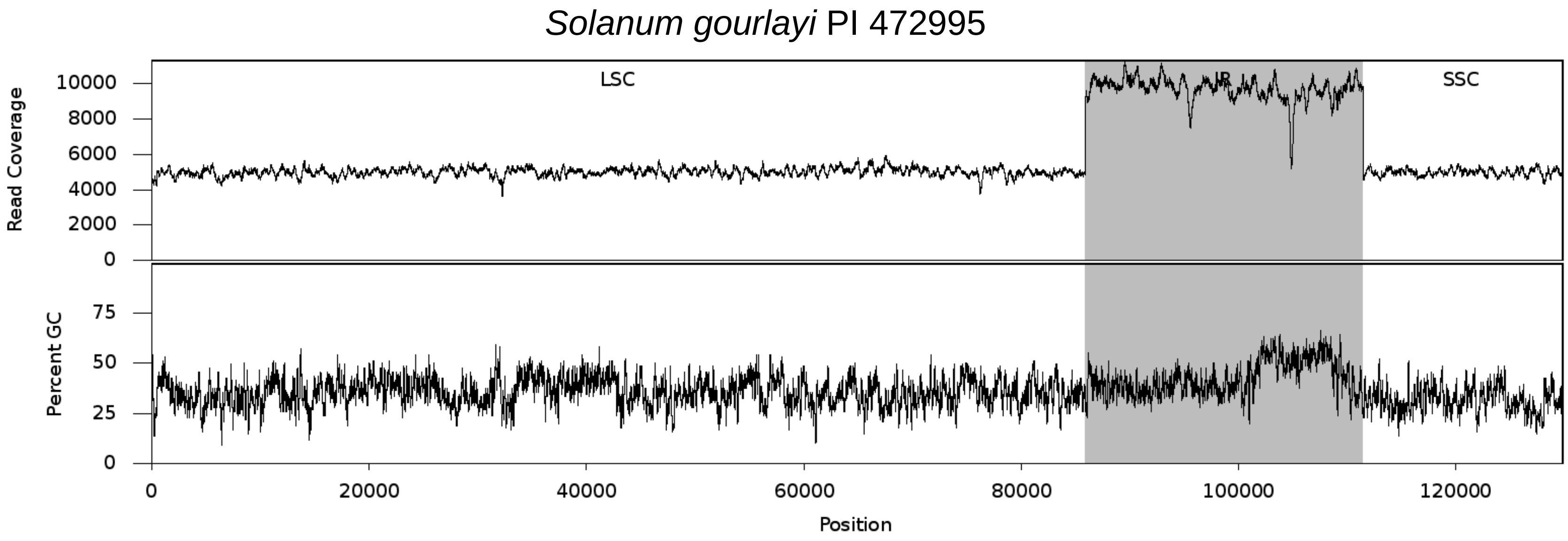
Solanum gourlayi PI 472995
LSC
IR
SSC
Read Coverage
10000
8000
6000
4000
2000
0
Percent GC
75
50
25
0
0
20000
40000
60000
80000
100000
120000
Position

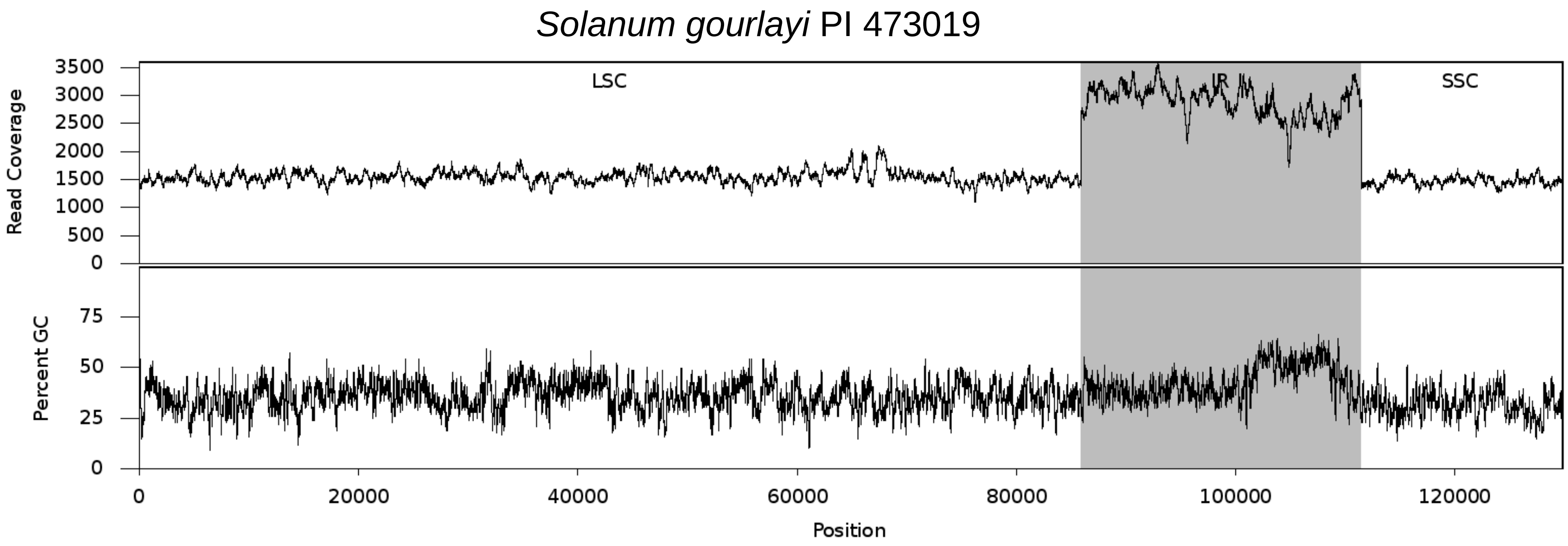
Solanum gourlayi PI 473019
Read Coverage
Percent GC
Position
LSC
IR
SSC
3500
3000
2500
2000
1500
1000
500
0
75
50
25
0
20000
40000
60000
80000
100000
120000

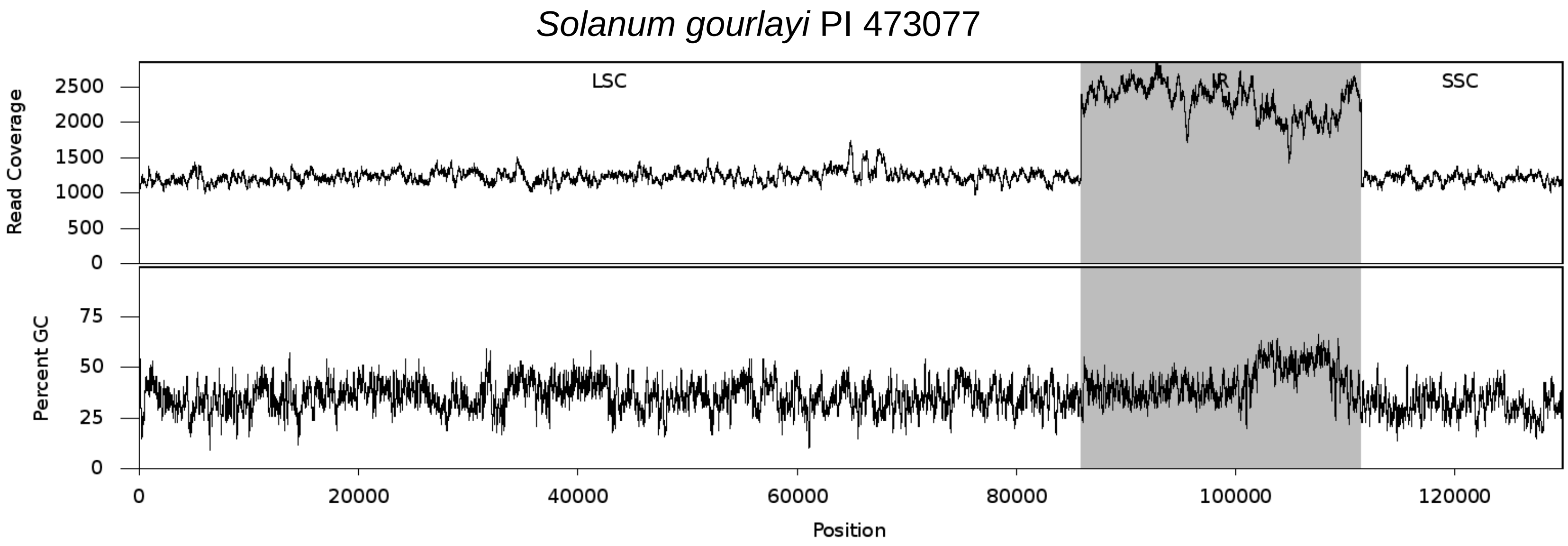
Solanum gourlayi PI 473077
Read Coverage
Percent GC
Position
LSC
IR
SSC
2500
2000
1500
1000
500
0
75
50
25
0
20000
40000
60000
80000
100000
120000

Solanum gourlayi PI 473106

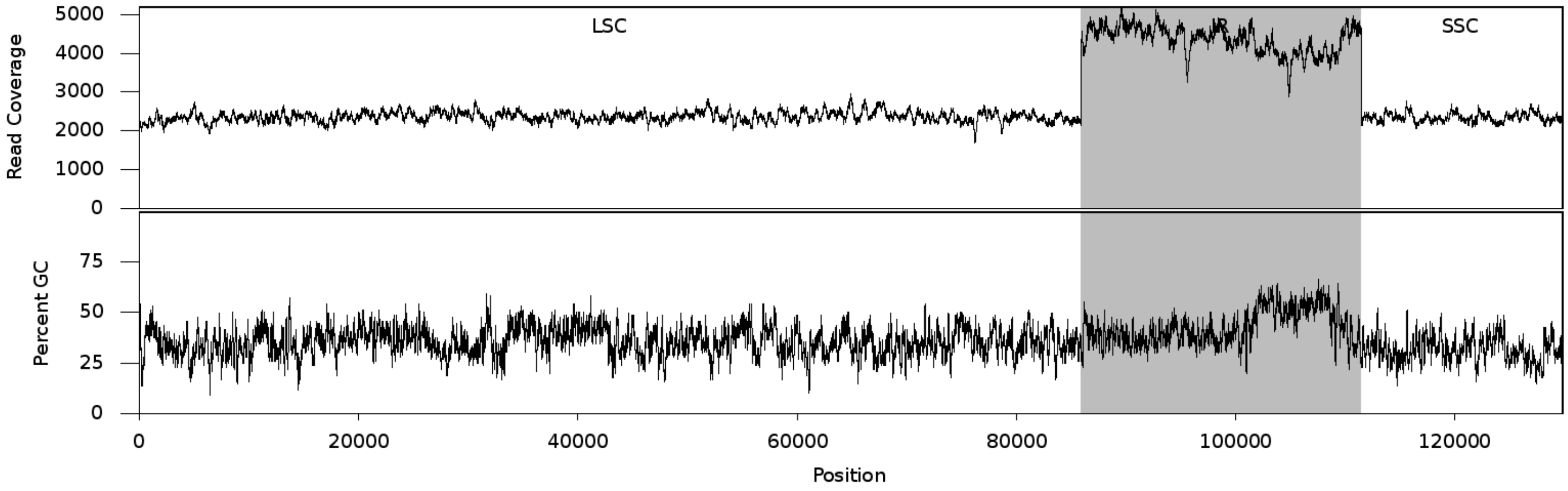

Solanum gourlayi PI 500022

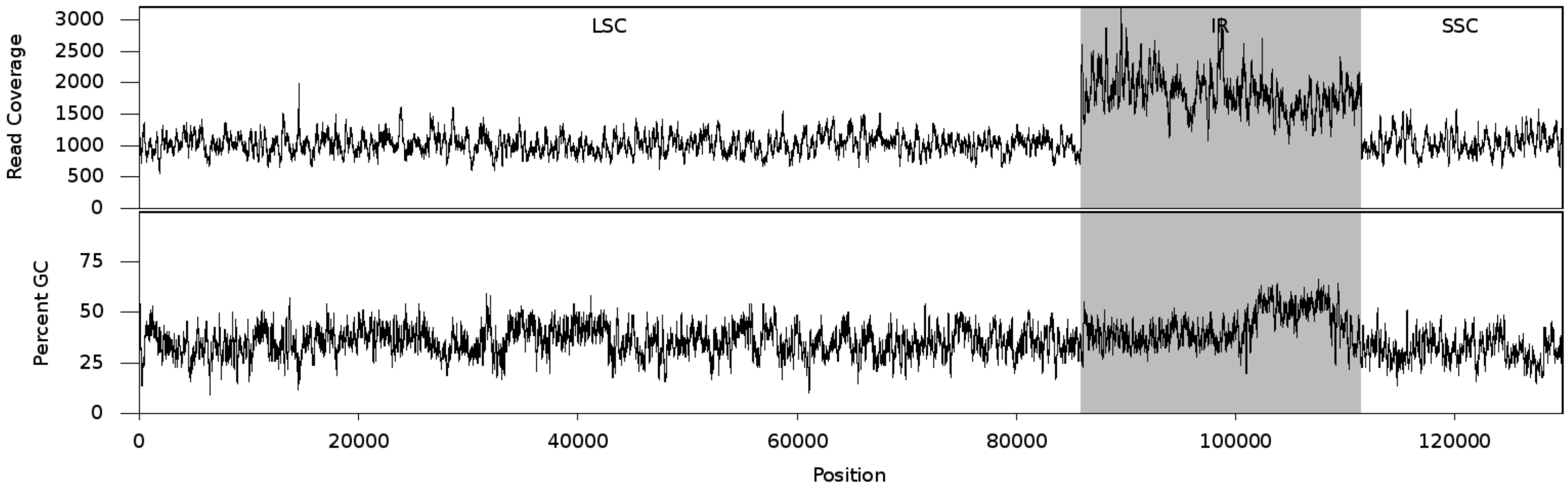

Solanum gourlayi PI 537026

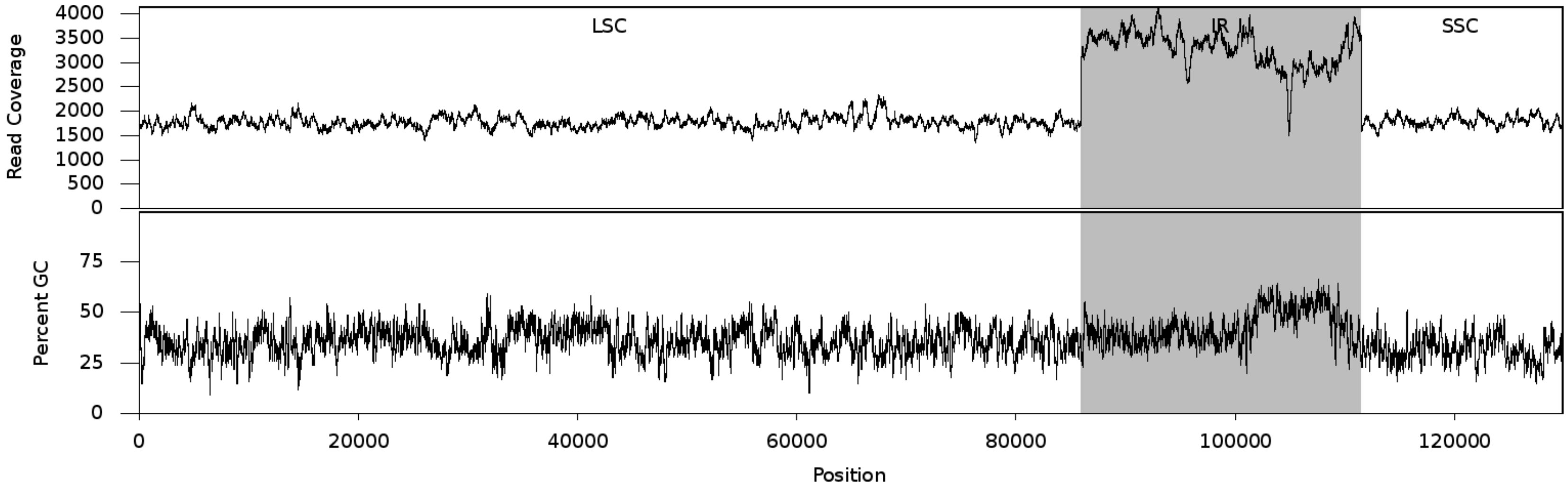

Solanum gourlayi PI 545865

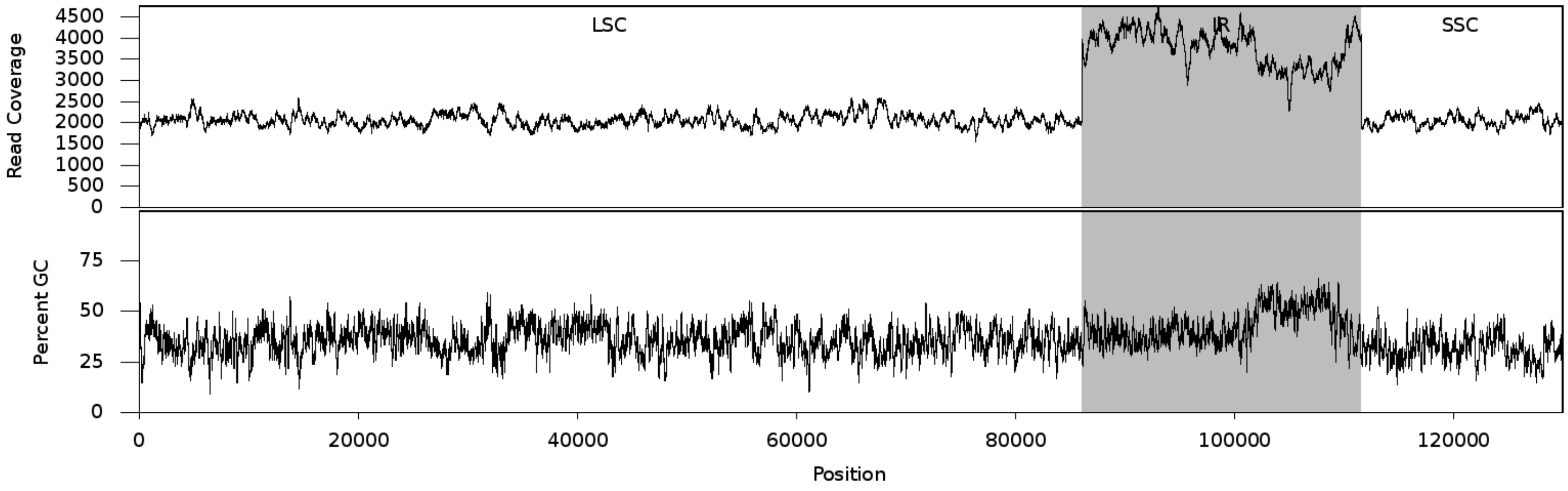

Solanum gourlayi PI 545975

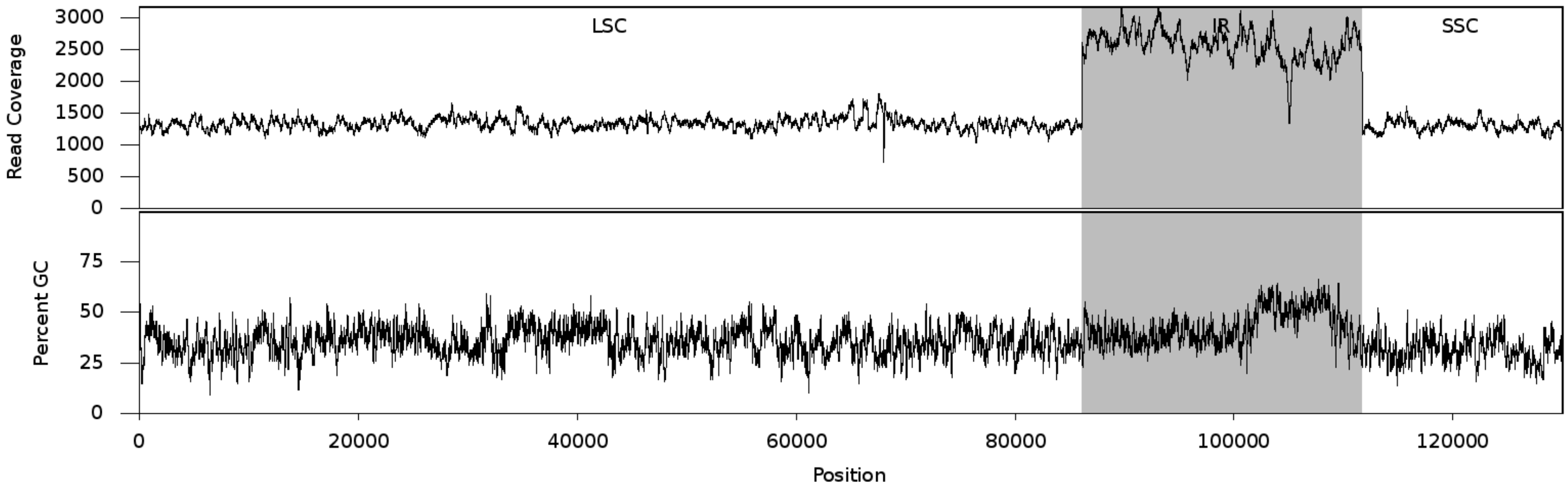

Solanum gourlayi PI 545978

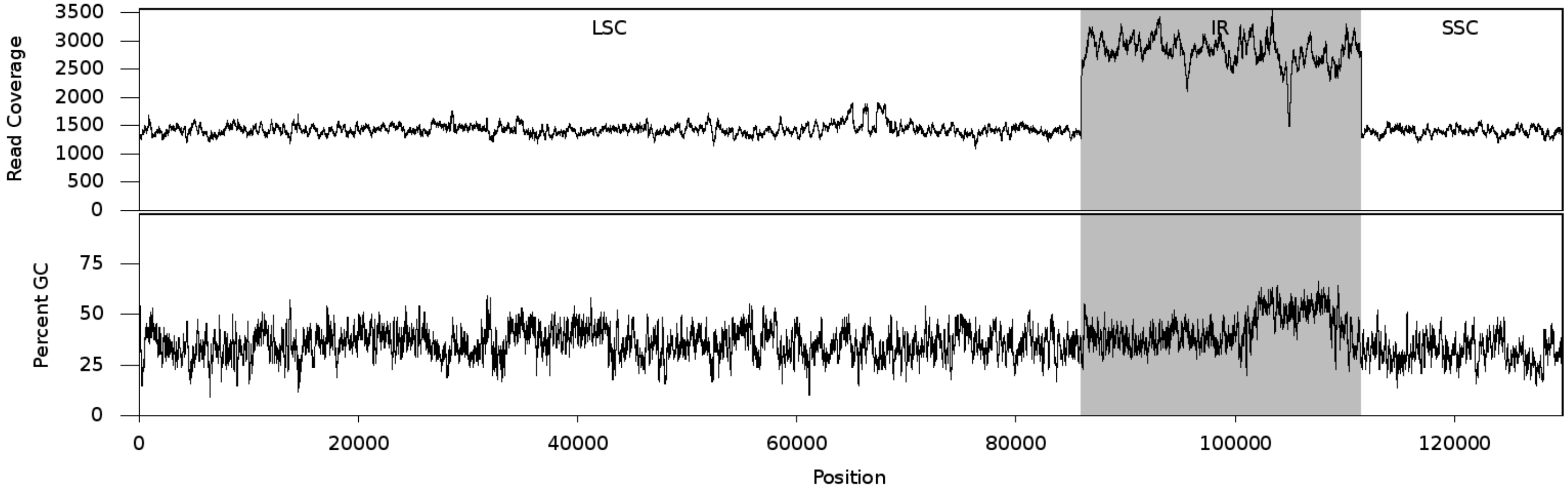

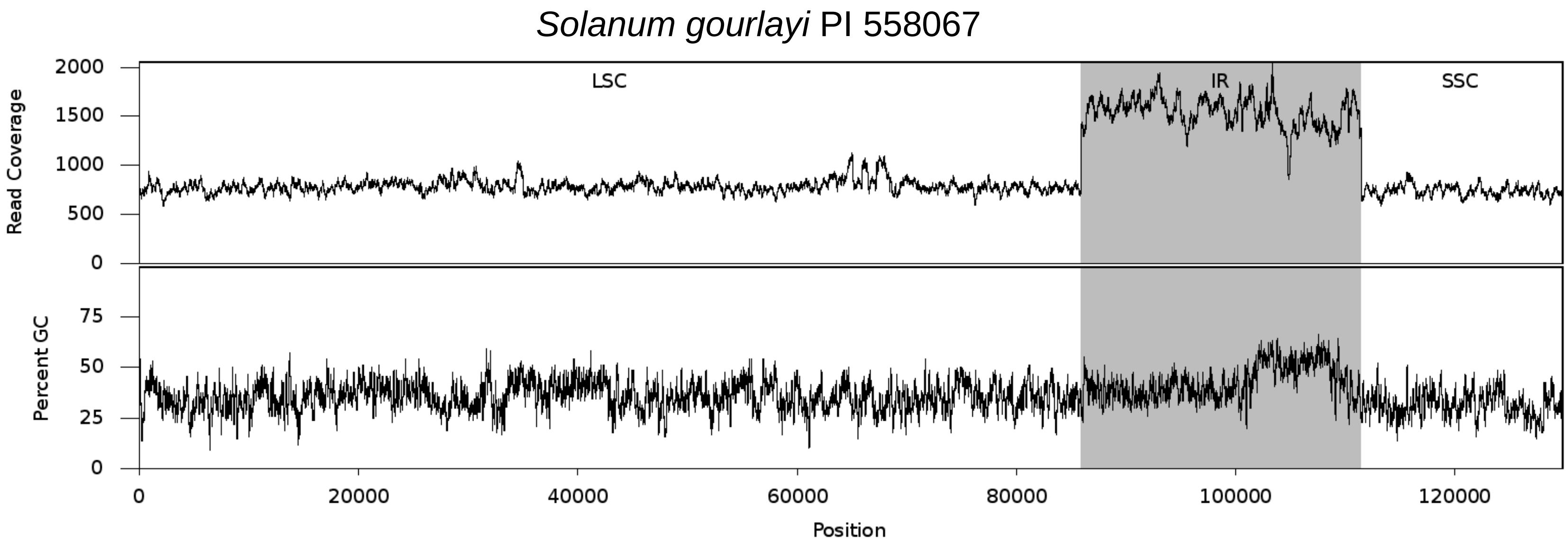
Solanum gourlayi PI 558067
Read Coverage
Percent GC
Position
LSC
IR
SSC
2000
1500
1000
500
0
75
50
25
0
20000
40000
60000
80000
100000
120000

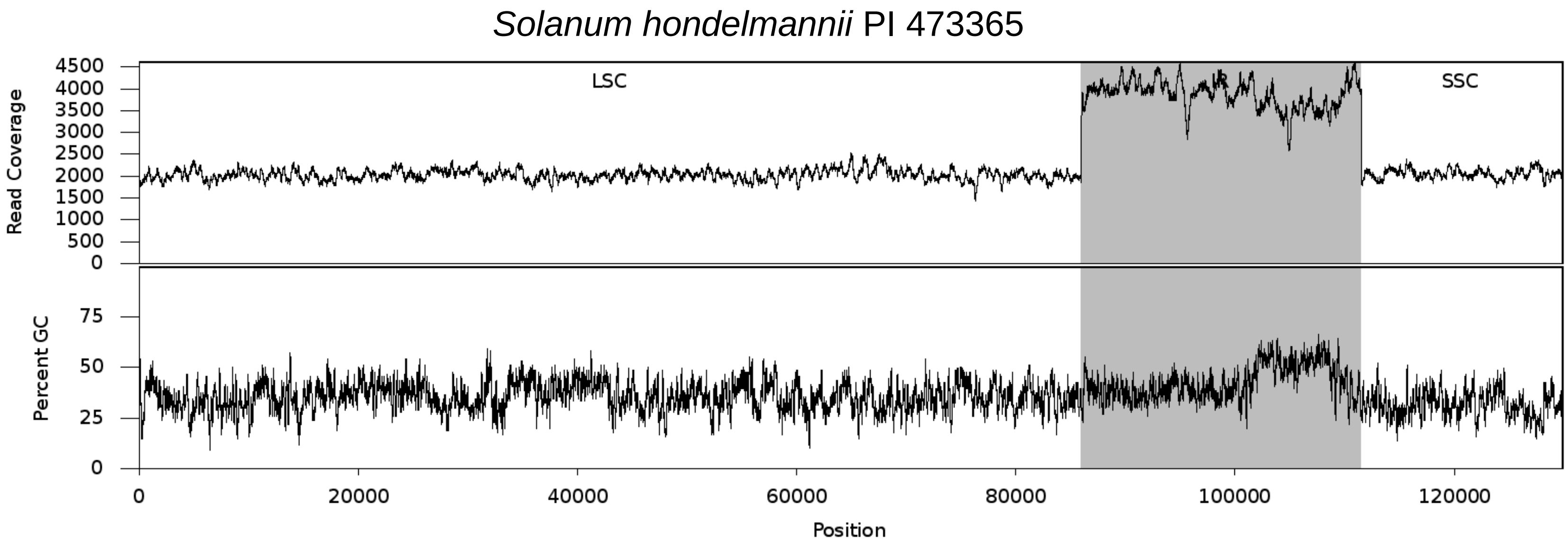
Solanum hondelmannii PI 473365
Read Coverage
Percent GC
Position
LSC
IR
SSC
4500
4000
3500
3000
2500
2000
1500
1000
500
0
75
50
25
0
20000
40000
60000
80000
100000
120000

Solanum hondelmannii PI 498067

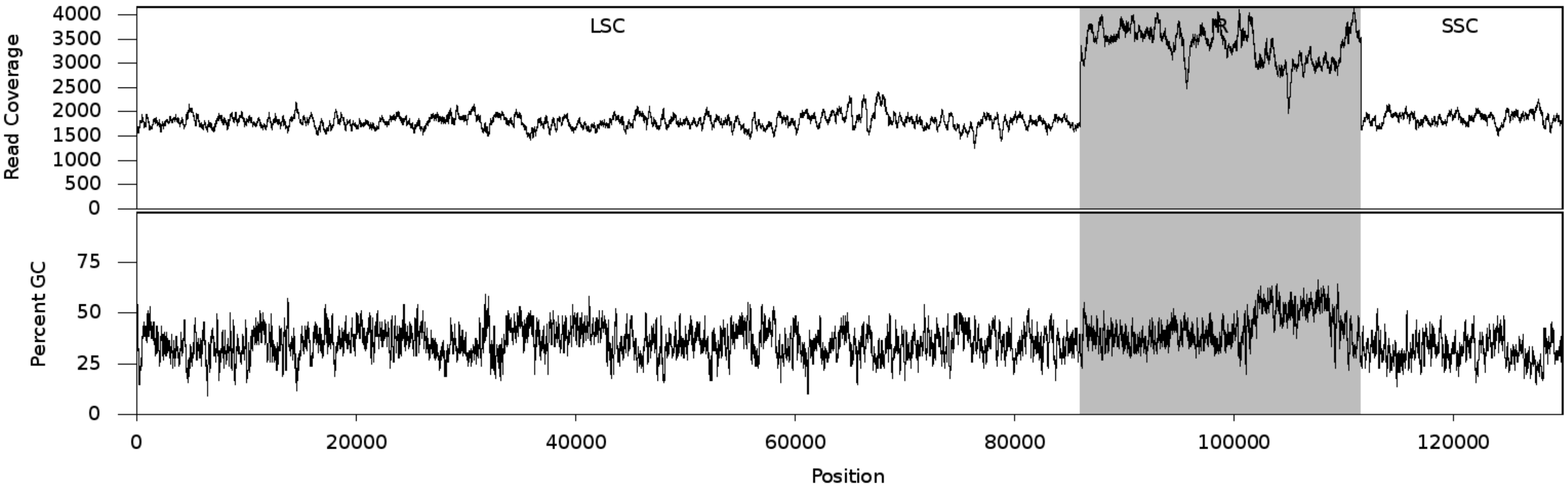

Solanum hondelmannii PI 498071

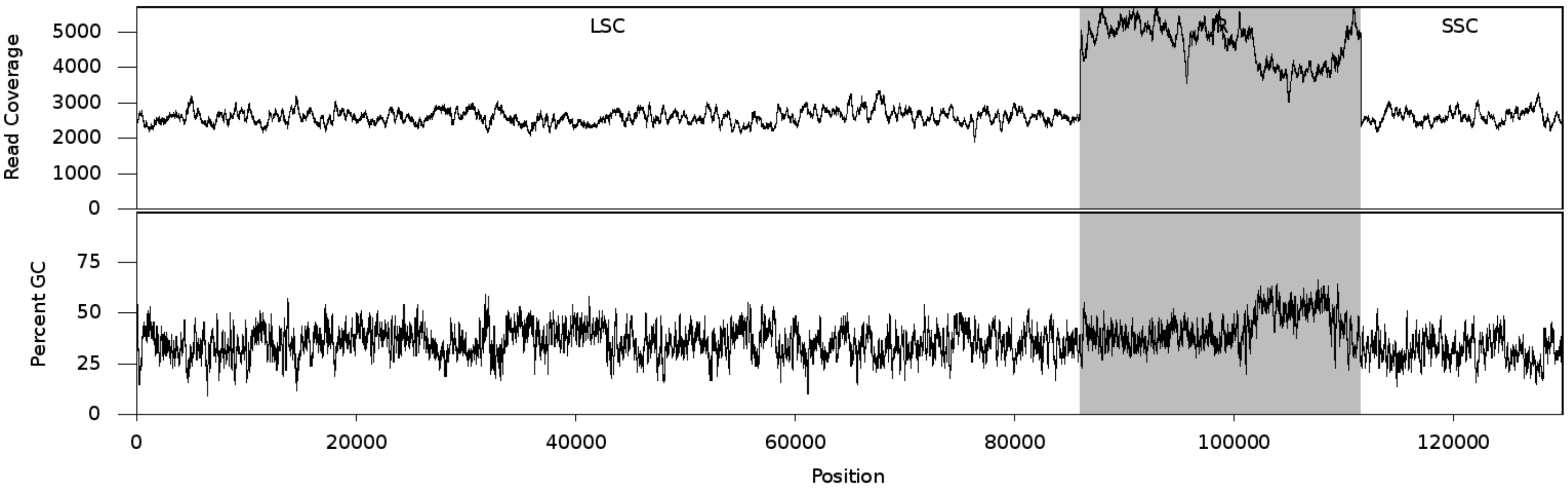

Solanum hondelmannii PI 545879

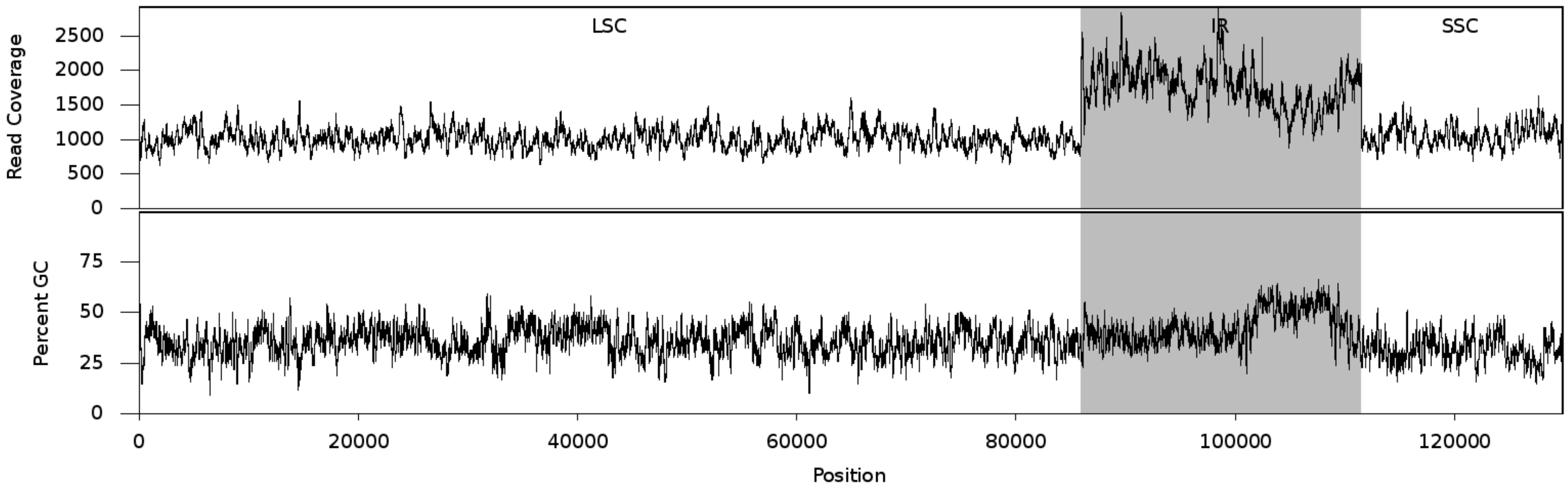

Solanum hypacrarthrum PI 473477

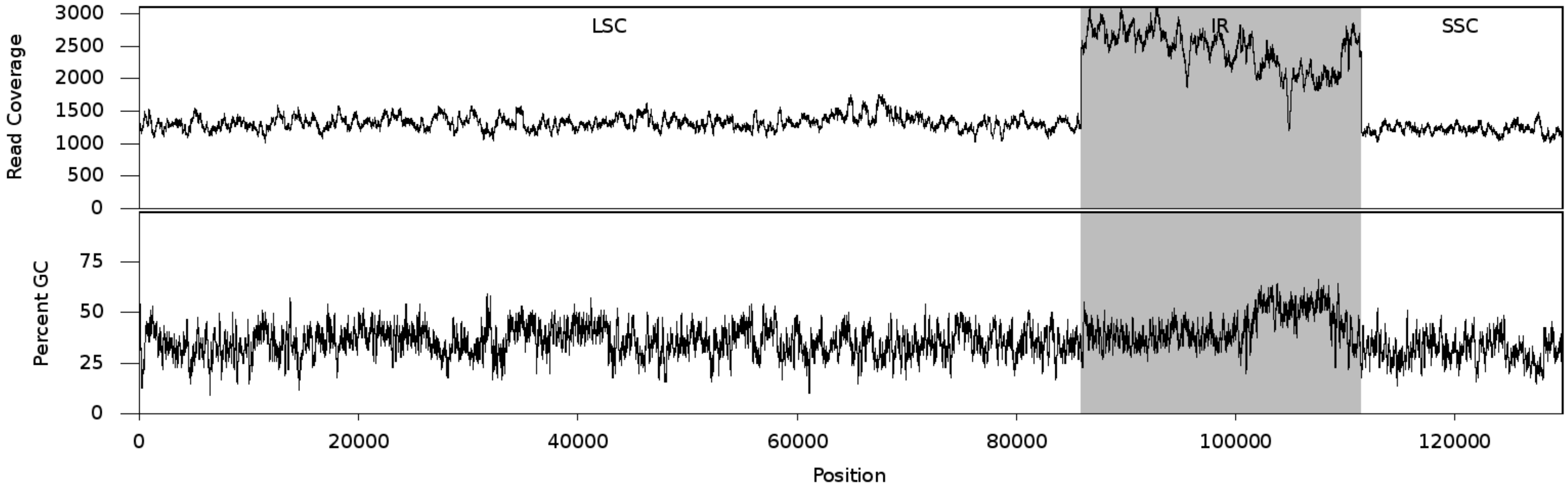

Solanum incamayoense PI 473060

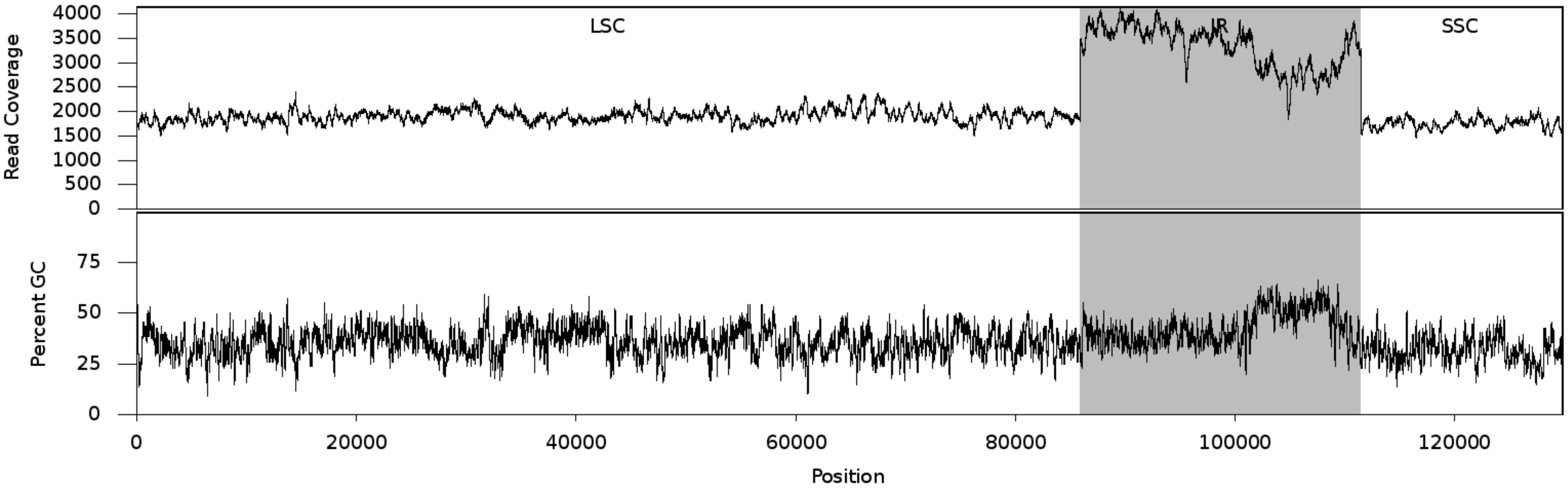

Solanum incamayoense PI 473067

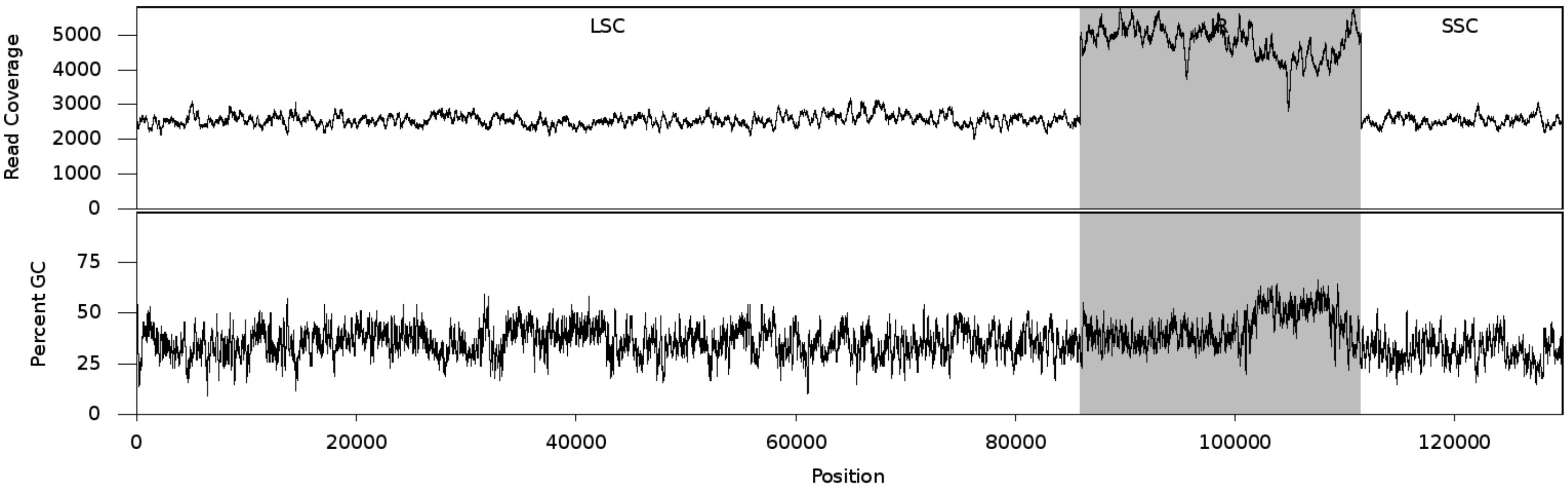

Solanum incamayoense PI 473069

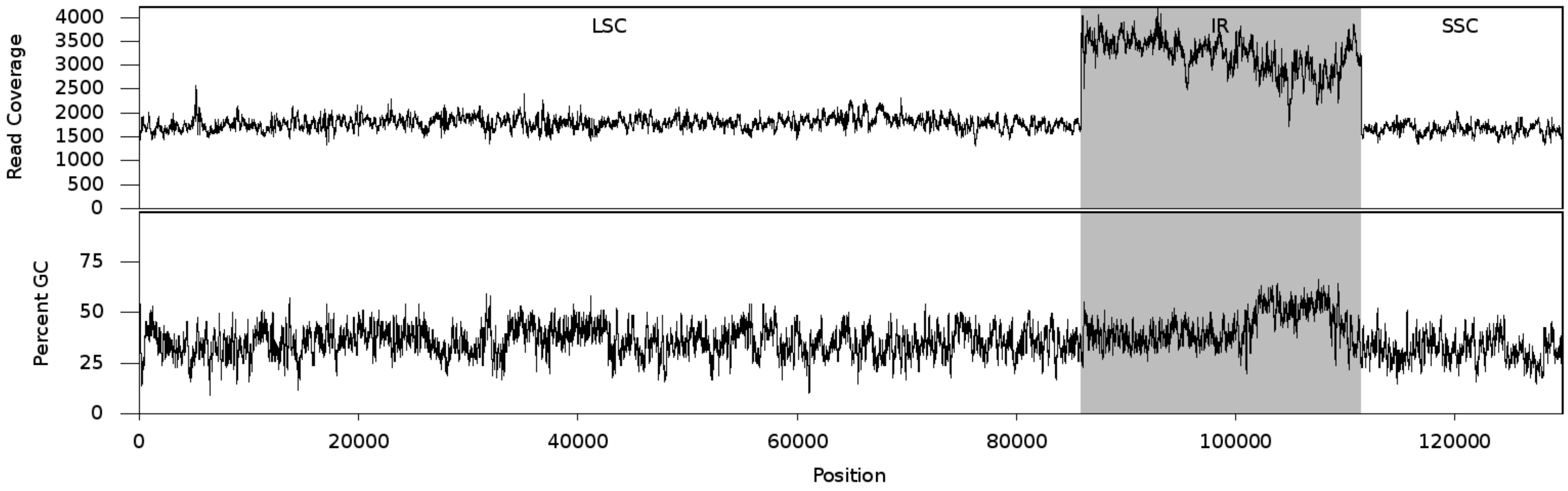

Solanum incamayoense PI 473070

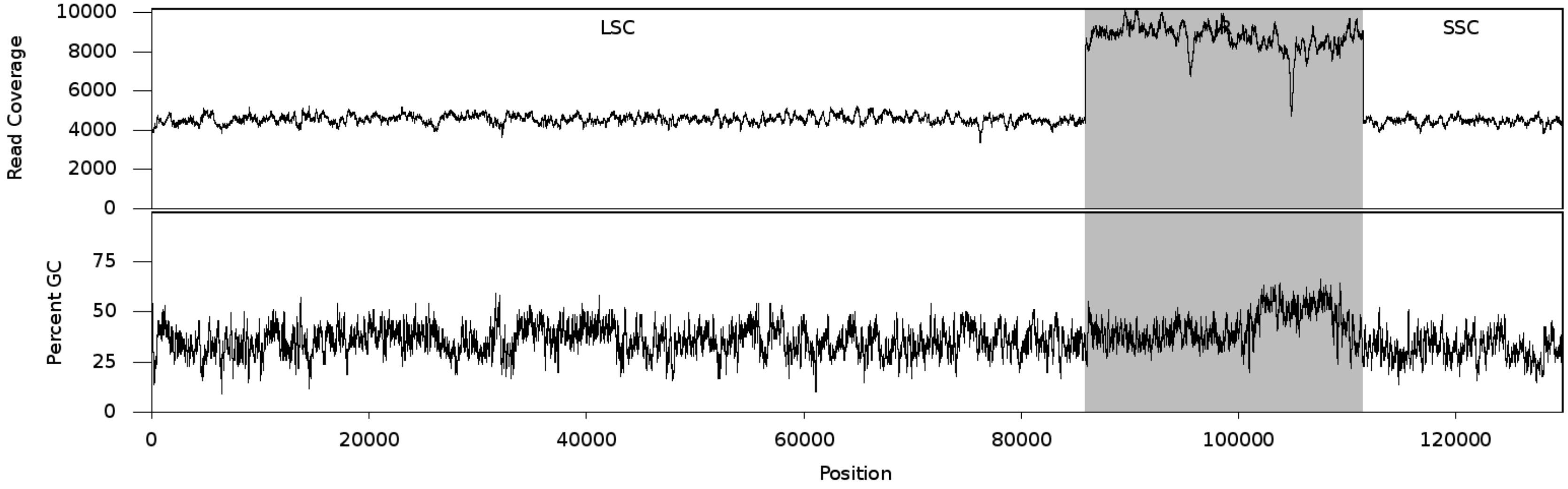

Solanum incamayoense PI 500048

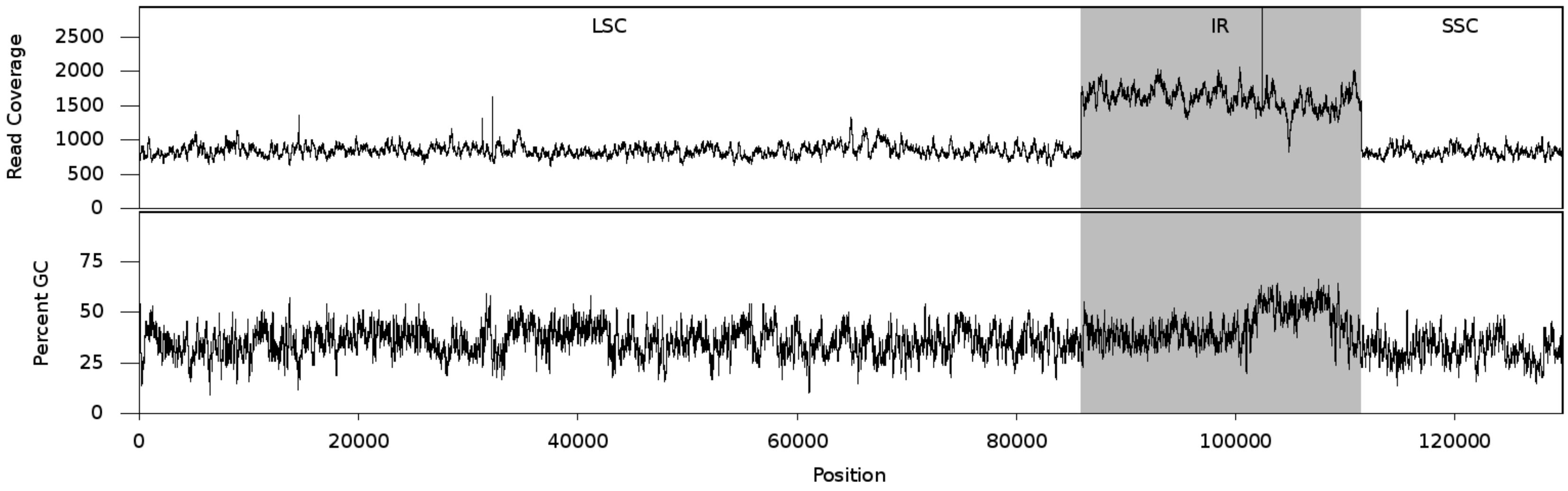

Solanum jamesii PI 641944

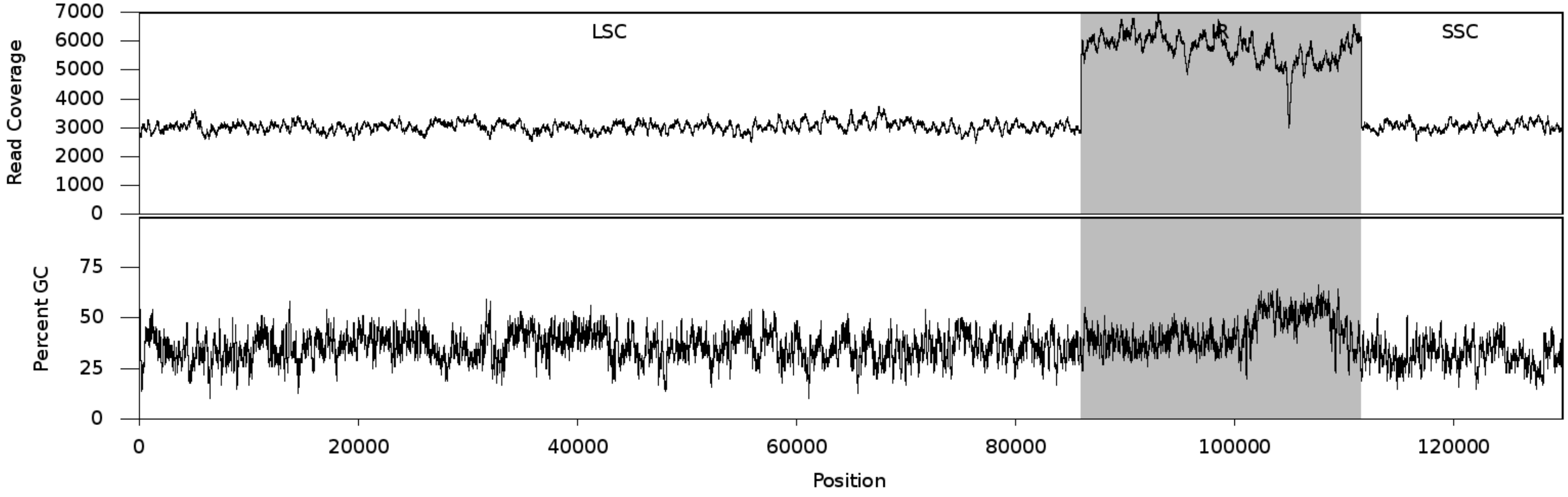

Solanum jamesii PI 664024

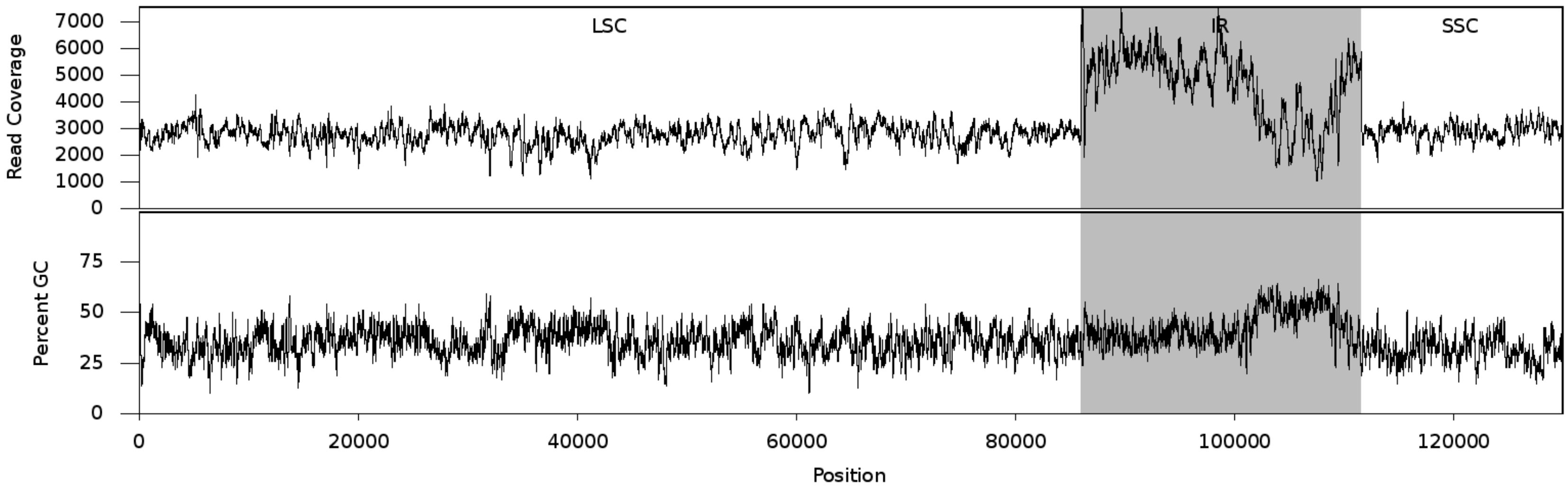

Solanum kurtzianum PI 320327

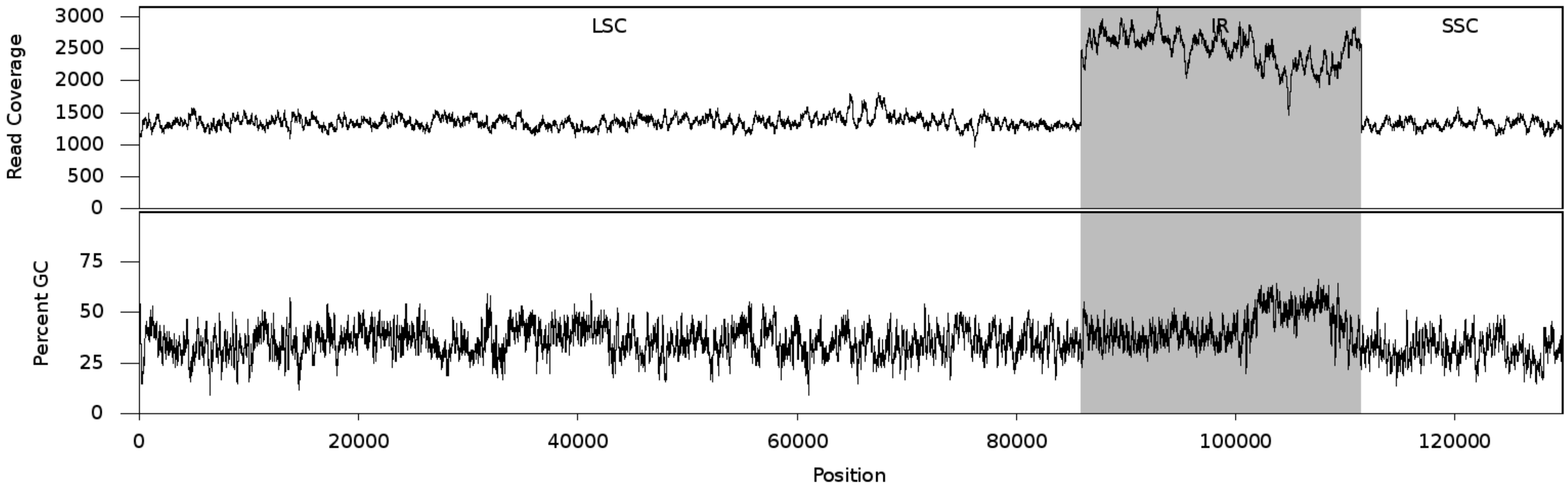

Solanum kurtzianum PI 472924

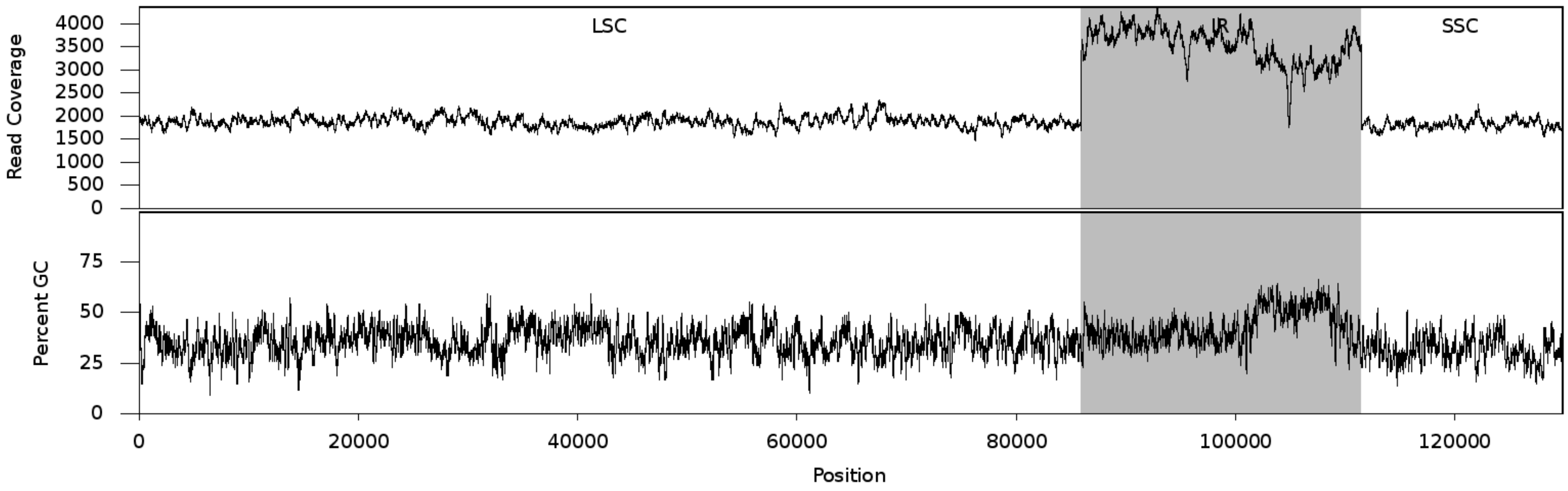

Solanum kurtzianum PI 472936

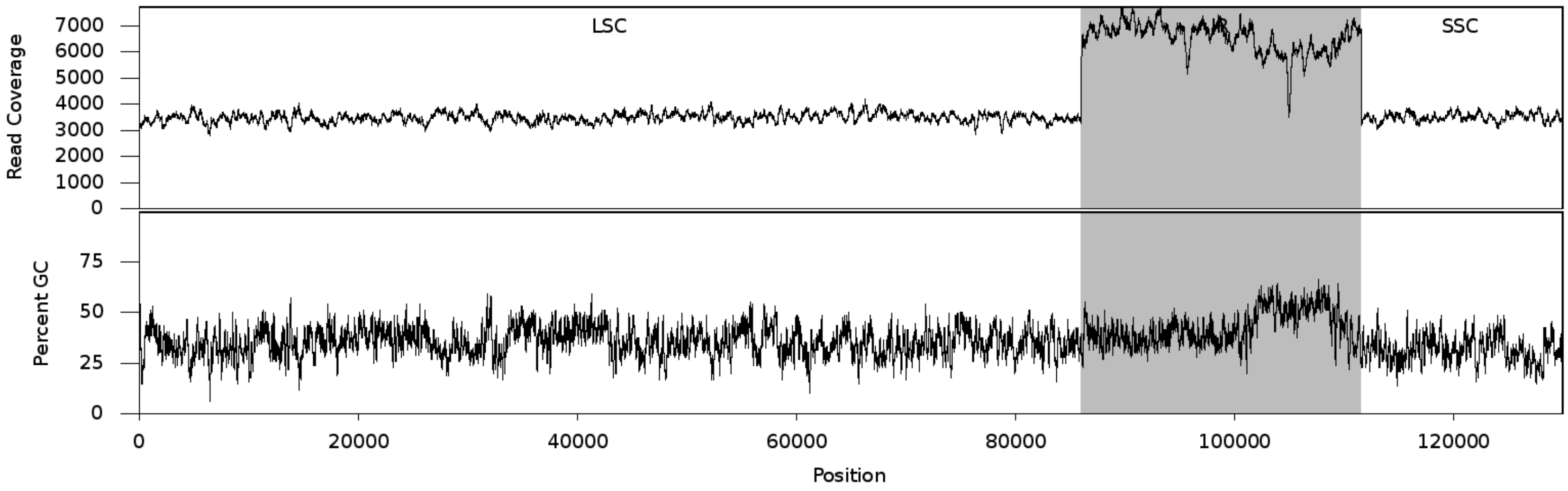

Solanum kurtzianum PI 472948

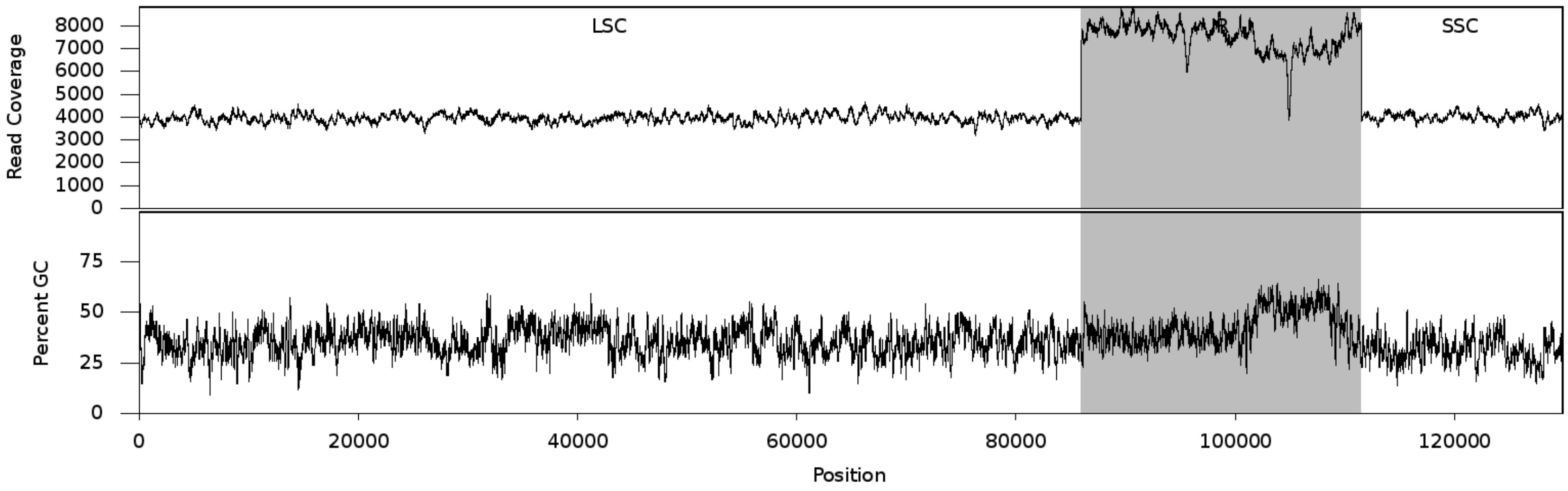

Solanum kurtzianum PI 472952

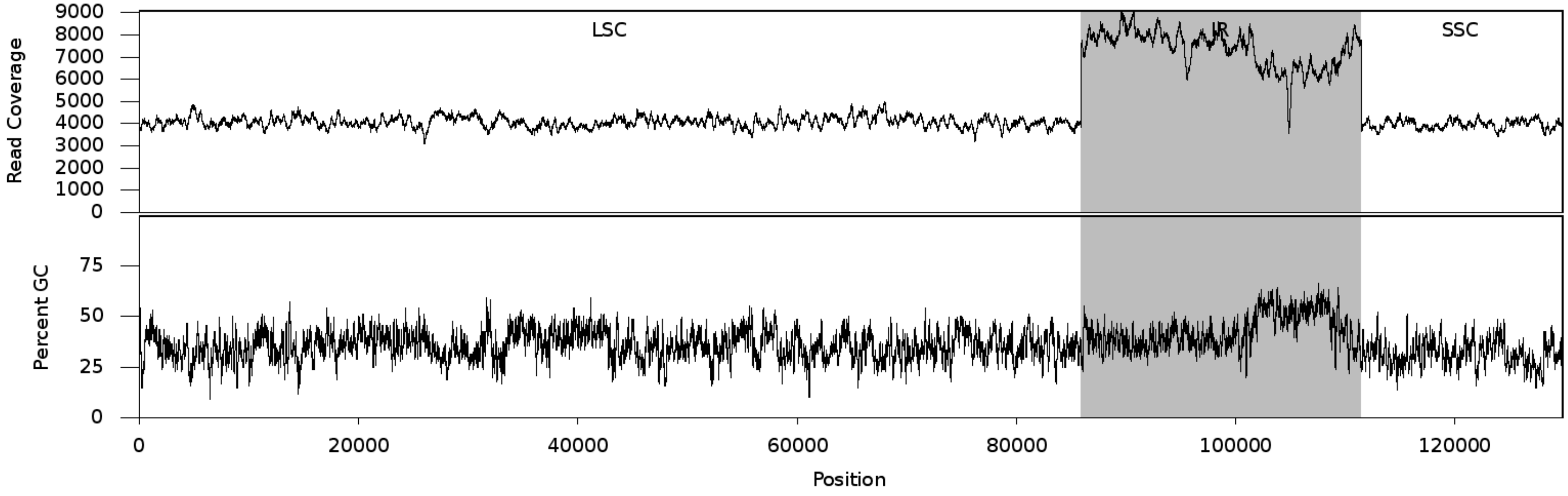

Solanum kurtzianum PI 558185

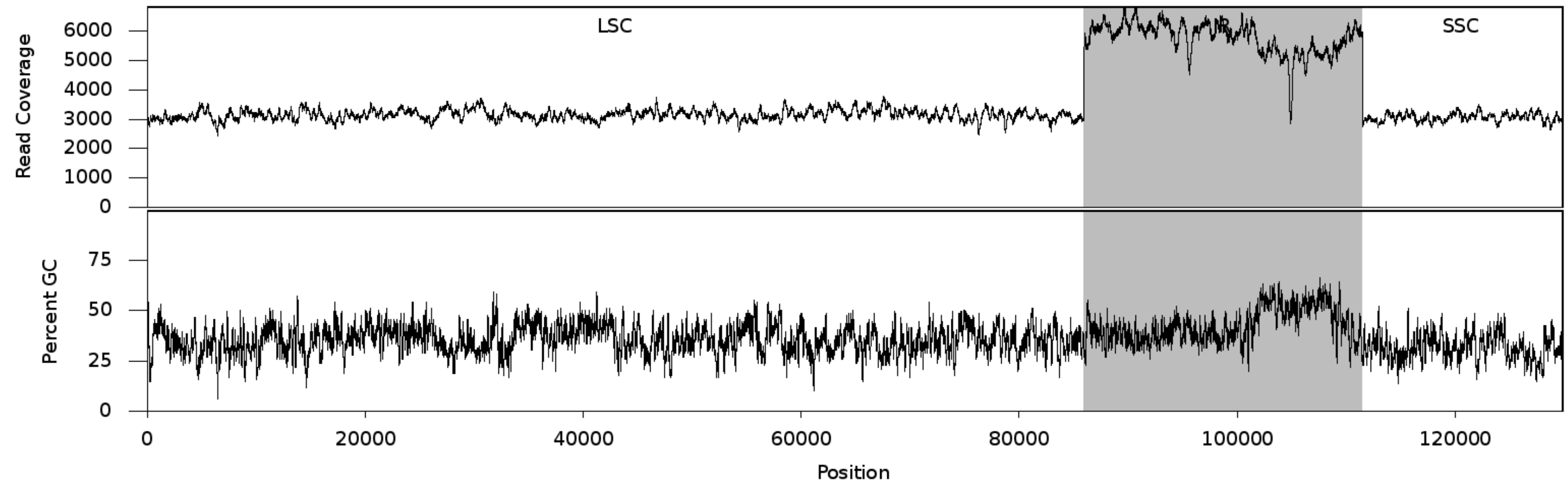

Solanum kurtzianum PI 558208

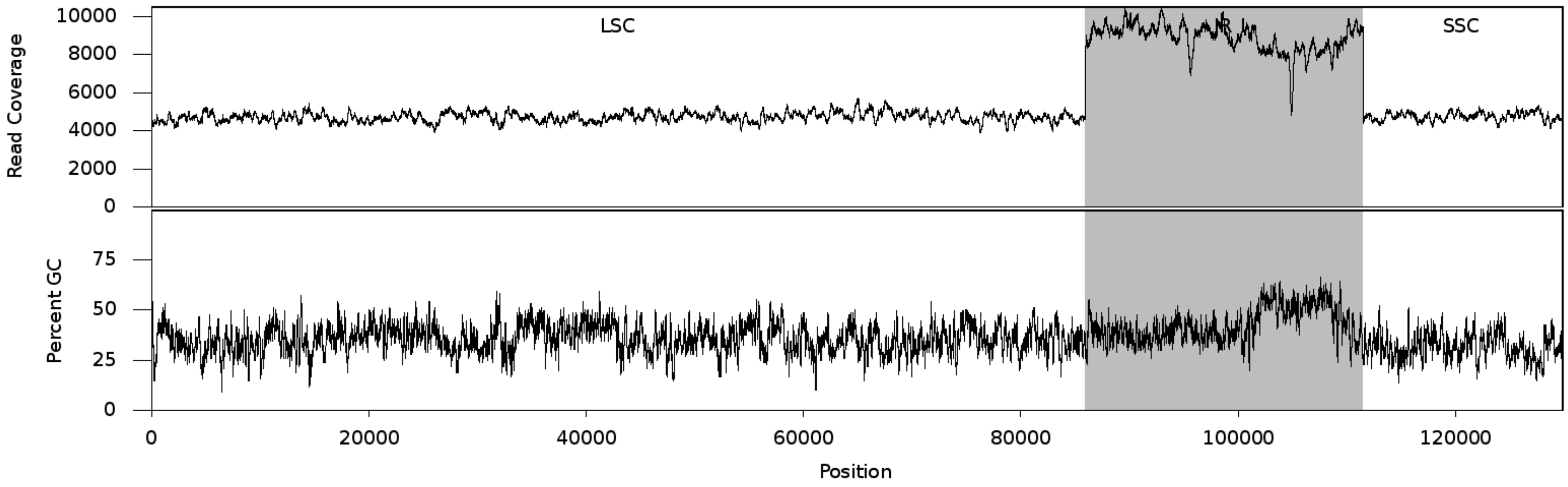

Solanum laxissimum PI 283088

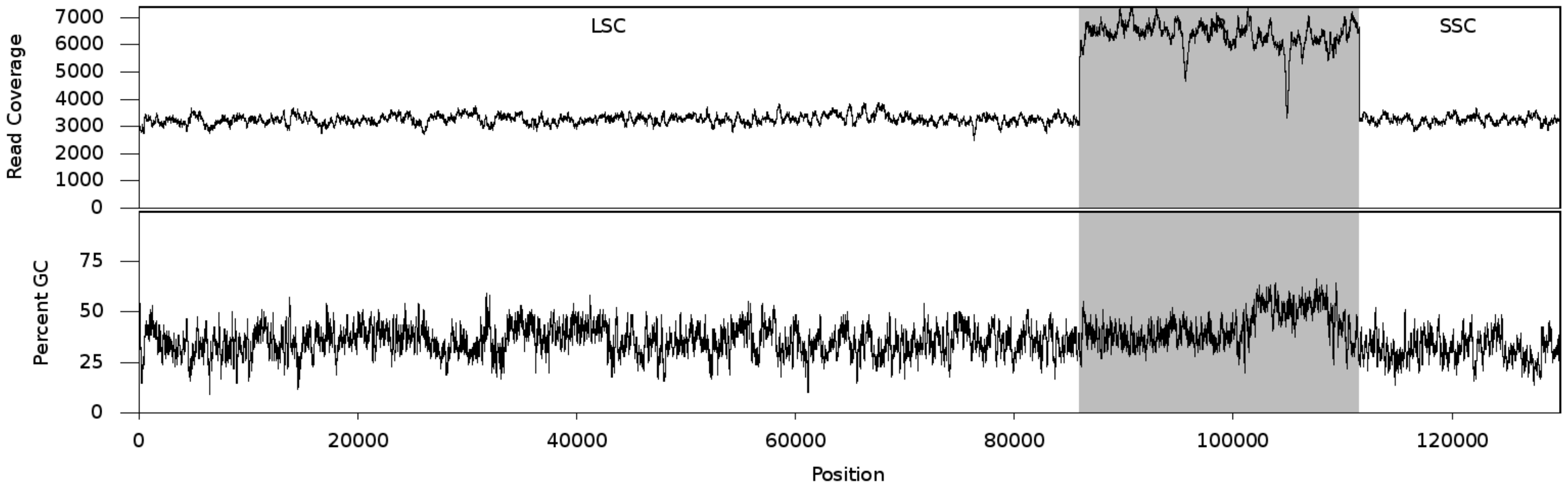

Solanum laxissimum PI 498252

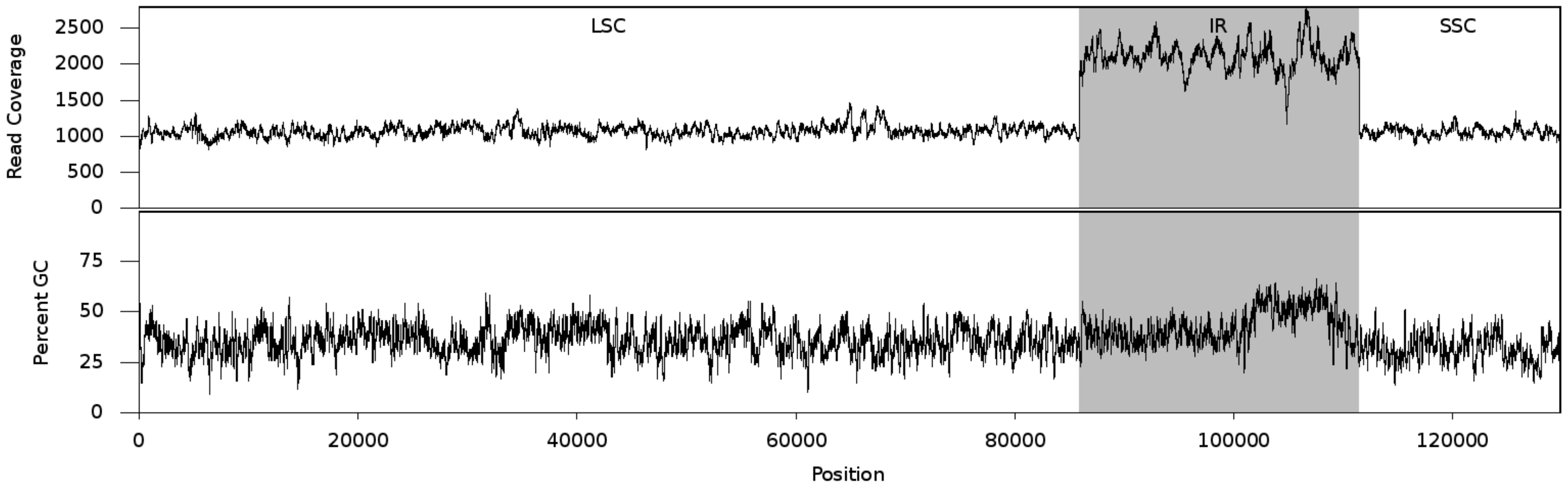

Solanum laxissimum PI 607887

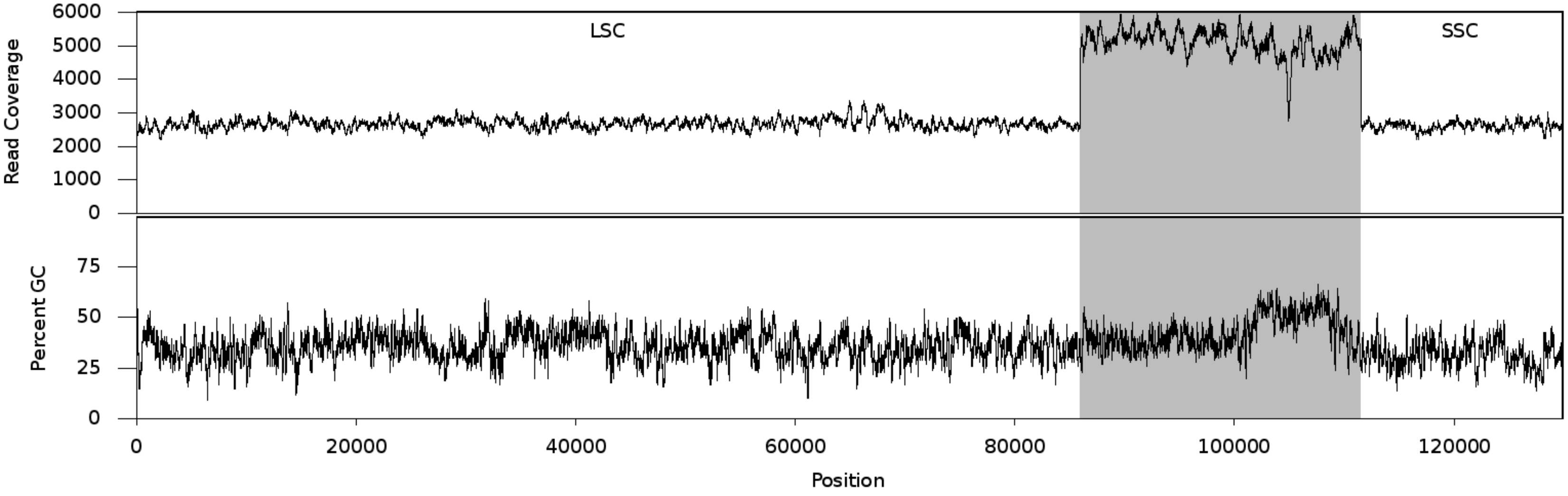

Solanum leptophyes PI 458378

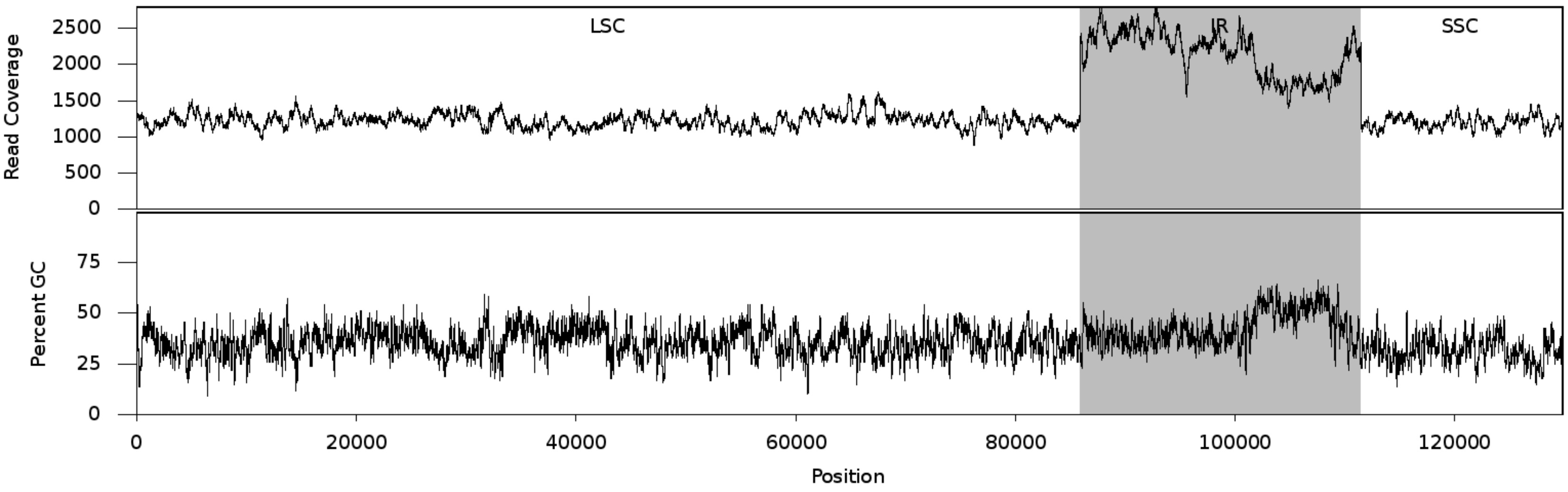

Solanum leptophyes PI 473342

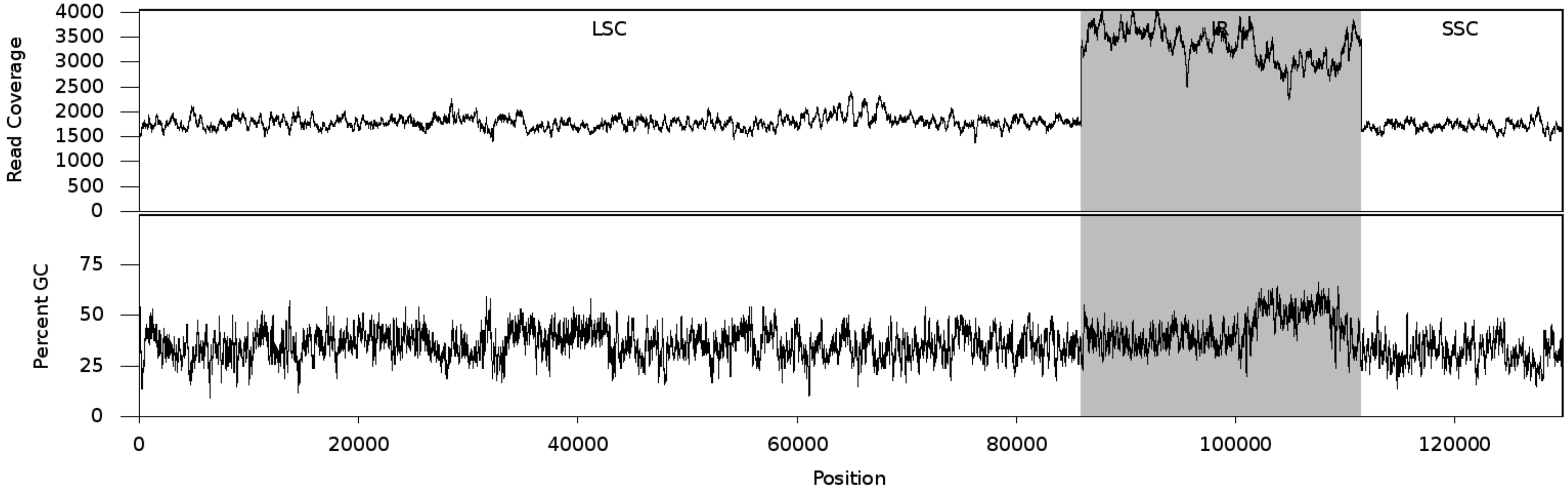

Solanum leptophyes PI 473446

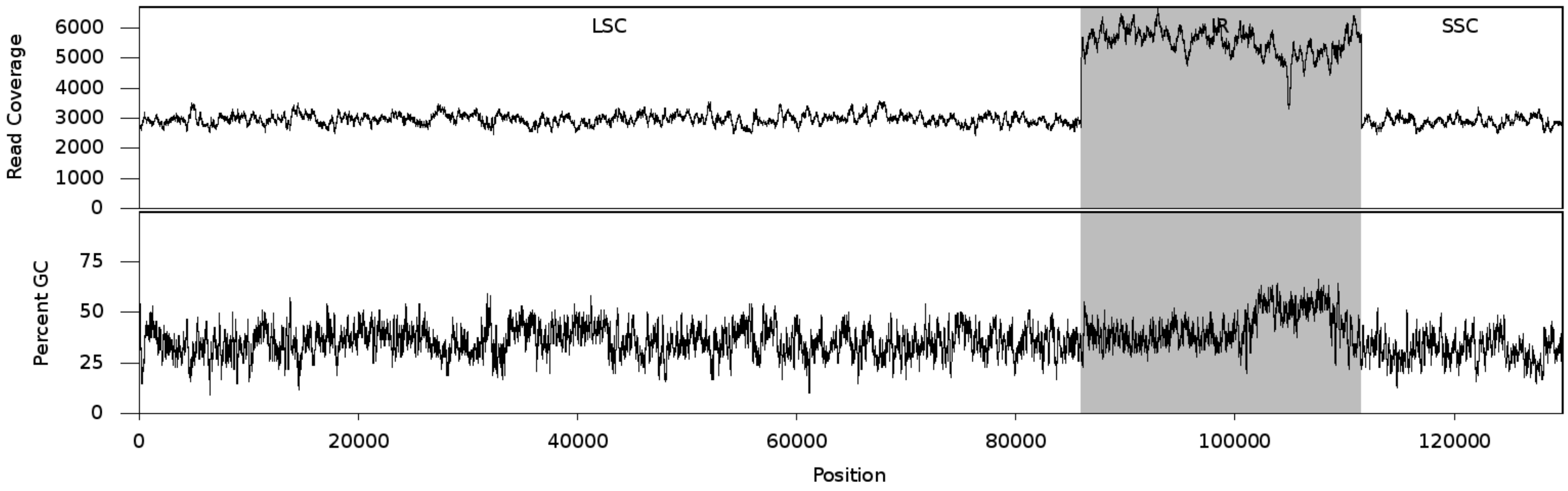

Solanum leptophyes PI 473451

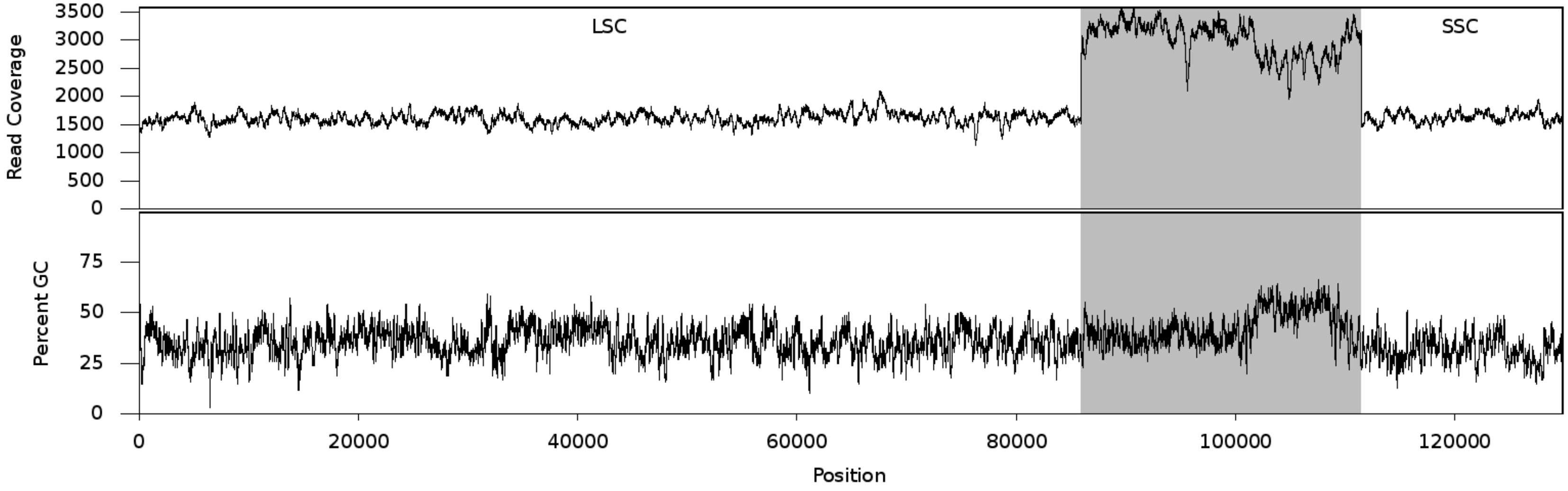

Solanum leptophyes PI 545985

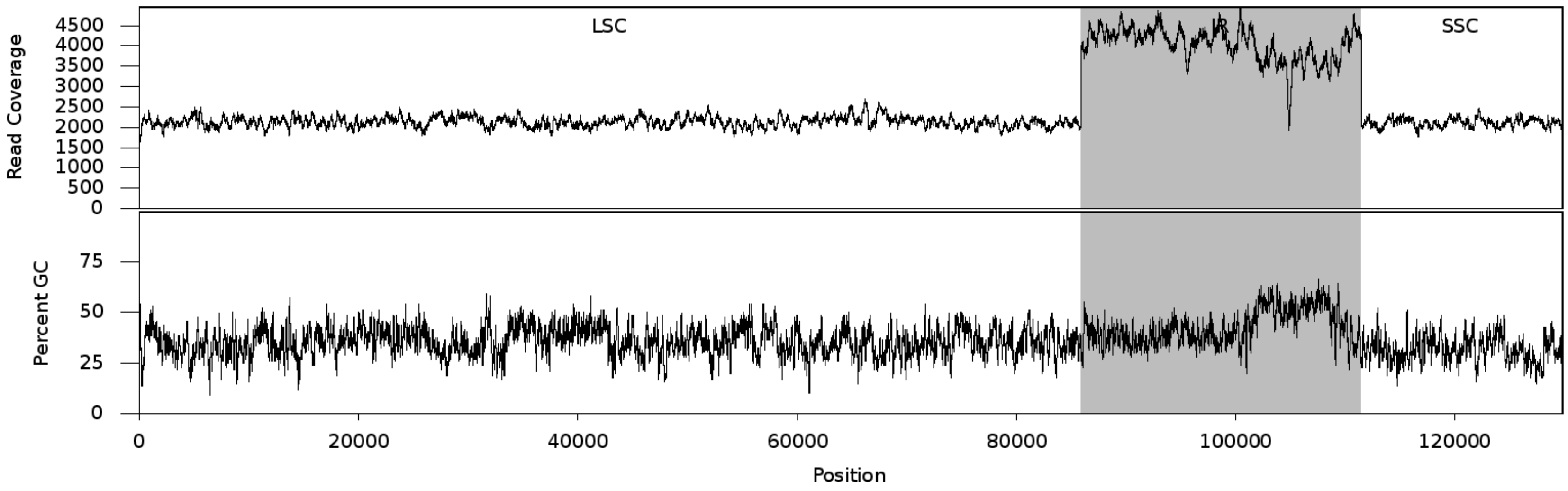

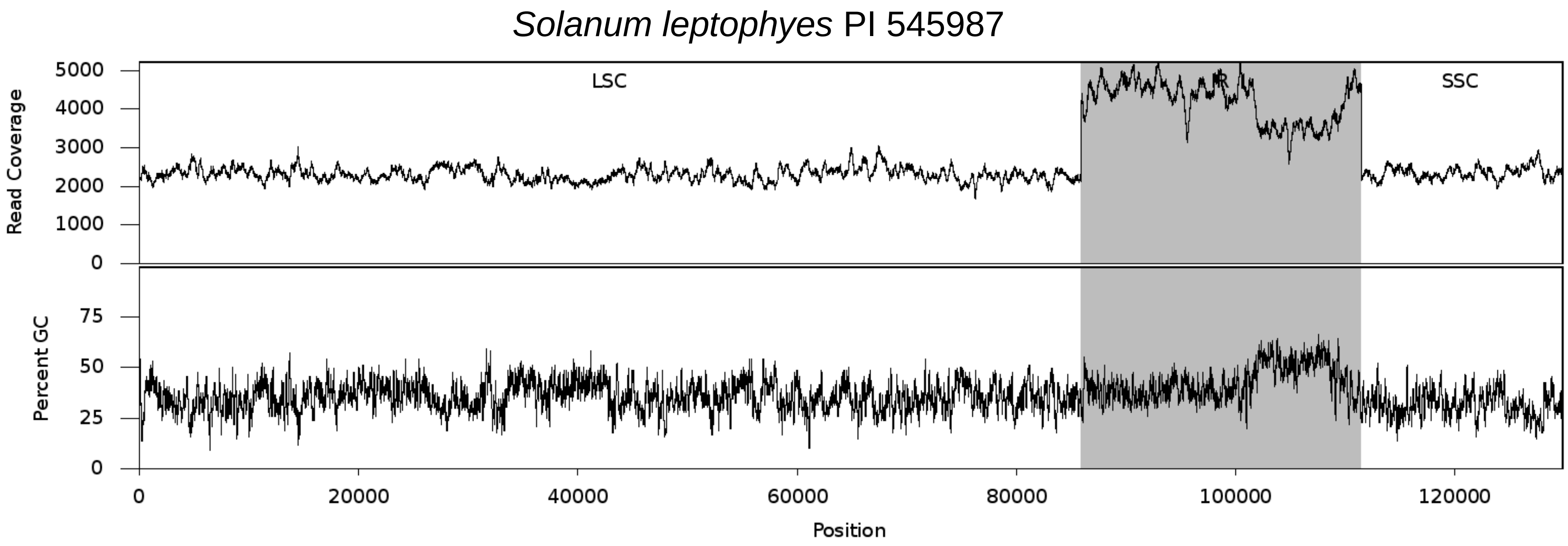
Solanum leptophyes PI 545987
LSC
IR
SSC
Read Coverage
5000
4000
3000
2000
1000
0
Percent GC
75
50
25
0
0
20000
40000
60000
80000
100000
120000
Position

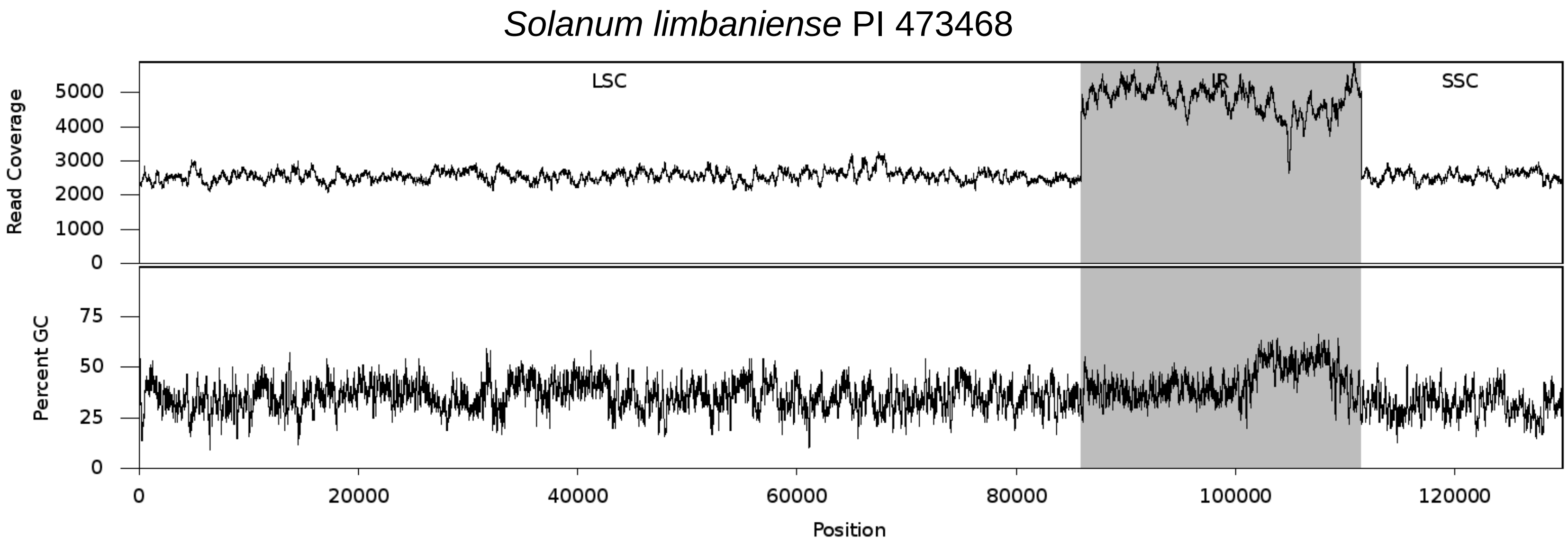
Solanum limbaniense PI 473468
LSC
IR
SSC
Read Coverage
5000
4000
3000
2000
1000
0
Percent GC
75
50
25
0
0
20000
40000
60000
80000
100000
120000
Position

Solanum marinasense PI 210040

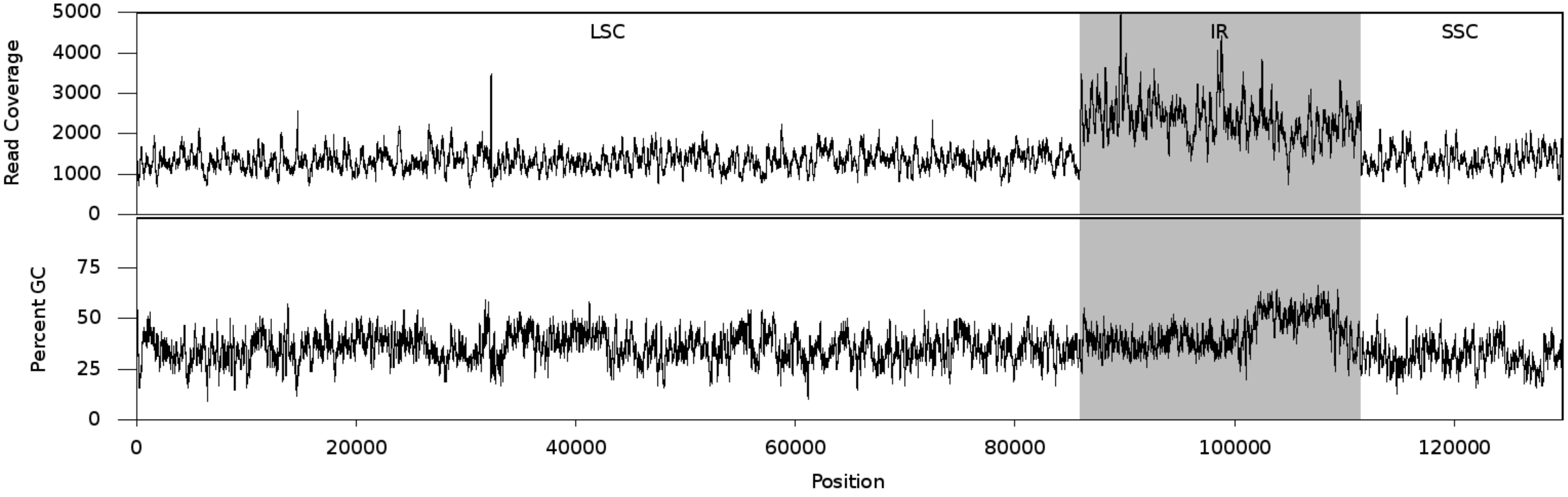

Solanum marinasense PI 310944

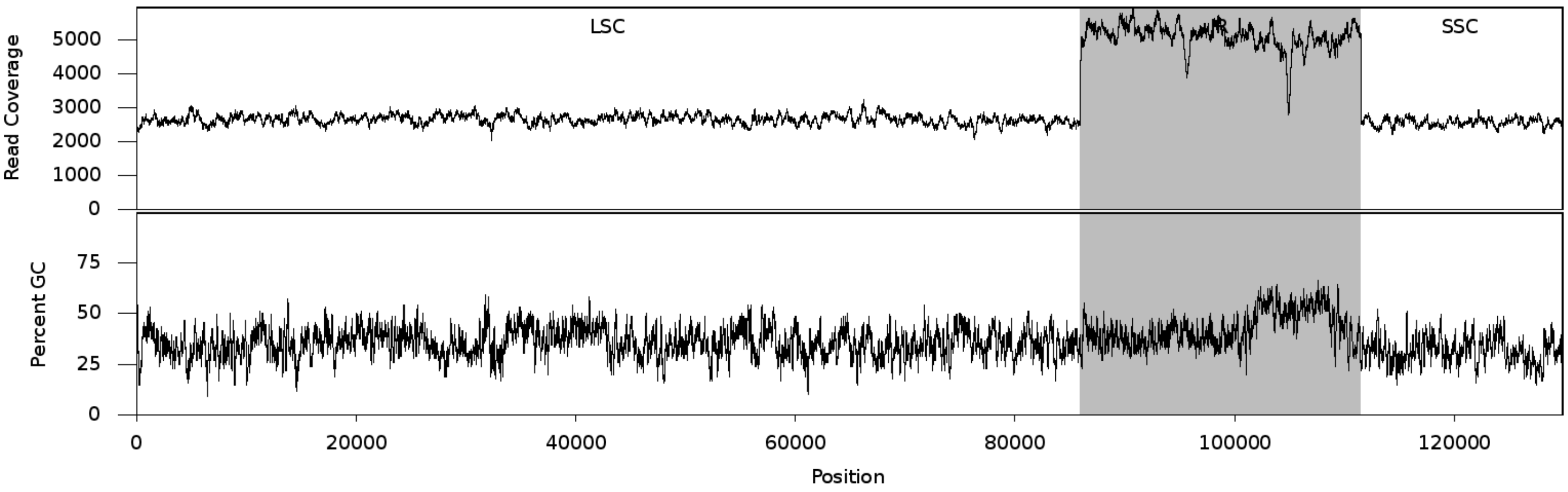

Solanum marinasense PI 498255

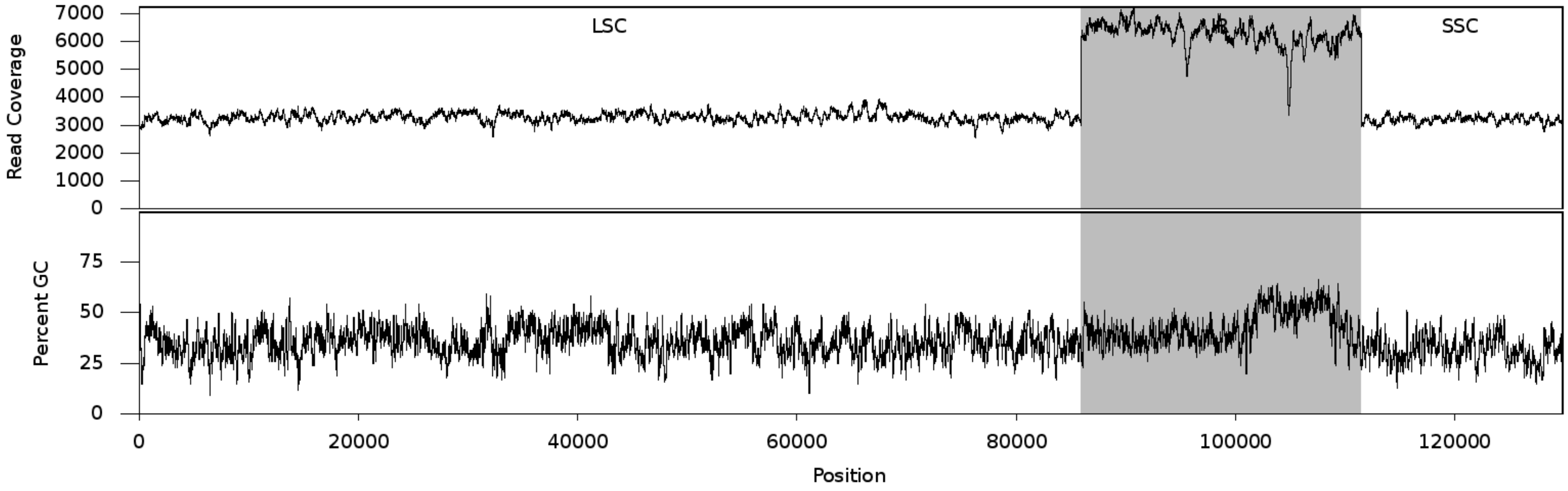

Solanum medians PI 210045

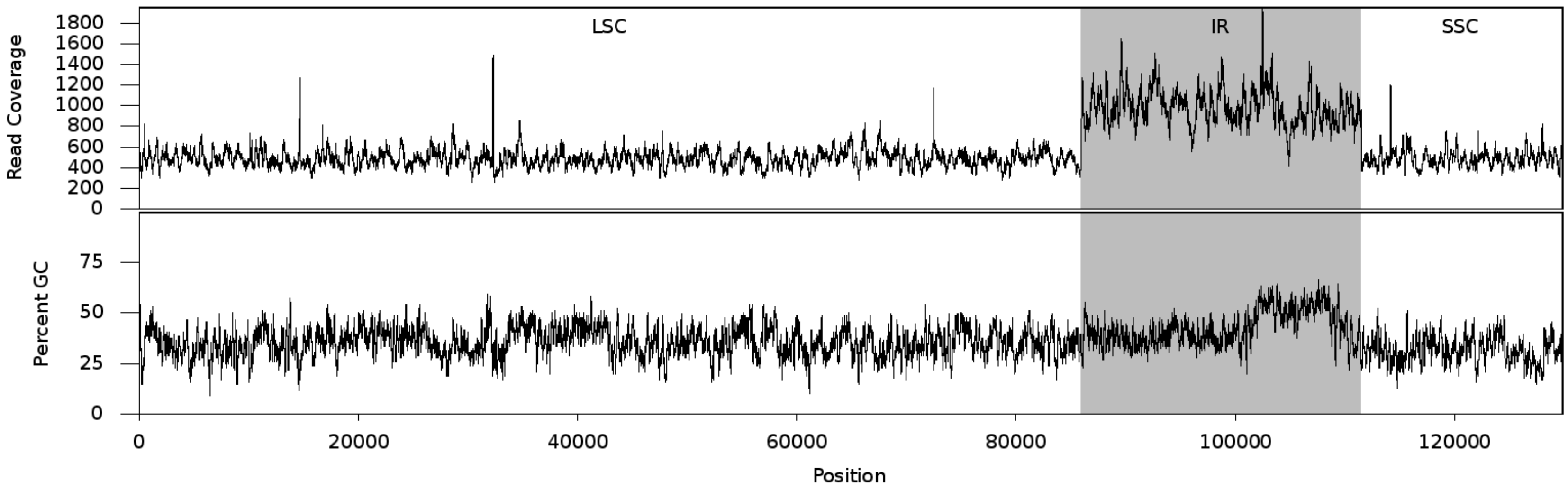

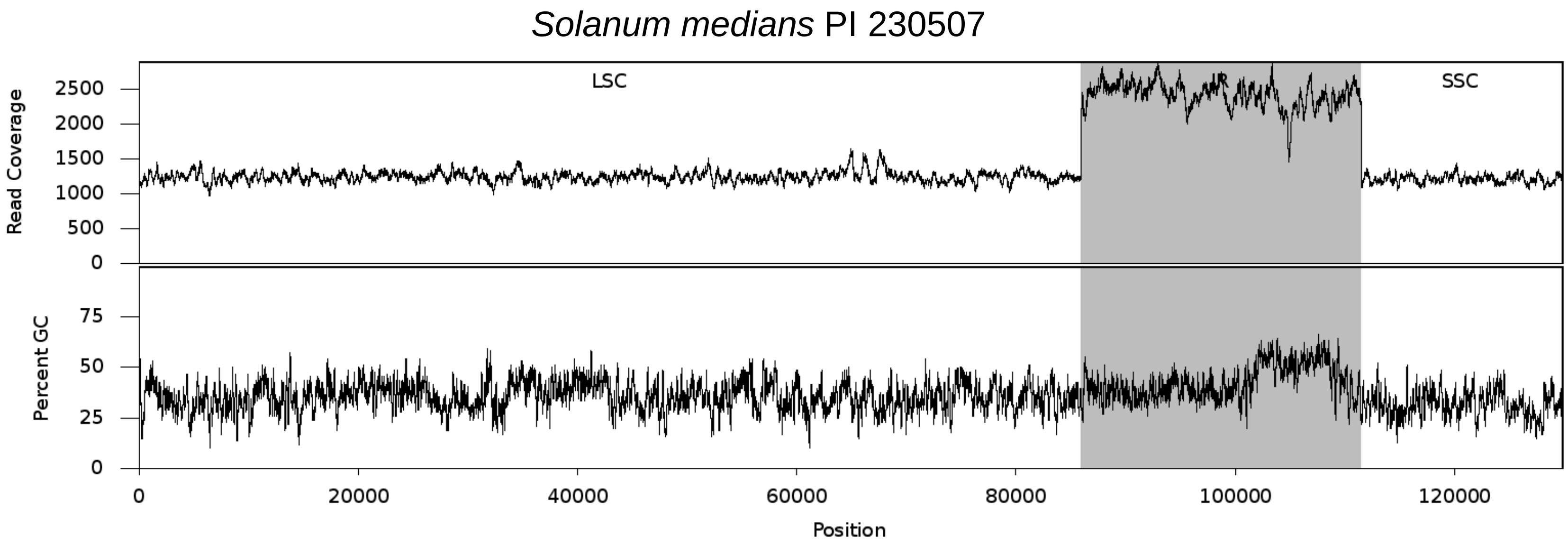

Solanum medians PI 230507
LSC
IR
SSC
Read Coverage
Percent GC
Position

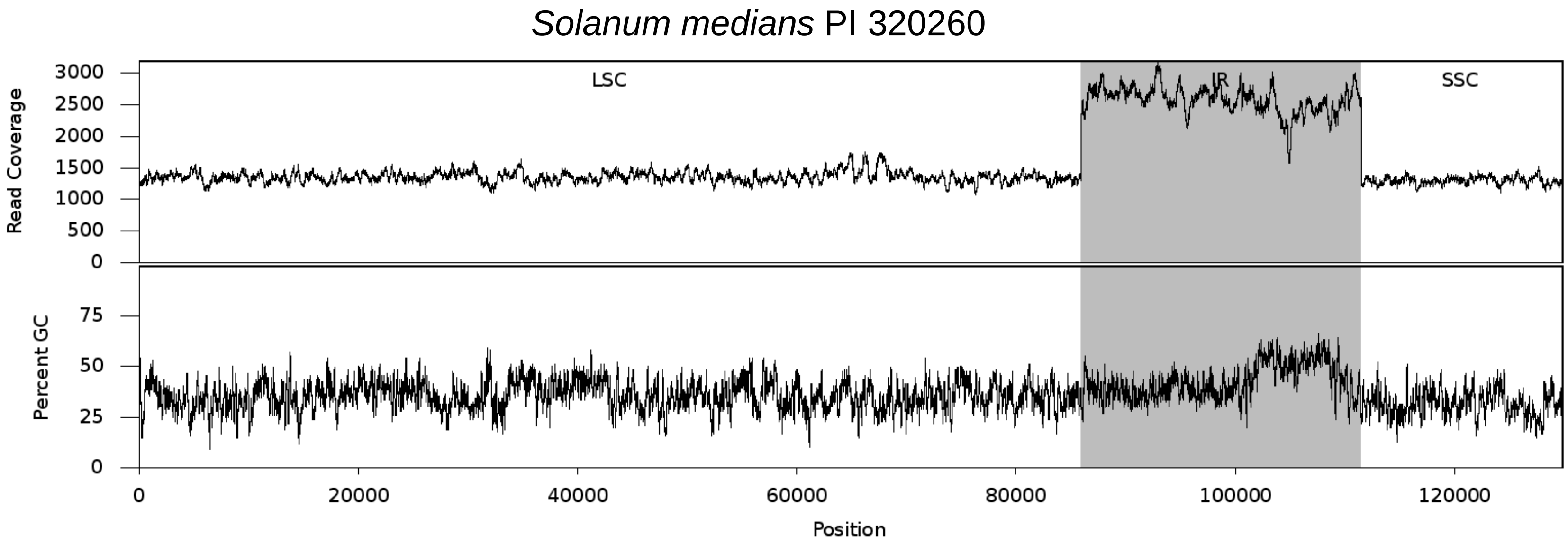

Solanum medians PI 320260
LSC
IR
SSC
Read Coverage
Percent GC
Position

Solanum medians PI 458402

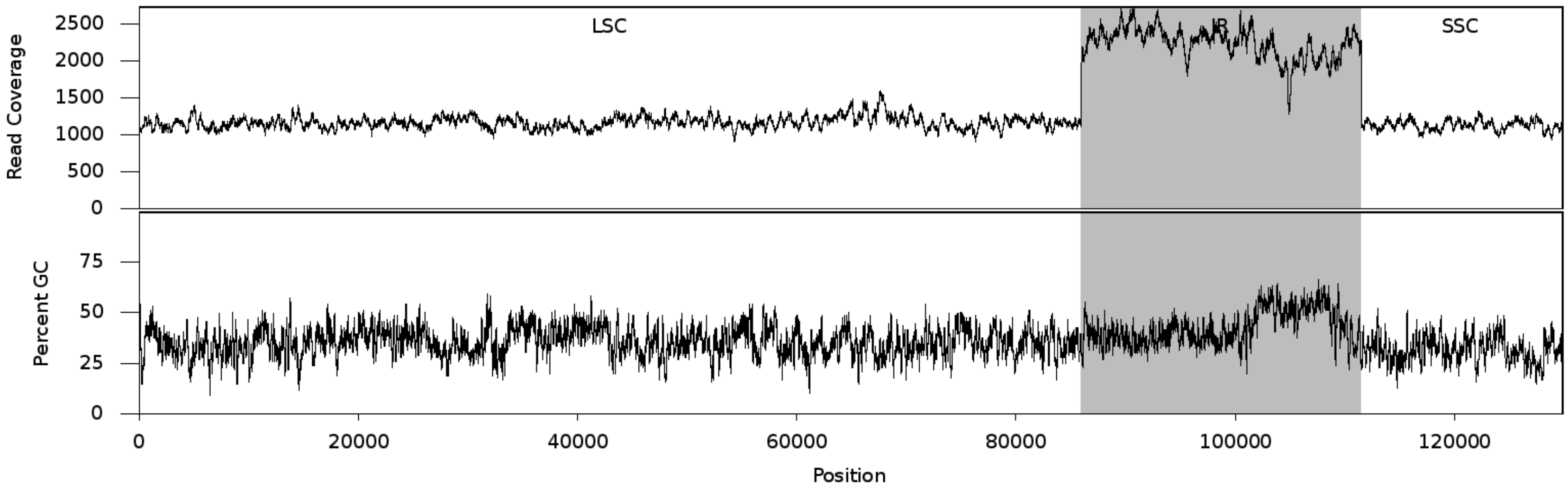

Solanum medians PI 473496

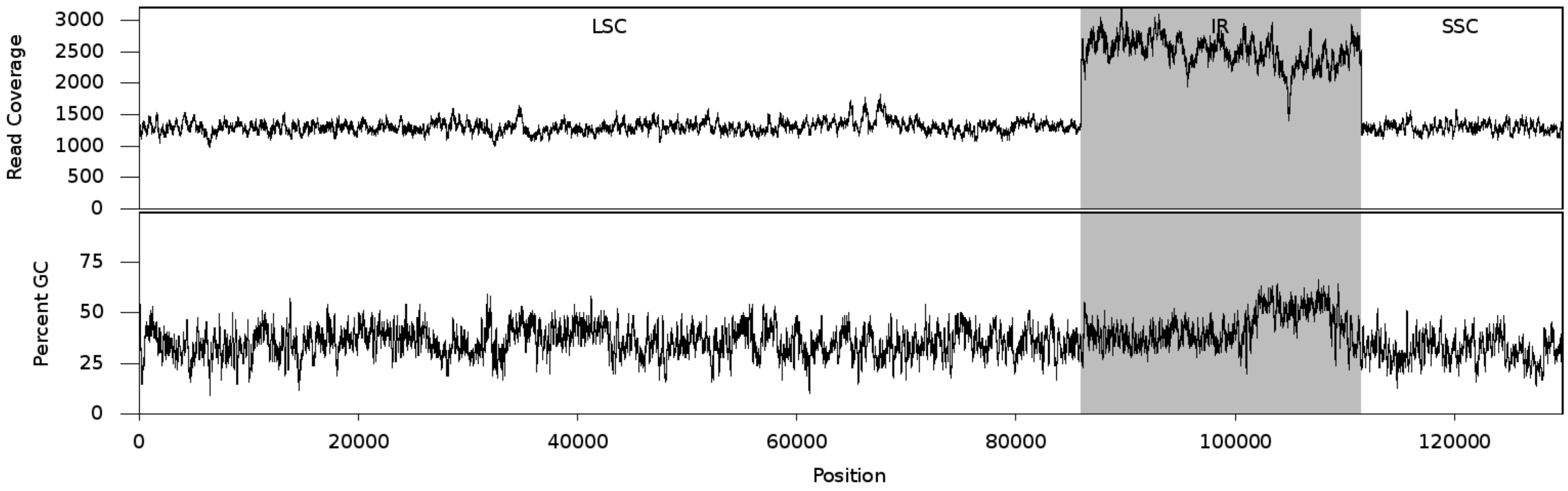

Solanum megistacrolobum PI 210034

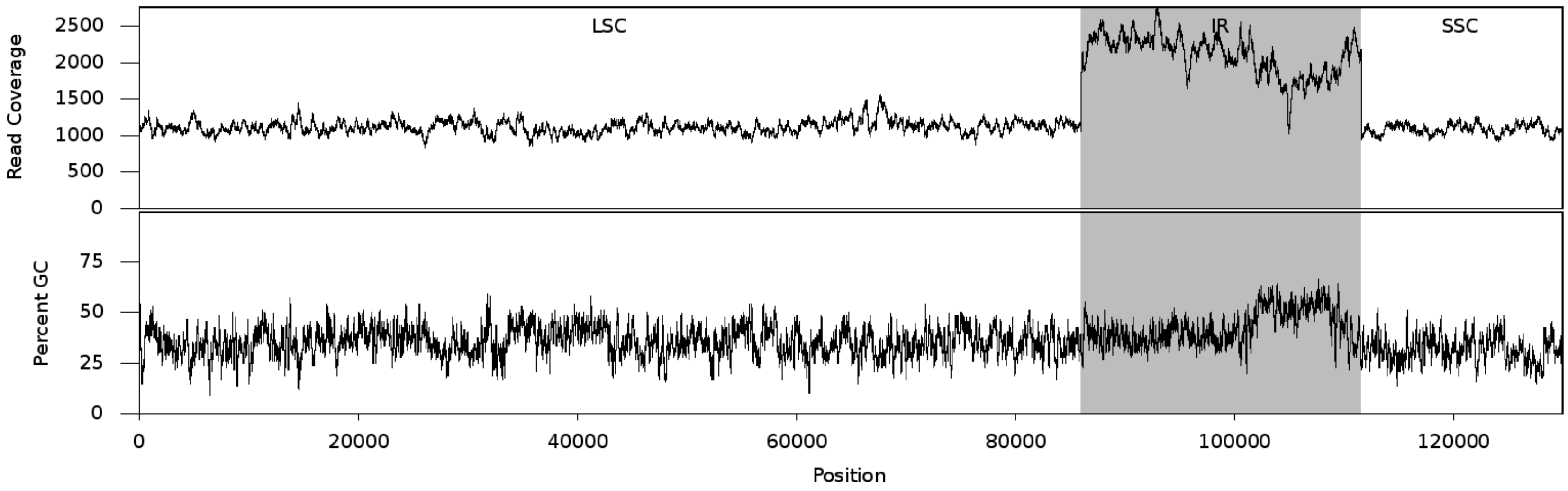

Solanum megistacrolobum PI 473158

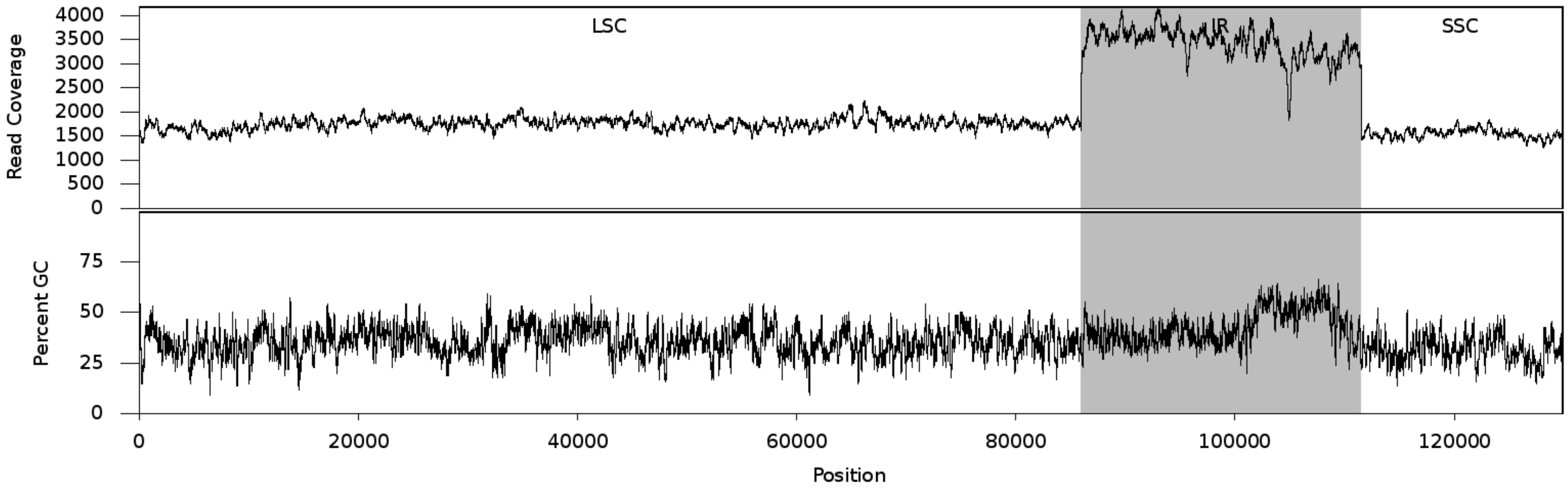

Solanum megistacrolobum PI 500029

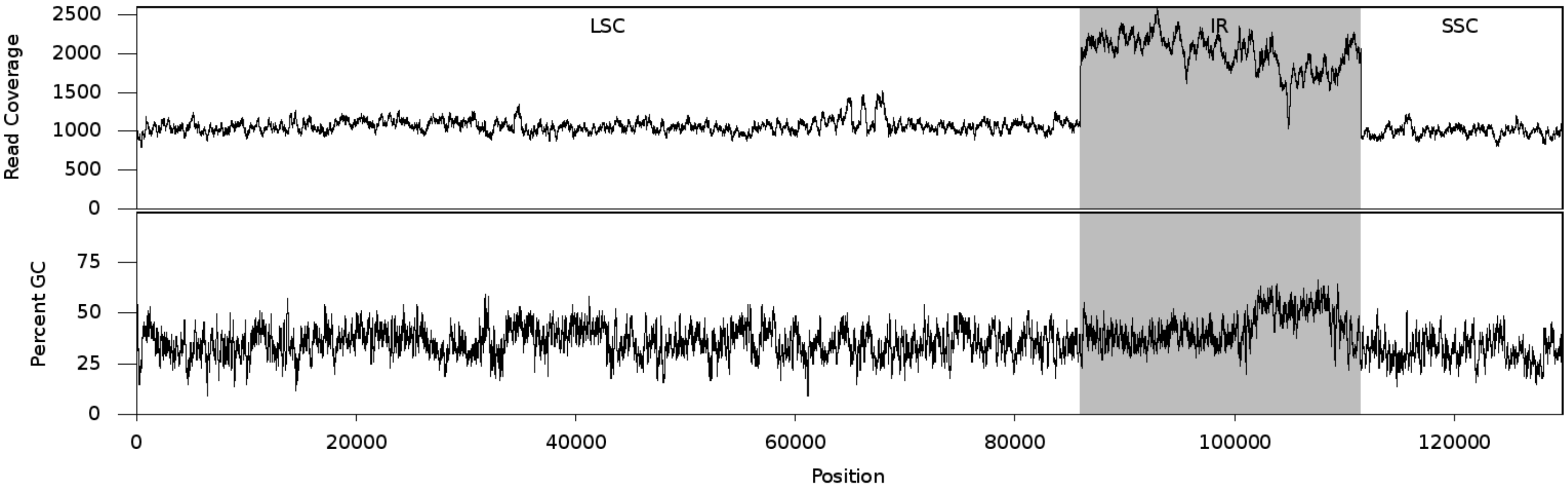

Solanum megistacrolobum PI 546000

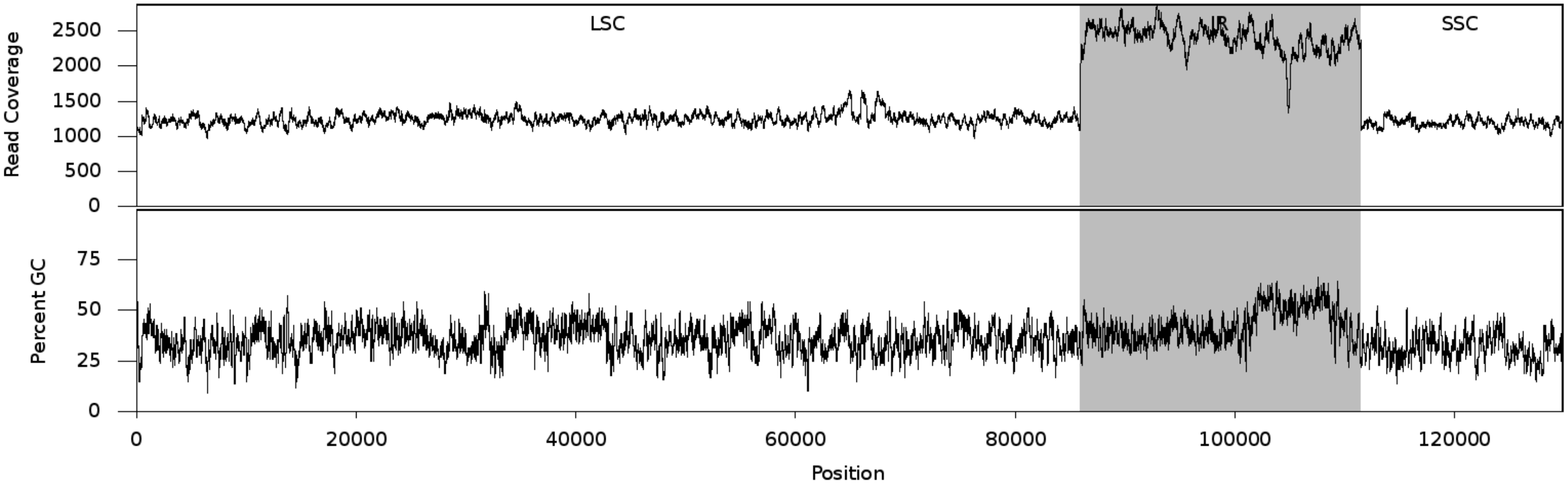

Solanum microdontum PI 218225

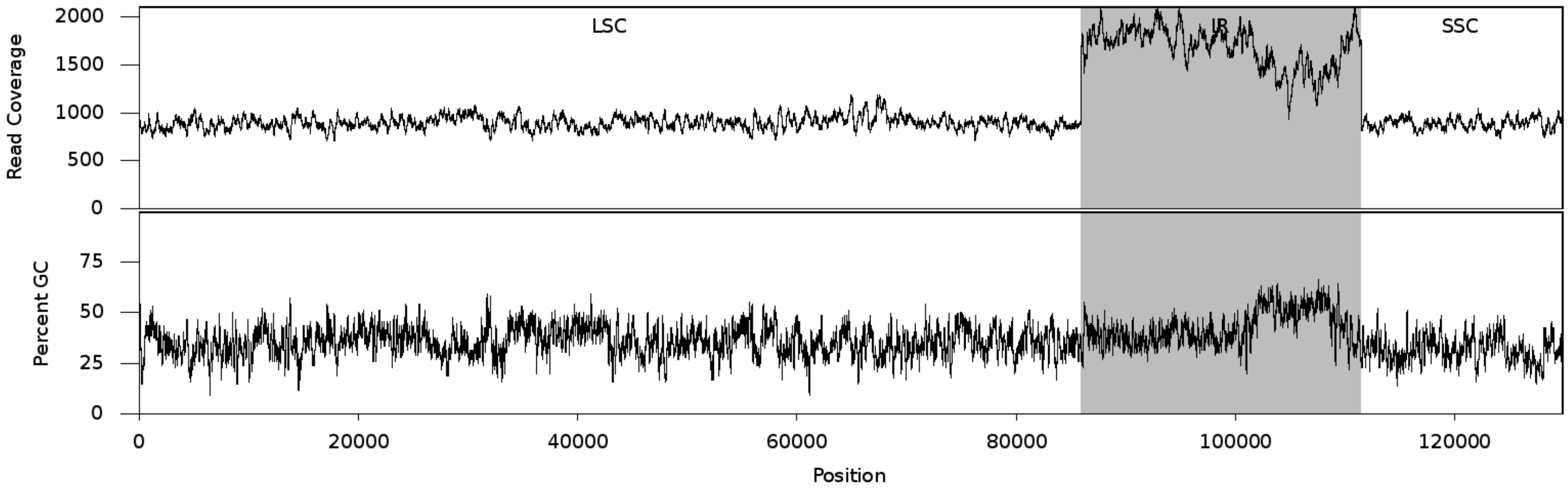

Solanum microdontum PI 545884

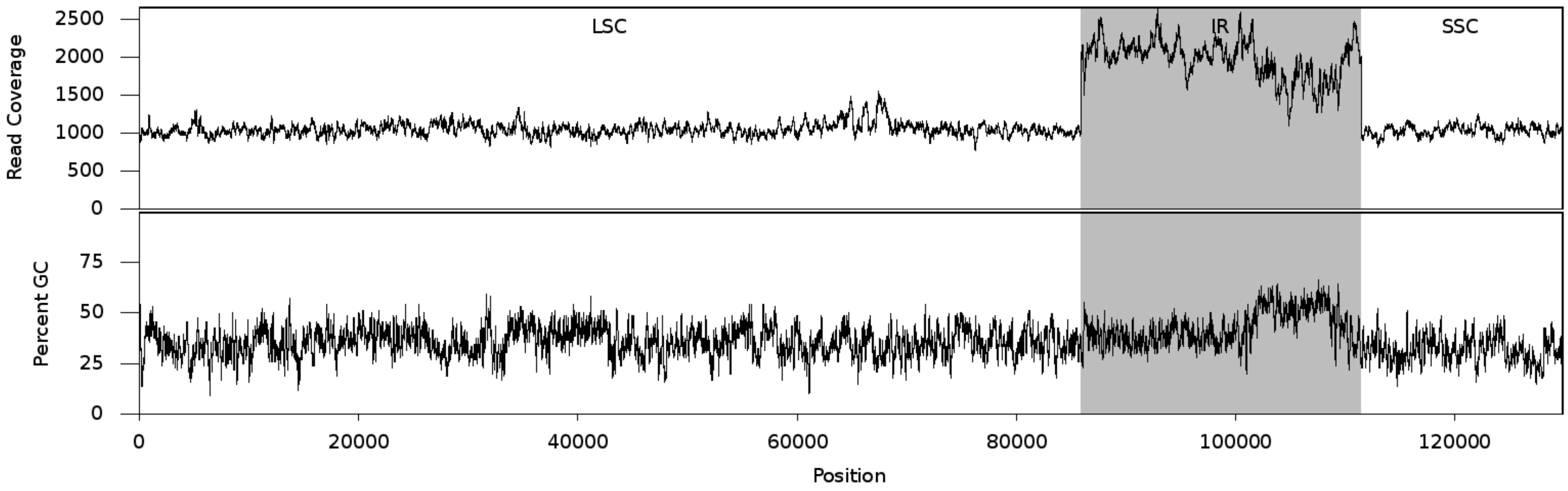

Solanum multidissectum PI 210052

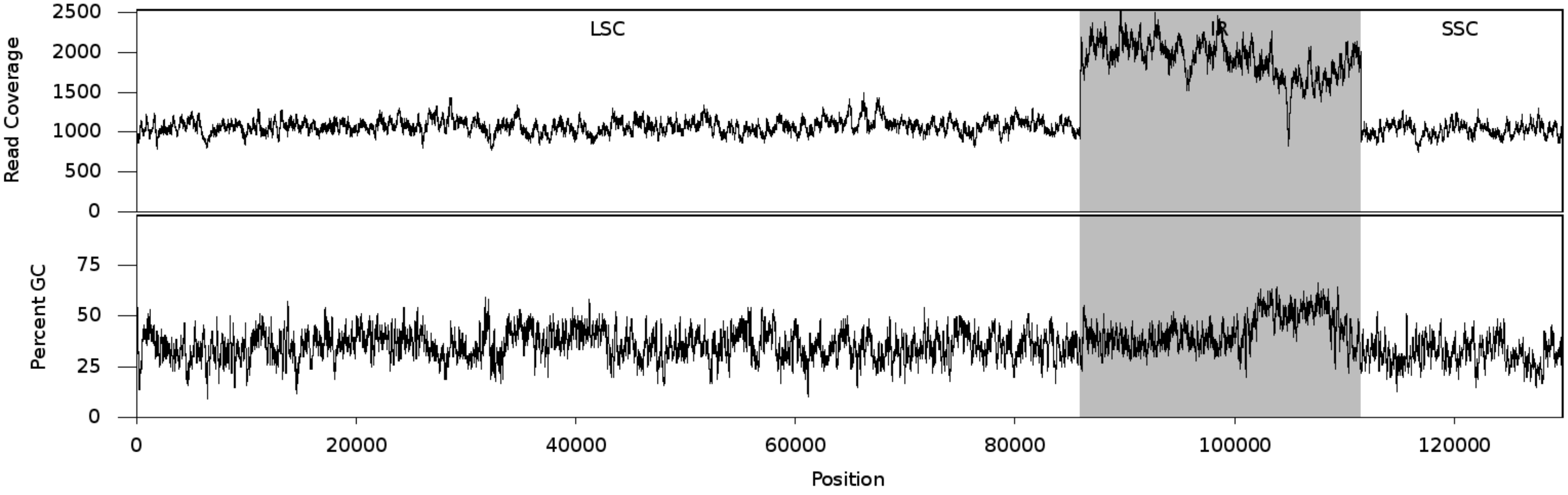

Solanum multidissectum PI 210055

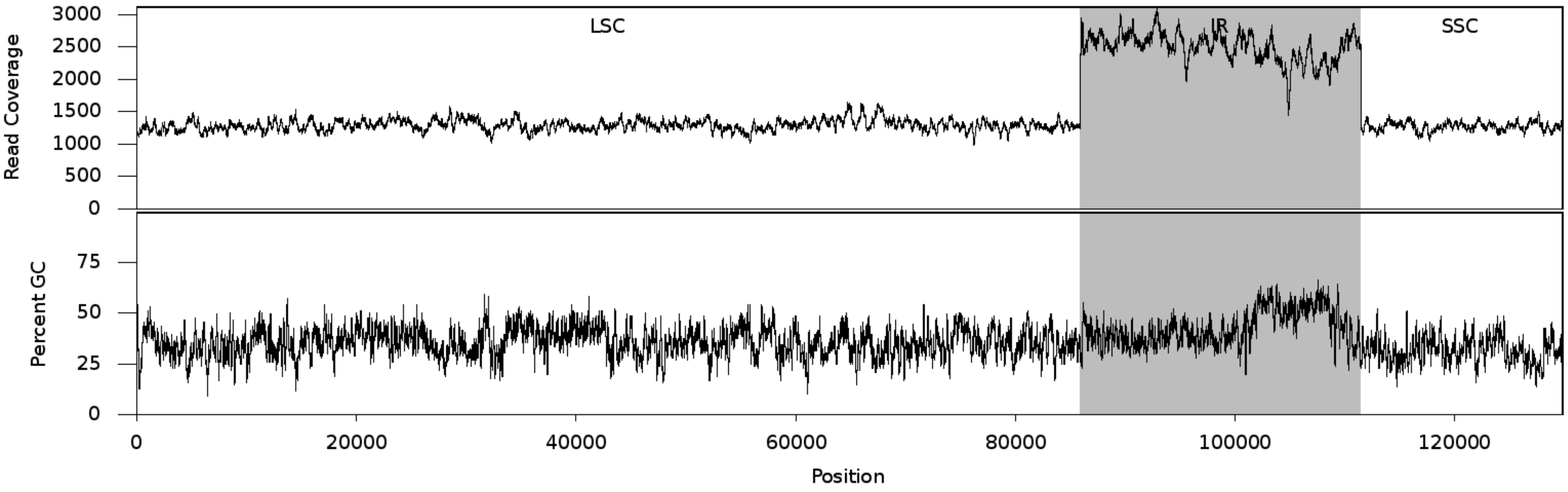

Solanum multidissectum PI 275272

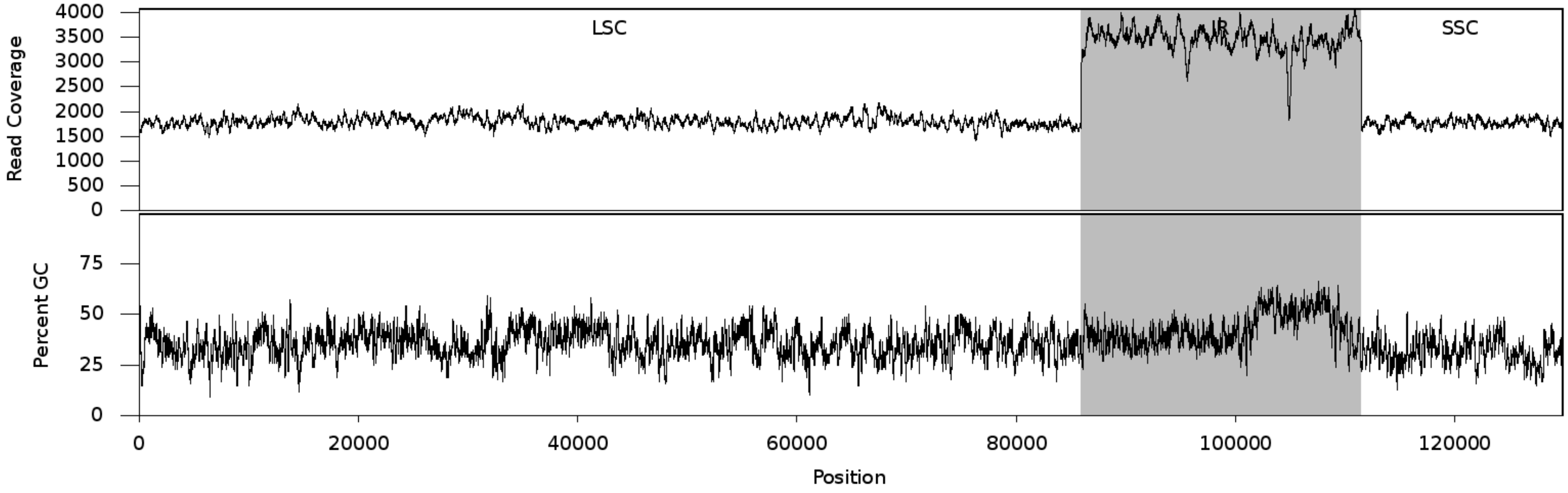

Solanum multidissectum PI 473349

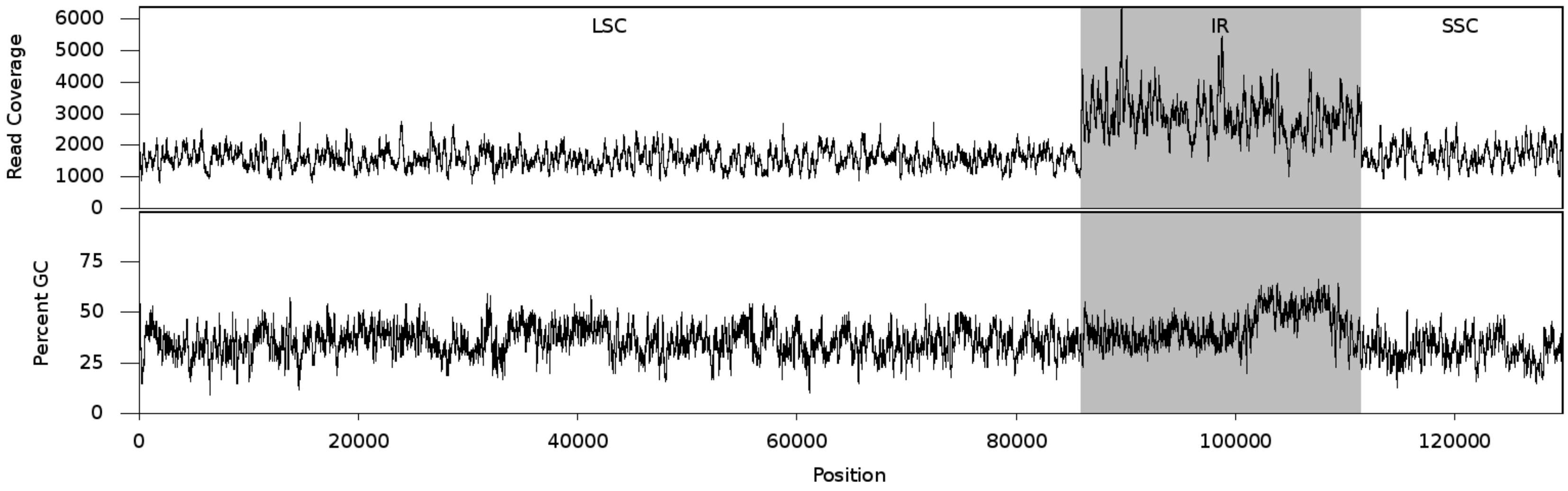

Solanum multidissectum PI 473352

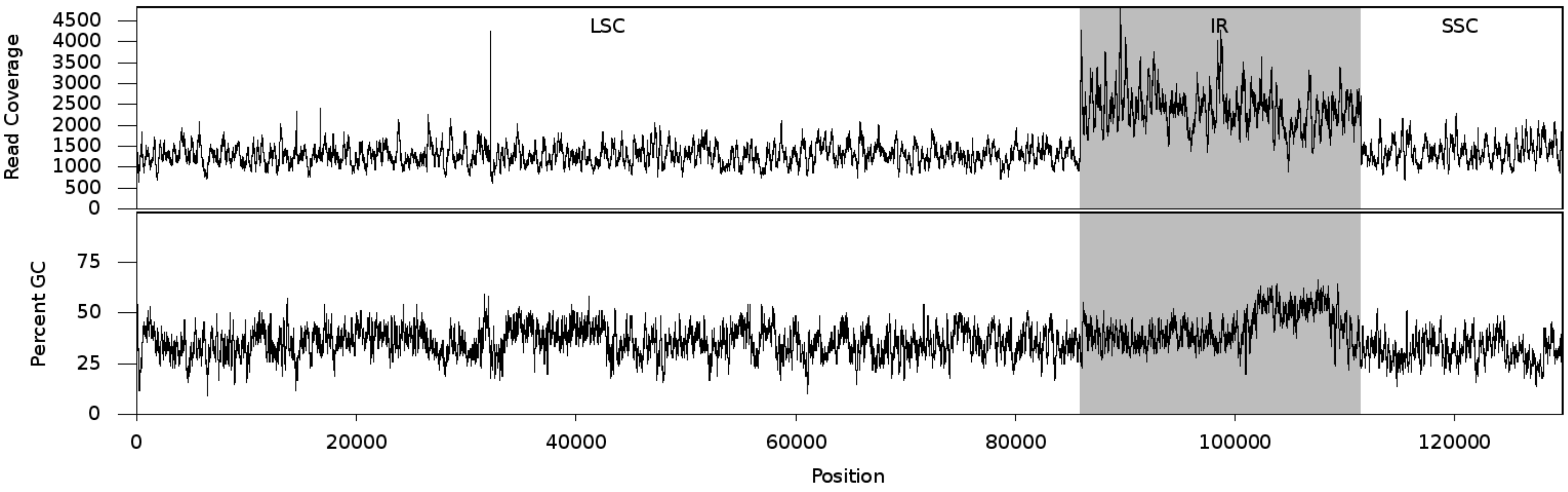

Solanum multiinterruptum PI 210044

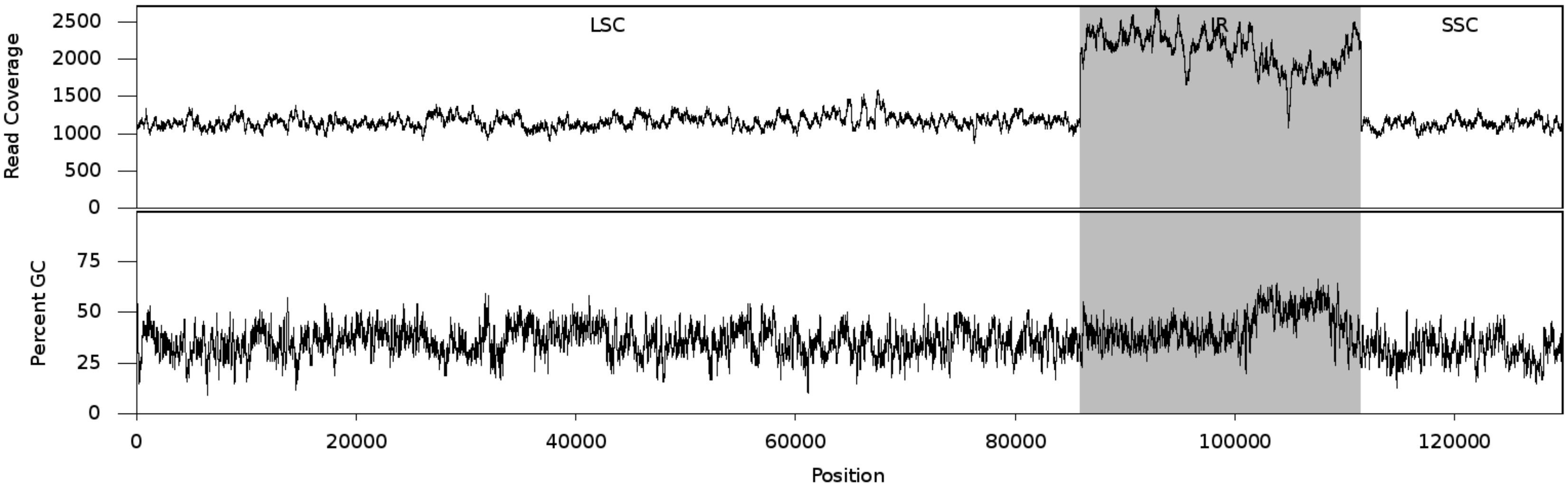

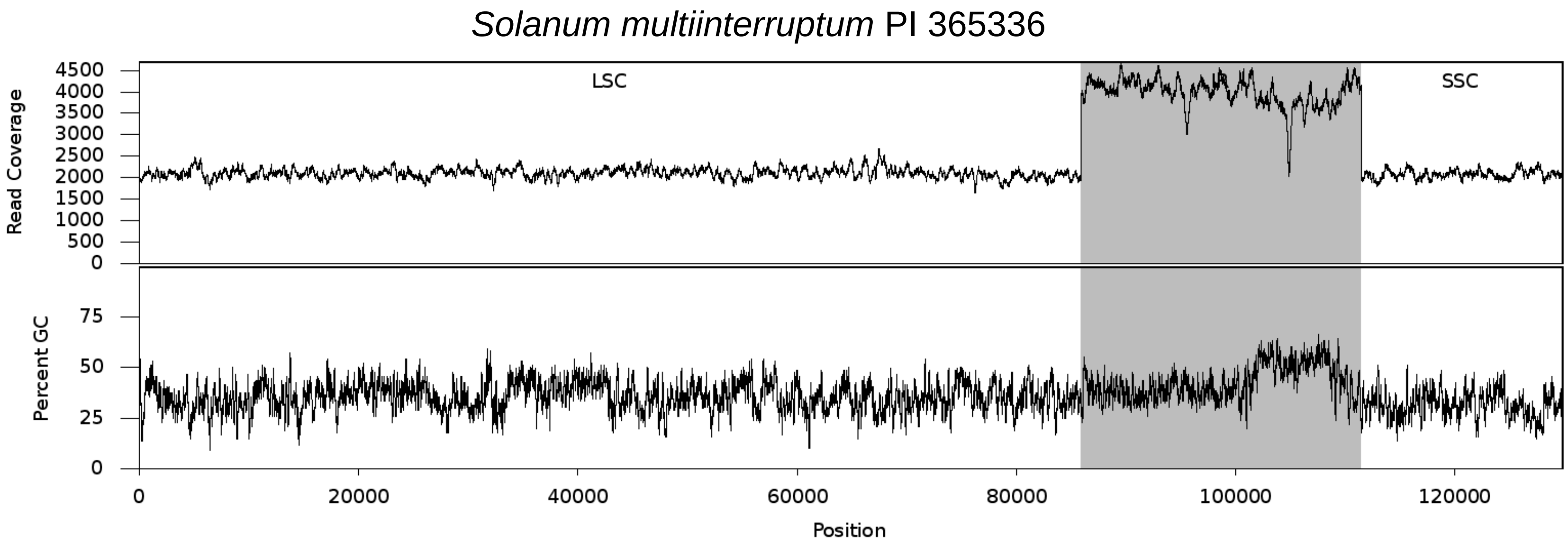
Solanum multiinterruptum PI 365336
LSC
IR
SSC
Read Coverage
4500
4000
3500
3000
2500
2000
1500
1000
500
0
Percent GC
75
50
25
0
0
20000
40000
60000
80000
100000
120000
Position

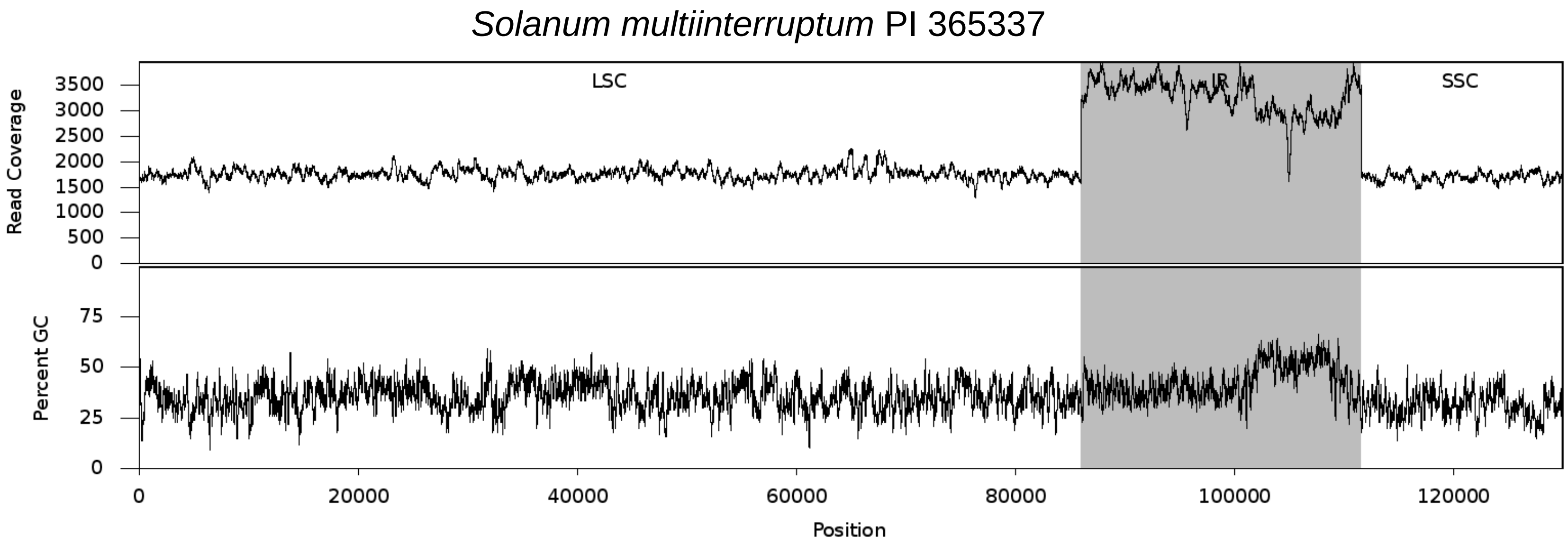
Solanum multiinterruptum PI 365337
LSC
IR
SSC
Read Coverage
3500
3000
2500
2000
1500
1000
500
0
Percent GC
75
50
25
0
0
20000
40000
60000
80000
100000
120000
Position

Solanum multiinterruptum PI 365338

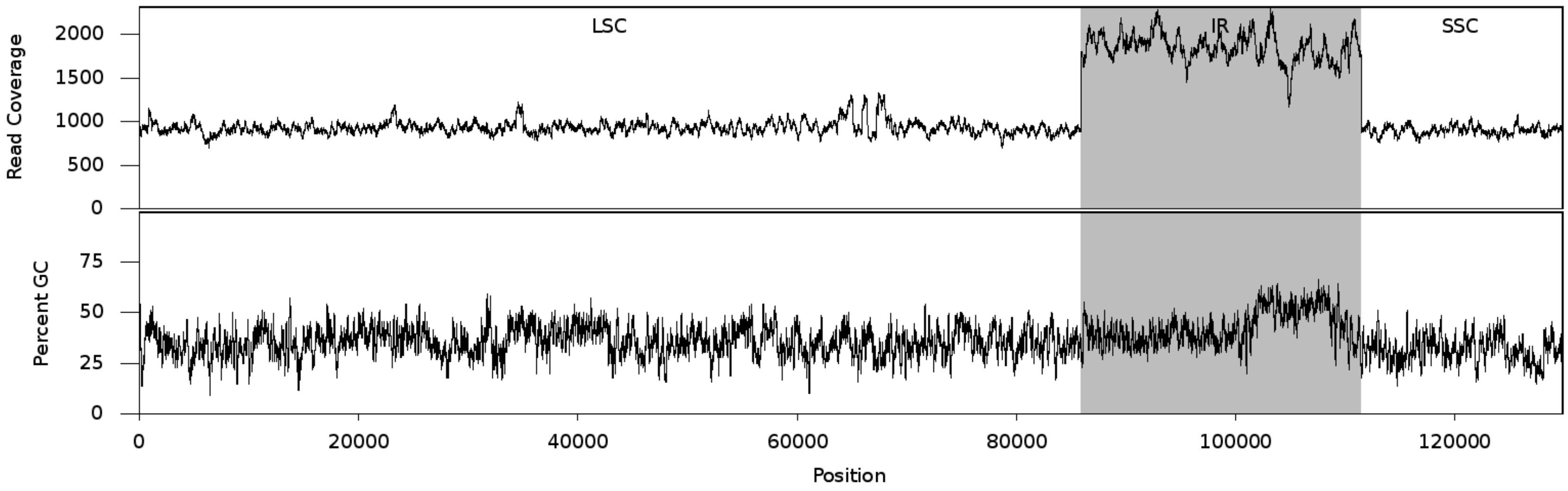

Solanum multiinterruptum PI 498266

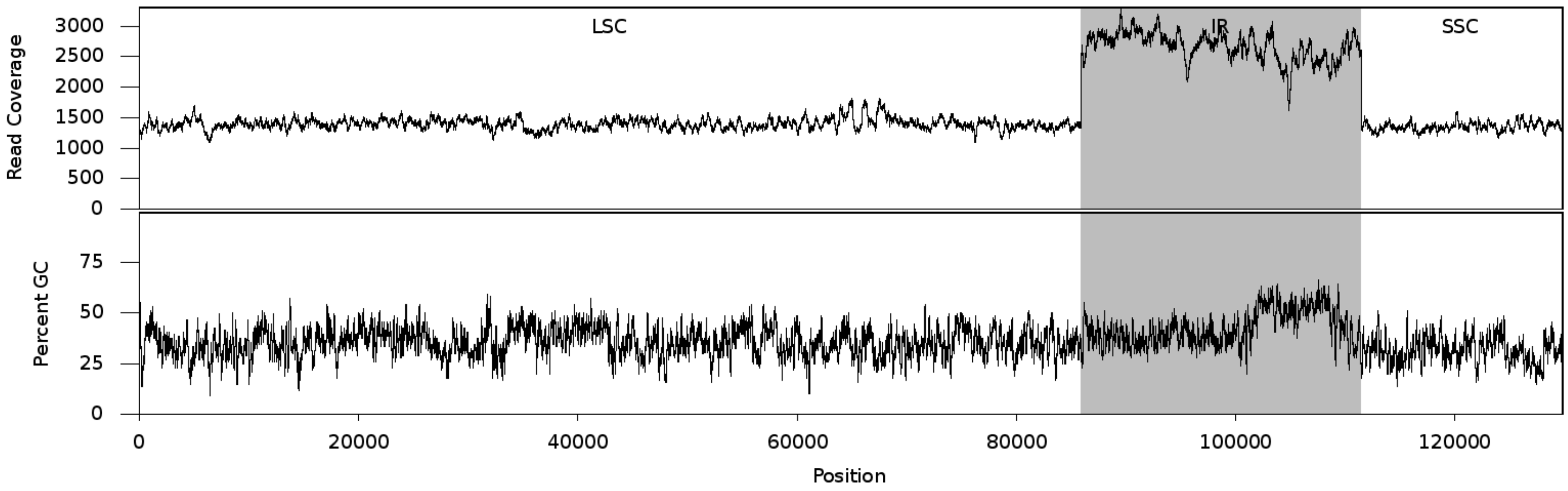

Solanum palustre PI 245763

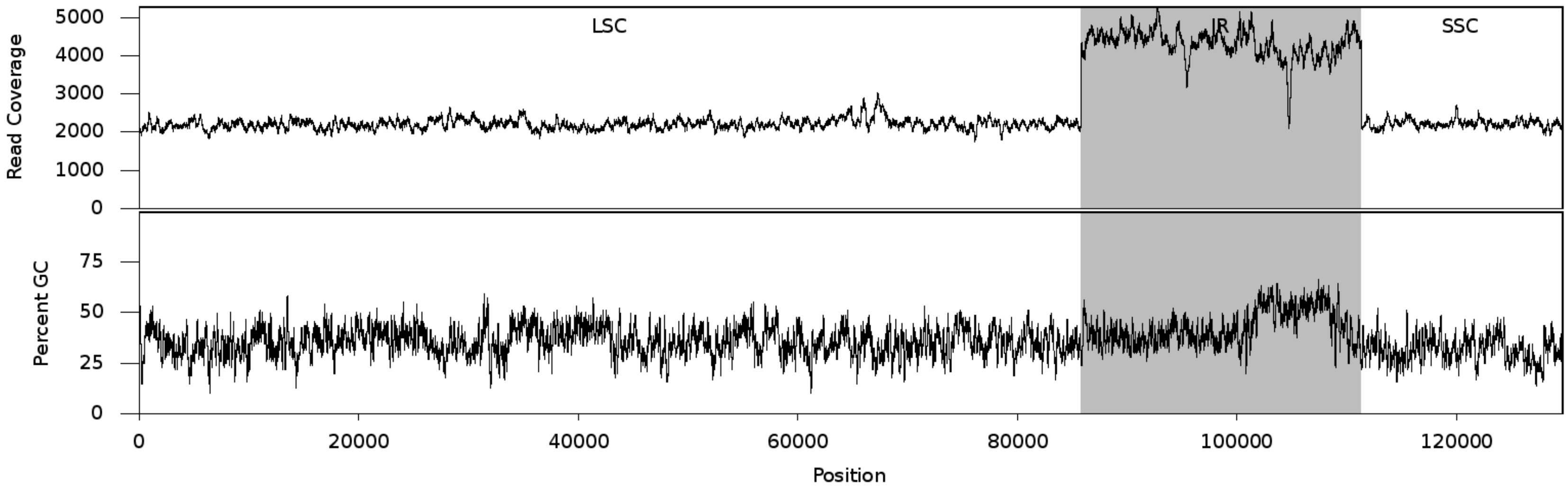

Solanum pampasense PI 275274

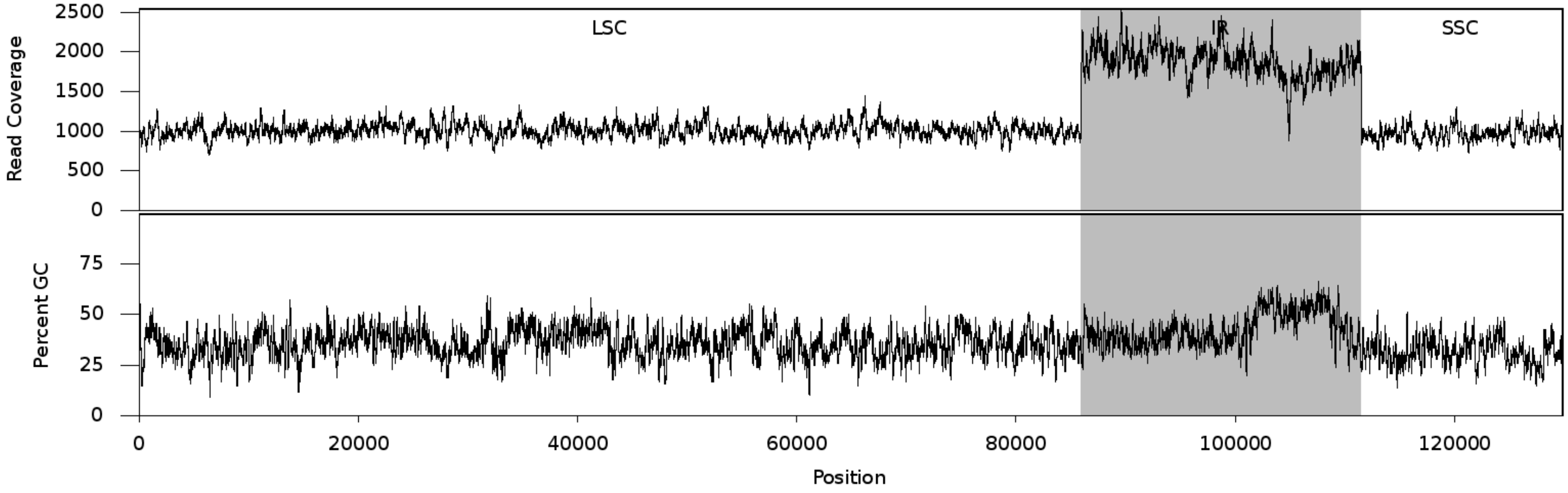

Solanum pampasense PI 275275

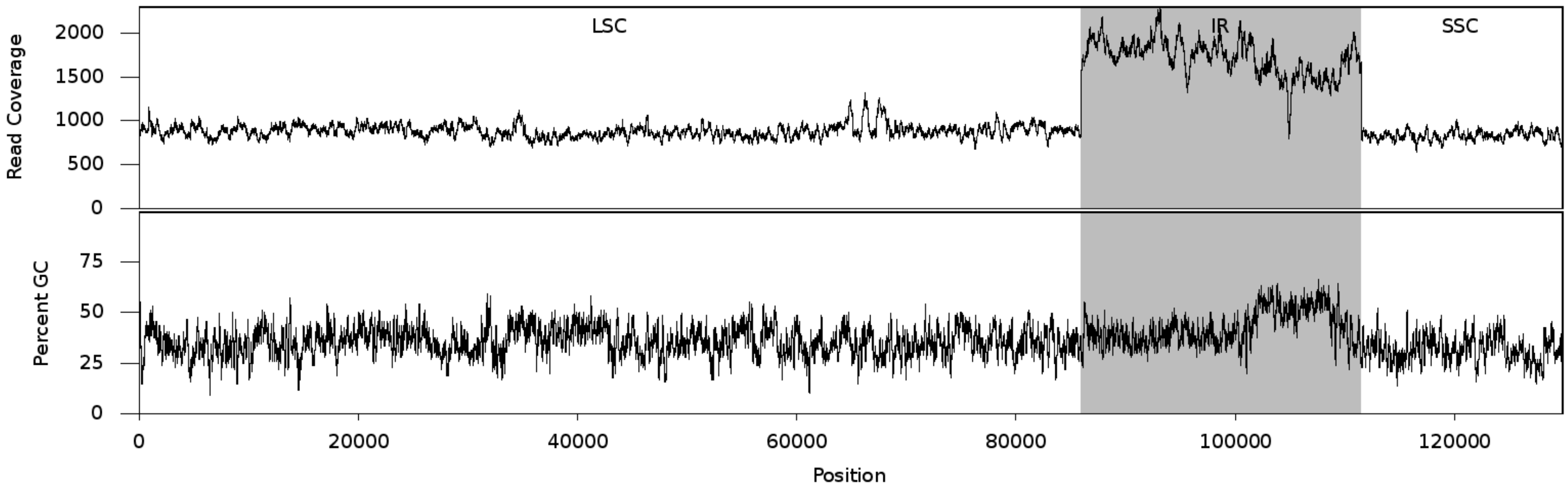

Solanum pampasense PI 442697

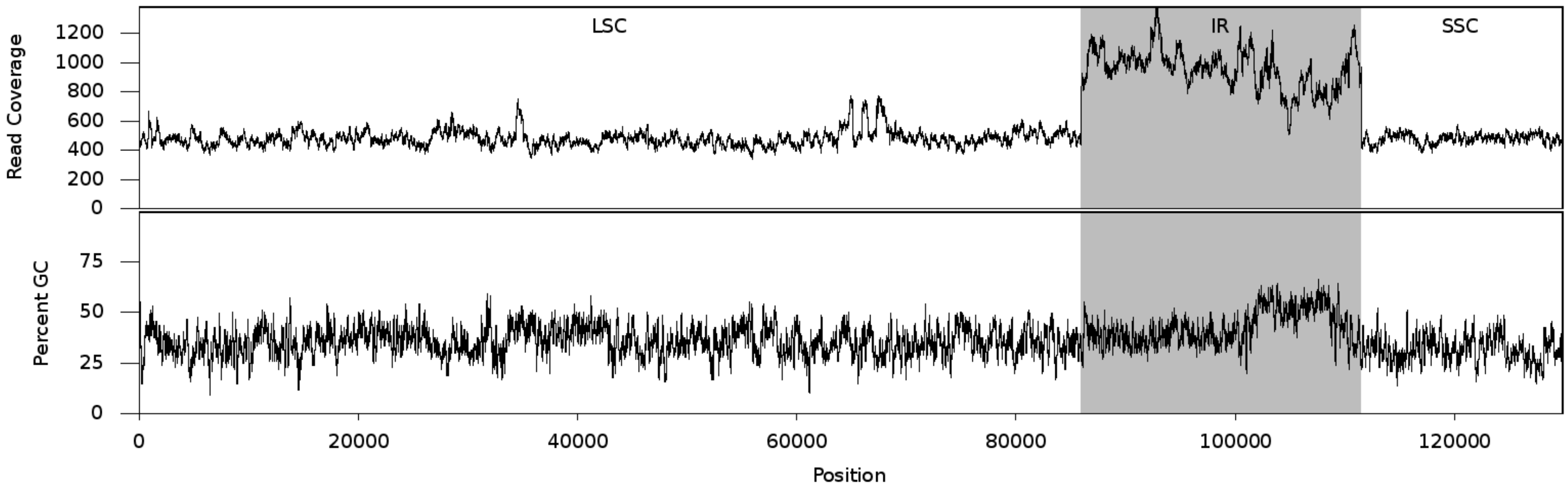

Solanum pampasense PI 458381

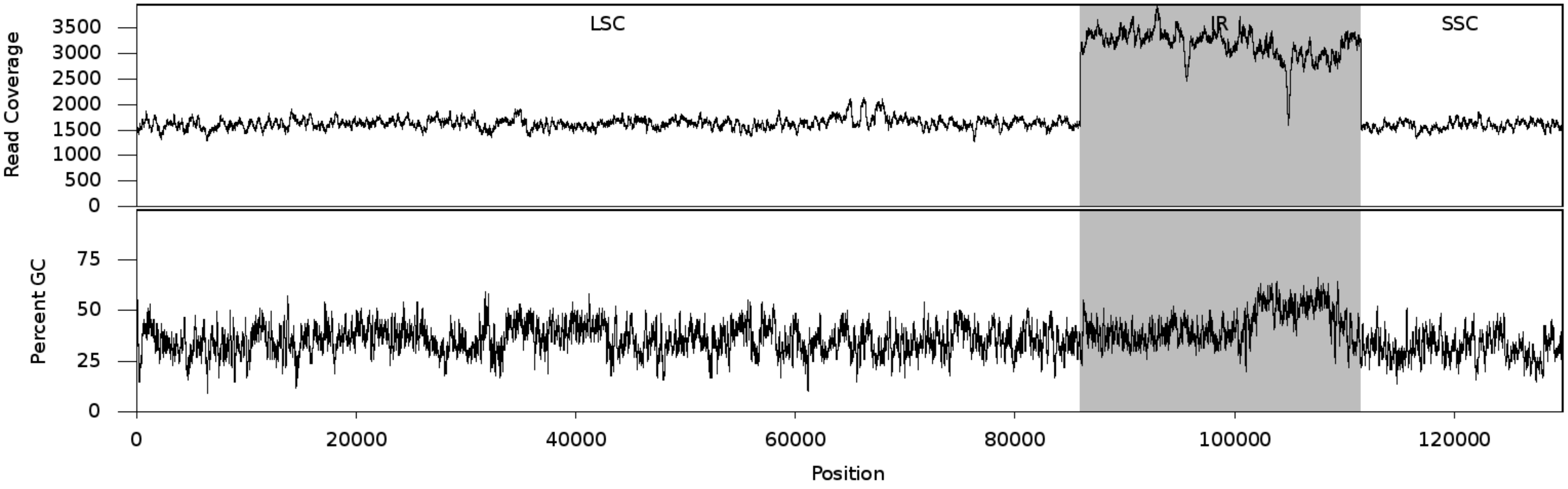

Solanum phureja PI 195191

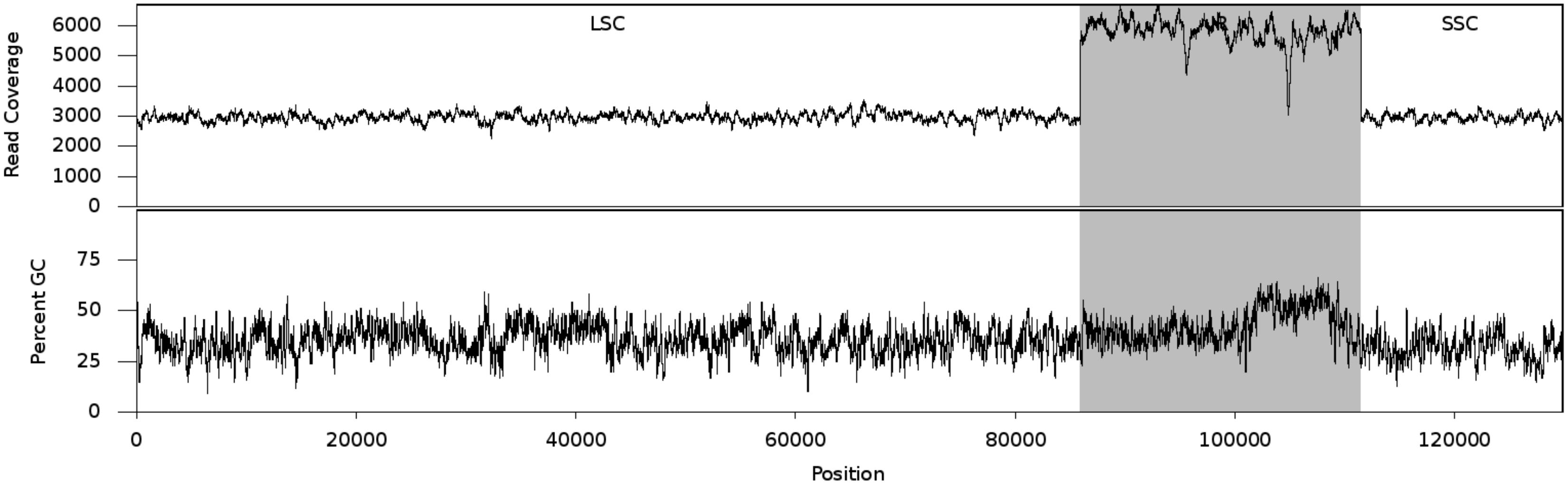

Solanum phureja PI 195198

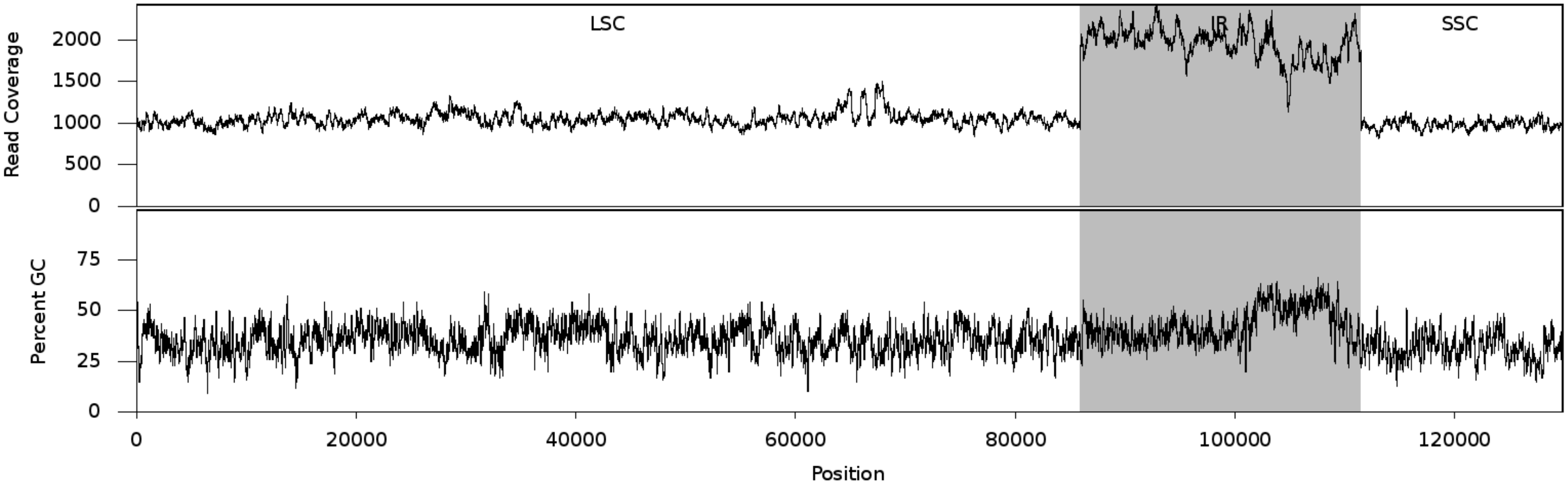

Solanum phureja PI 225665

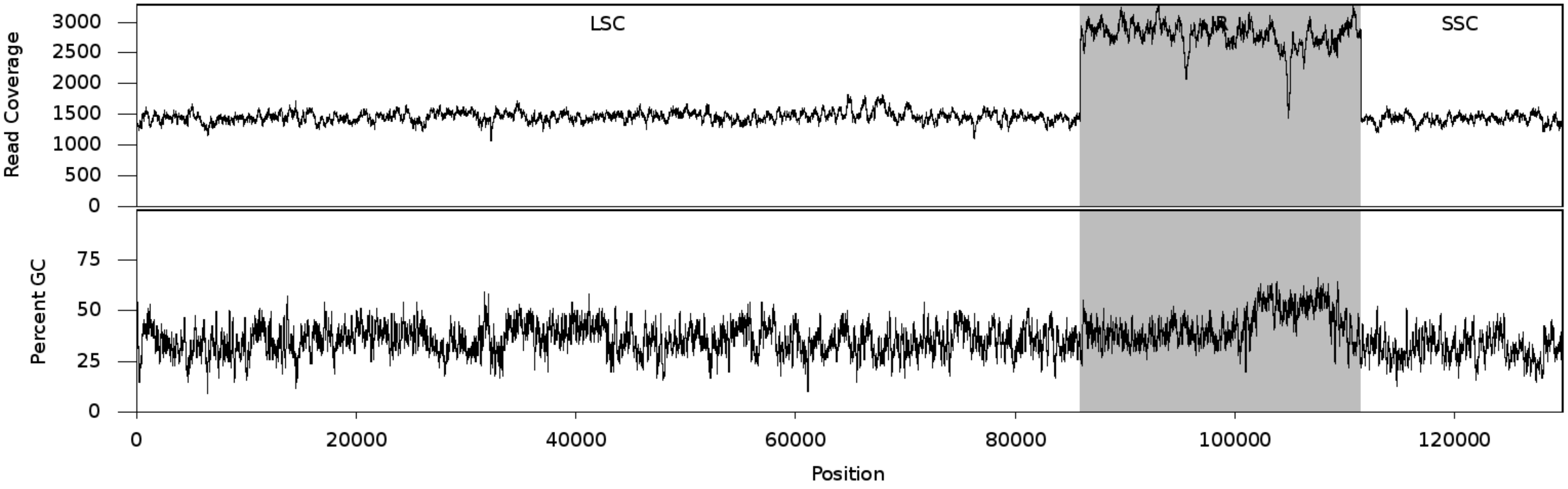

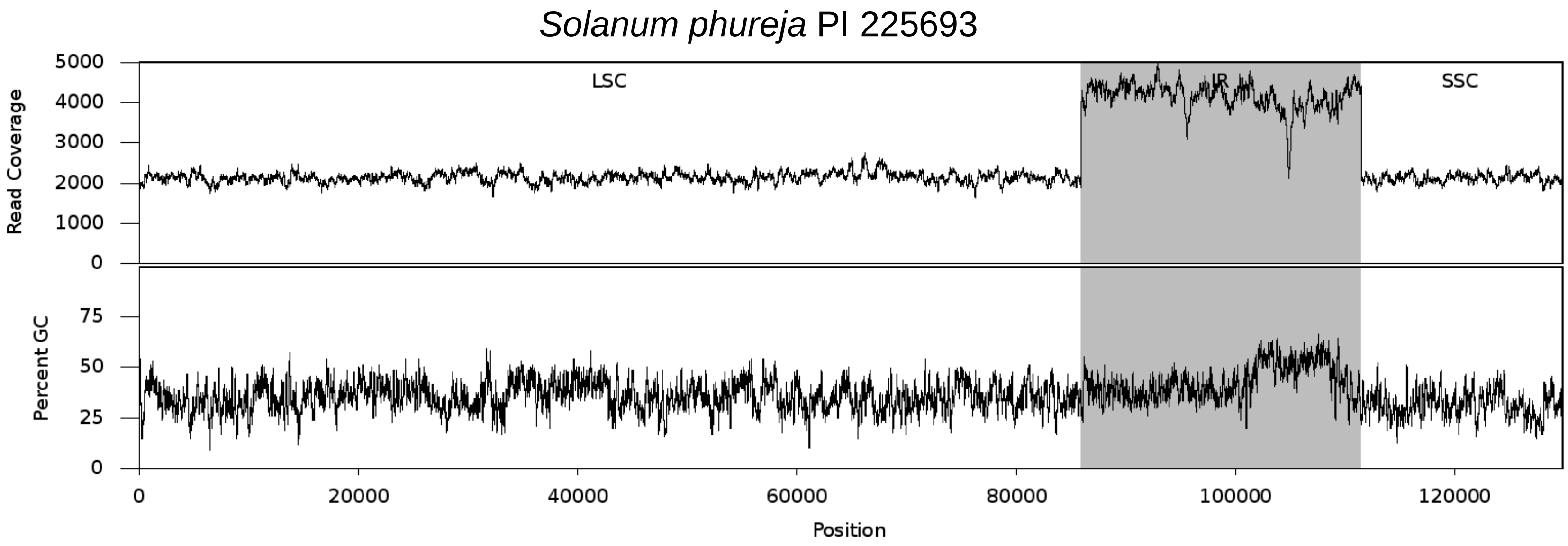

Solanum phureja PI 225693
Read Coverage
Percent GC
LSC
IR
SSC
Position
0
20000
40000
60000
80000
100000
120000
5000
4000
3000
2000
1000
75
50
25

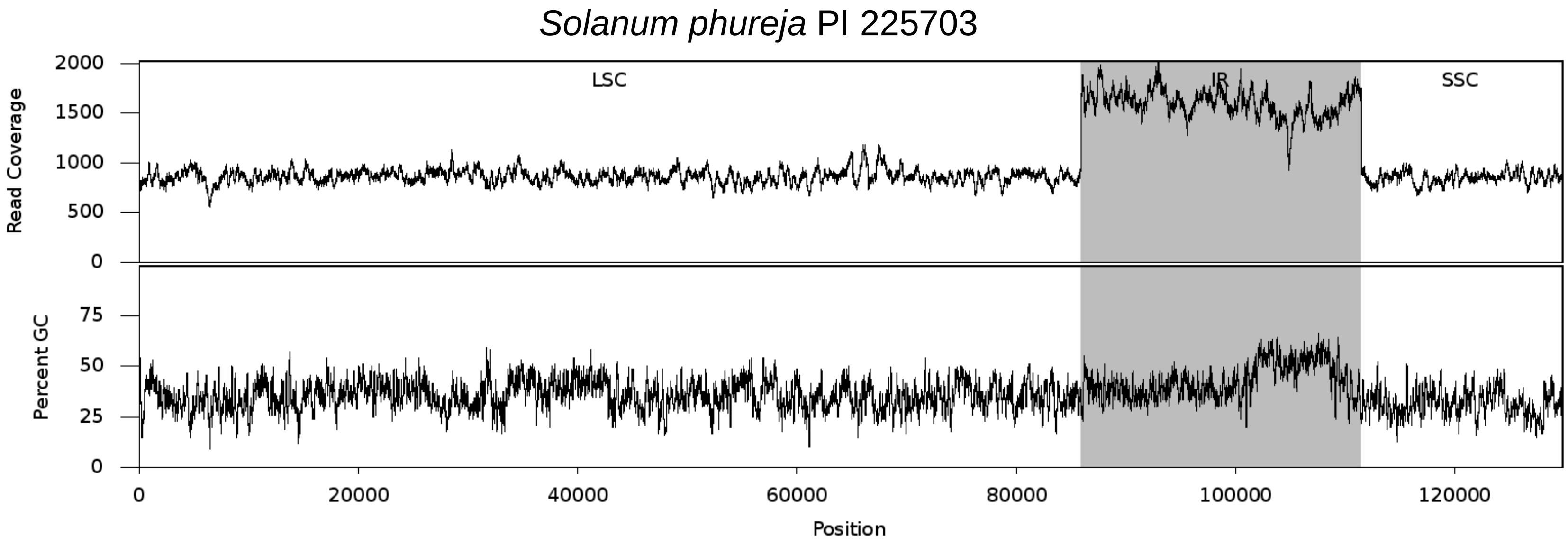

Solanum phureja PI 225703
Read Coverage
Percent GC
LSC
IR
SSC
Position
0
20000
40000
60000
80000
100000
120000
2000
1500
1000
500
75
50
25

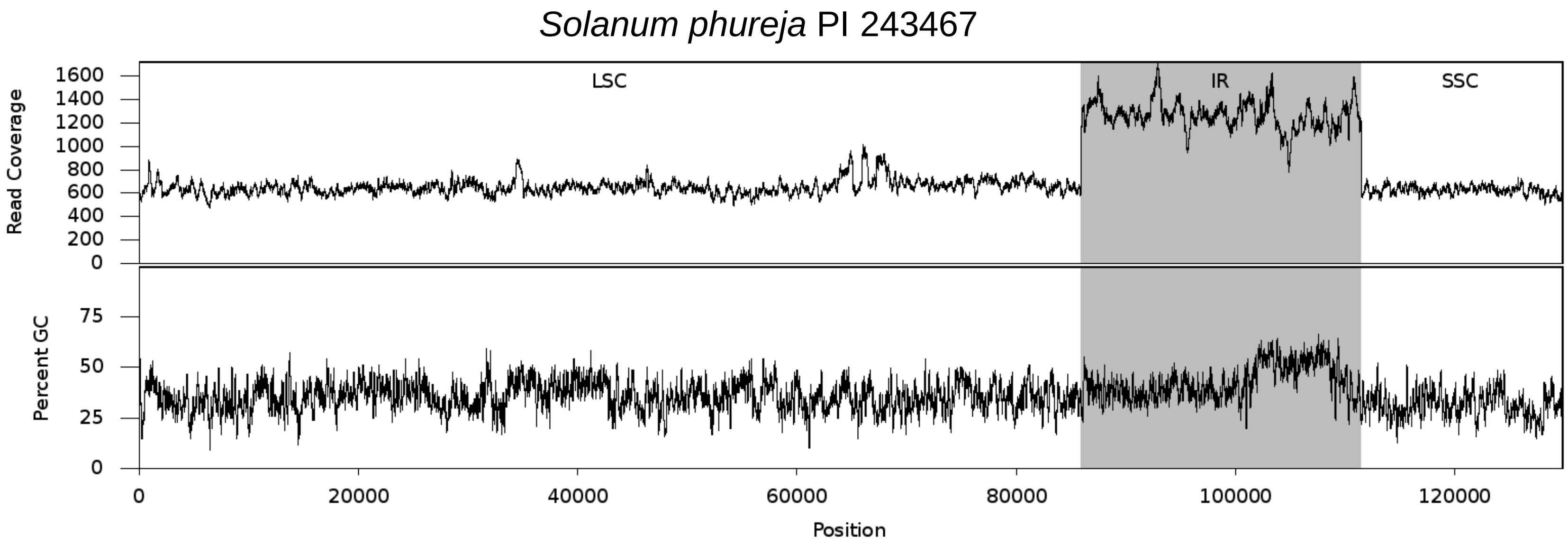
Solanum phureja PI 243467
LSC
IR
SSC
Read Coverage
Percent GC
Position
0
20000
40000
60000
80000
100000
120000

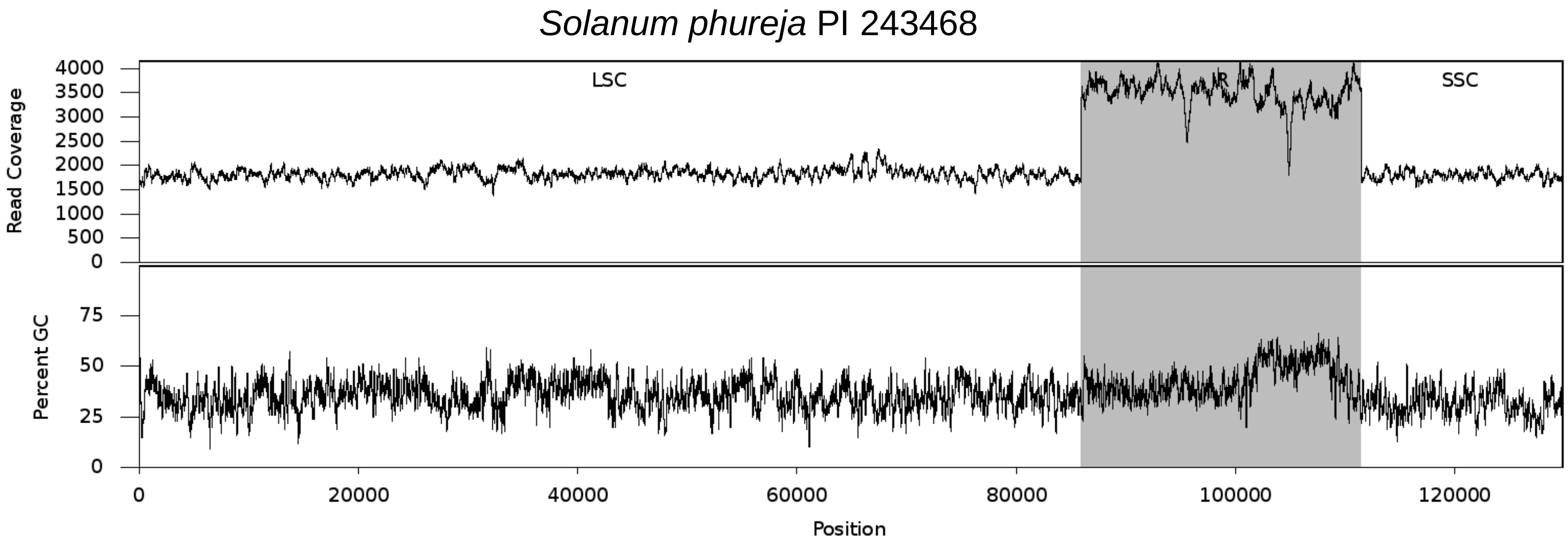
Solanum phureja PI 243468
LSC
IR
SSC
Read Coverage
Percent GC
Position
0
20000
40000
60000
80000
100000
120000

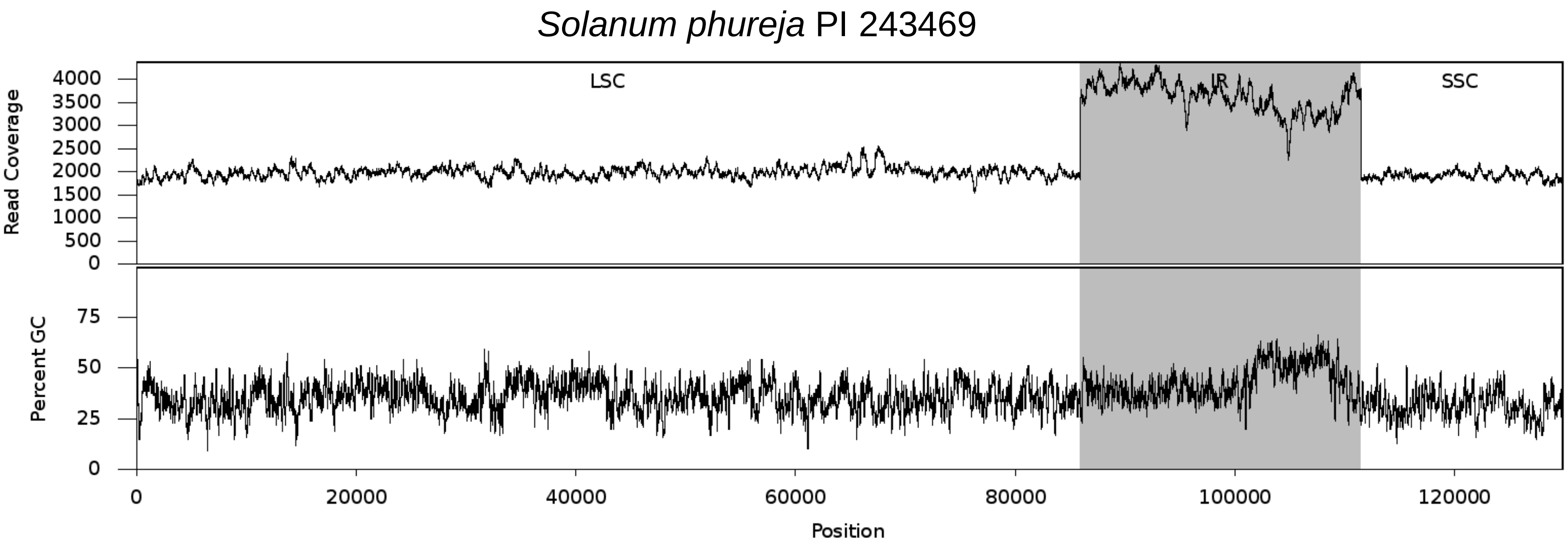
Solanum phureja PI 243469
LSC
IR
SSC
Read Coverage
4000
3500
3000
2500
2000
1500
1000
500
0
Percent GC
75
50
25
0
0
20000
40000
60000
80000
100000
120000
Position

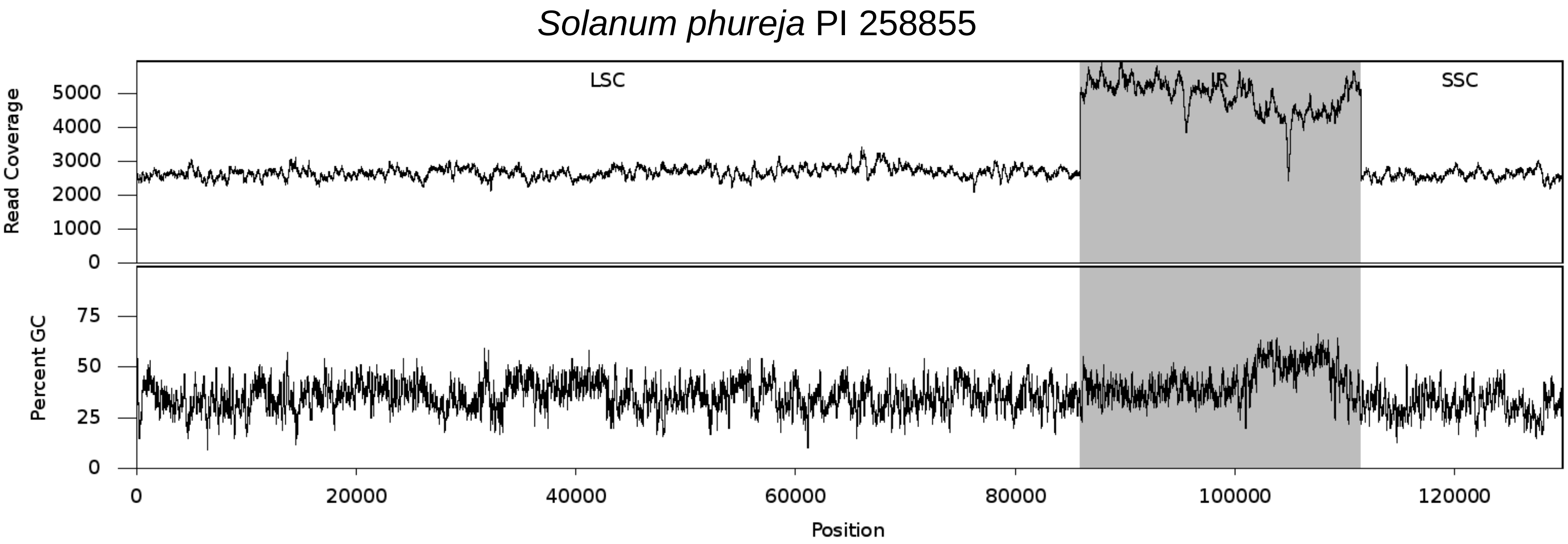
Solanum phureja PI 258855
LSC
IR
SSC
Read Coverage
5000
4000
3000
2000
1000
0
Percent GC
75
50
25
0
0
20000
40000
60000
80000
100000
120000
Position

Solanum pinnatisectum PI 253214

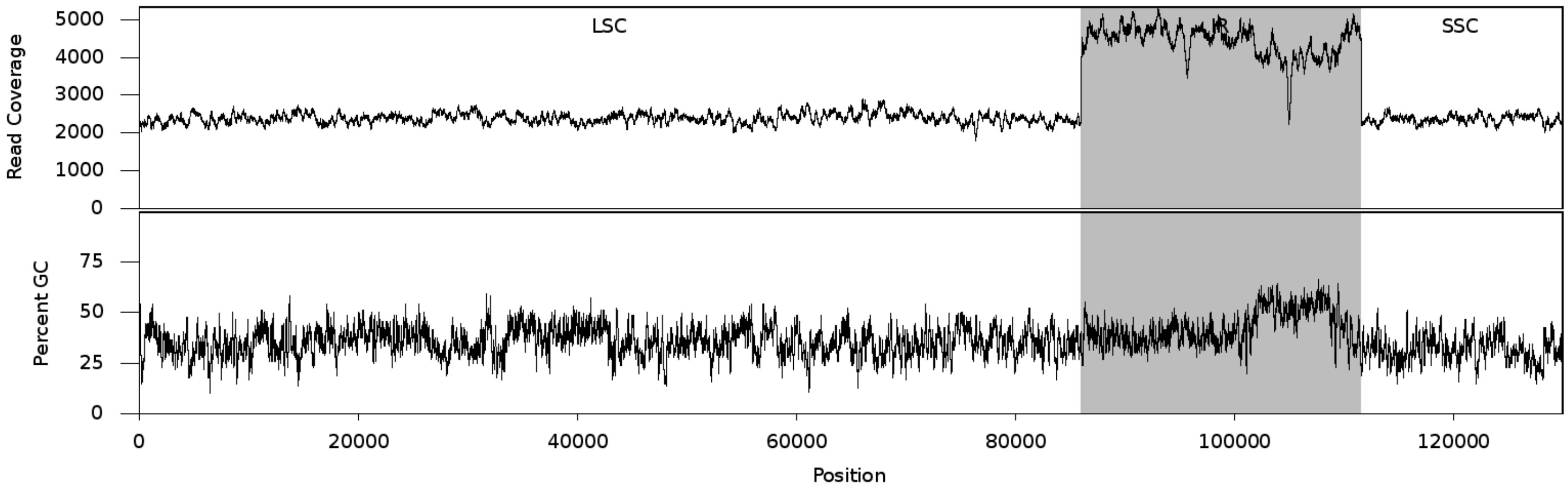

Solanum pinnatisectum PI 537023

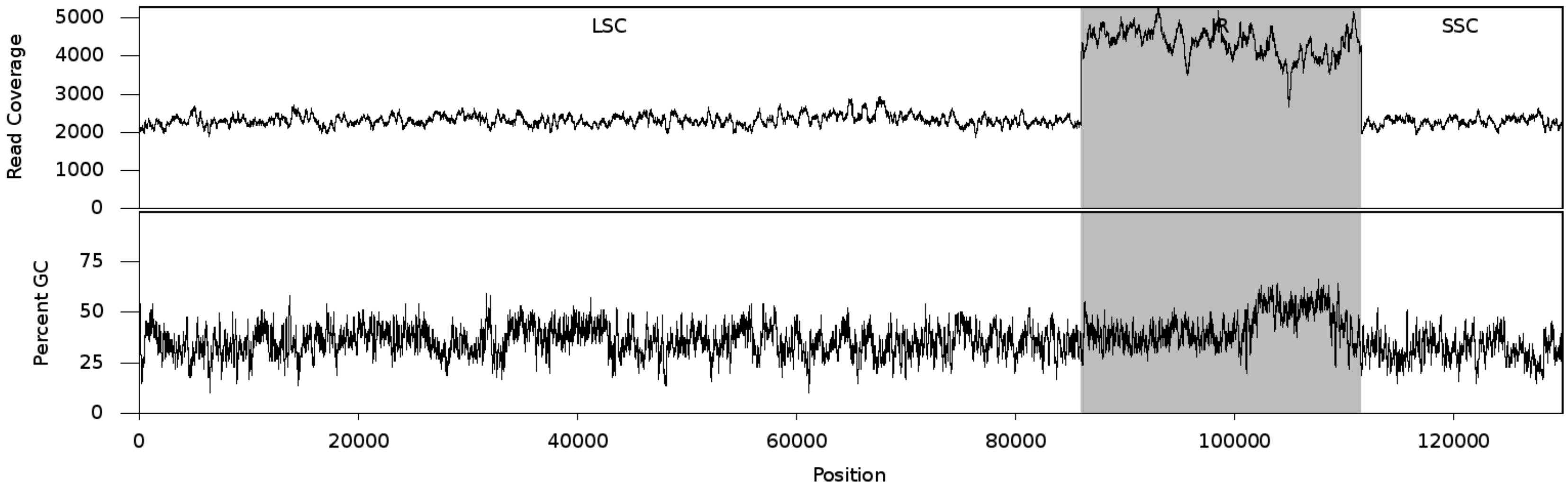

Solanum polyadenium PI 161728

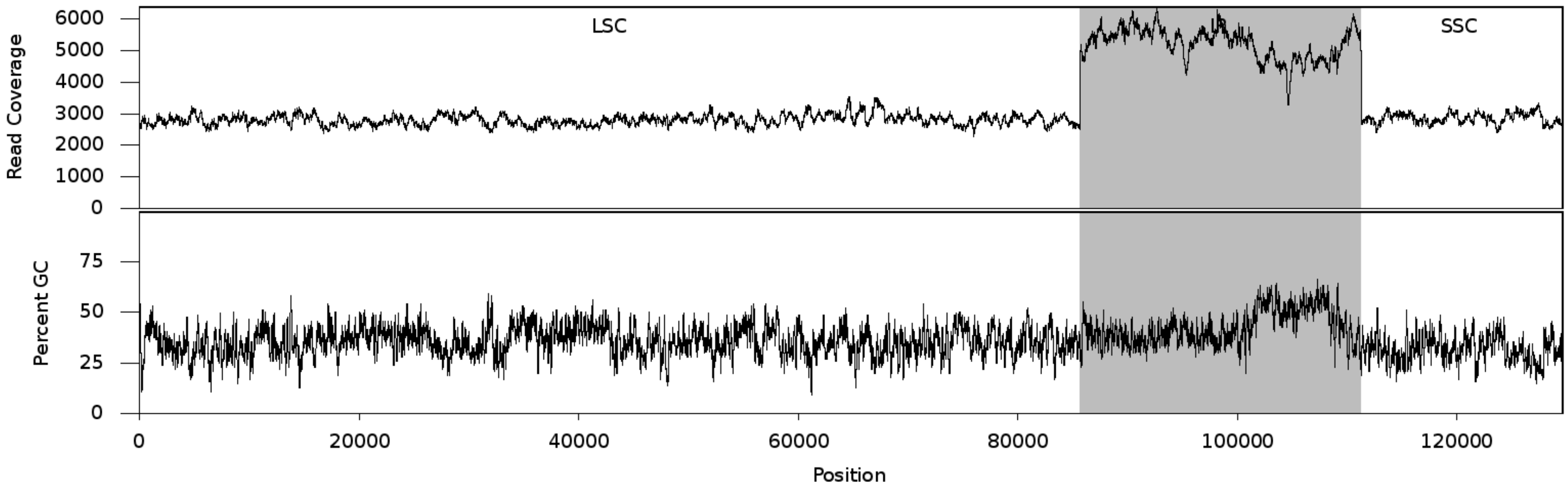

Solanum polyadenium PI 347770

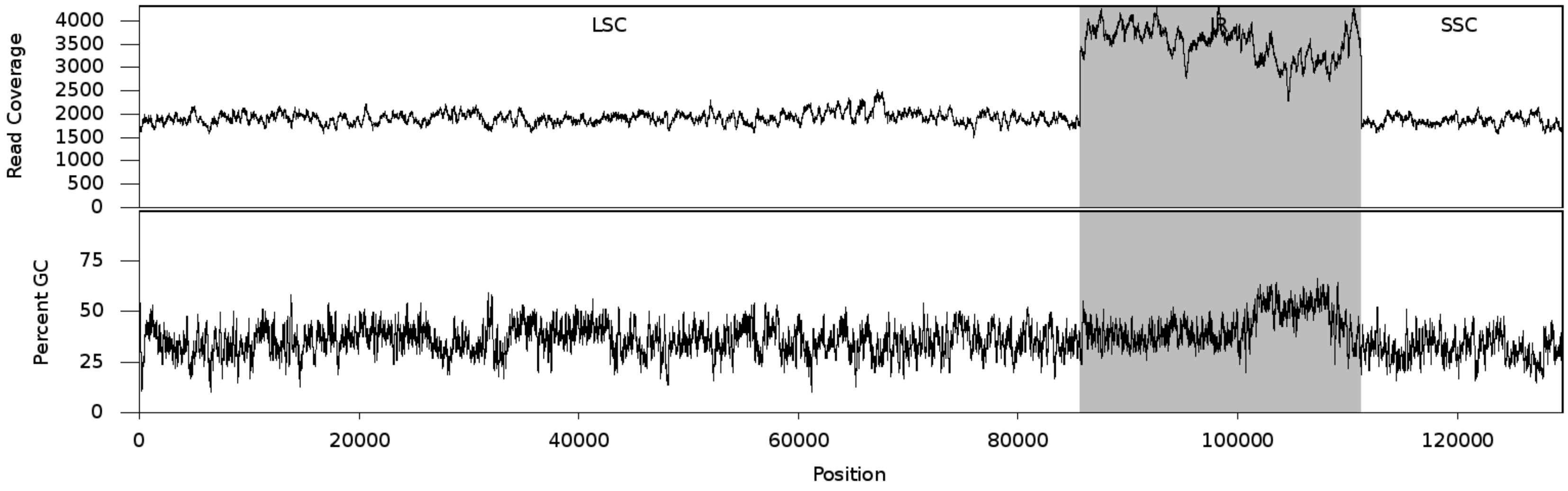

Solanum sogarandinum PI 230510

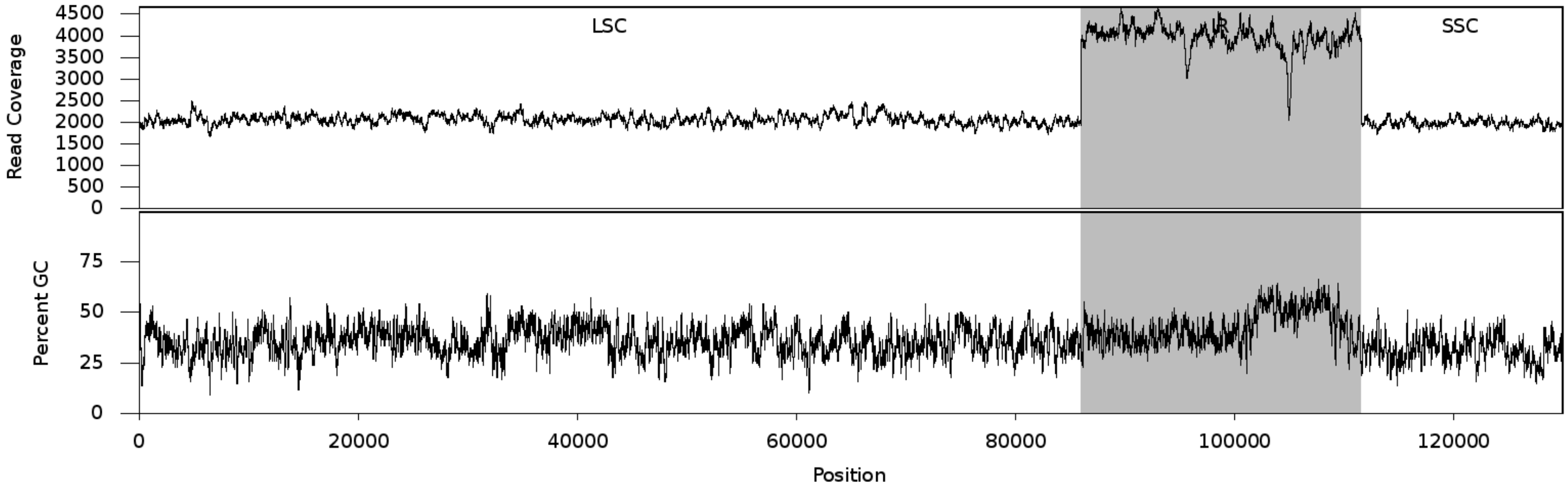

Solanum sogarandinum PI 365360

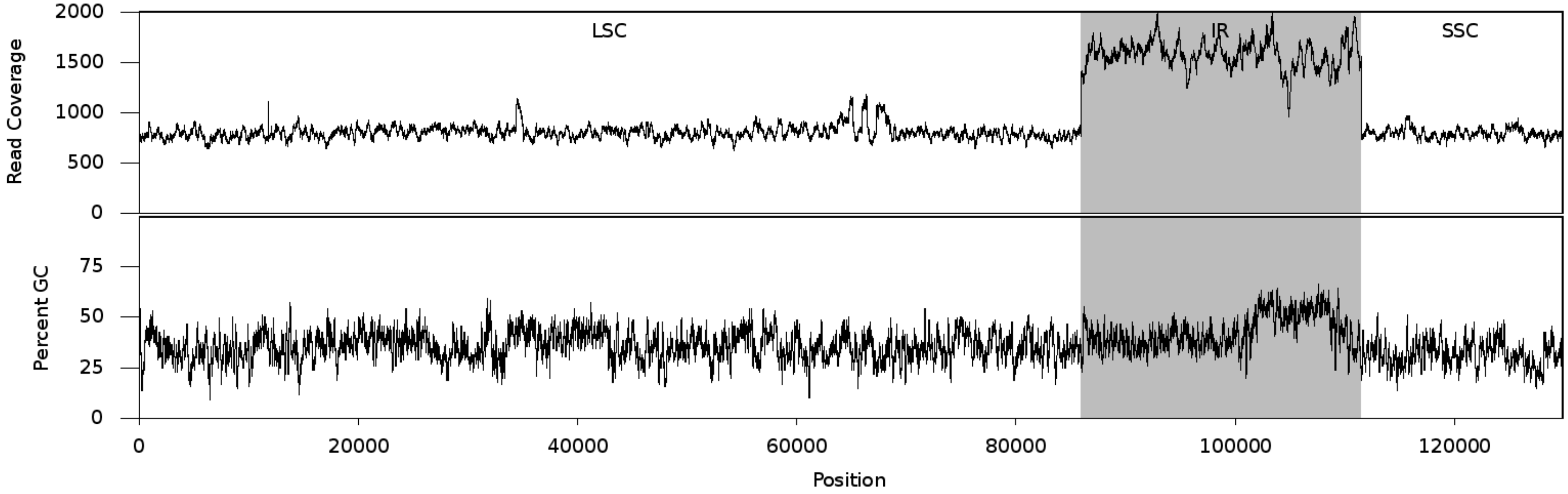

Solanum sparsipilum PI 246536

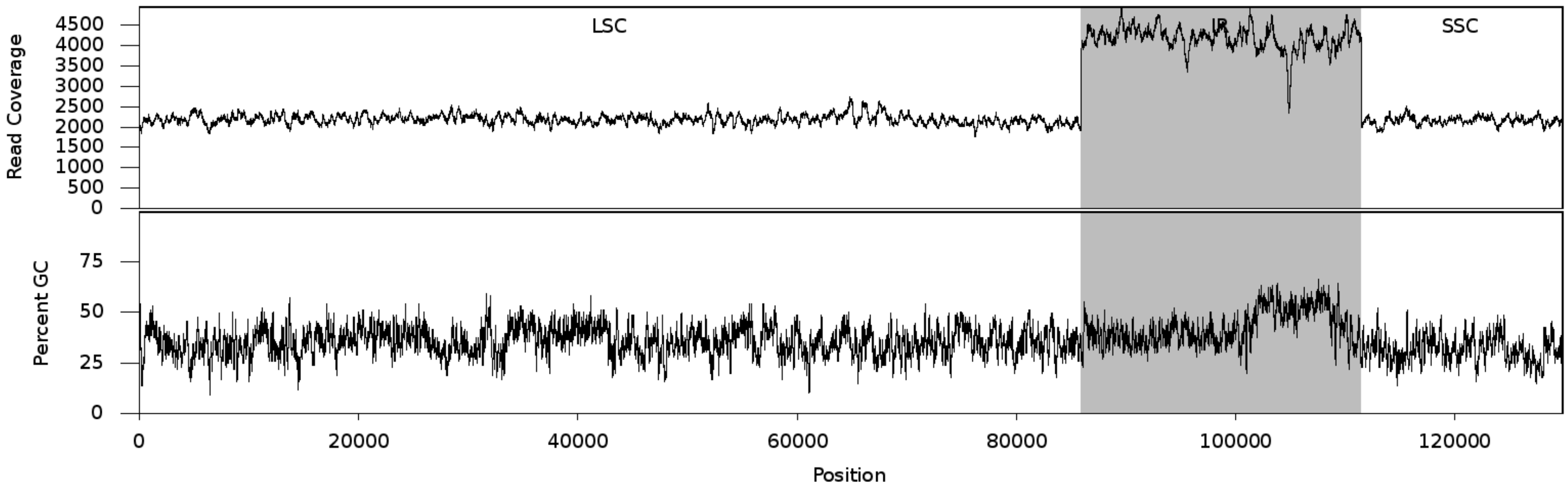

Solanum sparsipilum PI 473375

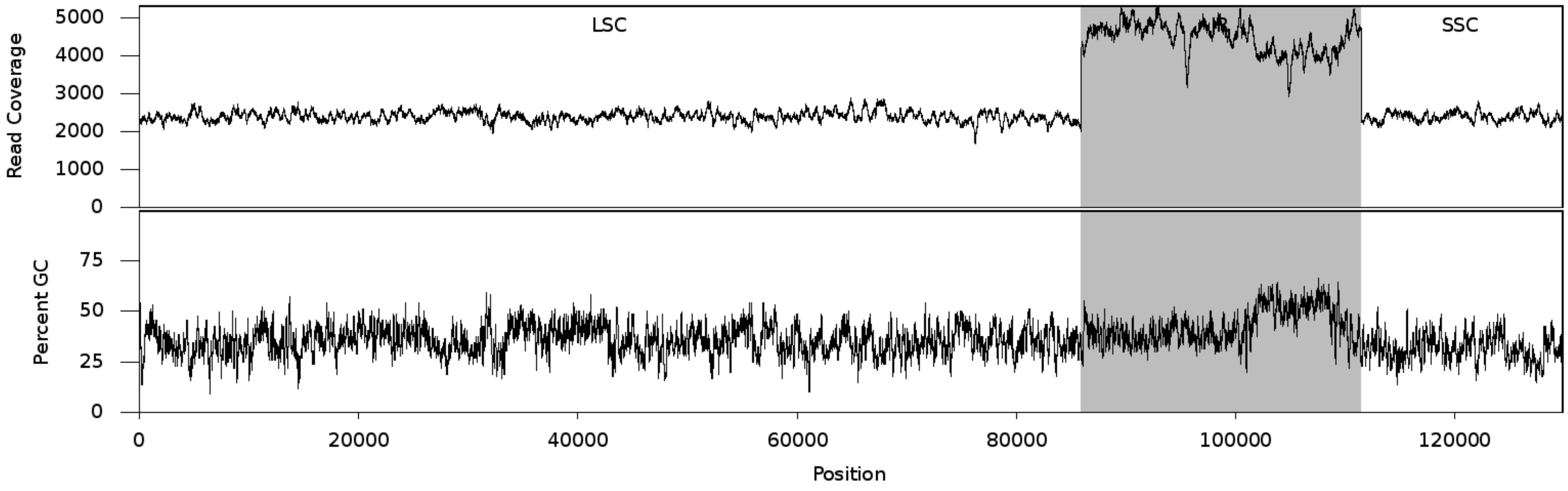

Solanum sparsipilum PI 473385

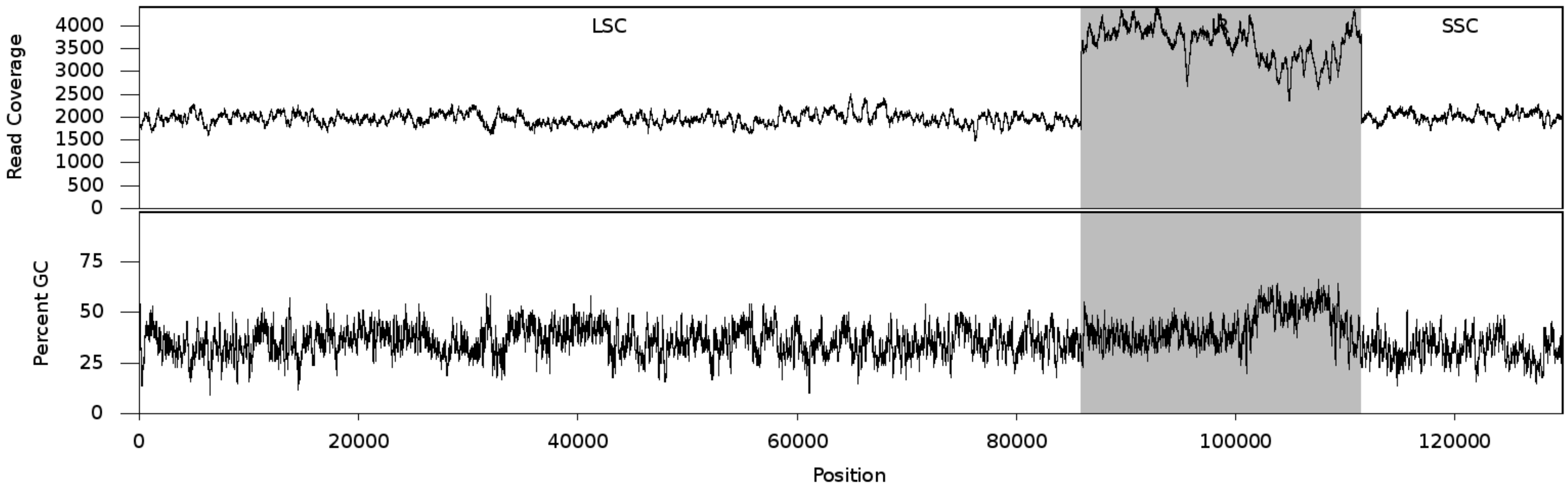

Solanum sparsipilum PI 498134

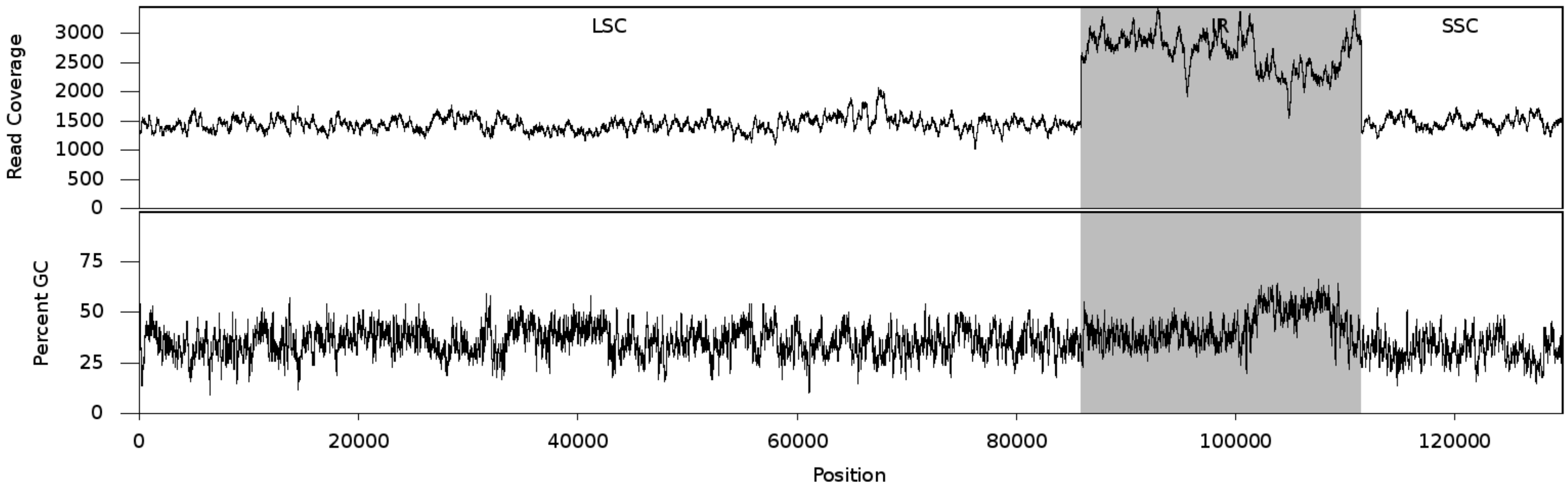

Solanum sparsipilum PI 498284

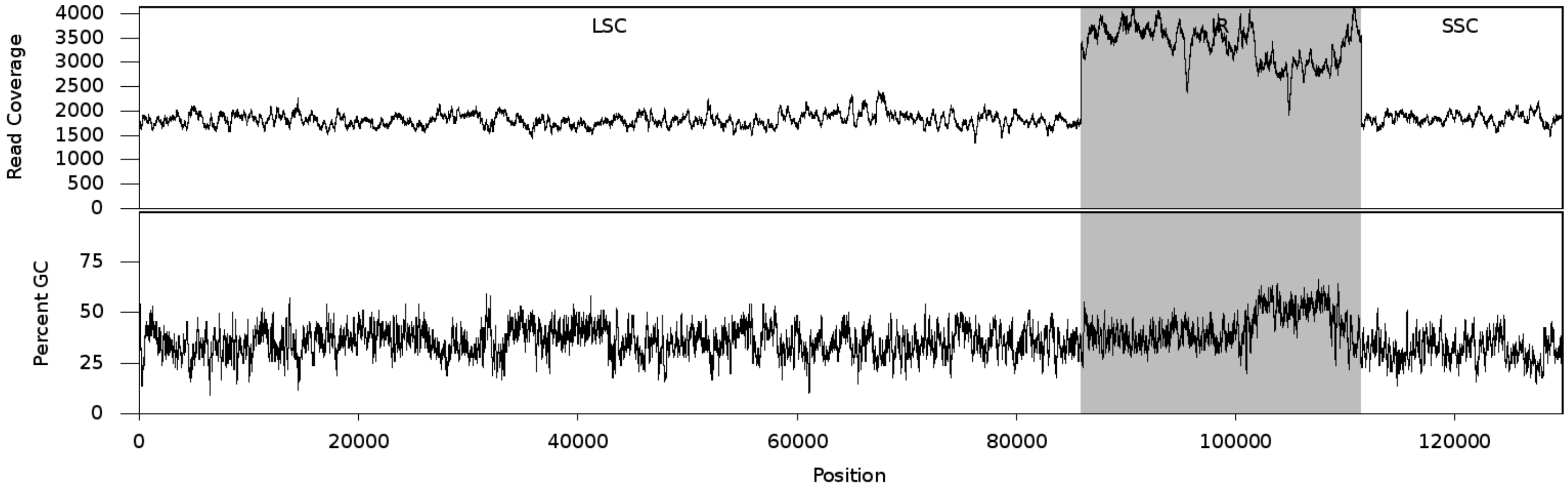

Solanum sparsipilum PI 498285

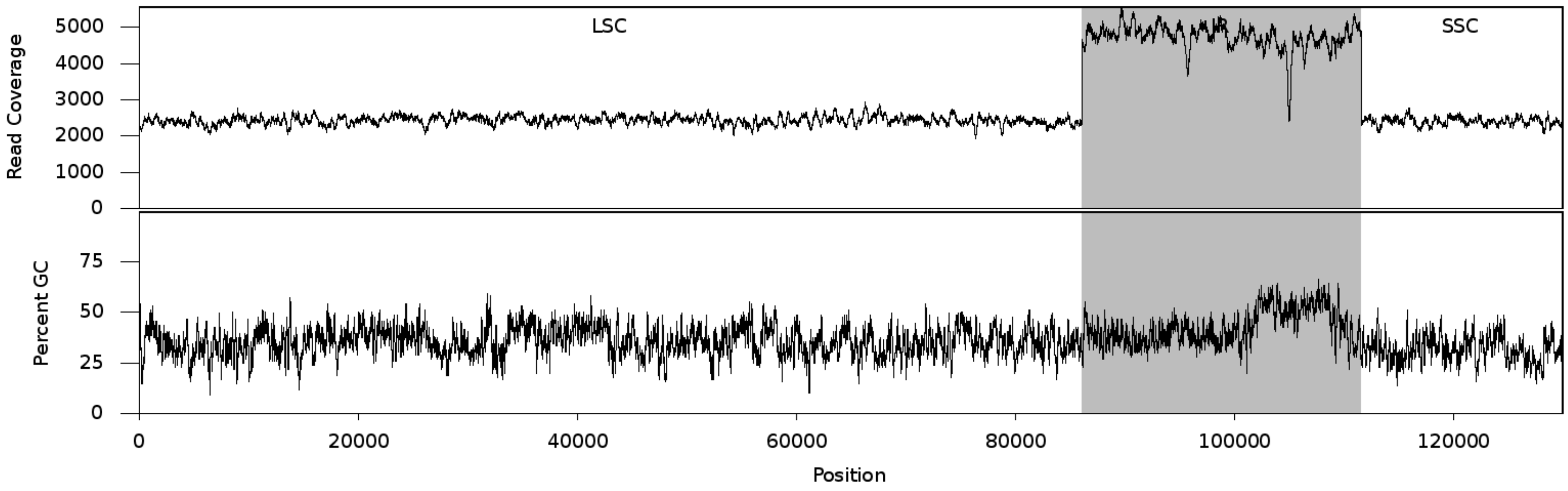

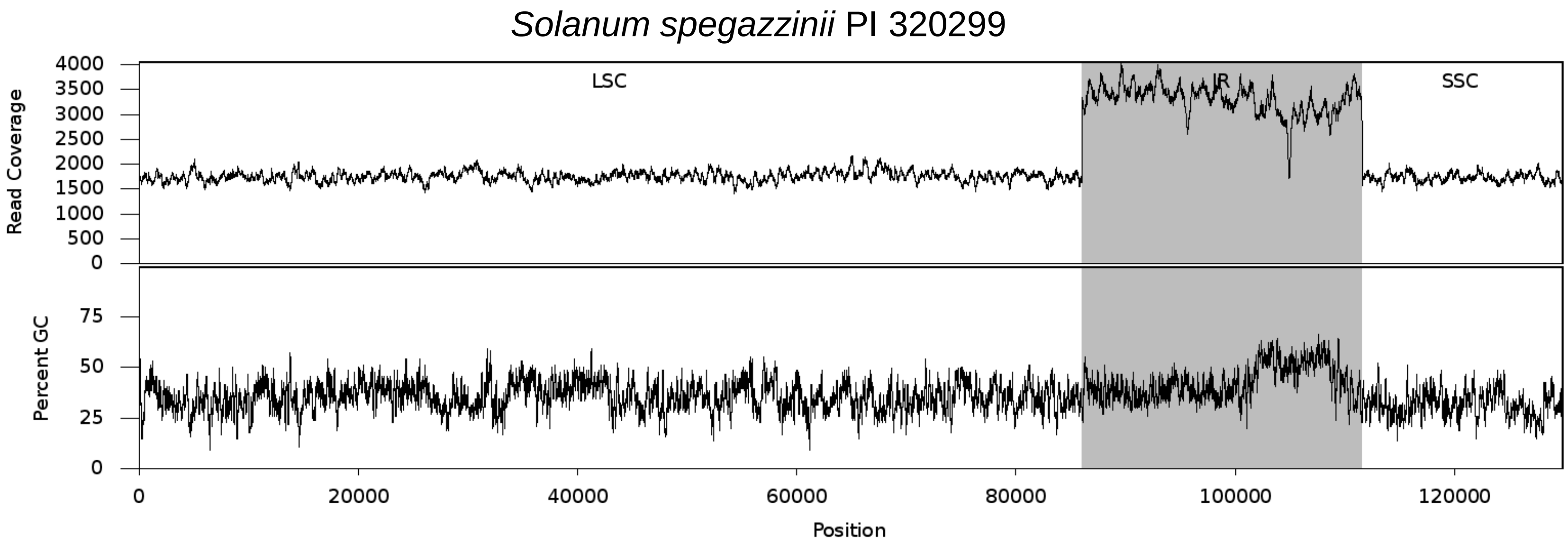
Solanum spegazzinii PI 320299
LSC
IR
SSC
Read Coverage
Percent GC
Position
0
500
1000
1500
2000
2500
3000
3500
4000
25
50
75
20000
40000
60000
80000
100000
120000

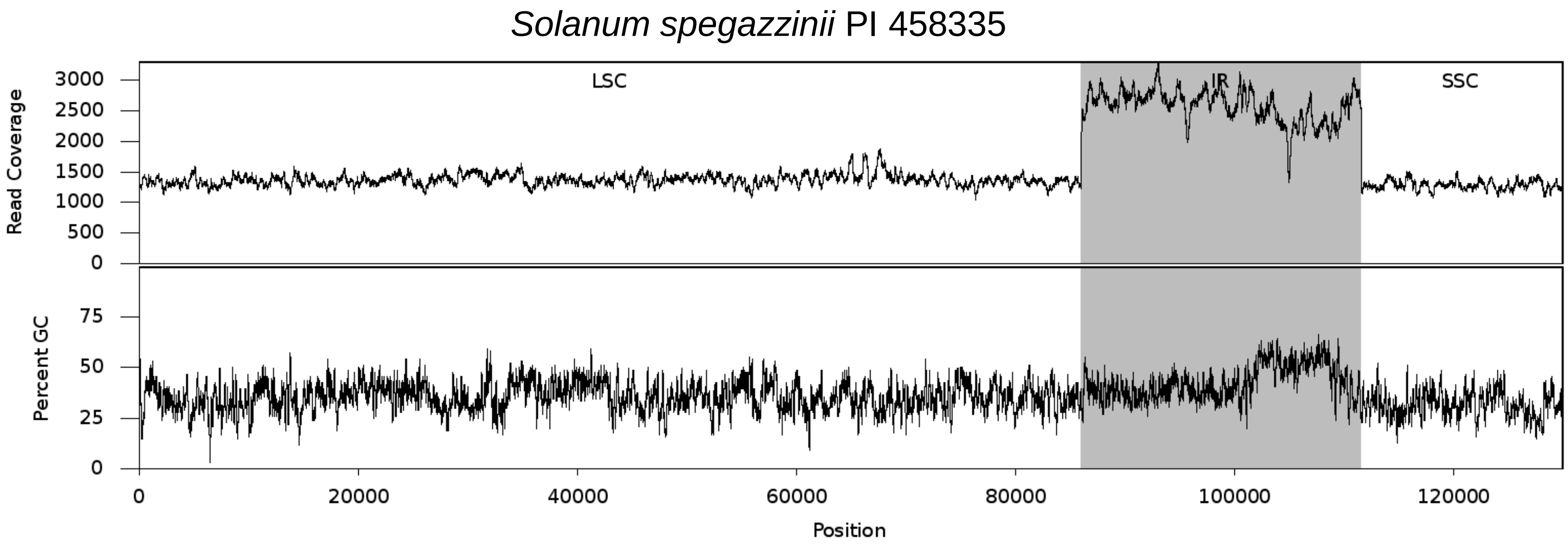
Solanum spegazzinii PI 458335
LSC
IR
SSC
Read Coverage
Percent GC
Position
0
500
1000
1500
2000
2500
3000
25
50
75
20000
40000
60000
80000
100000
120000

Solanum spegazzinii PI 458337

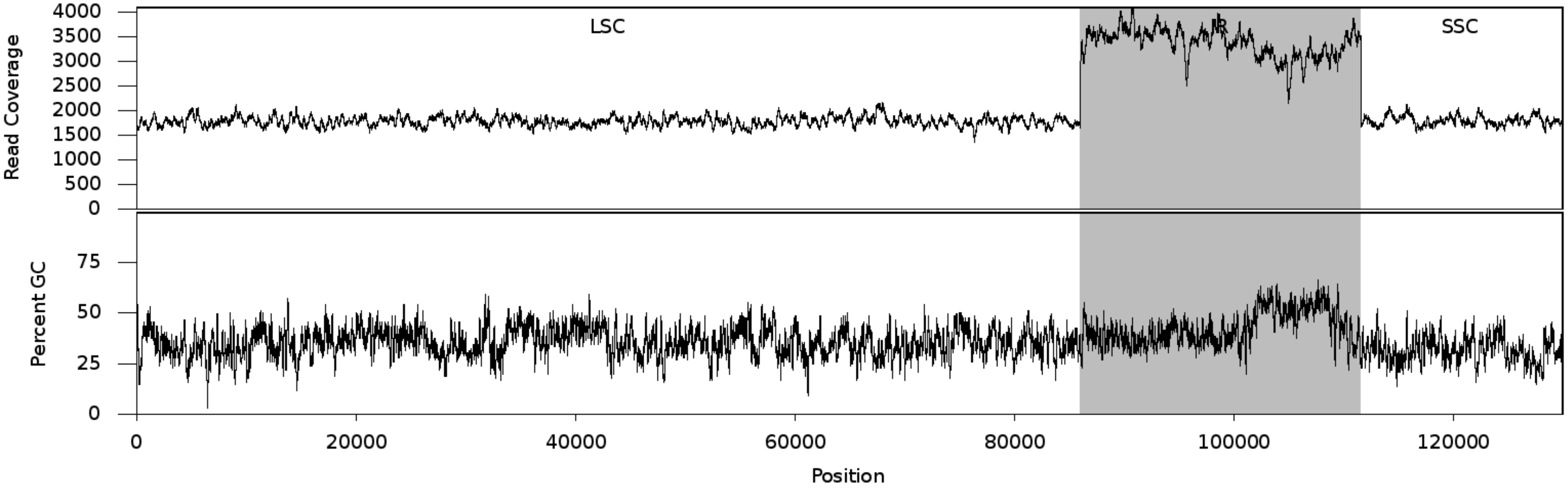

Solanum spegazzinii PI 472966

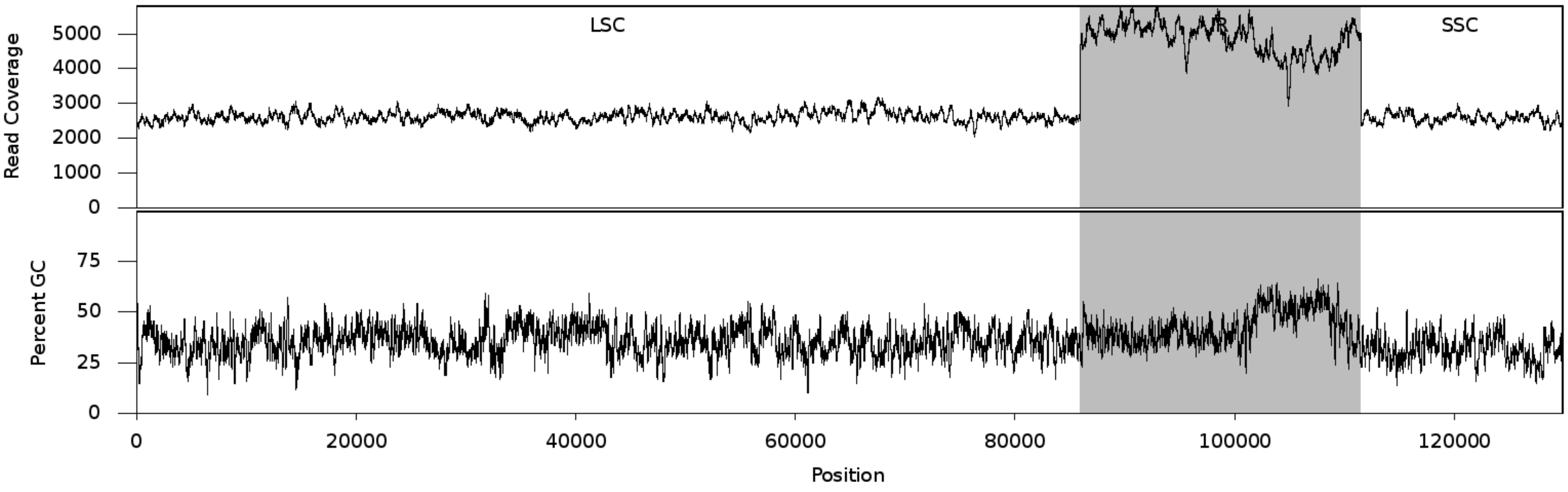

Solanum spegazzinii PI 472988

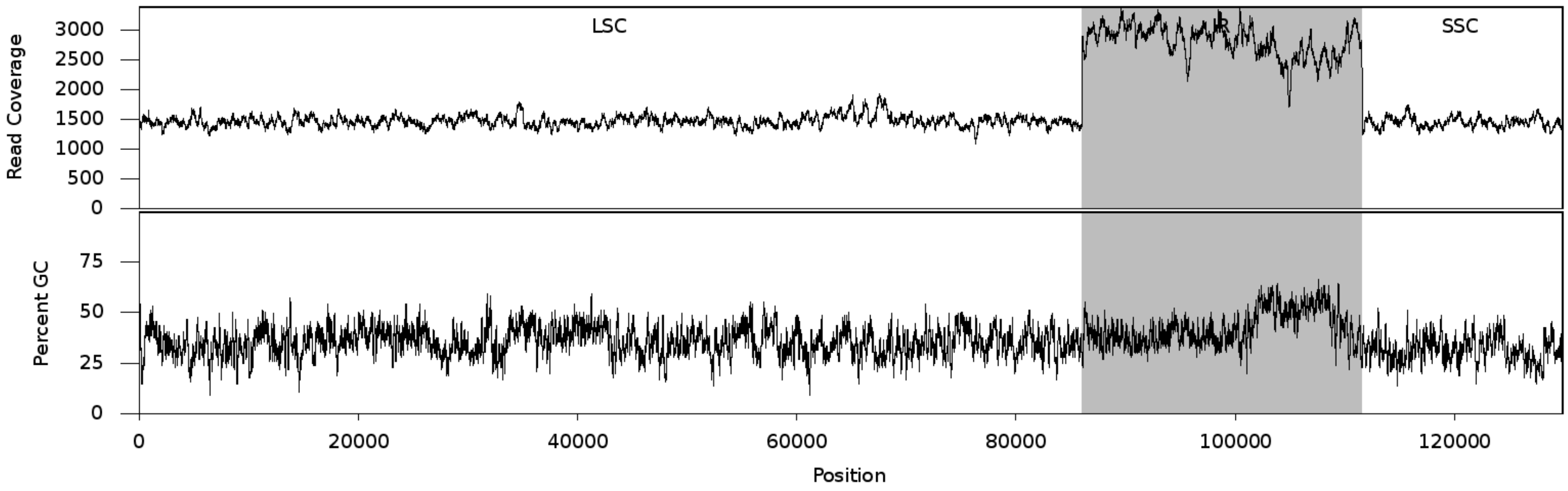

Solanum spegazzinii PI 472990

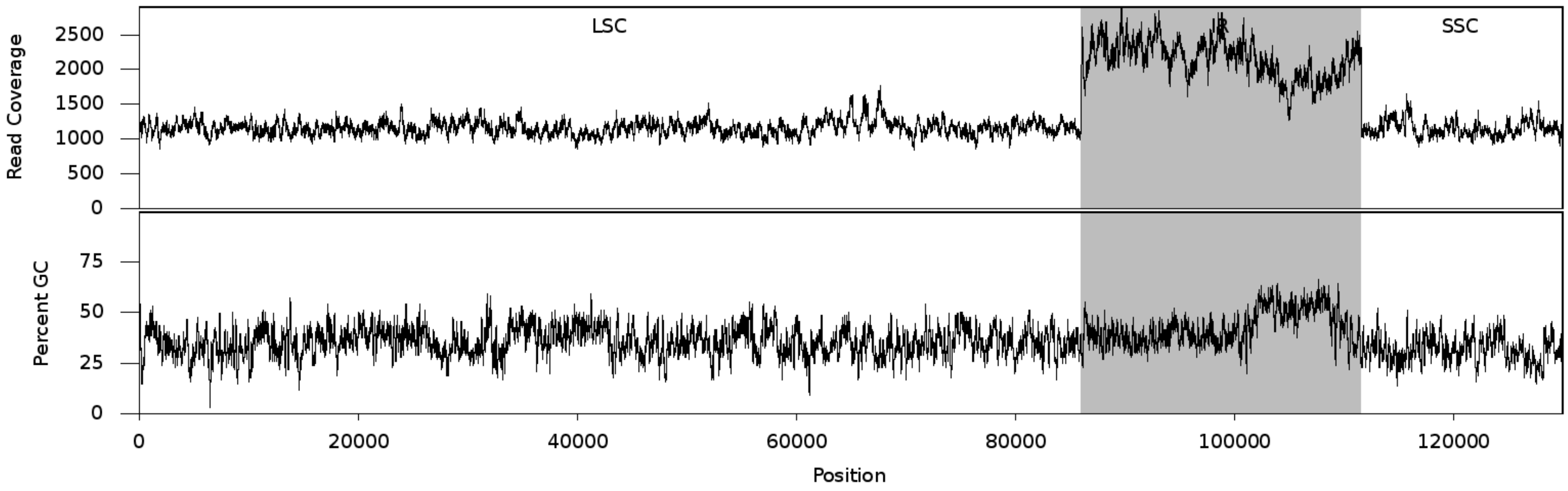

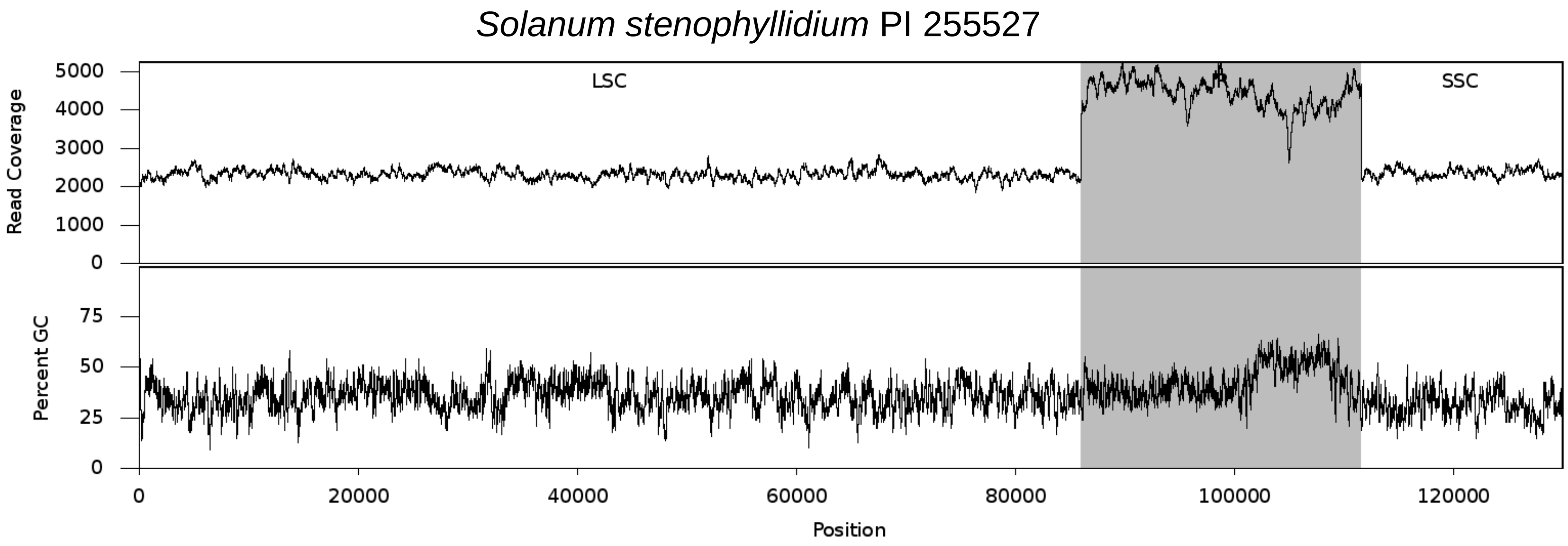
Solanum stenophyllidium PI 255527
Read Coverage
5000
4000
3000
2000
1000
0
LSC
SSC
Percent GC
75
50
25
0
0
20000
40000
60000
80000
100000
120000
Position

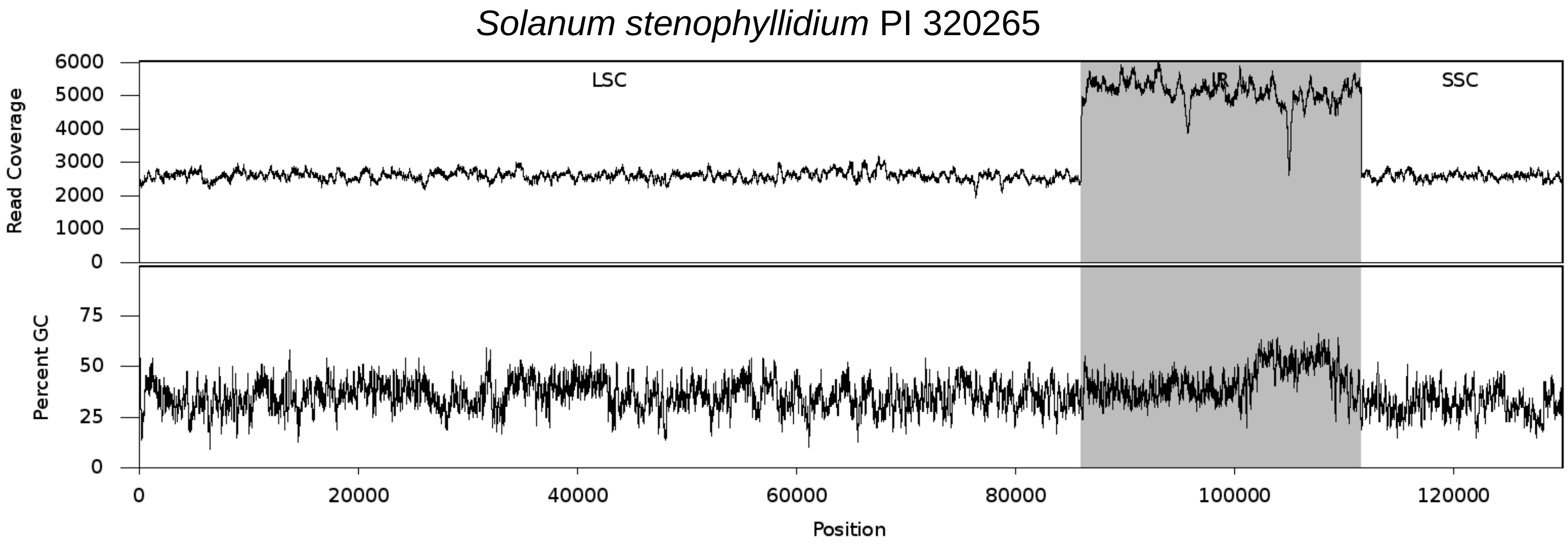
Solanum stenophyllidium PI 320265
Read Coverage
6000
5000
4000
3000
2000
1000
0
LSC
IR
SSC
Percent GC
75
50
25
0
0
20000
40000
60000
80000
100000
120000
Position

Solanum stenophyllidium PI 558460

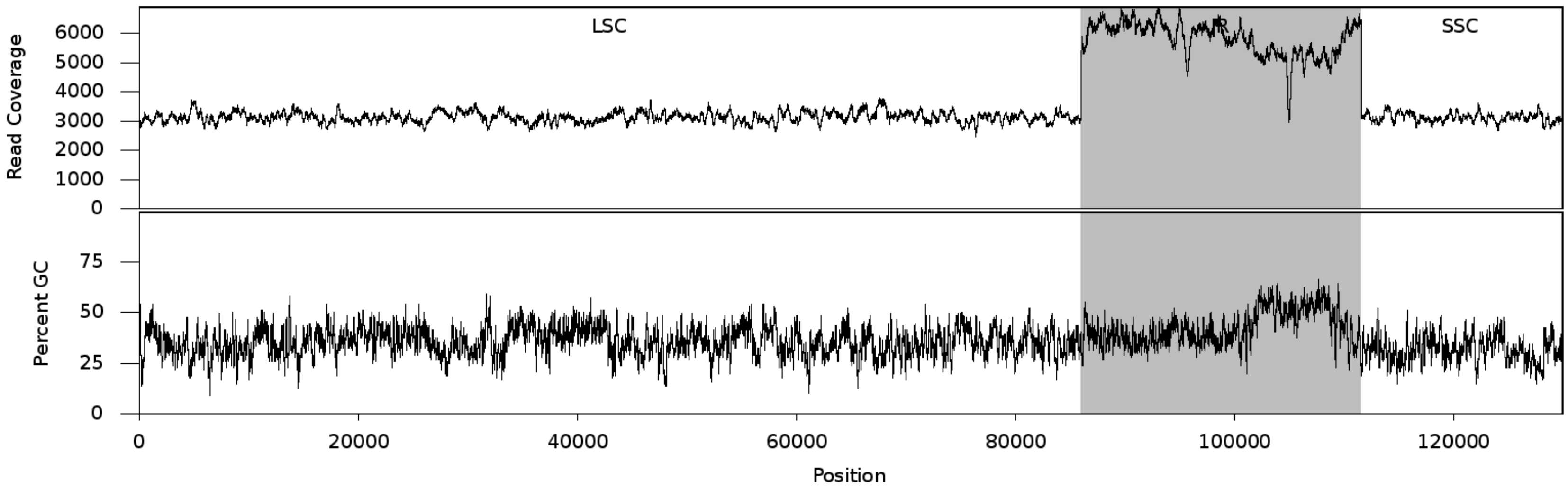

Solanum stenotomum PI 195204

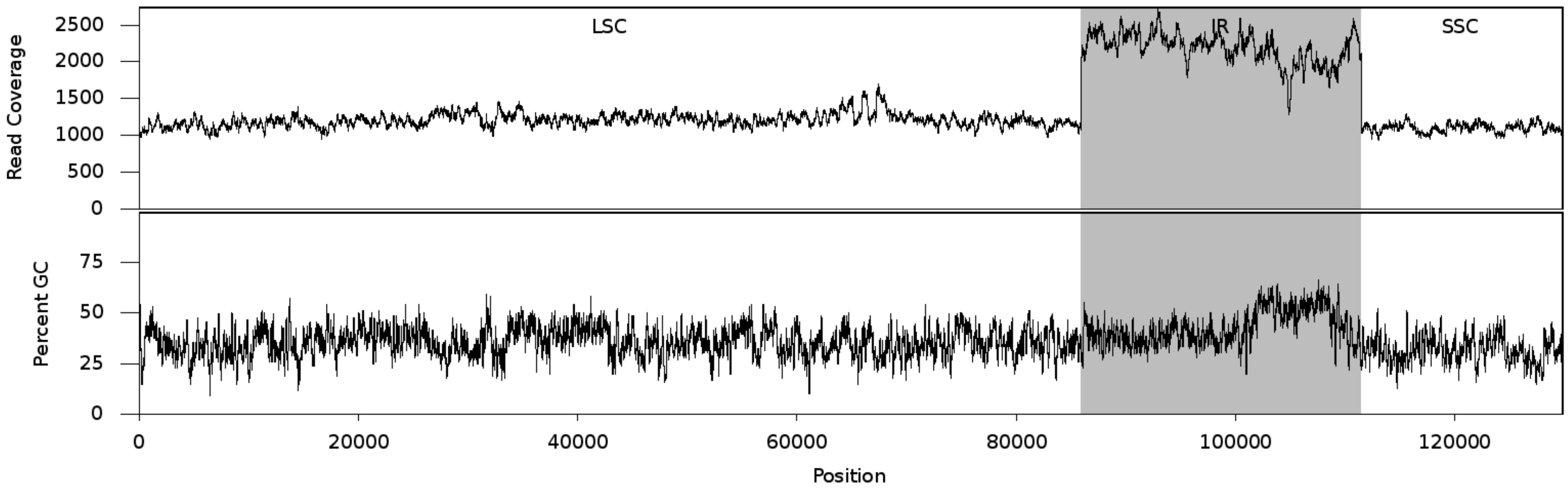

Solanum stenotomum PI 205527

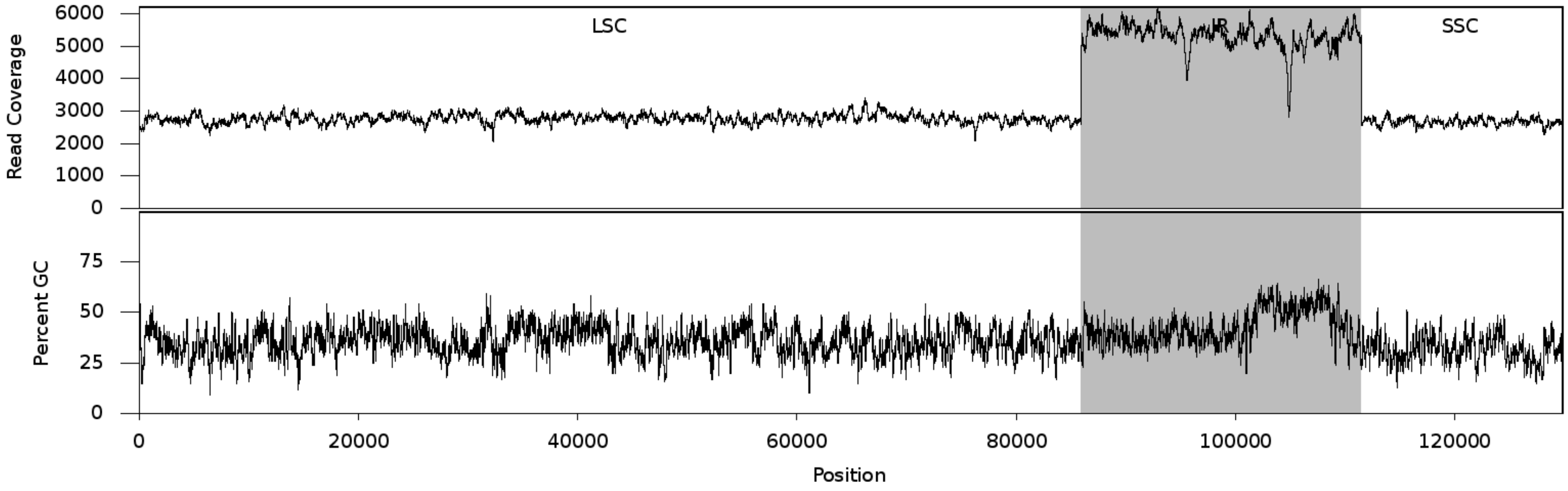

Solanum stenotomum PI 230512

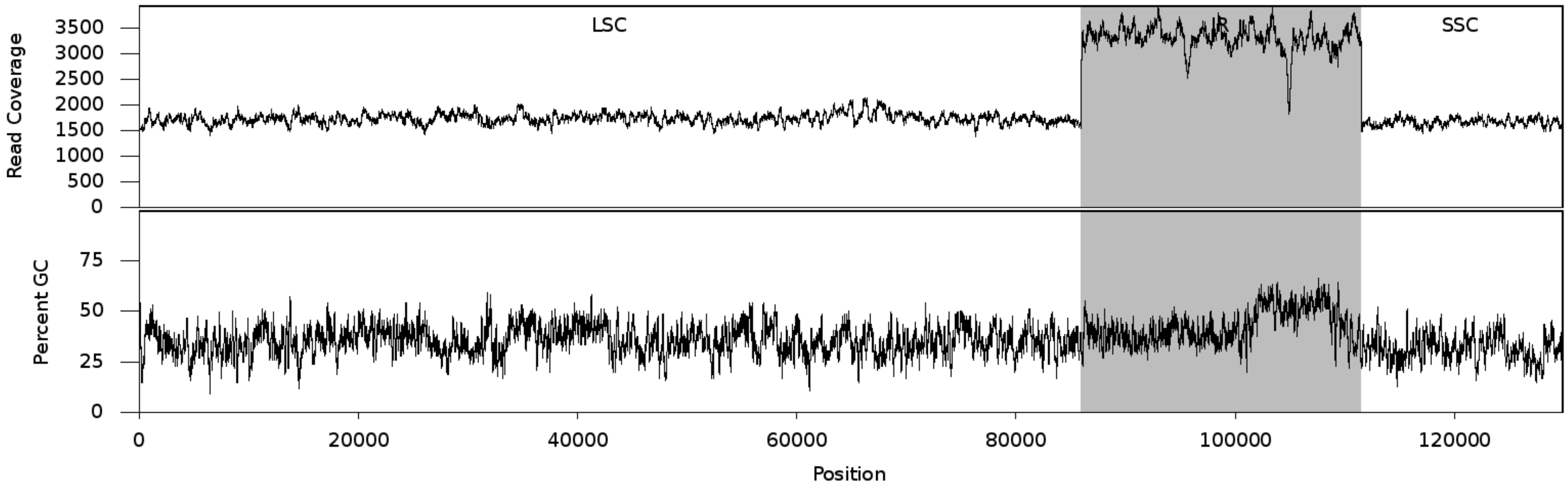

Solanum stenotomum PI 230513

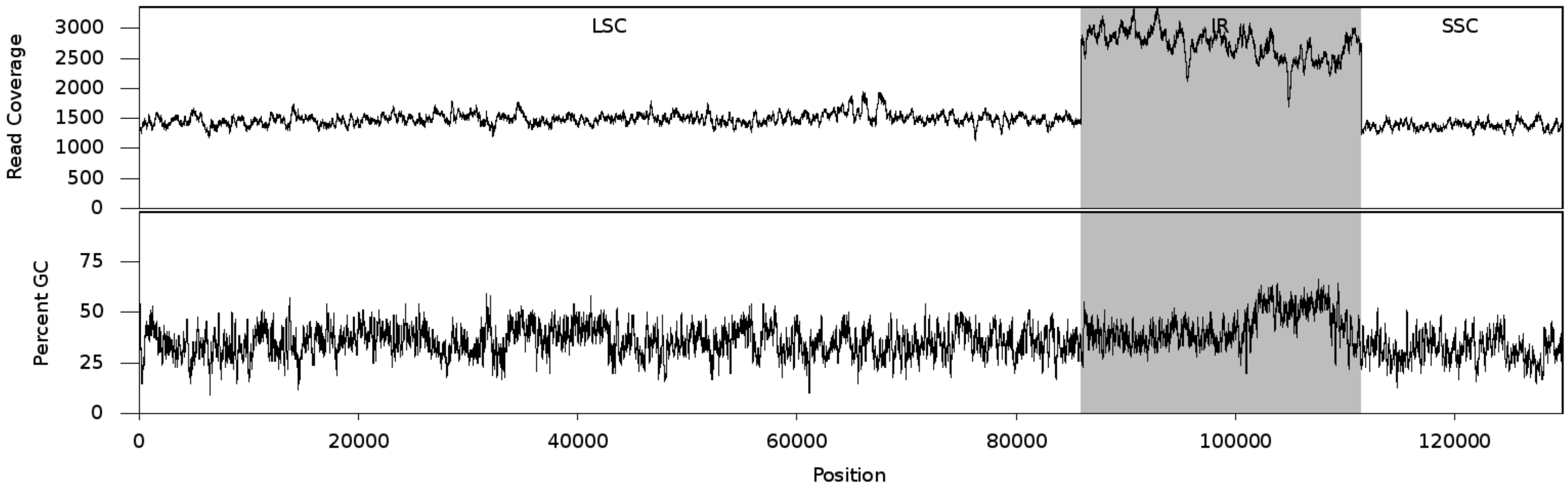

Solanum stenotomum PI 234011

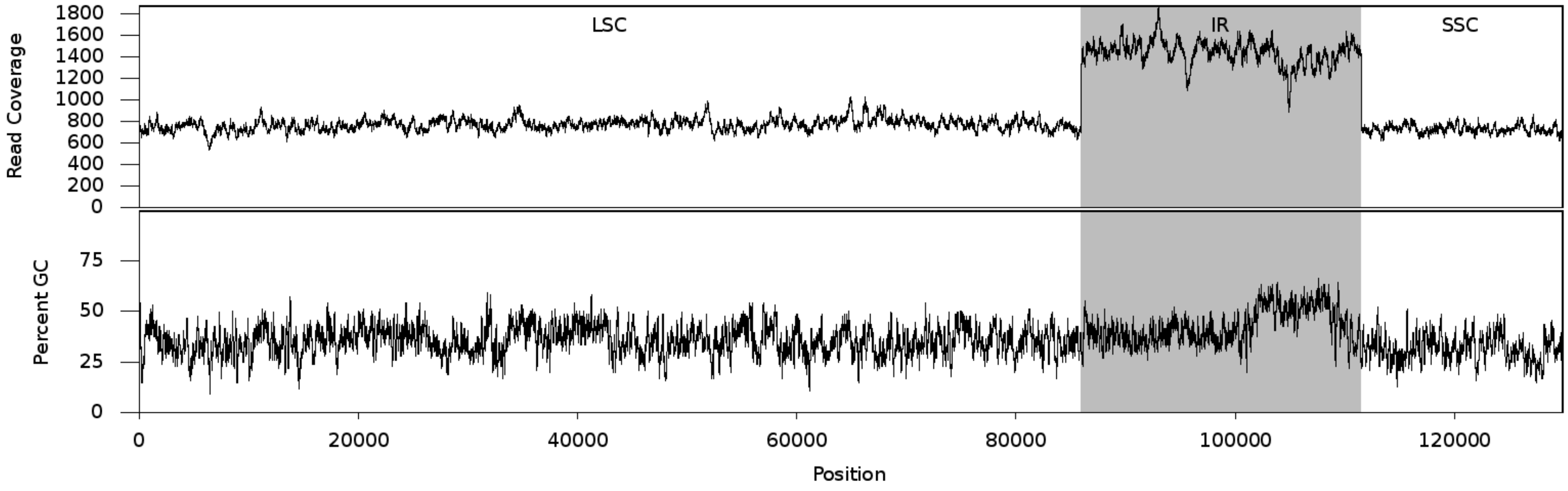

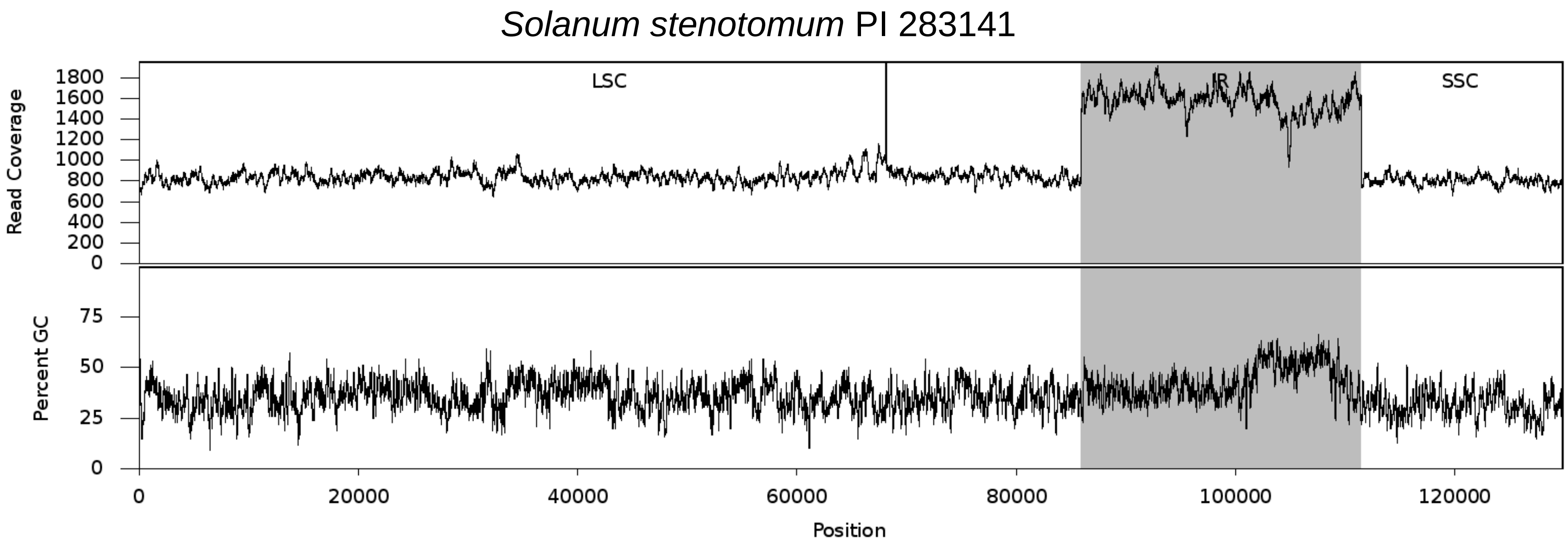

Solanum stenotomum PI 283141
Read Coverage
1800
1600
1400
1200
1000
800
600
400
200
0
LSC
IR
SSC
Percent GC
75
50
25
0
0
20000
40000
60000
80000
100000
120000
Position

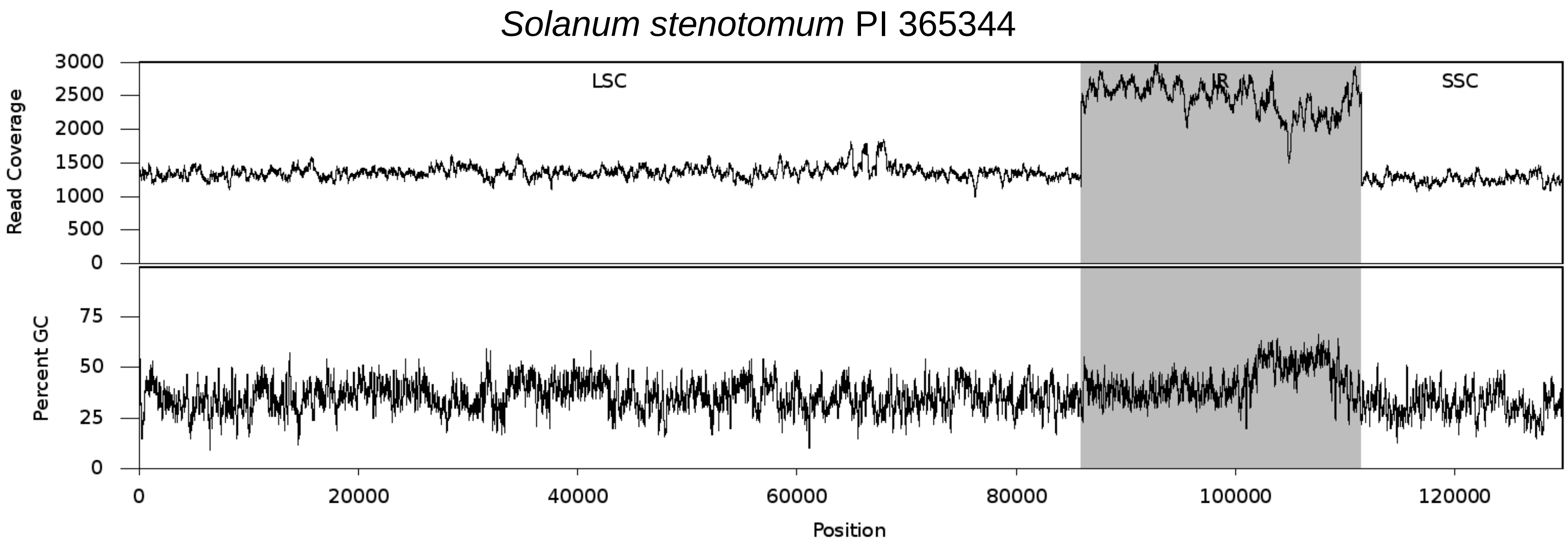

Solanum stenotomum PI 365344
Read Coverage
3000
2500
2000
1500
1000
500
0
LSC
IR
SSC
Percent GC
75
50
25
0
0
20000
40000
60000
80000
100000
120000
Position

Solanum tarijense PI 217457

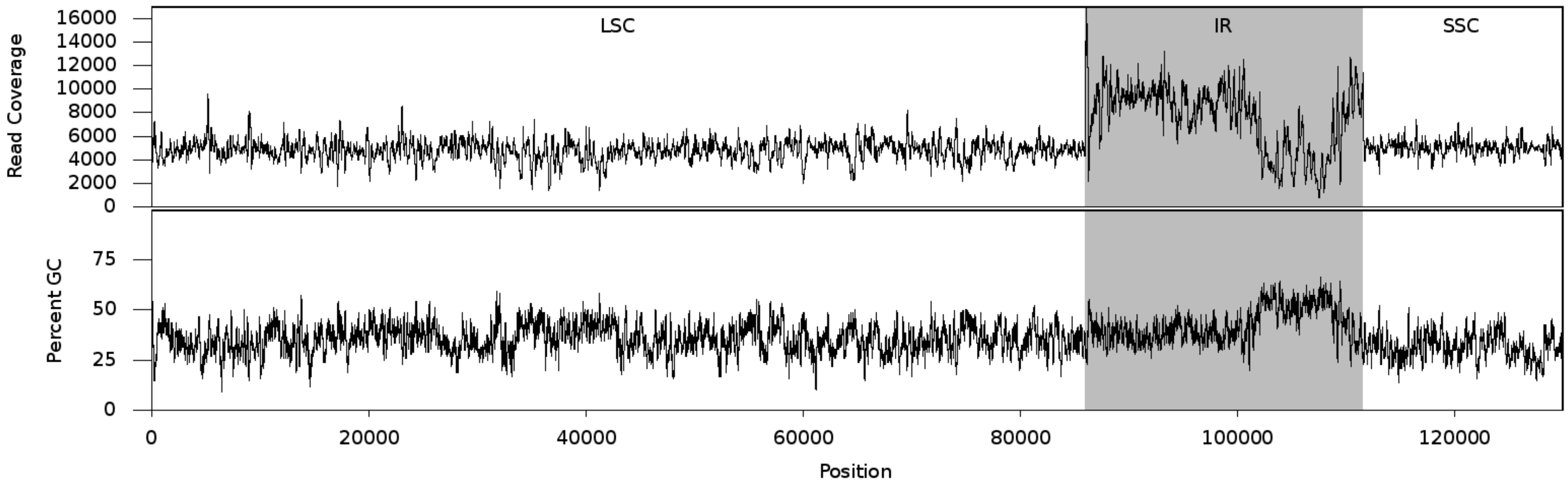

Solanum tarijense PI 414152

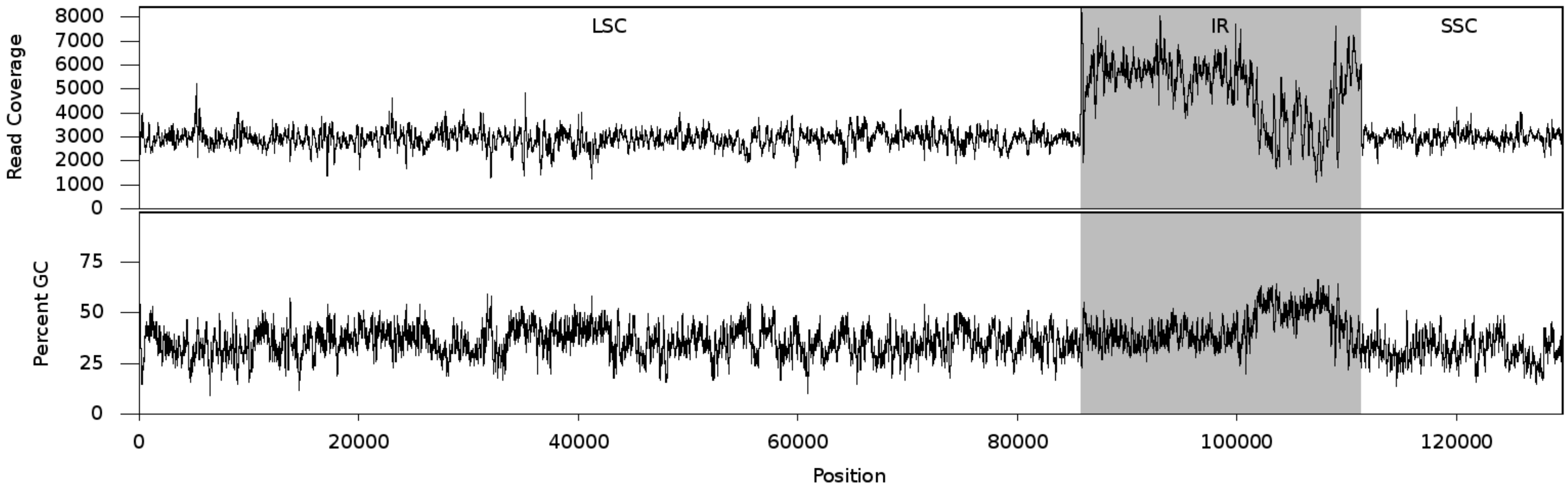

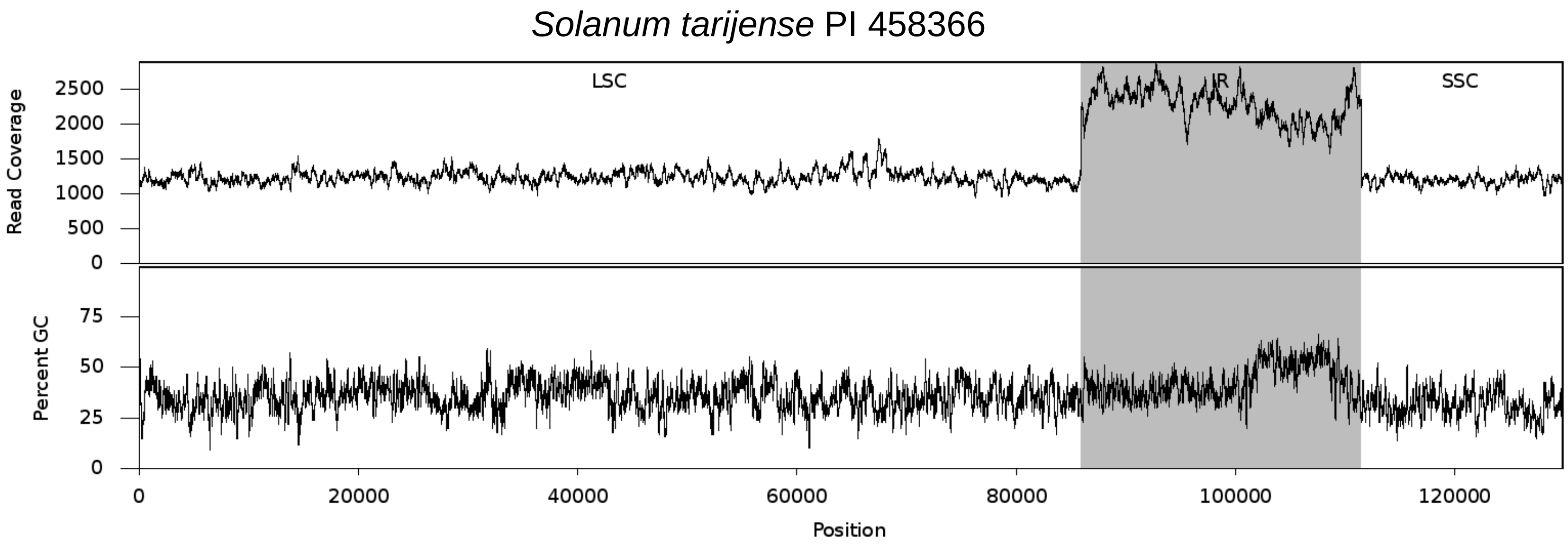
Solanum tarijense PI 458366
LSC
IR
SSC
Read Coverage
Percent GC
Position

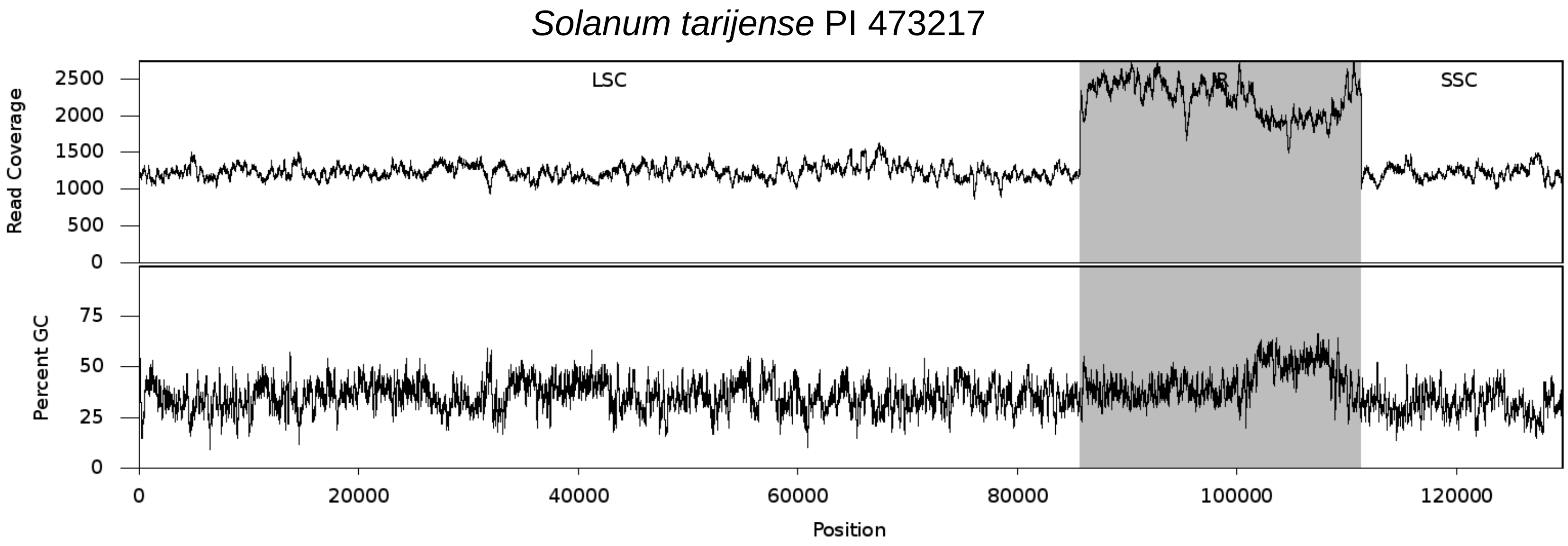
Solanum tarijense PI 473217
LSC
IR
SSC
Read Coverage
Percent GC
Position

Solanum tarijense PI 473218

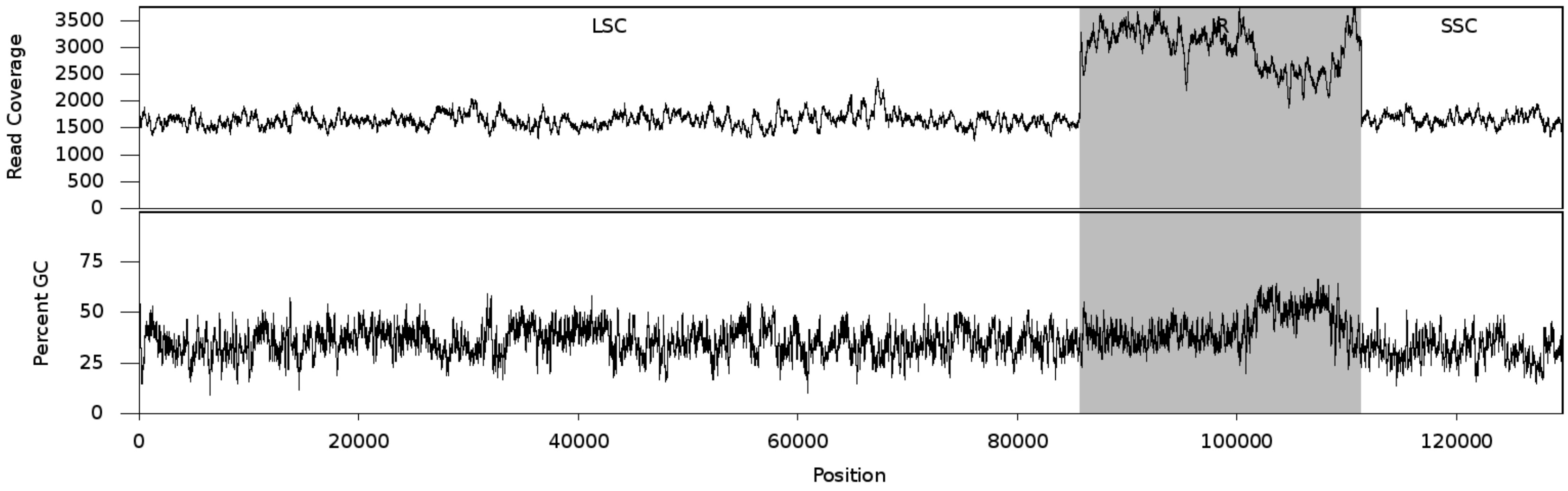

Solanum tuberosum PI 320364

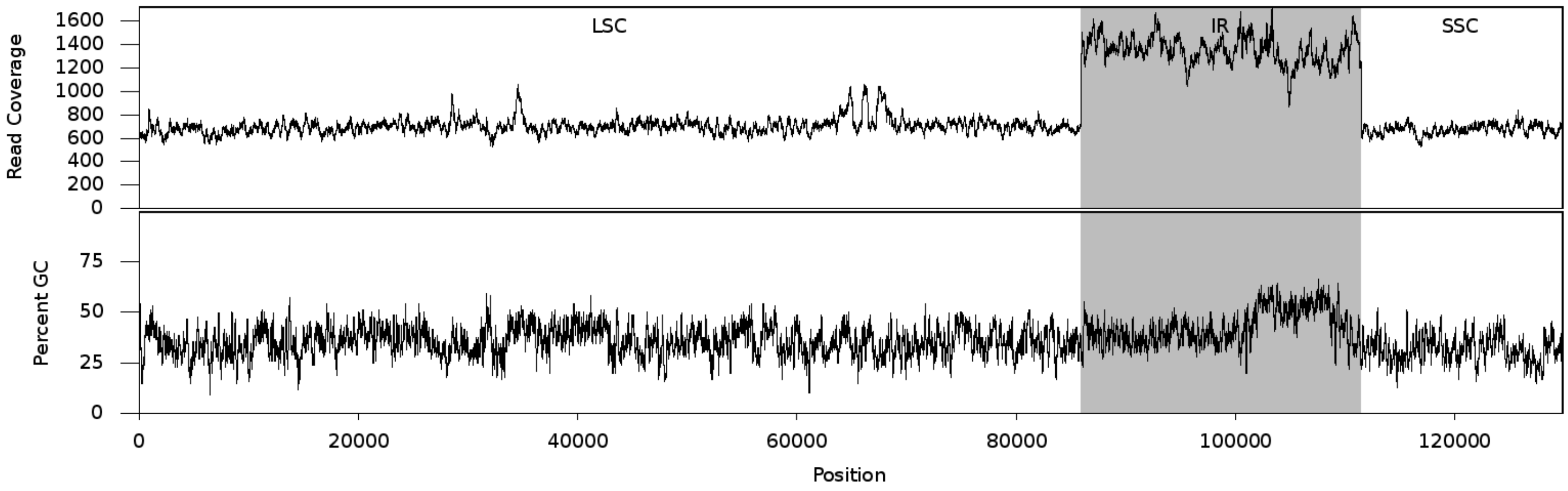

Solanum vernei PI 320332

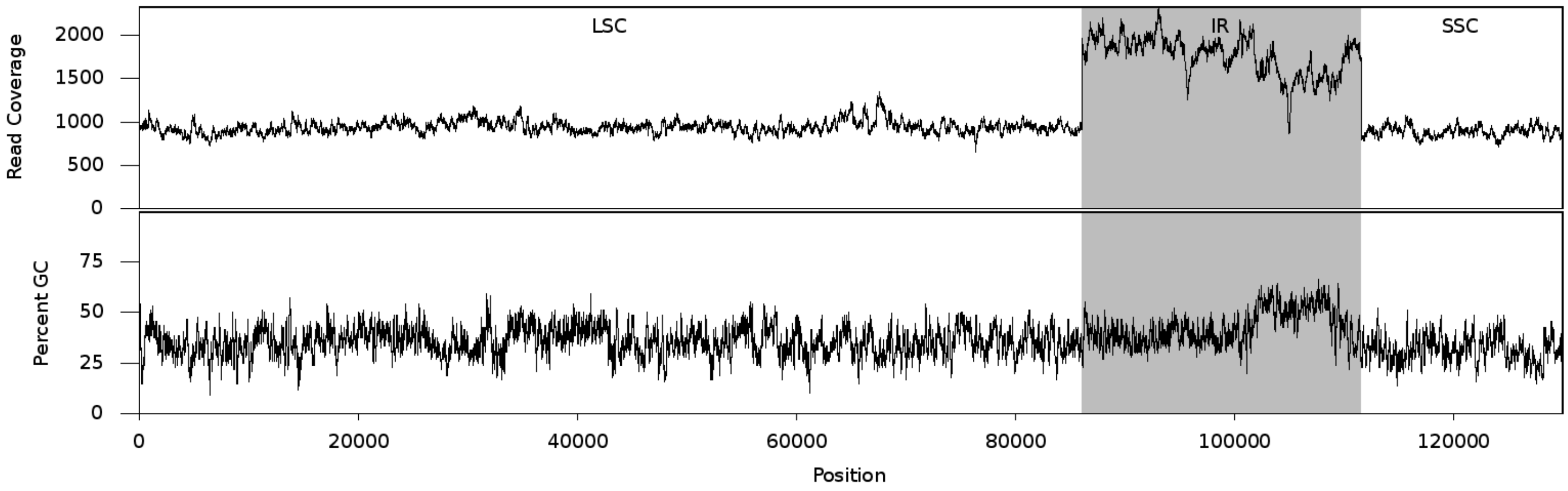

Solanum vernei PI 320333

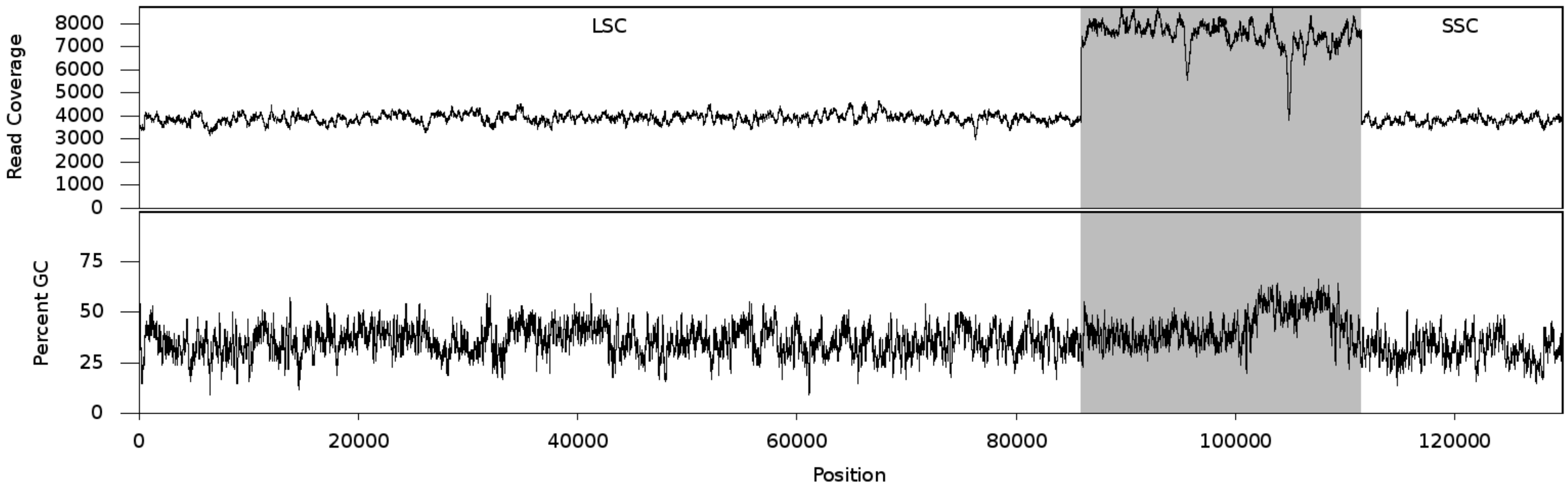

Solanum vernei PI 458370

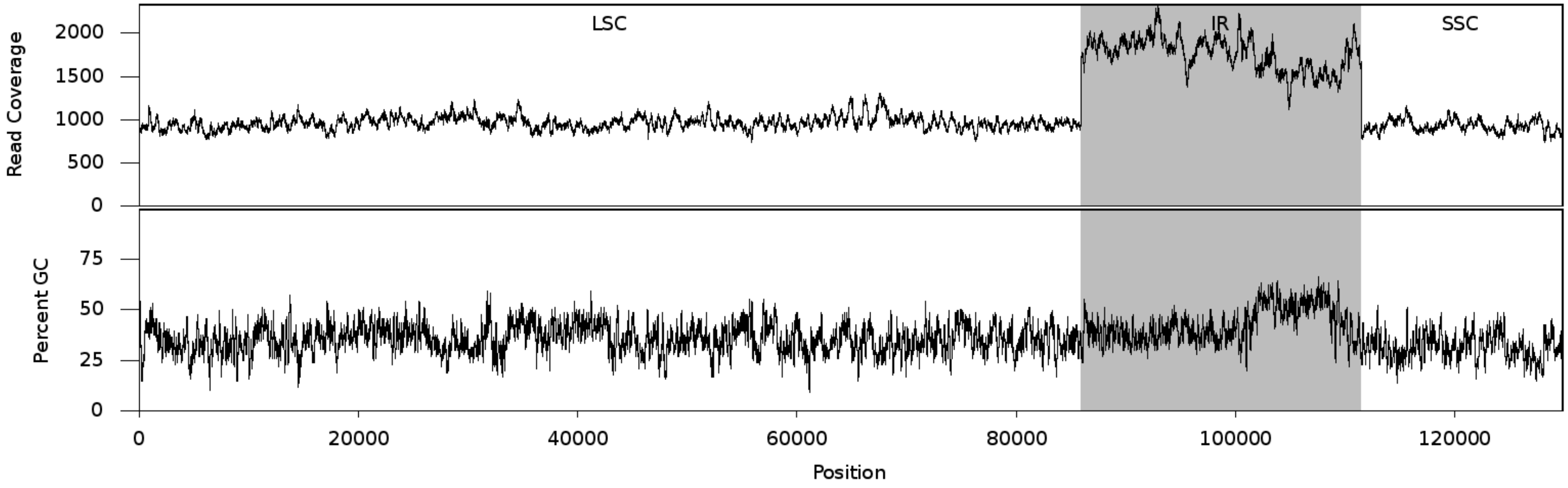

Solanum vernei PI 473303

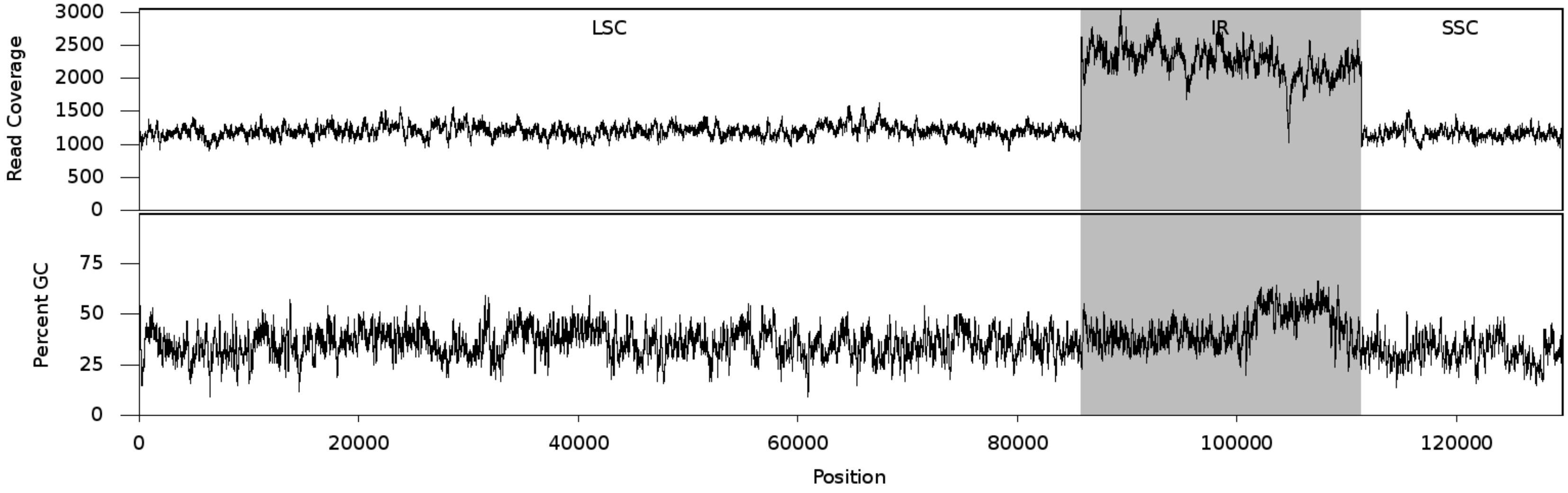

Solanum vernei PI 473309

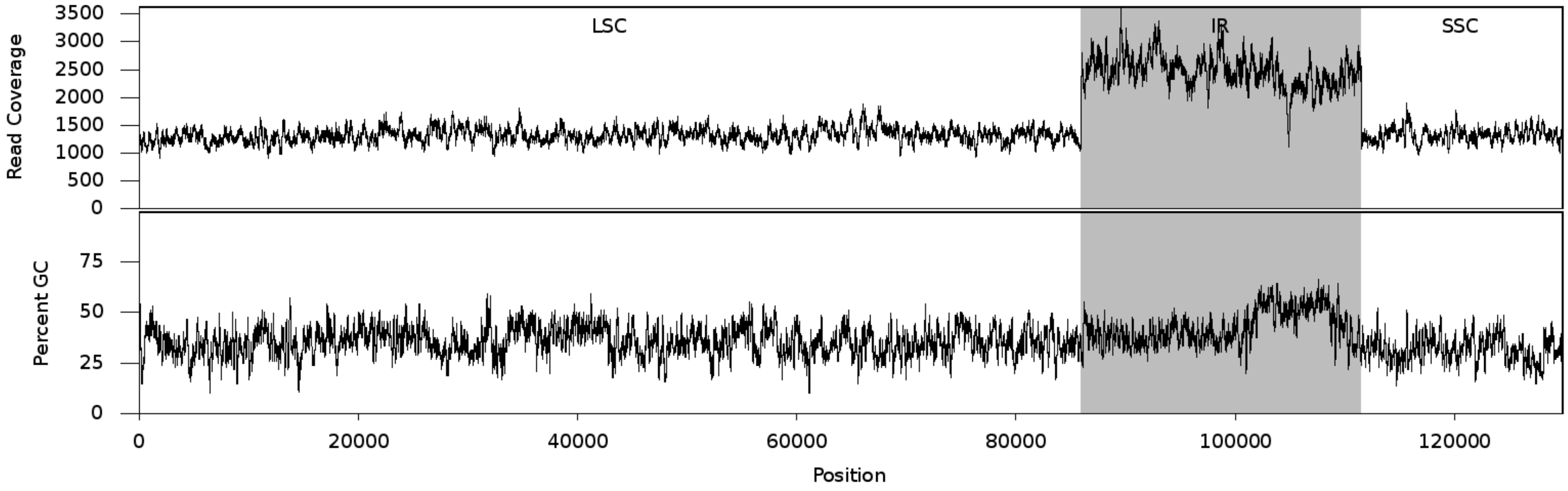

Solanum vernei PI 500070

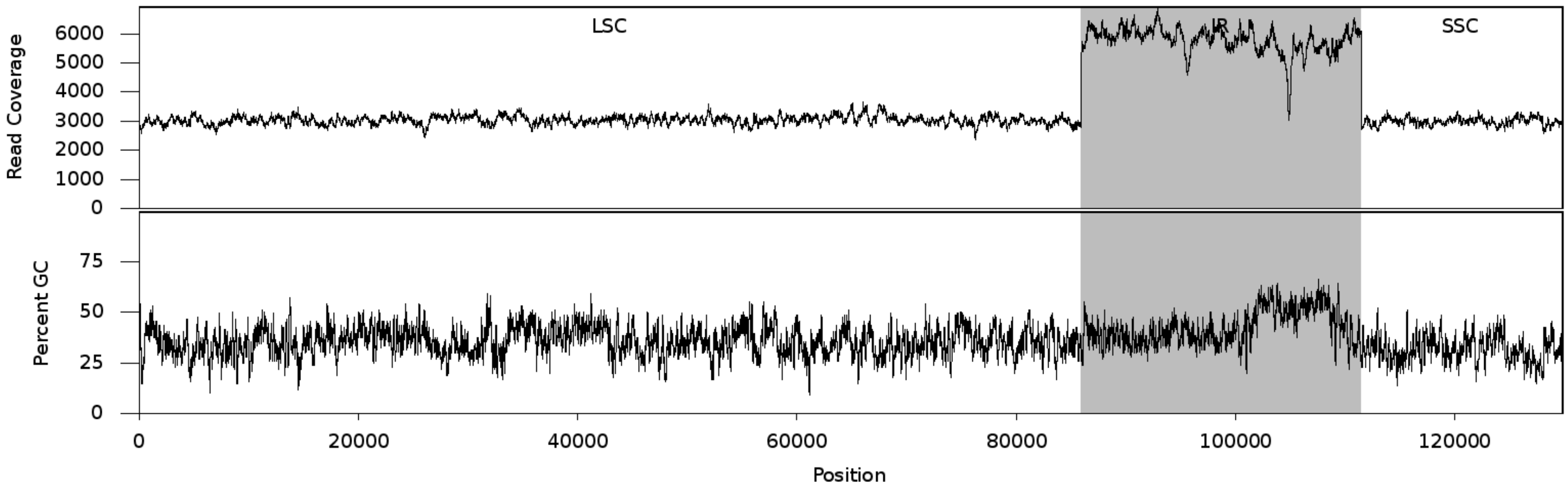

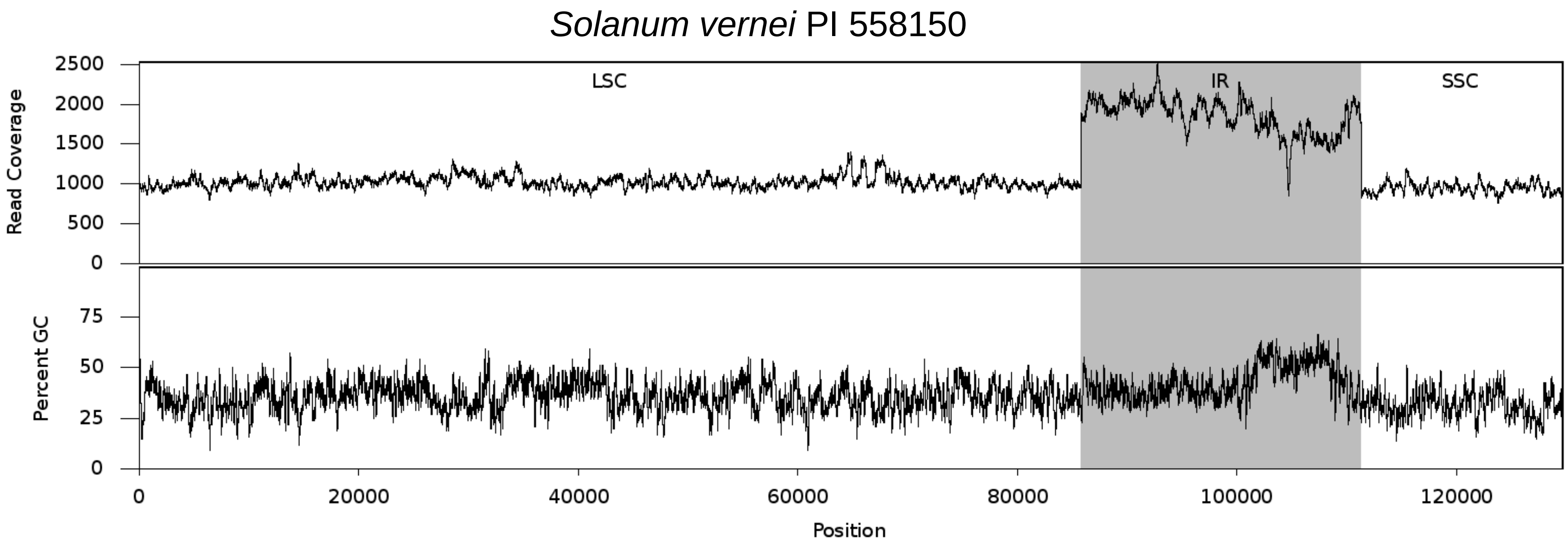
Solanum vernei PI 558150
LSC
IR
SSC
Read Coverage
2500
2000
1500
1000
500
0
Percent GC
75
50
25
0
0
20000
40000
60000
80000
100000
120000
Position

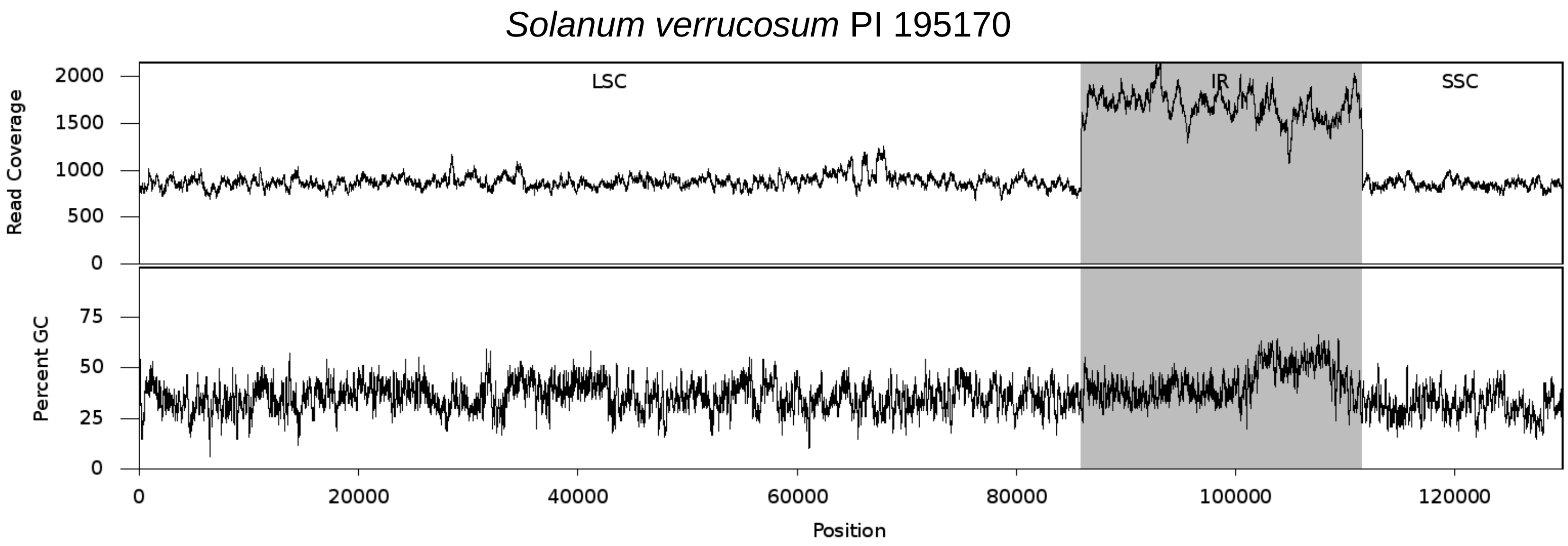
Solanum verrucosum PI 195170
LSC
IR
SSC
Read Coverage
2000
1500
1000
500
0
Percent GC
75
50
25
0
0
20000
40000
60000
80000
100000
120000
Position

Solanum verrucosum PI 275256

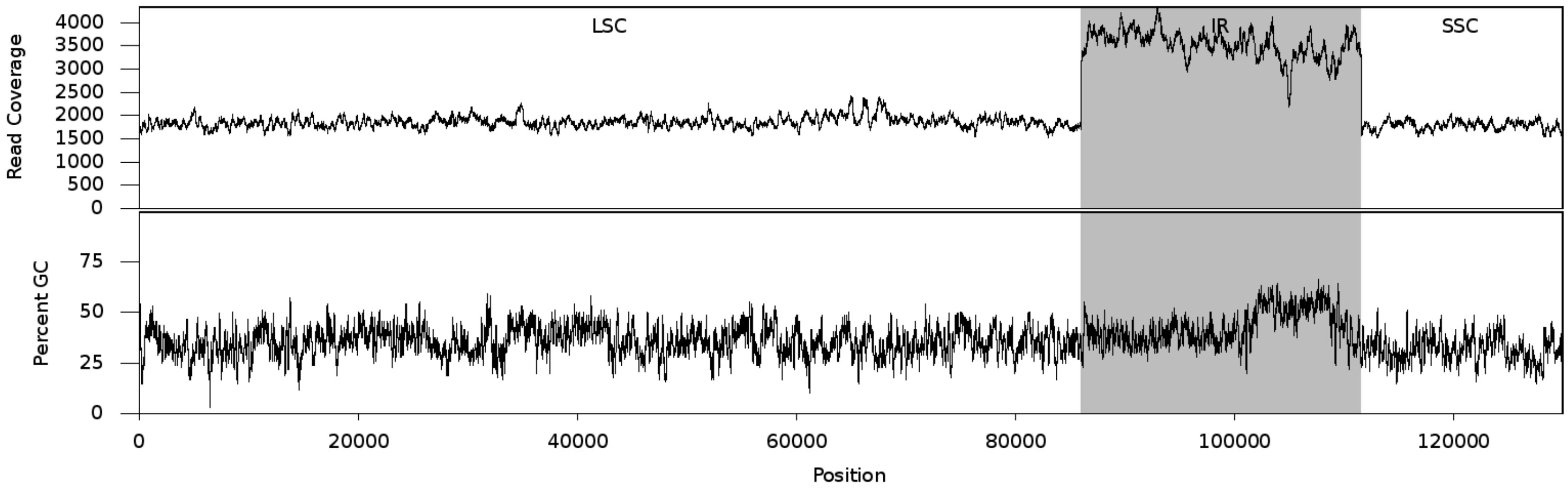

Solanum verrucosum PI 275260

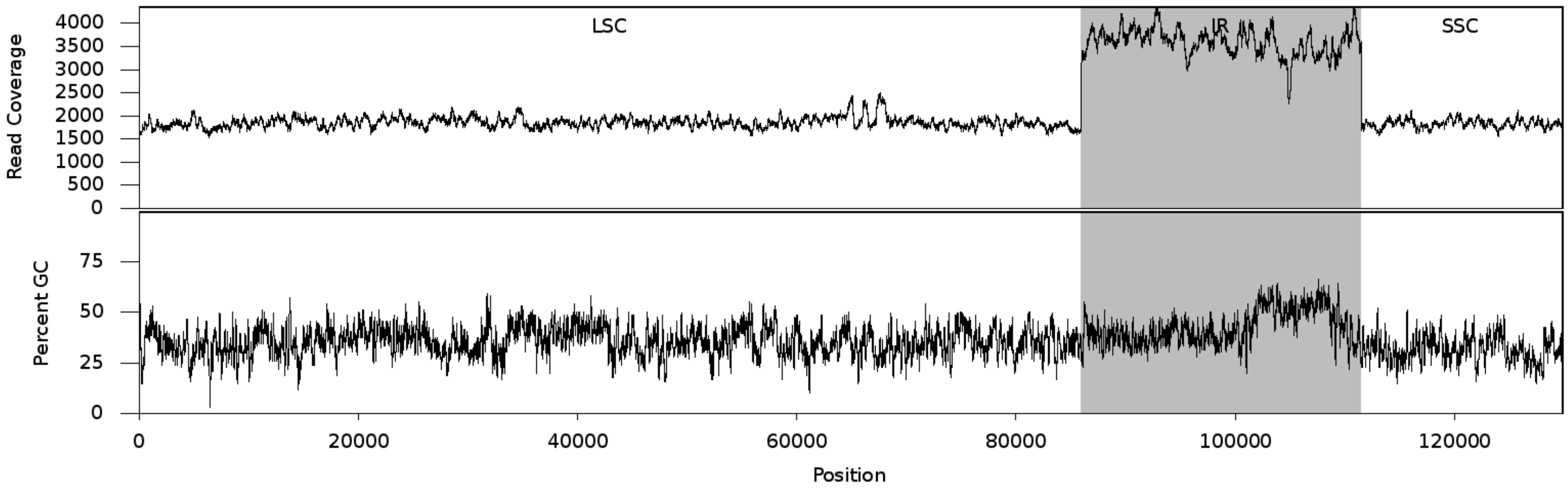

Solanum verrucosum PI 498010

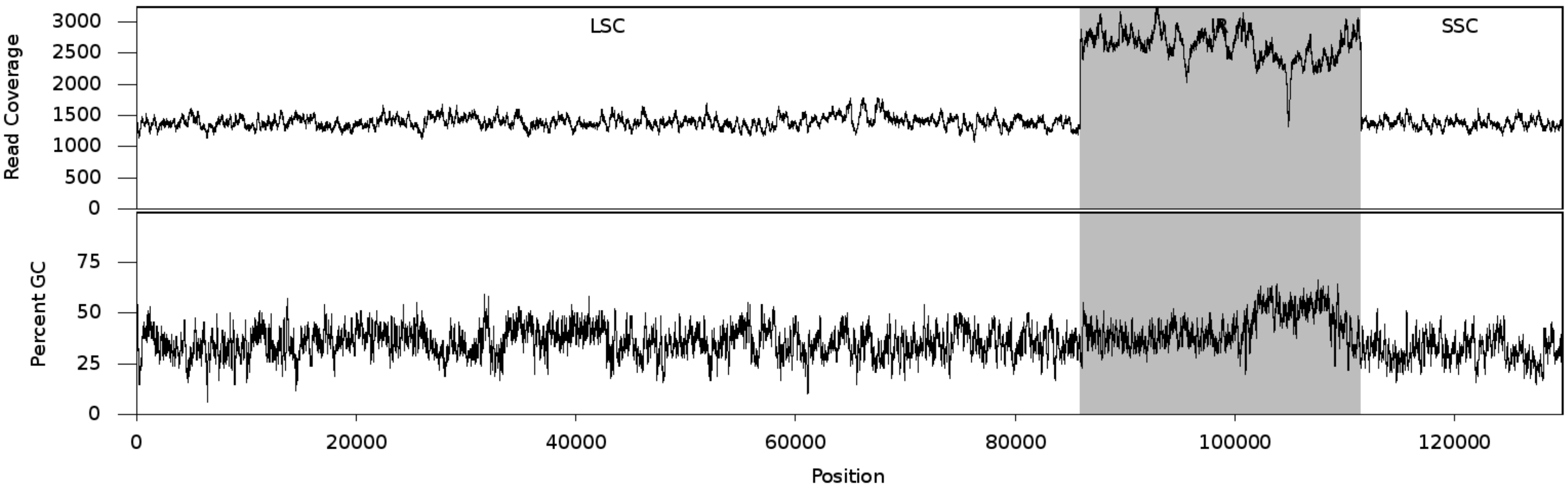

Solanum verrucosum PI 498061

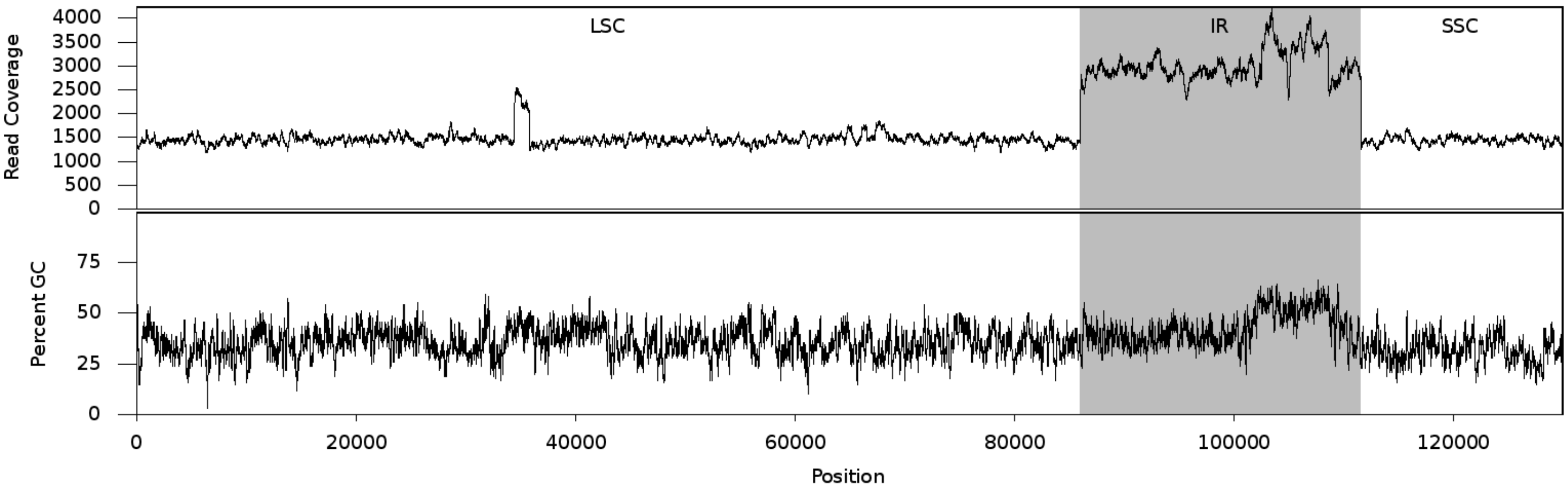

Solanum verrucosum PI 545745

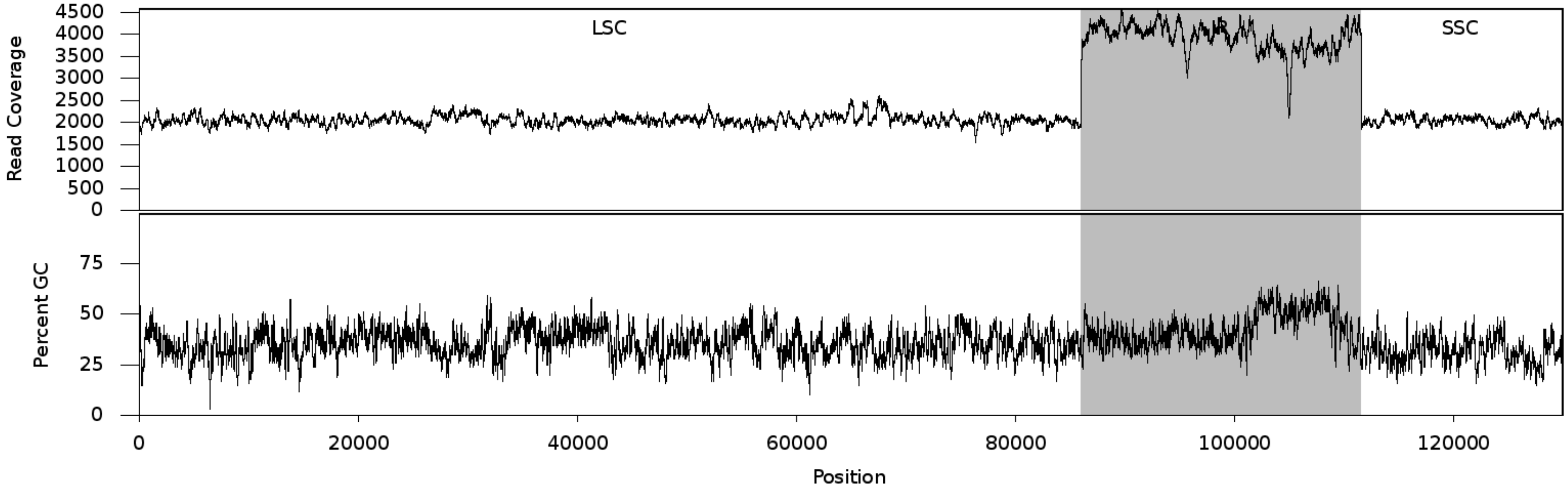

Solanum verrucosum PI 545747

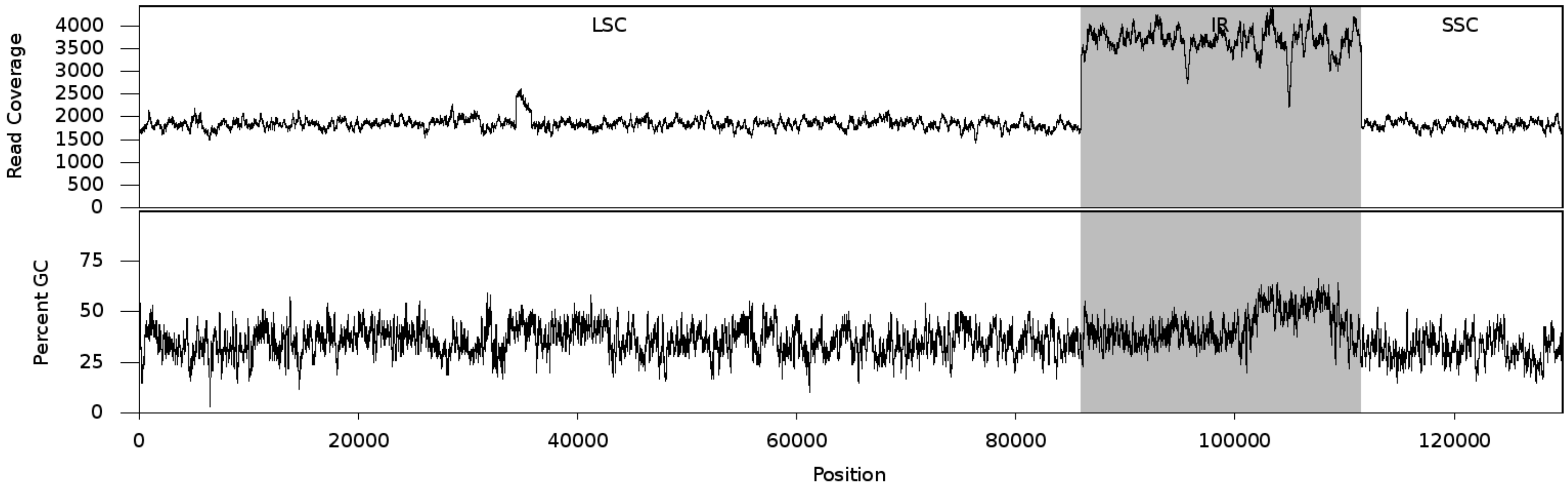

Solanum verrucosum PI 558463

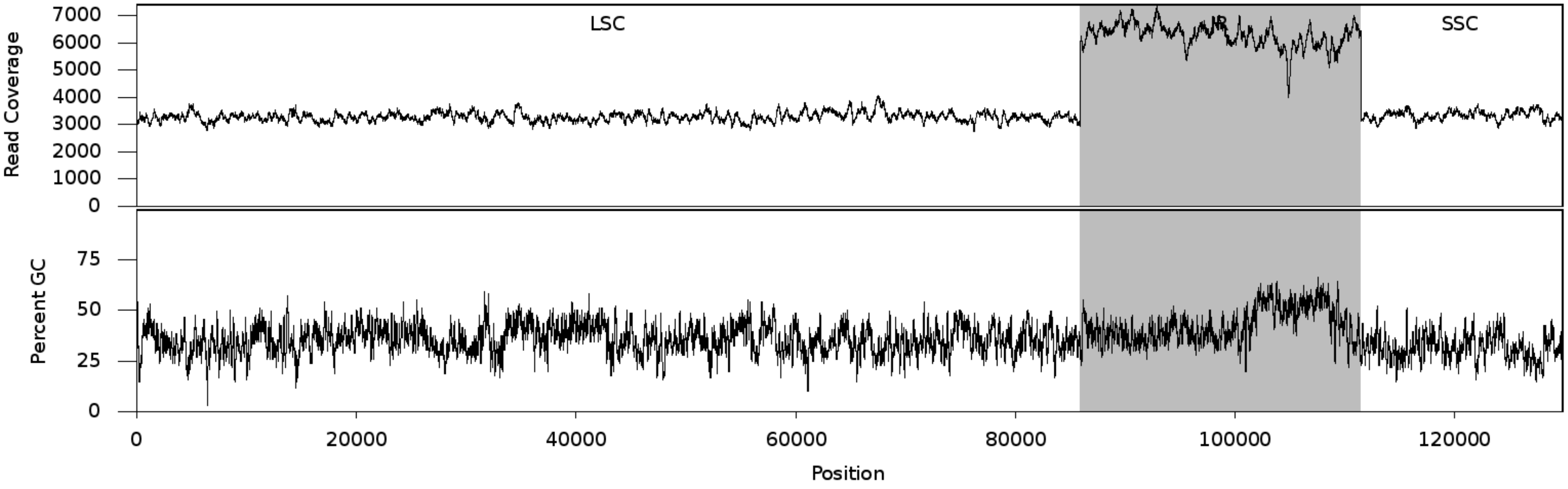

Solanum verrucosum PI 558488

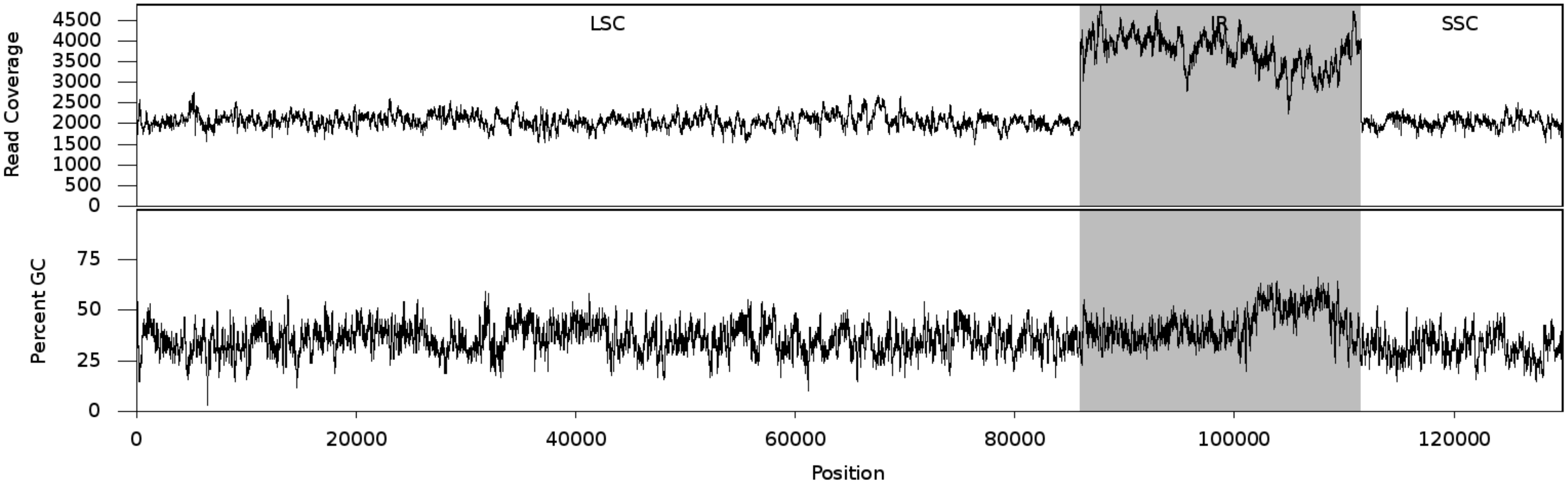

Solanum violaceimarmoratum PI 473396

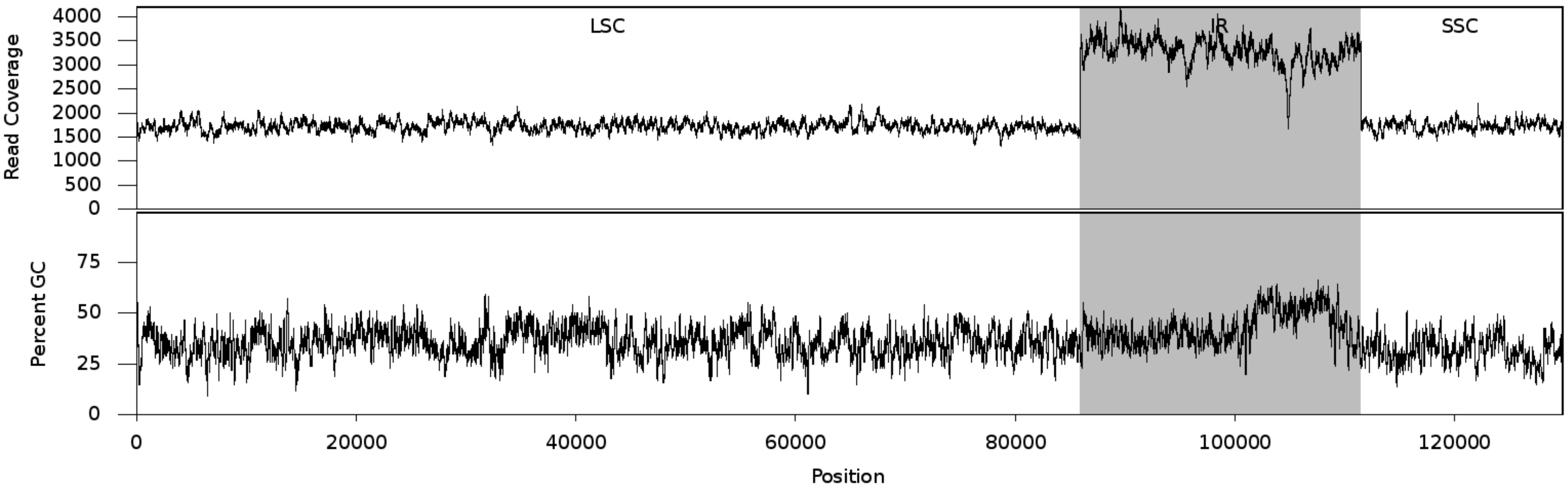

Solanum violaceimarmoratum PI 473398

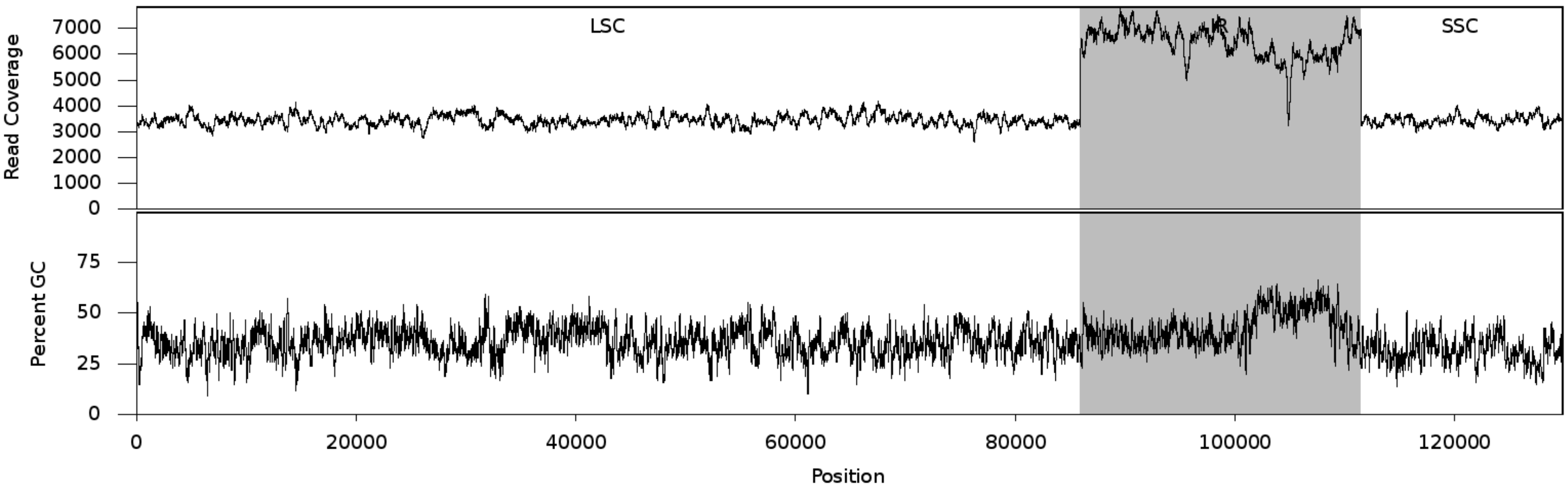

Solanum violaceimarmoratum PI 498296

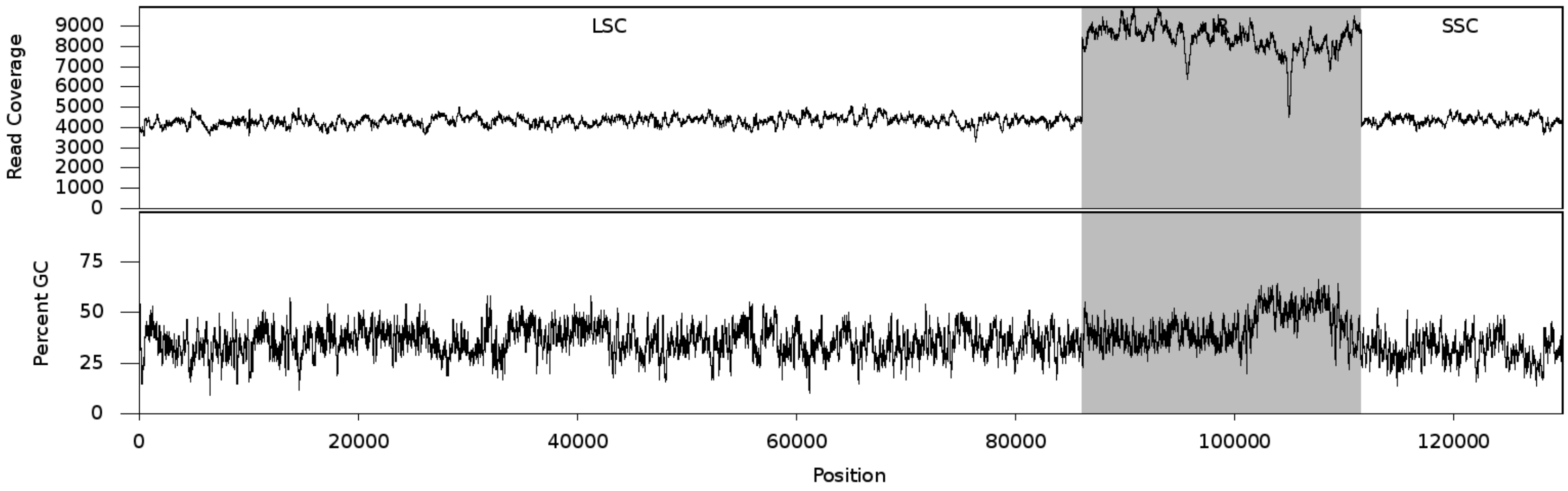

Supplemental Figure 2. ML tree

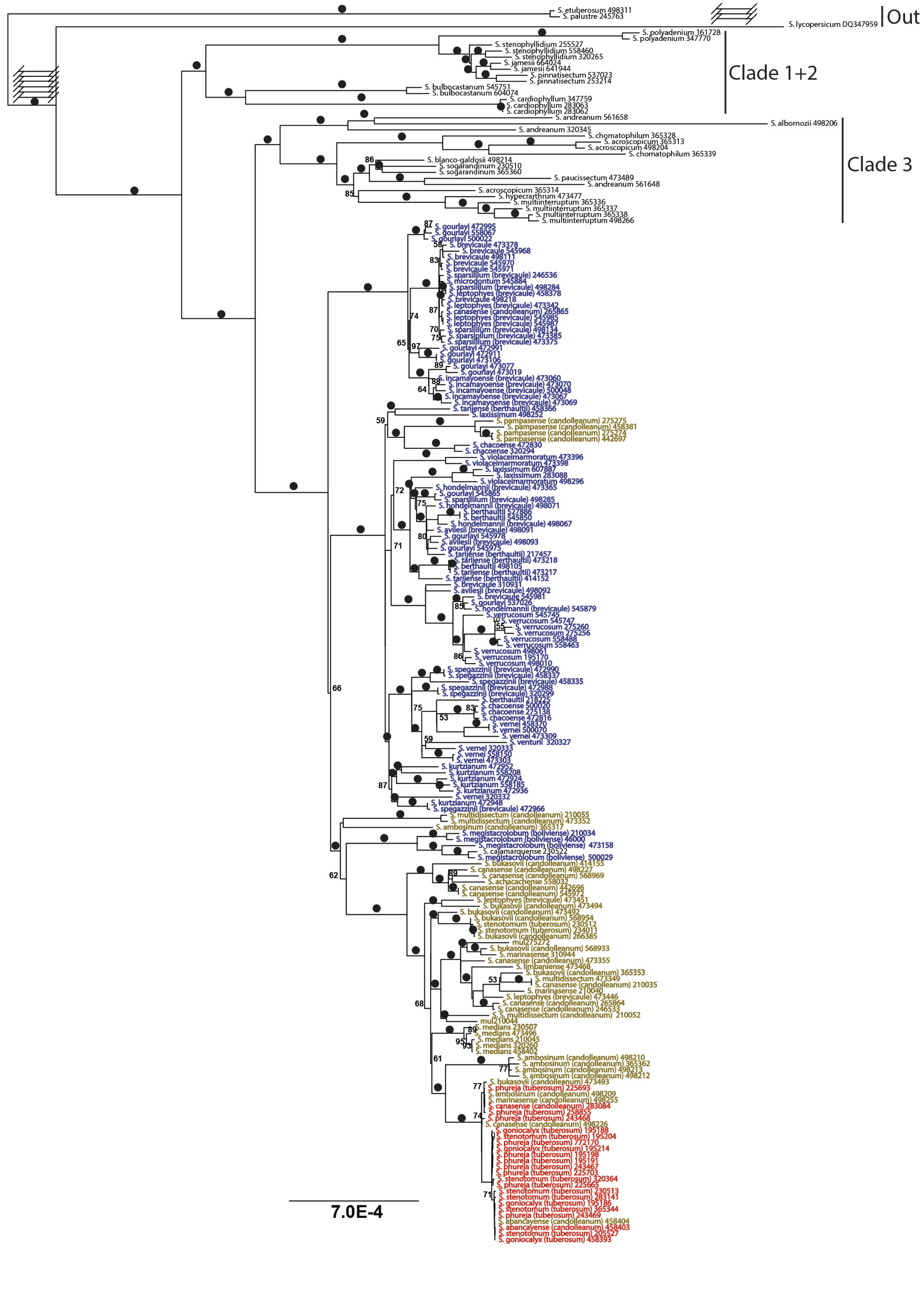

Supplement: Supplementary file 1 — Supplementary Tables 1–5; Figures 1,2. [file 41598_2019_40790_MOESM1_ESM.pdf]
